# Multi-system diseases and death trajectory of metabolic dysfunction-associated fatty liver disease: findings from the UK Biobank

**DOI:** 10.1186/s12916-023-03080-6

**Published:** 2023-10-20

**Authors:** Yu Jia, Dongze Li, Yi You, Jing Yu, Wenli Jiang, Yi Liu, Rui Zeng, Zhi Wan, Yi Lei, Xiaoyang Liao

**Affiliations:** 1 General Practice Ward/International Medical Center Ward, General Practice Medical Center, West China Hospital, Sichuan University, 37 Guoxue Road, Chengdu, 610041 China; 2 Department of Emergency Medicine, West China Hospital, Sichuan University, Chengdu, Sichuan China; 3 School of Computer Science, Sichuan University, Chengdu, Sichuan China; 4 Department of Cardiology, West China Hospital, West China School of Medicine, Sichuan University, Chengdu, Sichuan China

**Keywords:** Metabolic dysfunction-associated fatty liver disease, Disease trajectory, Mortality, Nonalcoholic fatty liver disease

## Abstract

**Background:**

Metabolic dysfunction-associated fatty liver disease (MAFLD) is a newly defined condition encompassing hepatic steatosis and metabolic dysfunction. However, the relationship between MAFLD and multi-system diseases remains unclear, and the time-dependent sequence of these diseases requires further clarification.

**Methods:**

After propensity score matching, 163,303 MAFLD subjects and 163,303 matched subjects were included in the community-based UK Biobank study. The International Classification of Diseases, Tenth Revision (ICD-10), was used to reclassify medical conditions into 490 and 16 specific causes of death. We conducted a disease trajectory analysis to map the key pathways linking MAFLD to various health conditions, providing an overview of their interconnections.

**Results:**

Participants aged 59 (51–64) years, predominantly males (62.5%), were included in the study. During the 12.9-year follow-up period, MAFLD participants were found to have a higher risk of 113 medical conditions and eight causes of death, determined through phenome-wide association analysis using Cox regression models. Temporal disease trajectories of MAFLD were established using disease pairing, revealing intermediary diseases such as asthma, diabetes, hypertension, hypothyroid conditions, tobacco abuse, diverticulosis, chronic ischemic heart disease, obesity, benign tumors, and inflammatory arthritis. These trajectories primarily resulted in acute myocardial infarction, disorders of fluid, electrolyte, and acid–base balance, infectious gastroenteritis and colitis, and functional intestinal disorders. Regarding death trajectories of MAFLD, malignant neoplasms, cardiovascular diseases, and respiratory system deaths were the main causes, and organ failure, infective disease, and internal environment disorder were the primary end-stage conditions. Disease trajectory analysis based on the level of genetic susceptibility to MAFLD yielded consistent results.

**Conclusions:**

Individuals with MAFLD have a risk of a number of different medical conditions and causes of death. Notably, these diseases and potential causes of death constitute many pathways that may be promising targets for preventing general health decline in patients with MAFLD.

**Supplementary Information:**

The online version contains supplementary material available at 10.1186/s12916-023-03080-6.

## Background

Nonalcoholic fatty liver disease (NAFLD) is characterized by the presence of excessive hepatic steatosis (> 5%) in individuals without heavy drinking or viral hepatitis [1]. It is the main cause of liver disease worldwide, with an estimated prevalence of 25–45% [2]. The prevalence of NAFLD has increased rapidly owing to its strong association with diabetes, metabolic syndrome, and obesity [2]. To integrate the current understanding of patient heterogeneity, more accurately reflect pathogenesis, and strengthen hierarchical patient management, a group of experts proposed a new term in 2020: metabolic dysfunction-associated fatty liver disease (MAFLD) [3].

NAFLD-related adverse outcomes have been well documented in a previous study [4]. In recent years, populations with MAFLD have been shown to have a higher risk of both intrahepatic and extrahepatic system diseases [5], such as hypertension, diabetes, atherosclerosis, coronary heart disease, dementia, sarcopenia, chronic kidney disease, enteritis, cirrhosis, and tumors [6–12]. Our understanding of the relationship between MAFLD and multi-system diseases remains incomplete. Importantly, the occurrence of these diseases may have a time-dependent sequence that constitutes a complex disease network derived from MAFLD. Because no specific treatment for MAFLD exists, it is crucial to decipher the crosslinking of the MAFLD disease network and propose interventions to prevent health decline. Therefore, a comprehensive understanding of the disease trajectories of MAFLD is highly important, which may help investigate the critical link between MAFLD and related multi-system diseases and deaths from a holistic perspective.

Disease trajectory analysis, proposed by Jensen et al., is a new visual method to explore the development of diseases over the course of a population’s lifespan [13]. By visualizing the networks of emerging diseases, the time sequence among the initial, secondary, and terminal diseases is displayed, providing an approach to investigate the complex causal relationship and sequential pattern of multiple diseases. Disease trajectory analysis has been used to research temporal, population-wide disease progression patterns in seven million cohorts [14]. Other researchers have applied disease trajectories to analyze the sequential progression of a specific disease, such as depression and breast neoplasms [15, 16]. Therefore, it is valuable to apply this innovative trajectory analysis method to visualize MAFLD disease trajectories.

The UK Biobank cohort study, with a population of half a million, has fully evaluated MAFLD using a non-invasive approach and collected data on disease diagnosis and causes of death records using International Classification of Diseases, Tenth Revision (ICD-10) codes since 2006. Thus, it provides ideal data resources and sufficient follow-up time to analyze the disease trajectory network of MAFLD. Therefore, the purpose of this study was to investigate the key pathway of MAFLD leading to subsequent multi-system diseases and cause-specific death and to draw a panorama of the disease tree diagram through disease trajectory analysis based on the UK Biobank community cohort.

## Methods

### Study design and population

The UK Biobank study design was presented in a previous study [17]. From 2006 to 2010, the UK Biobank research cohort recruited 502,655 adults in the UK aged 39 to 69 years via the UK National Health Service through a postal invitation to recruit a representative UK population as far as possible [18]. Participants completed a 90-min comprehensive assessment, including demographic characteristics, medical history, and physical measurements, using an electronic questionnaire. Biological samples were obtained, and laboratory examinations were performed at the time of enrolment. The details of data collection are available on the website http://www.ukbiobank.ac.uk. Data and materials were obtained from the website https://ukbiobank.dnanexus.com/panx/projects. The National Research Ethics Committee approved the experimental protocol. All participants signed written informed consent forms; the research was conducted according to the ethical guidelines of the Helsinki Declaration, and the protocol was approved by each institutional review board. Data usage was approved by the Human Ethical Committee of the West China Hospital of Sichuan University.

**Fig. 1:**
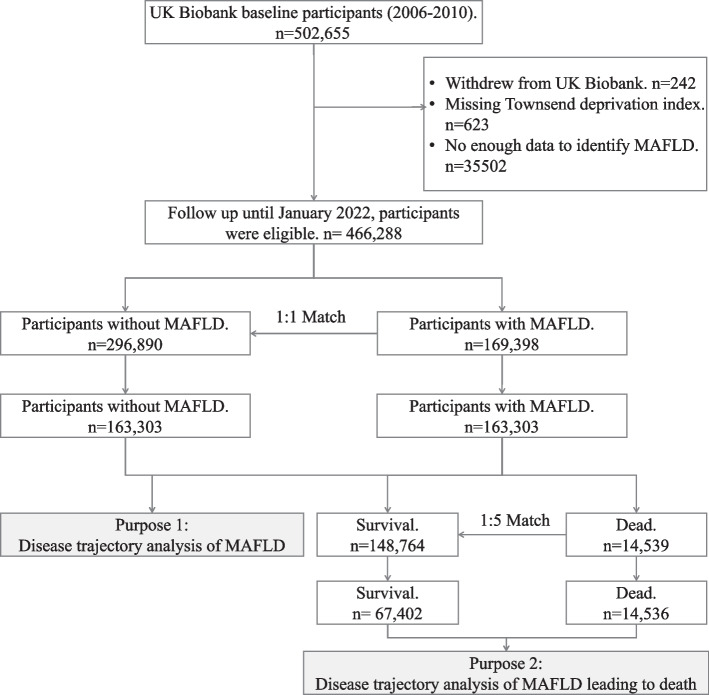
Study flow chart. MAFLD, metabolic dysfunction-associated fatty liver disease

We enrolled 502,413 active participants in this study. To analyze the disease trajectory of MAFLD, subjects who withdrew from the UK Biobank study (*n* = 242) were missing the Townsend deprivation index (*n* = 623), or missing data to identify MAFLD (*n* = 35,502) were excluded. Thus, 466,288 participants were included in this study. To reduce confounding biases in this observational study, we conducted propensity score matching (1:1) according to age, sex, and the Townsend deprivation index (assigned by the postcode of participants’ location, which reflected the level of material deprivation the participant experiences) for participants with and without MAFLD. In total, 326,606 participants were included in the analysis of MAFLD-related trajectories (Fig. 1). Furthermore, to analyze the cause-specific death trajectory of MAFLD, age, sex, and Townsend deprivation index, match-surviving (*n* = 67,402) and dead (*n* = 14,536) individuals were included in the MAFLD group (Fig. 1). A similar investigation was conducted to analyze the disease trajectory of genetic susceptibility to MAFLD.

### Definition of MAFLD

MAFLD was defined based on international expert consensus [3]. MAFLD is defined as hepatic steatosis plus one of the following conditions: (1) type 2 diabetes mellitus, (2) overweight/obesity (body mass index (BMI) ≥ 25 kg/m^2^), or (3) metabolic abnormality including any of the two: insulin resistance (not collected in the UK Biobank study), prediabetes (fasting glucose ≥ 100 mg/dl or Hemoglobin A1c ≥ 5.7%), low high-density lipoprotein cholesterol (< 1.03 mmol/L for males; < 1.29 mmol/L for females), hypertriglyceridemia (≥ 1.7 mmol/L), hypertension (≥ 130/85 mmHg or use of antihypertensive medication), and increased waist circumference (≥ 102 cm for males; ≥ 88 cm for females).

Hepatic steatosis was assessed using the fatty liver index (FLI) according to the formula for waist circumference, gamma-glutamyl transferase, triglycerides, and BMI [19]. FLI ≥ 60 was used to diagnose hepatic steatosis, which yielded a sensitivity of 87% and specificity of 86% [19]. The FLI has been verified to be adequate in diagnosing hepatic steatosis, achieving a sensitivity of 81% and specificity of 90% in a meta-analysis based on a multiethnic population study [20].

### Advanced liver fibrosis assessment

Advanced liver fibrosis was assessed via NAFLD fibrosis score using the following formulas: − 1.675 + [0.037 × age (years)] + [0.094 × BMI (kg/m^2^)] + [1.13 × T2D (yes = 1, no = 0)] + [0.99 × AST/ALT ratio] − [0.013 × platelet count (10^9^/L)] − [0.66 × albumin (g/dL)]. For predicting advanced fibrosis, the cutoff of the NAFLD fibrosis score is − 1.455, which achieved a negative predictive value of 93% according to previous reports [21].

### Diagnoses of medical conditions and death

The diagnosis of each medical disease for the population in the UK Biobank study originated from the main and secondary diagnostic data during hospitalization, which were recorded using ICD-10 codes. ICD-10 codes for pregnancy, childbirth, and unclassified symptoms or signs were excluded. All medical diagnoses were made using the three-digit ICD-10 codes. We combined diagnoses with high clinical or biological similarity, reducing the number of diagnostic categories from 1645 to 490. For example, we considered all cases of typhoid and paratyphoid fever, cholera, and shigellosis, which are defined as bacterial intestinal infections. For patients with multiple disease records, only the first record and the date of the first hospital visit were used as the diagnosis dates. The discharge coding accuracy of medical conditions of inpatients in the UK was verified, with a primary diagnosis accuracy of 96% and an average diagnostic accuracy of 80.3% [22]. Original and combined ICD-10 codes can be found in Additional file 1. Detailed codes, definitions, and other related classification information of various diseases can be queried at the official website of the World Health Organization (https://icd.who.int/browse10/2019/en).

The cause of death of participants in the UK Biobank was defined as the primary or secondary cause of death recorded in the mortality data. According to ICD-10, there were 16 causes of death.

### Genotyping

Genotyping and imputation originated from the UK BiLEVE and UK Biobank Axiom arrays in the UK Biobank study; other detailed information has been provided elsewhere [23]. Single-nucleotide polymorphisms (SNPs) were selected from a recently published genome-wide association studies for MAFLD, including MBOAT7 rs641738 [24], GCKR rs1260326 [25], TM6SF2 rs58542926 [26], and PNPLA3 rs738409 [27]. All SNPs were coded 2, 1, and 0 for homozygous, heterozygous, and noncarriers, respectively. The polygenic risk scores (PRS) were calculated as follows: (β1 × SNP1 + β2 × SNP2 + … + βn × SNPn) × (total number of SNPs/sum of the β-coefficients), where β was derived from the original genome-wide association studies. A PRS was constructed to summarize the impact of genetic predisposition on MAFLD [6]. The PRS ranged from 0 to 8 and was subsequently categorized into tertiles: low (< 0.76 points), intermediate (0.76–2.35 points), and high (> 2.35 points) genetic risk.

### Danish Disease Trajectory Browser

The database for the Danish Disease Trajectory Browser is the Danish National Patient Register, which includes 7 million individuals from 1994 to 2018, and covers 122 million hospital admissions with 1777 unique ICD-10 codes recorded [14]. The address of the Danish Disease Trajectory Browser is published on an online analysis platform (http://dtb.cpr.ku.dk/). Because no ICD-10 code exists for MAFLD, the ICD-10 codes K76 (nonalcoholic liver disease) and K70 (alcoholic liver disease) were used to validate the reproducibility of the disease trajectory of MAFLD.

### Statistical analysis

#### Trajectory analysis

Referring to methods used in previous studies [14–16], we performed a disease trajectory analysis of MAFLD (Additional file 2: Fig. S1). First, a phenome-wide association analysis (PheWAS) using Cox regression was conducted to identify the disease risk of participants with MAFLD compared to that of matched controls from all 490 disease categories. To analyze each disease outcome in the Cox regression model, a subcohort was formed by excluding participants with a history of the disease outcome at baseline. To achieve adequate statistical power, analyses were limited to disease occurrence in > 1% of the MAFLD participants (*n* = 1633). According to the results of the Cox regression analysis, diseases with a hazard ratio (HR) > 1 and *p* < 0.05/n (Bonferroni-corrected threshold; *n*: number of disease categories) were retained. Second, we analyzed all possible disease 1 (D1) and disease 2 (D2) pairs calculated by n*(*n* − 1), and pairs that occurred in > 0.5% of MAFLD participants (*n* = 816) were included. To ensure logical temporal order, we conducted binomial tests to determine whether more MAFLD individuals (> 50%) had D2 diagnosed later than D1 among those with both D1 and D2 diagnoses. Disease pairs with *P* < the Bonferroni-corrected threshold were considered statistically significant. Third, we set D1 as the exposure and D2 as the outcome, and a logistic regression model was used to ensure an association between D1 and D2 in all disease pairs. Fourth, disease pairs with a significantly increased risk of D2 after D1 (odds ratio [OR] > 1; *P* < Bonferroni-corrected threshold) were confirmed.

To analyze the disease trajectory of MAFLD leading to death, we performed similar steps (Additional file 2: Fig. S2). Briefly, PheWAS was conducted to investigate the possible causes of death in MAFLD. Subsequently, diseases such as exposure and death were set as outcomes, and the PheWAS was conducted further to identify the association between medical conditions (selected from the first step) and each cause of death. Next, all possible D1 and D2 pairs were calculated using *n* × (*n* − 1) for each cause of death. Finally, binomial tests and logistic regression analyses were conducted to confirm the D1 and D2 pairs with a significantly increased risk of D2 after diagnosis of D1. The detailed steps of the trajectory analysis are described in Additional file 3. The codes for this study are shared at https://github.com/youyialex/MAFLD.

A tree diagram of the disease trajectory was drawn using overlying disease pairs. For example, disease pairs D1 to D2 and D2 to D3 were combined in trajectory D1 to D2 to D3, and the connecting line from D1 to D3 could be deleted.

#### Subgroup and sensitivity analyses

To analyze whether the disease phenotype of MAFLD was consistent in different sexes, males and females were separated. Subsequently, to investigate the impact of alcohol, obesity, liver fibrosis, and genotyping on the disease phenotype of MAFLD, subgroups were established based on whether individuals were overweight/obese (BMI ≥ 25 kg/m^2^), daily consuming excessive alcohol (≥ 20 g for women and ≥ 30 g for men), carrying risk alleles, or had liver fibrosis.

MAFLD may be caused or complicated by other diseases, which may lead to subsequent diseases in trajectories unrelated to MAFLD. Therefore, we performed a sensitivity analysis in which PheWAS was performed after excluding the population with the same disease category (consistent with the 16 categories of cause of death) from the baseline in the subcohort. For example, to investigate the relationship between MAFLD and heart failure, participants with any cardiovascular disease at baseline were excluded.

A similar study flow path was implemented for the trajectory analysis of genetic susceptibility to MAFLD (Additional file 2: Fig. S3-5). Individuals in the lowest tertile of the PRS were defined as controls, and those in the highest tertile of the PRS were defined as those exposed to MAFLD.

A two-tailed *p*-value < 0.05/number of disease categories for Bonferroni corrections was considered significant for all tests. All statistical analyses were performed using R software 4.2.3 Python version 3.11.2 and Cytoscape Desktop version 3.10.0.

## Results

### Baseline characteristics

In the UK Biobank study, 326,606 participants were included after propensity score matching according to age, sex, and the Townsend deprivation index. Among those, 163,303 participants were determined to have MAFLD (Fig. 1). The baseline characteristics of the patients are shown in Table 1. These participants were predominantly males (62.5%), had a mean age of 59 (51–64) years, a median BMI of 27.7 (24.9–31.0) kg/m^2^, and a mean follow-up period of 12.9 years.

**Table 1:** Basic characteristics of the participants with and without MAFLD after propensity score matching

Characteristics	Total (*N* = 326,606)	Non-MAFLD (*N* = 163,303)	MAFLD (*N* = 163,303)
Male, *n* (%)	203,980 (62.5)	102,019 (62.5)	101,961 (62.4)
Age (years)	59 (51–64)	59 (51–64)	59 (51–63)
Deprivation Index	− 2.02 (− 3.58–0.79)	− 2.03 (− 3.60–0.77)	− 2.01 (− 3.57–0.80)
Alcohol intake (g/day)	10 (0.66–22.86)	10 (1.43–21.43)	11.43 (0–25.71)
BMI (kg/m^2^)	27.65 (24.94–31.01)	25.03 (23.27–26.77)	30.86 (28.59–33.87)
Follow-up time (years)	12.92 (12.18–13.64)	12.91 (12.19–13.63)	12.92 (12.16–13.65)

Data are expressed as *n* (%) and median (25th–75th). Propensity score matching was conducted according to age, sex, and Townsend deprivation index (assigned by the postcode of participant location, which reflects the level of social deprivation in which the participant lives) for subjects with and without MAFLD. The recording of alcohol intake is based on recalling and estimating the average total amount of red wine, liquor, beer, and fruit wine consumed per week or month

*BMI* body mass index, *MAFLD* metabolic dysfunction-associated fatty liver disease

### Medical conditions and causes of death related to MAFLD

**Fig. 2:**
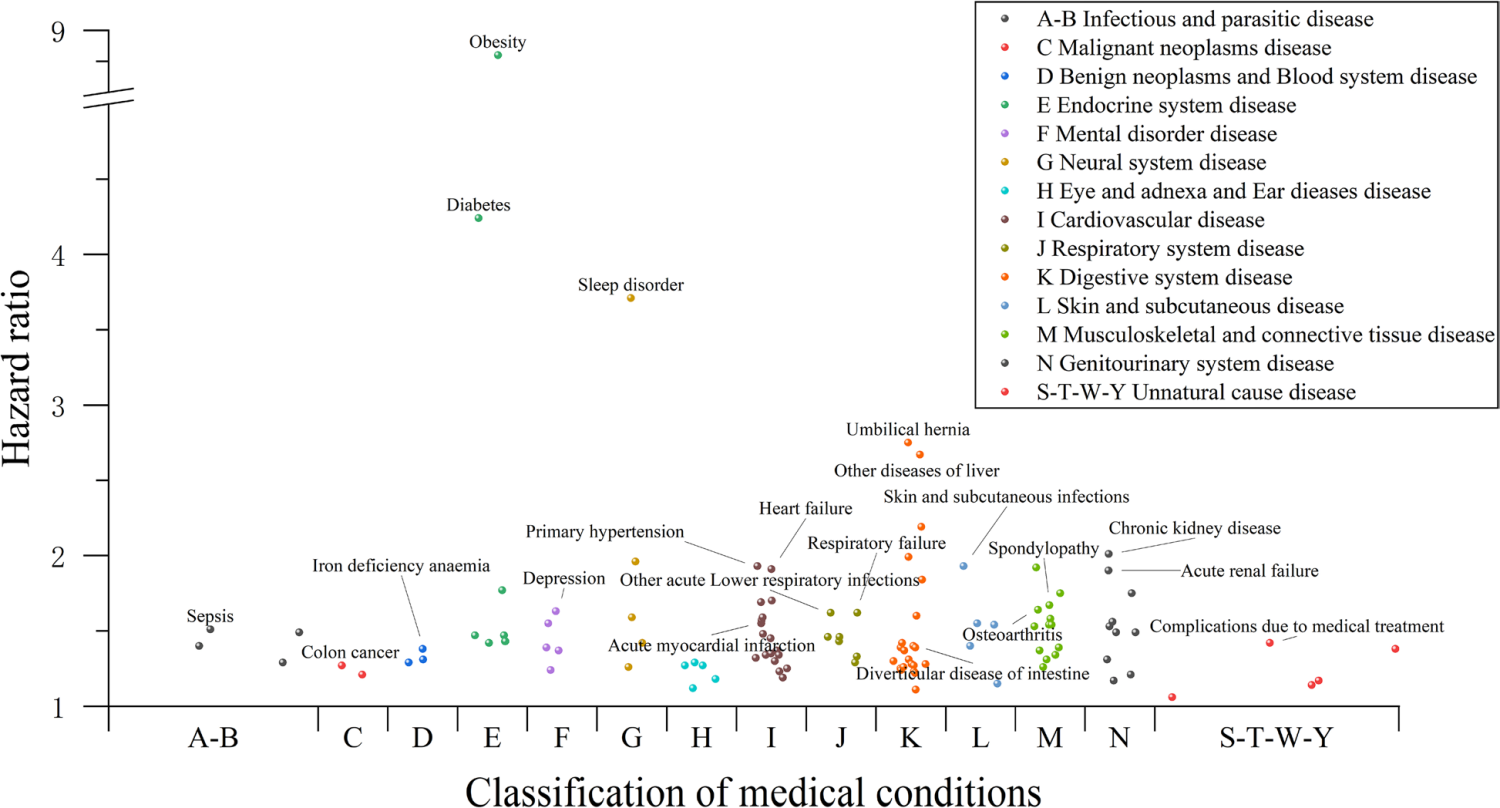
PheWAS using Cox regression was conducted to investigate the relationship between MAFLD and subsequent medical conditions. The *x*-axis shows the disease categories according to the combined ICD-10 codes. The *y*-axis shows the hazard ratios. Each point represents a medical condition that is significantly related to MAFLD after Bonferroni correction

After disease limiting occurred in ≥ 1% of MAFLD participants and conducting PheWAS using Cox regression models, participants with MAFLD had a higher risk of 113 medical conditions out of 490 total medical conditions (Fig. 2). Similar to results from previous studies, MAFLD was associated with almost all systemic diseases (Additional file 4: Table S1), such as sepsis (HR 1.51, 95% CI 1.45–1.58), colon cancer (HR 1.27, 95% CI 1.18–1.35), benign tumors (HR 1.29, 95% CI 1.26–1.32), iron deficiency anemia (HR 1.38, 95% CI 1.33–1.43), diabetes (HR 4.24, 95% CI 4.11–4.37), depression (HR 1.63, 95% CI 1.58–1.68), sleep disorder (HR 3.71, 95% CI 3.50–3.93), acute myocardial infarction (HR 1.55, 95% CI 1.48–1.62), respiratory failure (HR 1.62, 95% CI 1.53–1.71), and diverticular disease of the intestine (HR 1.40, 95% CI 1.37–1.43).

Among the 16 categories of causes of death, eight causes of death were significantly associated with MAFLD (Table 2). The highest HR of causes of death was endocrine system death (HR 3.23, 95% CI 2.95–3.53), and the majority of individuals died because of malignant neoplasms (incidence (%): 7942/15484 (51.3%), HR 1.26, 95% CI 1.21–1.30).

**Table 2:** PheWAS using Cox regression was conducted to investigate the relationship between MAFLD and causes of death in males and females

Causes of death	Total (*N* = 326,606)		Female (*N* = 122,626)		Male (*N* = 203,980)	
	**No.**^**a**^	**HR (95% CI)**	**No.**^**a**^	**HR (95% CI)**	**No.**^**a**^	**HR (95% CI)**
Cardiovascular death	6025	1.64 (1.58–1.71)	1603	1.97 (1.81–2.14)	4422	1.55 (1.48–1.62)
Digestive system death	1409	1.81 (1.66–1.98)	441	2.30 (1.95–2.73)	968	1.65 (1.49–1.83)
Endocrine system death	2007	3.23 (2.95–3.53)	605	4.04 (3.38–4.83)	1402	2.97 (2.67–3.29)
Genitourinary system death	1203	2.05 (1.86–2.26)	383	2.49 (2.07–3.01)	820	1.89 (1.68–2.12)
Infectious and parasitic death	867	1.63 (1.46–1.80)	332	1.60 (1.35–1.90)	535	1.50 (1.31–1.71)
Malignant neoplasms death	7488	1.26 (1.21–1.30)	2556	1.28 (1.21–1.36)	4932	1.24 (1.19–1.30)
Respiratory system death	3606	1.21 (1.15–1.27)	1070	1.50 (1.36–1.65)	2536	1.12 (1.05–1.18)
Unnatural cause death	1083	1.21 (1.11–1.32)	280	1.45 (1.21–1.75)	803	1.14 (1.03–1.27)

After Bonferroni correction, a total of 8 causes of death were significantly associated with MAFLD

*HR* hazard ratio, *CI* confidence interval, *MAFLD* metabolic dysfunction-associated fatty liver disease

^**a**^Number of MAFLD participants who died due to the corresponding causes, including primary and secondary causes of death

### Temporal disease trajectories of MAFLD

According to the analysis shown in Additional file 2: Fig. S1, 353 disease pairs (D1–D2) were confirmed (Additional file 4: Table S2). The three largest OR for disease pairing were chronic ischemic heart disease leading to acute myocardial infarction (OR 85.25, 95% CI 52.08–139.55), angina pectoris leading to acute myocardial infarction (OR 10.78, 95% CI 8.53–13.61), and metastatic cancer leading to external causes of morbidity related to medical treatment (OR 5.88, 95% CI 5.11–6.77).

**Fig. 3:**
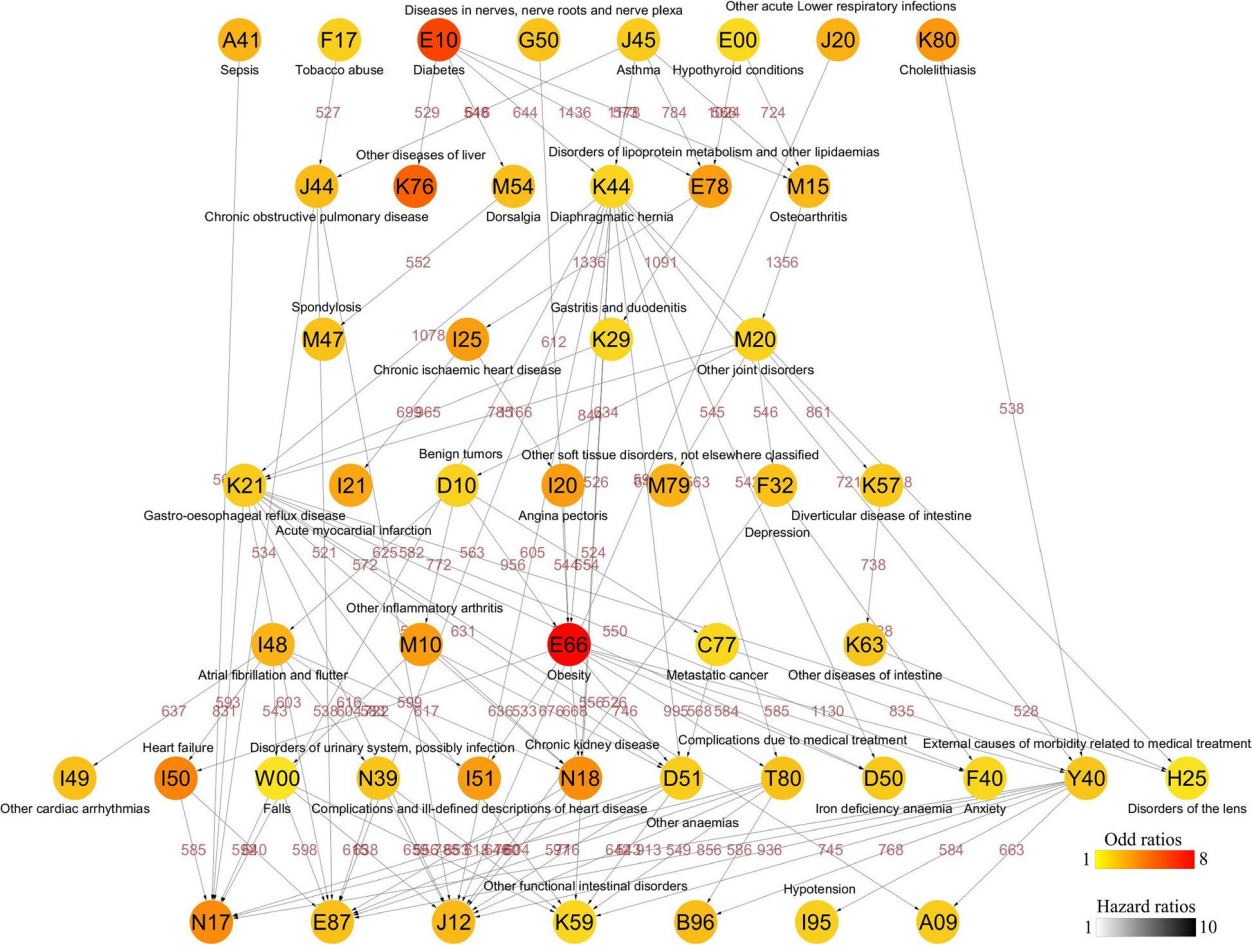
Overview map of the temporal disease trajectories of MAFLD. The codes in the circle correspond to combined ICD-10 codes, and their disease descriptions are provided. The color of the circle indicates the hazard ratios of the medical conditions when comparing MAFLD to non-MAFLD. The grayscale of the arrows presents the odds ratio of the temporal relationship of the two medical conditions in MAFLD individuals. The number above the arrow connecting two circles presents the number of disease pairs in MAFLD individuals. Detailed data can be found in Additional file 4: Table S1-S2

Figure 3 presents an overview of the complex temporal disease trajectories of MAFLD. In this complex MAFLD network, the original diseases in the tree diagram mainly included asthma, diabetes, hypothyroid conditions, and tobacco abuse. Furthermore, in the trajectory of MAFLD, the intermediate diseases that mediated most downstream diseases were diverticular diseases of the intestine (*n* = 26), chronic ischemic heart disease (*n* = 25), obesity (*n* = 20), benign tumors (*n* = 19), and inflammatory arthritis (*n* = 17). Moreover, circulatory system diseases (disorders of fluid, electrolyte, and acid–base balance, acute myocardial infarction, chronic rheumatic heart disease, non-rheumatic valve disorders, and hypotension) and digestive system diseases (infectious gastroenteritis and colitis, functional intestinal disorders, hemorrhoids and perianal venous thrombosis, diseases of the liver, and diseases of the digestive system) were the primary last layer diseases in the tree diagram (Fig. 3).

### Disease trajectory of MAFLD leading to death

**Fig. 4:**
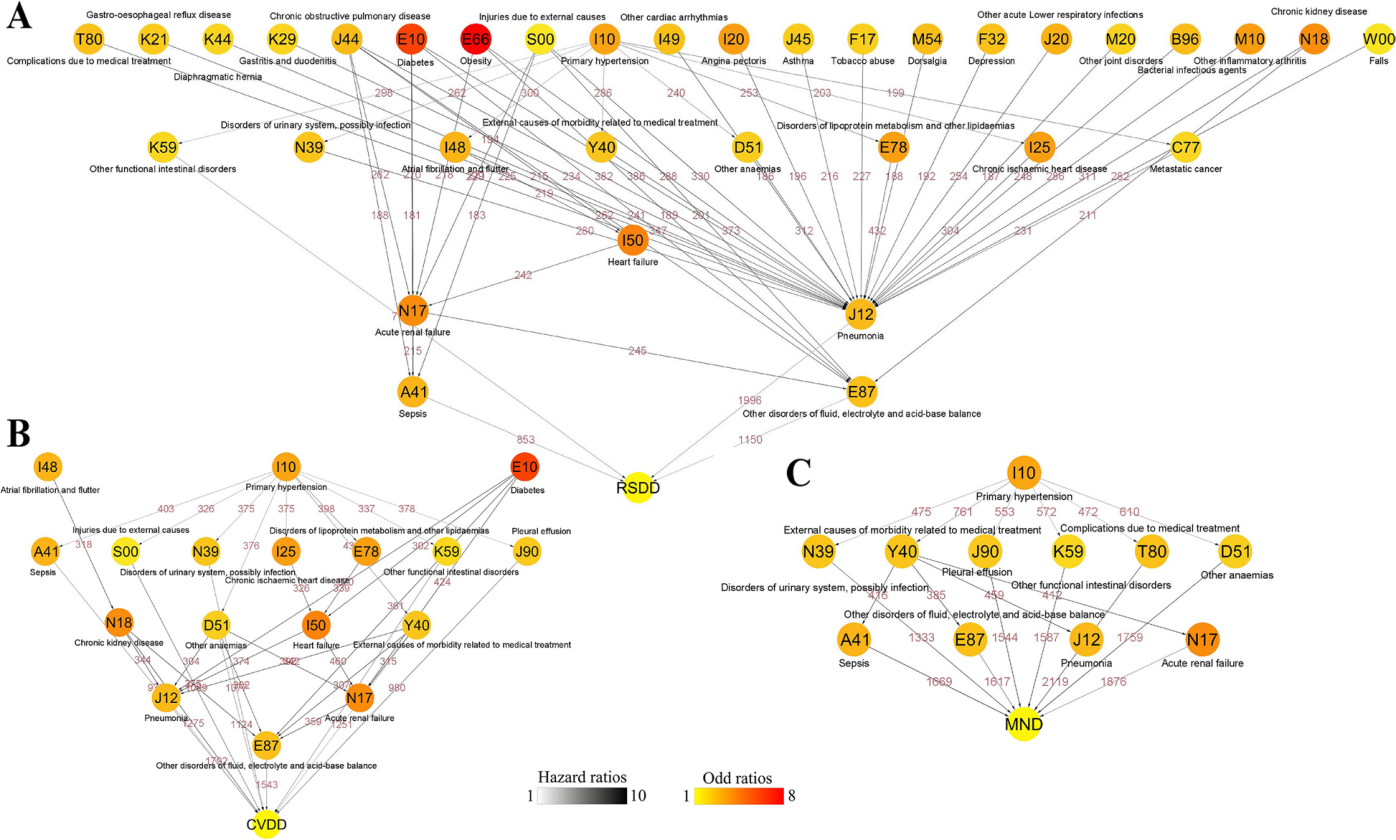
Disease trajectories of MAFLD leading to three main causes of death: **A** malignant neoplasm death, **B** endocrine system disease death, and **C** cardiovascular disease death. A detailed explanation can be found in Fig. 3, and related data can be found in Additional file 4: Table S4-S5

In this study, 14,536 dead individuals and 67,402 matched surviving individuals with MAFLD were included. Baseline characteristics are shown in Additional file 4: Table S3. According to the analysis in Additional file 2: Fig. S2, 102 medical conditions were associated with causes of death (Additional file 4: Table S4), and 374 disease pairs (D1 to D2) were related to causes of death (Additional file 4: Table S5). Malignant neoplasm death, cardiovascular death, and respiratory system death were the main causes of death, and external causes of morbidity related to medical treatment leading to acute renal failure (OR 3.87, 95% CI 3.16–4.74), heart failure leading to acute renal failure (OR 6.97, 95% CI 5.44–8.94), and asthma leading to pneumonia (OR 25.11, 95% CI 12.90–48.89) were the most significant disease pairs for these causes of death (Fig. 4). The disease trajectory of MAFLD leading to other causes of death is shown in Additional file 2: Fig. S6.

### Subgroup and sensitivity analyses

The association of MAFLD with medical conditions and causes of death in different subgroups of sex, alcohol consumption, and body weight was found to be largely significant according to the PheWAS using Cox regression. Females, normal-weight individuals, and nonheavy drinkers had a relatively higher risk of certain diseases and causes of death (Table 2; Additional file 4: Tables S6-9).

In sensitivity analyses, by excluding individuals with pre-existing diagnosis-related system diseases, the relationship between MAFLD and the subsequent 113 disease conditions and eight causes of death was almost consistent (Additional file 4: Tables S10–11). Compared with MAFLD patients without advanced liver fibrosis, those with advanced liver fibrosis had a significantly higher risk of the most part of diseases and causes of death (Additional file 4: Tables S12-13). Moreover, MAFLD patients with risk alleles [MBOAT7 rs641738 (C > T), GCKR rs1260326 (C > T), TM6SF2 rs58542926 (C > T), or PNPLA3 rs738409 (C > G)] are more likely to develop certain medical conditions and causes of death (Additional file 4: Tables S14-21).

### Disease trajectories of genetic susceptibility to MAFLD

Furthermore, the disease and death trajectories of MAFLD by the level of genetic susceptibility were implemented (Additional file 2: Fig. S3–5), and the baseline characteristics are shown in Additional file 4: Tables S22–23. Compared with low genetic susceptibility, individuals with high genetic susceptibility to MAFLD had a significantly higher incidence of MAFLD (70.7% vs 11.7%). Similar to the MAFLD diagnosis, high genetic susceptibility to MAFLD was significantly associated with 108 medical conditions (Additional file 4: Table S24) and six causes of death (Additional file 4: Table S25). They also had largely common disease trajectories with respect to the cardiovascular system, respiratory system, and malignant tumors, while individuals with high genetic susceptibility to MAFLD had significantly specific progression pathways for digestive system death from liver disease (Additional file 4: Tables S26–28 and Additional file 2: Fig. S7–9).

### External verification by Danish Disease Trajectories Browser

Disease trajectories of alcoholic liver disease (ICD-10 K70) and other alcoholic liver disease (ICD-10 K76) using the Danish Disease Trajectories Browser were drawn in Additional file 2: Fig. S10-11. The search filter settings included the following: all 114,099 patients, pair length ≥ 2, relative risk > 1.2, events rate > 1%. A total of 57 medical conditions and 120 pairs were identified for K70 and K76 (Additional file 4: Tables S29-30). K70 induced death mediated by sepsis, bacterial infection, malignant neoplasm, volume depletion, delirium, cardiac arrest, intracerebral haemorrhage, respiratory failure, chronic kidney disease, diabetes mellitus, osteoporosis, disorders of fluid, electrolyte, and acid–base balance, etc. In addition, the downstream diseases of K76 were nearly identical to those of K70, except for cardiac arrest. Together, most of these disease phenotypes were consistent with the disease trajectory of MAFLD, but not cardiovascular disease.

## Discussion

The present study provides a panoramic view of temporal disease and death trajectories of MAFLD in the community-dwelling population of the UK Biobank cohort study. MAFLD was proved to be related to 113 medical conditions and eight causes of death when limiting disease incidence to ≥ 1%. The network of these diseases originates from asthma, diabetes, hypothyroid conditions, and tobacco abuse, with mediation primarily through diverticular diseases of intestine, chronic ischemic heart disease, obesity, benign tumors, and inflammatory arthritis. These pathways predominantly culminated in acute myocardial infarction, disorders of fluid, electrolyte, and acid–base balance, bacterial infectious agents, infectious gastroenteritis and colitis, and functional intestinal disorders. Regarding the death trajectories of MAFLD, the main causes of death included malignant neoplasm, cardiovascular, and respiratory system deaths. The prevalent lethal medical conditions associated with MAFLD were acute renal failure, heart failure, sepsis, pneumonia, and disorders of fluid, electrolyte, and acid–base balance. These maps provide a series of key pathways that link MAFLD to a broad range of health conditions. Considering the huge disease burden caused by MAFLD worldwide, these findings suggest potential key intervention targets for inhibiting the progression of MAFLD-related health events.

In the subgroup analysis, the association of MAFLD with medical conditions and death in different subgroups of sex, alcohol consumption, body weight, and liver fibrosis was largely significant. Although males had a higher prevalence of MAFLD, females had a higher risk of developing subsequent medical conditions. These sex-related differences may be explained by estrogen levels [28]. Overweight is a very common condition in the current era; even without being diagnosed with MAFLD, the proportion of overweight remained as high as 49.9%. Interestingly, this study found that individuals with lean MAFLD (BMI < 25 kg/m^2^) had a higher risk of multi-system diseases than did those who were overweight/obese. However, Liu et al. demonstrated that this effect remained significant only for hepatic outcomes after fully adjusting for confounding factors [5]. Based on our results, individuals with MAFLD who consumed alcohol experienced a protective effect with regard to both intrahepatic and extrahepatic outcomes and mortality. Clinical data have not conclusively confirmed the effects of alcohol consumption on MAFLD outcomes [29–31]. Based on the basic medical principle of “first, do no harm,” it is premature to recommend that MAFLD patients consume moderate alcohol. Similar to findings from previous studies, individuals with MAFLD and fibrosis had a higher risk of cardiac events and nonhepatic mortality [32, 33]. In the sensitivity analysis, the relationship between MAFLD and subsequent disease conditions and causes of death was still significant after excluding individuals with pre-existing diagnosis-related diseases. These results emphasize that MAFLD is a significant health problem in individuals with different characteristics.

Consistent with a previous study’s findings, the pathophysiological mechanism linking MAFLD and cardiovascular disease could be explained by diabetes, obesity, hypertension, and dyslipidemia [34–36]. We found that systemic inflammatory diseases and thrombotic diseases may also be critical to the development of MAFLD in cardiovascular disease. In addition, digestive system diseases, particularly infectious gastroenteritis and colitis, functional intestinal disorders, hemorrhoids, and perianal venous thrombosis, are prominent in patients with MAFLD. Regarding the gut–liver axis, these diseases may be related to enteric dysbacteriosis caused by MAFLD [37]. In this study, MAFLD mediated several common complications, particularly infections including sepsis, mycoses, infective gastroenteritis, pneumonia, lower respiratory infections, skin and subcutaneous infections, and urinary tract infections. According to animal research and ex vivo studies, the association between multi-system infection and MAFLD may be mediated by an immunosuppressive response induced by an altered hepatic metabolic profile [38, 39]. Therefore, further clinical research is required to demonstrate these relationships and provide intervention measures.

A series of medical conditions involved key nodes in MAFLD disease trajectories. Among these, hypertension, diabetes, obesity, and ischemic heart disease are widely considered adverse outcomes [7, 40, 41]. Moreover, asthma, hypothyroid conditions, tobacco abuse, and diverticulosis also dominated the beginning of the disease treegram and carried a large number of diseases downstream (e.g., complications caused by medical treatment; respiratory disease; osteoarthritis; disorders of fluid, electrolyte, and acid–base balance secondary to asthma; and anemia, infection, inflammation, and abdominal hernia secondary to diverticulosis). Furthermore, crosslinking between asthma, tobacco abuse, anxiety, depression, functional intestinal disorders, and sleep disorders suggested that individuals with MAFLD are susceptible to psychosomatic diseases [42, 43]. Our results not only emphasize the association of MAFLD with these medical conditions but also indicate the potential key intervention targets to relieve overall health issues induced by MAFLD.

MAFLD-induced malignant tumors and related deaths have always been a concern. Similar to the findings of Hwang et al., malignant neoplasms were the main cause of death (51.5%) in patients with MAFLD [44]. Our results showed that colon cancer was the cancer most likely to occur among individuals with MAFLD. In addition, disorders of fluid, electrolyte, and acid–base balance, renal failure, and infection were the main causes of death in malignant neoplasms at the end stage. Cardiovascular death was another concern, accounting for 41.4% of deaths in this study. As expected, most individuals died from the failure of multiple organs, such as the heart, kidneys, and respiratory system [45]. Although respiratory system death has attracted less attention in previous research [46], it was the third leading cause of death and accounted for 24.8% of deaths in our study. Several pathways may need to be considered. For example, (1) gastroesophageal reflux disease, tobacco abuse, metastatic cancer, falls, or asthma leading to pneumonia, (2) chronic obstructive pulmonary disease, trauma, heart failure, or renal failure leading to sepsis, and (3) hypertension leading to atrial fibrillation to stroke (supposedly intermediate factor) leading to pneumonia.

Although the results of the subgroup and sensitivity analyses were consistent, the observed disease phenotypes of MAFLD may also be explained by common etiologies, such as comorbidities, environmental factors, and lifestyle. The PRS can be used to assess the genetic risk of individual-specific diseases or disease characteristics and is not affected by other confounding factors [47]. Therefore, we utilized gene mutations strongly associated with MAFLD to investigate whether genetic susceptibility to MAFLD is associated with disease outcomes to reveal the reliability of the disease trajectory of MAFLD. Based on the results, individuals in the highest PRS tertile had 6 times the risk of MAFLD than those in the lowest PRS tertile. Critical pathways identified in the disease and death trajectories of MAFLD also exist in those with high genetic susceptibility to MAFLD, specifically in cardiovascular events, infections, organ failure, cancer, and related deaths that are of particular concern. Notably, genetic susceptibility to MAFLD had a specific pathway from liver disease to death from digestive system diseases. Consistent with previous reports that PRS of MAFLD is strongly associated with cirrhosis and hepatocellular carcinoma [48, 49], these results strongly support the key medical conditions constituting the complex disease networks of MAFLD.

The repeatability of the UK Biobank results was partially verified by the Danish National Patient Register data, but there was heterogeneity in cardiovascular diseases, such as hypertension, coronary heart disease, angina pectoris, myocardial infarction, and heart failure. However, previous studies have validated that cardiovascular diseases and cardiovascular death are important adverse events and outcomes of MAFLD [8]. Therefore, we speculate that this heterogeneity may be caused by omissions in the diagnosis of fatty liver disease using ICD codes in the Danish National Patient Register data.

Unlike the previous definition of NAFLD, which excluded other potential causes of liver diseases such as viral hepatitis or alcohol consumption, the MAFLD definition takes into account the presence of metabolic risk factors, including obesity, diabetes, dyslipidemia, and insulin resistance, in addition to evidence of hepatic steatosis [3]. It enables healthcare providers to diagnose and manage patients based on the underlying metabolic factors driving liver disease, rather than solely focusing on the presence of hepatic steatosis [50]. By broadening the scope of liver diseases associated with metabolic dysfunction, the MAFLD definition allows for a more comprehensive assessment of patients with fatty liver disease, may improve patient risk stratification, allows for a tailored management approach, and provides a better understanding of the underlying pathogenesis of metabolic liver diseases [51]. Our research findings emphasize the view that novel clinical trial designs based on the definition of MAFLD may provide a reference for comprehensive therapeutic interventions and interdisciplinary care aiming to enhance the quality of life for individuals with MAFLD [52].

The major strength of the present study is the large sample community-based cohort that prospectively collected complete data on disease diagnosis and was validated by a large sample from an electronic medical record database. Importantly, the disease trajectory analysis was used to draw a panoramic picture of the time-dependent disease occurrence and development pattern. By using data-driven methods instead of traditional analysis methods, we aimed to overcome the limitation of verifying single disease pairings based on specific hypotheses. Thus, the disease trajectory of MAFLD provides novel information that strengthens our understanding of its pathological effects. Moreover, identifying key nodes in the MAFLD disease network can provide potential intervention targets to mitigate the overall decline in health caused by MAFLD.

We acknowledge some limitations of this study. First, although liver biopsy is the gold standard for diagnosing hepatic steatosis, it is infeasible for large-scale cohort studies; thus, FLI with relatively high sensitivity and specificity is an acceptable alternative [5]. Moreover, a lack of serum insulin data may lead to an erroneous diagnosis of MAFLD in some individuals. Second, the lack of primary healthcare data may have led to the exclusion of less severe diseases, while limiting the incidence rate to > 1% in the analysis process, leaving out rare medical conditions. Therefore, the disease panorama of MAFLD has not yet been completely elucidated. Third, although previous studies have demonstrated that the accuracy of ICD-10 coding for primary diagnoses is relatively high, insufficient accuracy of secondary diagnoses may have affected the research conclusions. In addition, due to inconsistent guidelines or standards referenced by clinical doctors, the same ICD-10 code may originate from different defined diseases. Fourth, although propensity score matching was performed, many confounding factors in the PheWAS of MAFLD were not fully adjusted. To avoid the influence of confounding factors, we conducted a disease trajectory analysis at the level of genetic susceptibility to MAFLD and obtained similar results. To the best of our knowledge, this is the first study to adopt this method. Therefore, the scientific nature of this method needs to be validated. Fifth, the disease tree diagram of MAFLD has been presented according to the referred analysis methods [14–16], the causal relationship between paired diseases has not been fully demonstrated, and some diseases have bidirectional relationships that cannot be fully analyzed. Therefore, in the future, a more rigorous trajectory analysis method should be developed to provide more accurate data, comprehensive disease profiles, and clearer interpretations. Sixth, the response rate of participants in this study was only 5% approximately, the median age of the participants was 59 years (which differs from the progressively decreasing age of onset of MAFLD [53]), and the population included was mainly Caucasian; all of these impact factors may potentially introduce bias and limit the generalizability of the findings. Finally, considering that the disease trajectory analysis approach is purely data-driven, we conducted a test and verified the reliability and reproducibility using the Danish National Patient Register, which is the only available resource to our knowledge. However, these electronic medical record resources do not record complete baseline information of the population; thus, MAFLD cannot be determined. Additionally, the fatty liver disease referred to by ICD-10 codes was used to conduct disease trajectory analysis. Therefore, even if high consistency results are presented, caution still needs to be maintained.

## Conclusions

In conclusion, the disease trajectory analysis of MAFLD identified a range of increased risks for medical conditions and causes of death. Based on the trajectory panorama, we observed that malignant tumors, organ failure, infectious disease, and internal environment disorders were the main end-stage conditions for malignant neoplasm and cardiovascular and respiratory system death, while asthma, diabetes, hypertension, hypothyroid conditions, tobacco abuse, diverticulosis, chronic ischemic heart disease, obesity, benign tumors, and inflammatory arthritis were the primary intermediary medical conditions. Therefore, developing relevant intervention measures that target these key pathways may benefit the MAFLD population by preventing a decline in general health.

### Supplementary Information

**Additional file 1.** Supplementary data.

**Additional file 2: Fig. S1.** Research flowchart for disease trajectory. **Fig. S2.** Research flowchart for death trajectory. **Fig. S3.** Research flow path. **Fig. S4.** Research flowchart disease trajectory of genetic susceptibility to MAFLD. **Fig. S5.** Research flowchart for death trajectory of genetic susceptibility to MAFLD. **Fig. S6.** Tree diagram of disease trajectory for MAFLD to (A) genitourinary system disease death, (B) unnatural cause death, (C) digestive system disease death, and (D) endocrine system disease death. **Fig. S7.** Overview map of the disease trajectories of genetic susceptibility to MAFLD. **Fig. S8.** Disease trajectories of genetic susceptibility to MAFLD leading to (A) malignant neoplasm death, (B) endocrine system disease death, and (C) cardiovascular disease death. **Fig. S9.** Disease trajectory of genetic susceptibility to MAFLD leading to (A) genitourinary system disease death, (B) digestive system disease death, and (C) endocrine system disease death. **Fig. S10.** Disease trajectory of alcoholic liver disease leading to death. **Fig. S11.** Disease trajectory of other alcoholic liver diseases leading to death.

**Additional file 3.** Supplementary methods.

**Additional file 4: Table S1.** PheWAS of MAFLD and 490 medical conditions. **Table S2.** Temporal disease pairs in MAFLD individuals. **Table S3.** Basic characteristics of the dead and surviving participants. **Table S4.** PheWAS of medical conditions and 7 causes of death. **Table S5.** Temporal disease pairs in dead individuals with MAFLD. **Table S6.** PheWAS of MAFLD and 490 disease conditions in heavy drinkers and nonheavy drinkers. **Table S7.** PheWAS of MAFLD and 490 disease conditions in normal weight and overweight/obese individuals. **Table S8.** PheWAS of MAFLD and cause of death in normal weight and overweight/obese individuals. **Table S9.** PheWAS of MAFLD and cause of death in heavy drinkers and nonheavy drinkers. **Table S10.** Sensitivity analysis of the relationship between MAFLD and 490 medical conditions. **Table S11.** Sensitivity analysis of the relationship between MAFLD and cause of death. **Table S12.** PheWAS of nonfibrotic MAFLD and fibrotic MAFLD with 490 medical conditions. **Table S13.** PheWAS of nonfibrotic and fibrotic MAFLD with causes of death.**Table S14.** PheWAS of MAFLD with or without MBOAT7 rs641738 (C > T) and 490 medical conditions. **Table S15.** PheWAS of MAFLD with or without MBOAT7 rs641738 (C > T) and causes of death. **Table S16.** PheWAS of MAFLD with or without GCKR rs1260326 (C > T) and 490 medical conditions. **Table S17.** PheWAS of MAFLD with or without GCKR rs1260326 (C > T) and causes of death. **Table S18.** PheWAS of MAFLD with or without TM6SF2 rs58542926 (C > T) and 490 medical conditions. **Table S19.** PheWAS of MAFLD with or without TM6SF2 rs58542926 (C > T) and causes of death. **Table S20.** PheWAS of MAFLD with or without PNPLA3 rs738409 (C > G) and 490 medical conditions. **Table S21.** PheWAS of MAFLD with or without PNPLA3 rs738409 (C > G) and causes of death. **Table S22.** Basic characteristics of the participants with and without genetic susceptibility to MAFLD. **Table 23.** Basic characteristics of the dead and surviving participants with genetic susceptibility to MAFLD. **Table S24.** PheWAS of genetic susceptibility to MAFLD and 490 medical conditions in males and females. **Table S25.** PheWAS of genetic susceptibility to MAFLD and cause of death in males and females. **Table S26.** Temporal disease pairs in individuals with high genetic susceptibility to MAFLD. **Table S27.** PheWAS of medical conditions and 7 causes of death in individuals with genetic susceptibility to MAFLD. **Table S28.** Temporal disease pairs in dead individuals with genetic susceptibility to MAFLD. **Table S29.** Temporal disease pairs in individuals with alcoholic liver disease. **Table S30.** Temporal disease pairs in individuals with other alcoholic liver diseases.

## Data Availability

Data and materials can be obtained at https://ukbiobank.dnanexus.com/panx/projects. Codes of this study are shared at https://github.com/youyialex/MAFLD.

## Abbreviations

BMI: Body mass index
D: Disease
FLI: Fatty liver index
HR: Hazard ratio
ICD-10: International Classification of Diseases, Tenth Revision
MAFLD: Metabolic dysfunction-associated fatty liver disease
NAFLD: Nonalcoholic fatty liver disease
OR: Odds ratio
PheWAS: Phenome-wide association analysis
PRS: Polygenic risk score
SNP: Single-nucleotide polymorphism
