# Supplementary material for: Multi-system diseases and death trajectory of metabolic dysfunction-associated fatty liver disease: findings from the UK Biobank
Source: BMC Med. 2023 Oct 20;21:398. doi: 10.1186/s12916-023-03080-6 (PMC10590000; doi:10.1186/s12916-023-03080-6)
Supplement: Supplementary file 2 — Additional file 2: Fig. S1. Research flowchart for disease trajectory. Fig. S2. Research flowchart for death trajectory. Fig. S3. Research flow path. Fig. S4. Research flowchart disease trajectory of genetic susceptibility to MAFLD. Fig. S5. Research flowchart for death trajectory of genetic susceptibility to MAFLD. Fig. S6. Tree diagram of disease trajectory for MAFLD to (A) genitourinary system disease death, (B) unnatural cause death, (C) digestive system disease death, and (D) endocrine system disease death. Fig. S7. Overview map of the disease trajectories of genetic susceptibility to MAFLD. Fig. S8. Disease trajectories of genetic susceptibility to MAFLD leading to (A) malignant neoplasm death, (B) endocrine system disease death, and (C) cardiovascular disease death. Fig. S9. Disease trajectory of genetic susceptibility to MAFLD leading to (A) genitourinary system disease death, (B) digestive system disease death, and (C) endocrine system disease death. Fig. S10. Disease trajectory of alcoholic liver disease leading to death. Fig. S11. Disease trajectory of other alcoholic liver diseases leading to death. [file 12916_2023_3080_MOESM2_ESM.pptx]

## Slide 1
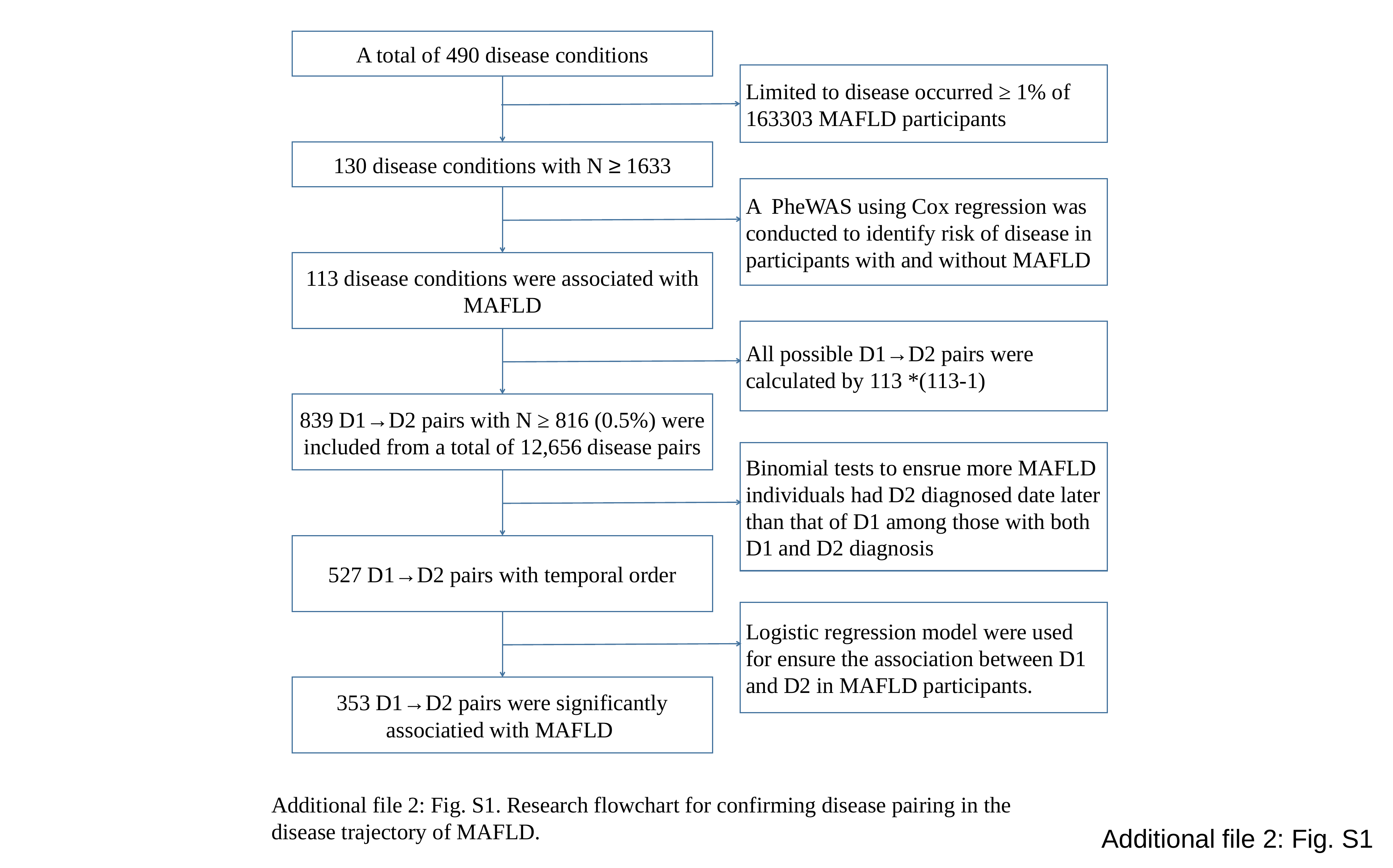

A total of 490 disease conditions
Limited to disease occurred ≥ 1% of 163303 MAFLD participants
130 disease conditions with N ≥ 1633
A PheWAS using Cox regression was conducted to identify risk of disease in participants with and without MAFLD
113 disease conditions were associated with MAFLD
All possible D1→D2 pairs were calculated by 113 *(113-1)
839 D1→D2 pairs with N ≥ 816 (0.5%) were included from a total of 12,656 disease pairs
Binomial tests to ensrue more MAFLD individuals had D2 diagnosed date later than that of D1 among those with both D1 and D2 diagnosis
527 D1→D2 pairs with temporal order
Logistic regression model were used for ensure the association between D1 and D2 in MAFLD participants.
353 D1→D2 pairs were significantly associatied with MAFLD
Additional file 2: Fig. S1. Research flowchart for confirming disease pairing in the disease trajectory of MAFLD.
Additional file 2: Fig. S1

## Slide 2
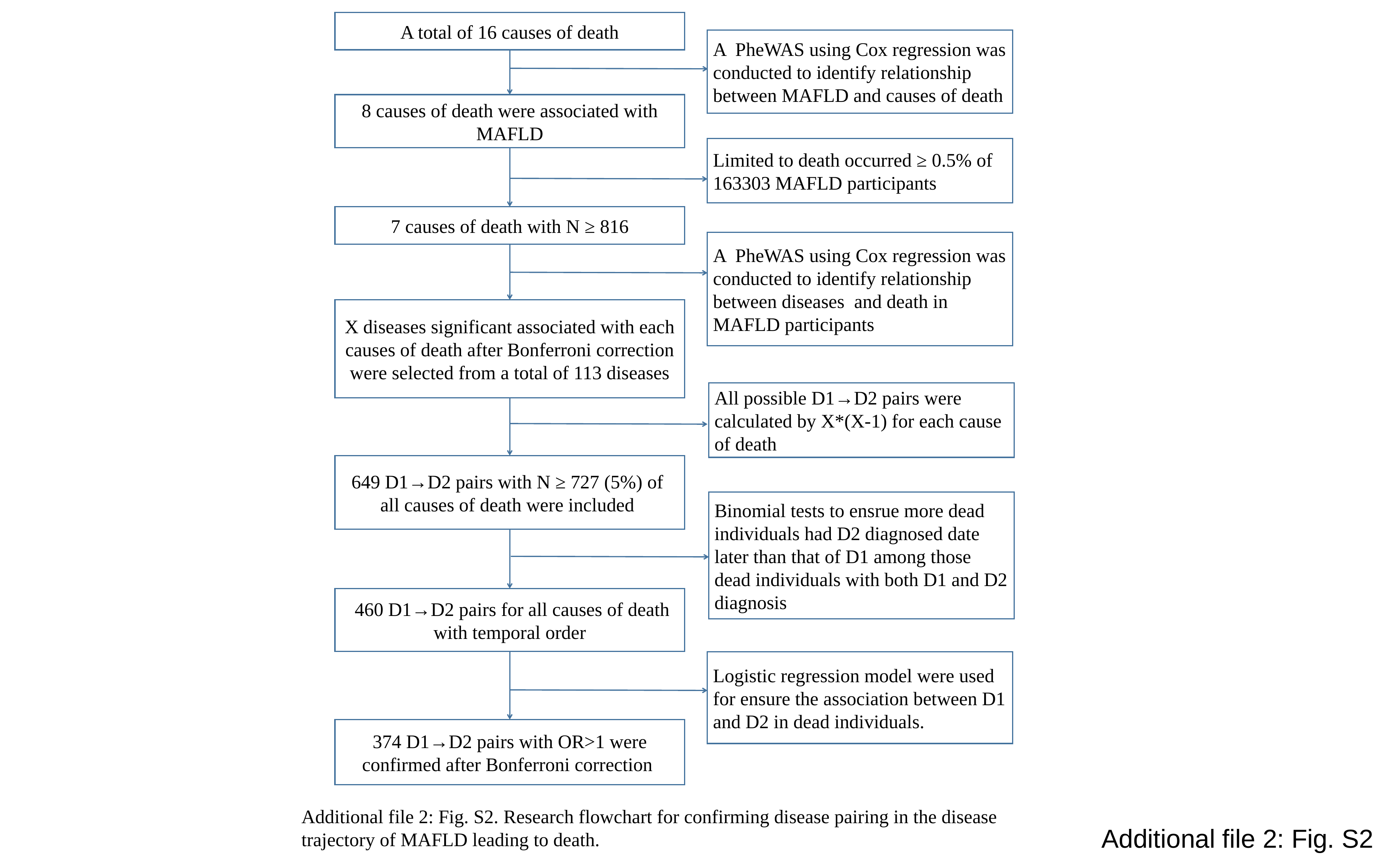

A total of 16 causes of death
A PheWAS using Cox regression was conducted to identify relationship between MAFLD and causes of death
8 causes of death were associated with MAFLD
Limited to death occurred ≥ 0.5% of 163303 MAFLD participants
7 causes of death with N ≥ 816
A PheWAS using Cox regression was conducted to identify relationship between diseases and death in MAFLD participants
X diseases significant associated with each causes of death after Bonferroni correction were selected from a total of 113 diseases
All possible D1→D2 pairs were calculated by X*(X-1) for each cause of death
649 D1→D2 pairs with N ≥ 727 (5%) of
all causes of death were included
Binomial tests to ensrue more dead individuals had D2 diagnosed date later than that of D1 among those dead individuals with both D1 and D2 diagnosis
 460 D1→D2 pairs for all causes of death with temporal order
Logistic regression model were used for ensure the association between D1 and D2 in dead individuals.
374 D1→D2 pairs with OR>1 were confirmed after Bonferroni correction
Additional file 2: Fig. S2. Research flowchart for confirming disease pairing in the disease trajectory of MAFLD leading to death.
Additional file 2: Fig. S2

## Slide 3
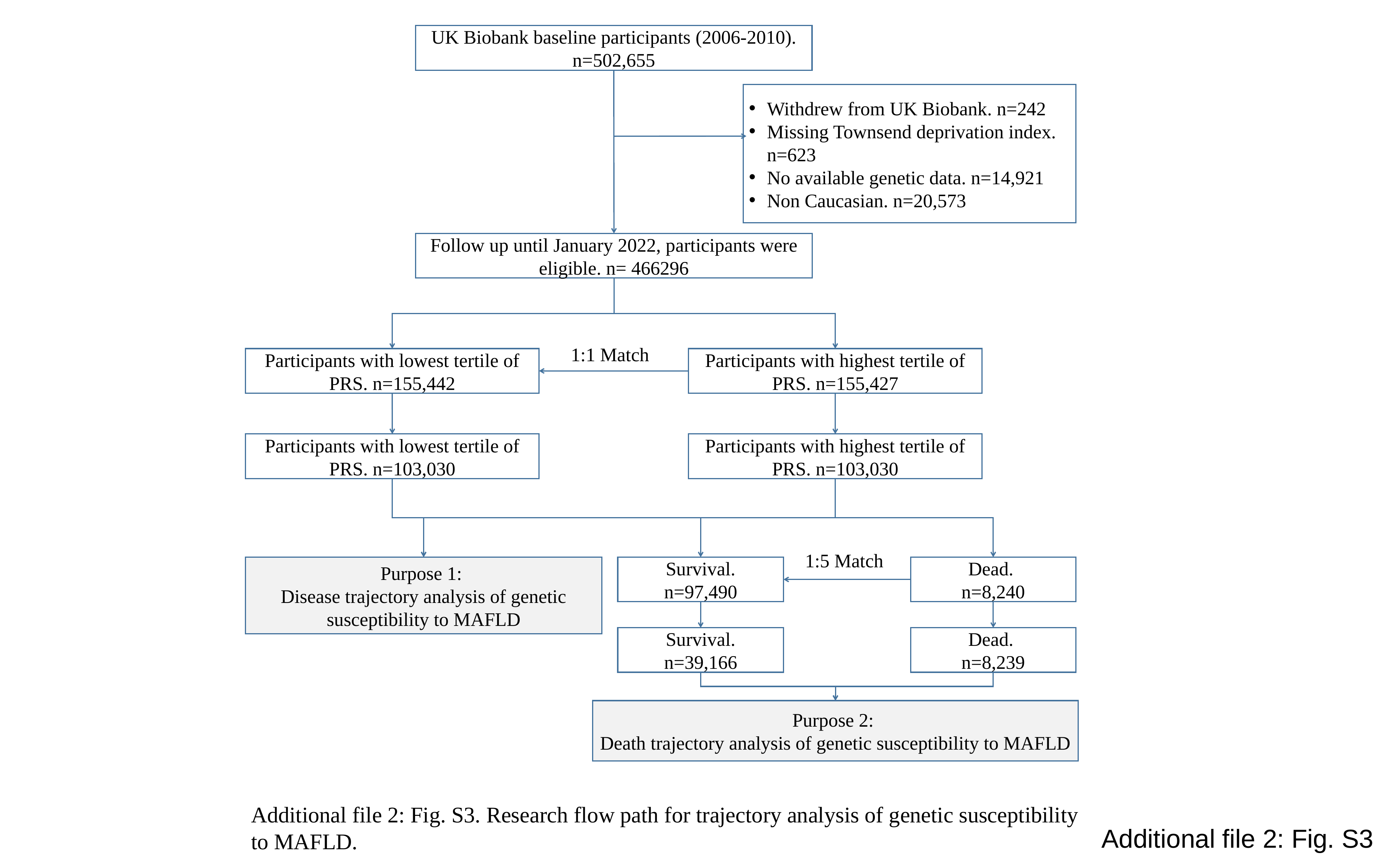

UK Biobank baseline participants (2006-2010). n=502,655
Withdrew from UK Biobank. n=242
Missing Townsend deprivation index. n=623
No available genetic data. n=14,921
Non Caucasian. n=20,573
Follow up until January 2022, participants were eligible. n= 466296
1:1 Match
Participants with lowest tertile of PRS. n=155,442
Participants with highest tertile of PRS. n=155,427
Participants with lowest tertile of PRS. n=103,030
Participants with highest tertile of PRS. n=103,030
1:5 Match
Purpose 1:
Disease trajectory analysis of genetic susceptibility to MAFLD
Survival.
n=97,490
Dead.
n=8,240
Survival.
n=39,166
Dead.
n=8,239
Purpose 2:
Death trajectory analysis of genetic susceptibility to MAFLD
Additional file 2: Fig. S3. Research flow path for trajectory analysis of genetic susceptibility to MAFLD.
Additional file 2: Fig. S3

## Slide 4
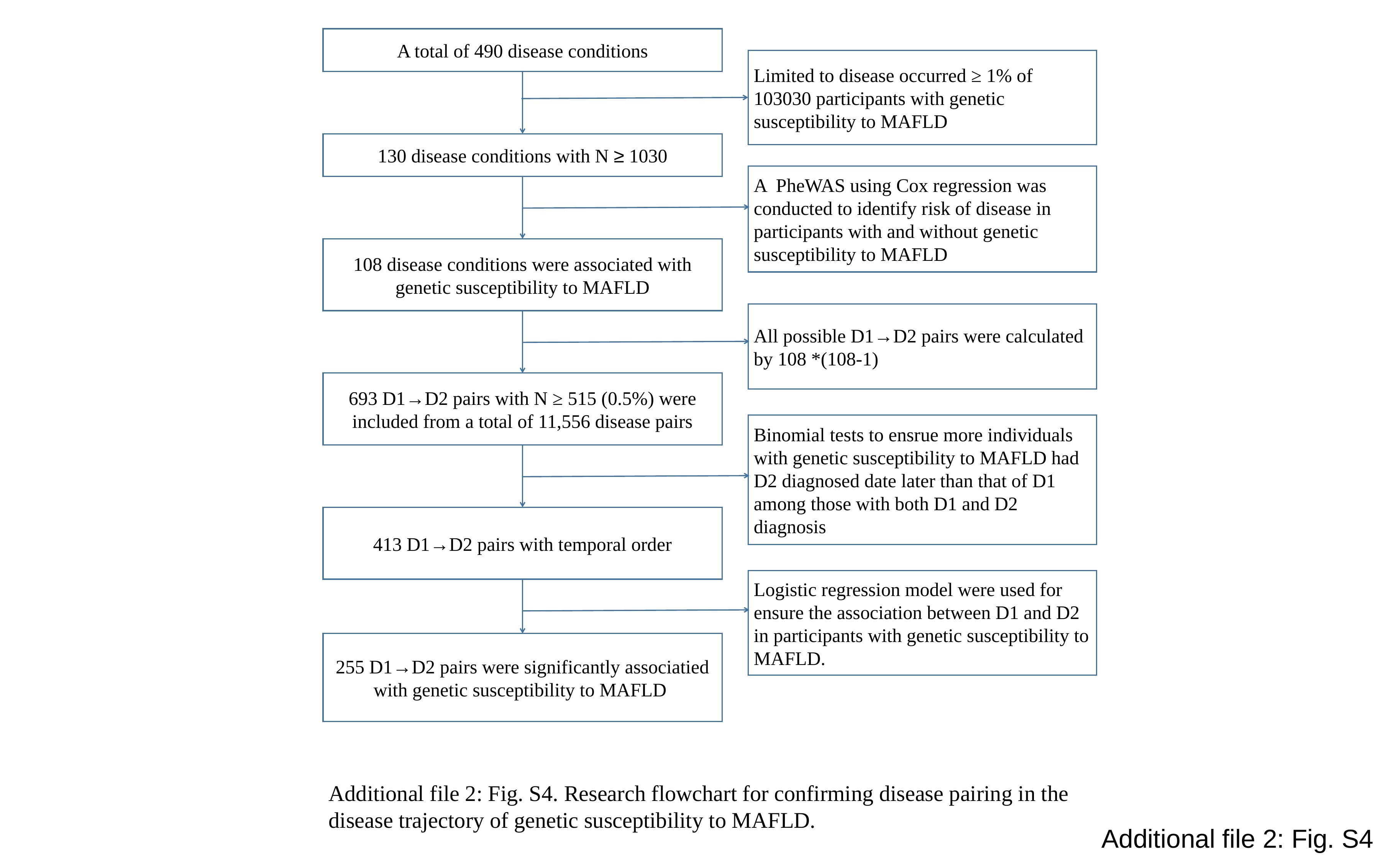

A total of 490 disease conditions
Limited to disease occurred ≥ 1% of 103030 participants with genetic susceptibility to MAFLD
130 disease conditions with N ≥ 1030
A PheWAS using Cox regression was conducted to identify risk of disease in participants with and without genetic susceptibility to MAFLD
108 disease conditions were associated with genetic susceptibility to MAFLD
All possible D1→D2 pairs were calculated by 108 *(108-1)
693 D1→D2 pairs with N ≥ 515 (0.5%) were included from a total of 11,556 disease pairs
Binomial tests to ensrue more individuals with genetic susceptibility to MAFLD had D2 diagnosed date later than that of D1 among those with both D1 and D2 diagnosis
413 D1→D2 pairs with temporal order
Logistic regression model were used for ensure the association between D1 and D2 in participants with genetic susceptibility to MAFLD.
255 D1→D2 pairs were significantly associatied with genetic susceptibility to MAFLD
Additional file 2: Fig. S4. Research flowchart for confirming disease pairing in the disease trajectory of genetic susceptibility to MAFLD.
Additional file 2: Fig. S4

## Slide 5
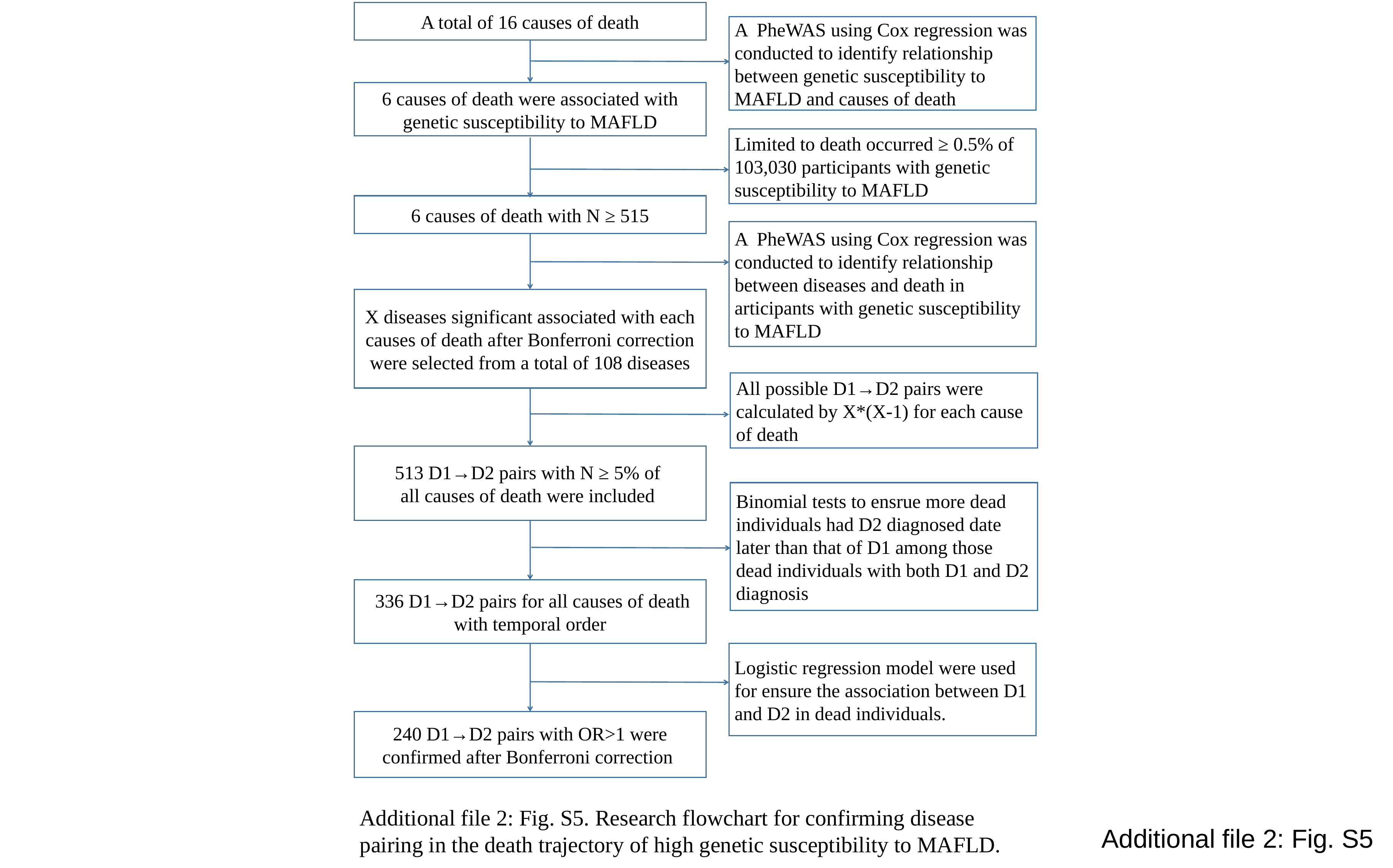

A total of 16 causes of death
A PheWAS using Cox regression was conducted to identify relationship between genetic susceptibility to MAFLD and causes of death
6 causes of death were associated with genetic susceptibility to MAFLD
Limited to death occurred ≥ 0.5% of 103,030 participants with genetic susceptibility to MAFLD
6 causes of death with N ≥ 515
A PheWAS using Cox regression was conducted to identify relationship between diseases and death in articipants with genetic susceptibility to MAFLD
X diseases significant associated with each causes of death after Bonferroni correction were selected from a total of 108 diseases
All possible D1→D2 pairs were calculated by X*(X-1) for each cause of death
513 D1→D2 pairs with N ≥ 5% of
all causes of death were included
Binomial tests to ensrue more dead individuals had D2 diagnosed date later than that of D1 among those dead individuals with both D1 and D2 diagnosis
 336 D1→D2 pairs for all causes of death with temporal order
Logistic regression model were used for ensure the association between D1 and D2 in dead individuals.
240 D1→D2 pairs with OR>1 were confirmed after Bonferroni correction
Additional file 2: Fig. S5. Research flowchart for confirming disease pairing in the death trajectory of high genetic susceptibility to MAFLD.
Additional file 2: Fig. S5

## Slide 6
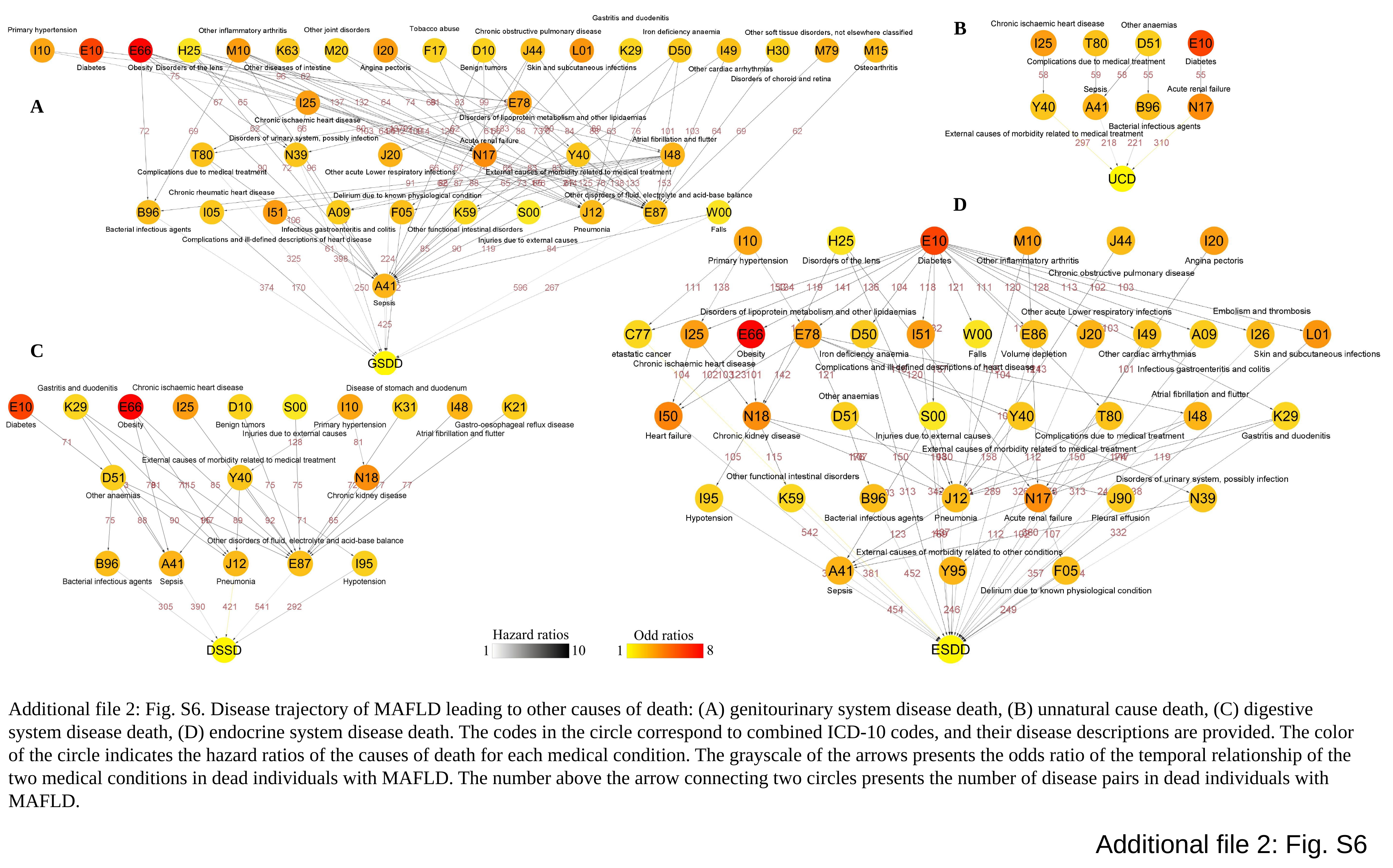

B
A
D
C
Additional file 2: Fig. S6. Disease trajectory of MAFLD leading to other causes of death: (A) genitourinary system disease death, (B) unnatural cause death, (C) digestive system disease death, (D) endocrine system disease death. The codes in the circle correspond to combined ICD-10 codes, and their disease descriptions are provided. The color of the circle indicates the hazard ratios of the causes of death for each medical condition. The grayscale of the arrows presents the odds ratio of the temporal relationship of the two medical conditions in dead individuals with MAFLD. The number above the arrow connecting two circles presents the number of disease pairs in dead individuals with MAFLD.
Additional file 2: Fig. S6

## Slide 7
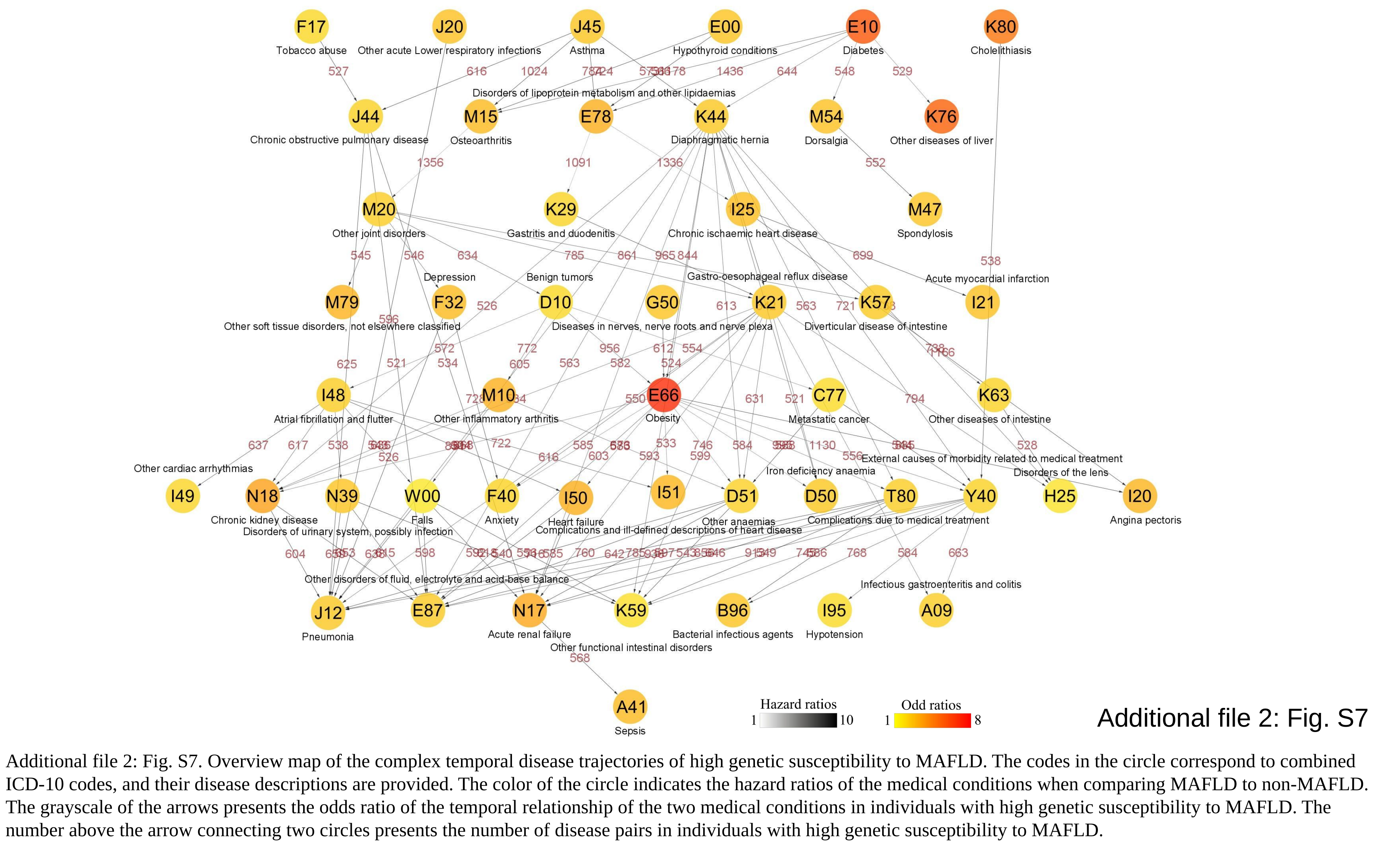

Additional file 2: Fig. S7
Additional file 2: Fig. S7. Overview map of the complex temporal disease trajectories of high genetic susceptibility to MAFLD. The codes in the circle correspond to combined ICD-10 codes, and their disease descriptions are provided. The color of the circle indicates the hazard ratios of the medical conditions when comparing MAFLD to non-MAFLD. The grayscale of the arrows presents the odds ratio of the temporal relationship of the two medical conditions in individuals with high genetic susceptibility to MAFLD. The number above the arrow connecting two circles presents the number of disease pairs in individuals with high genetic susceptibility to MAFLD.

## Slide 8
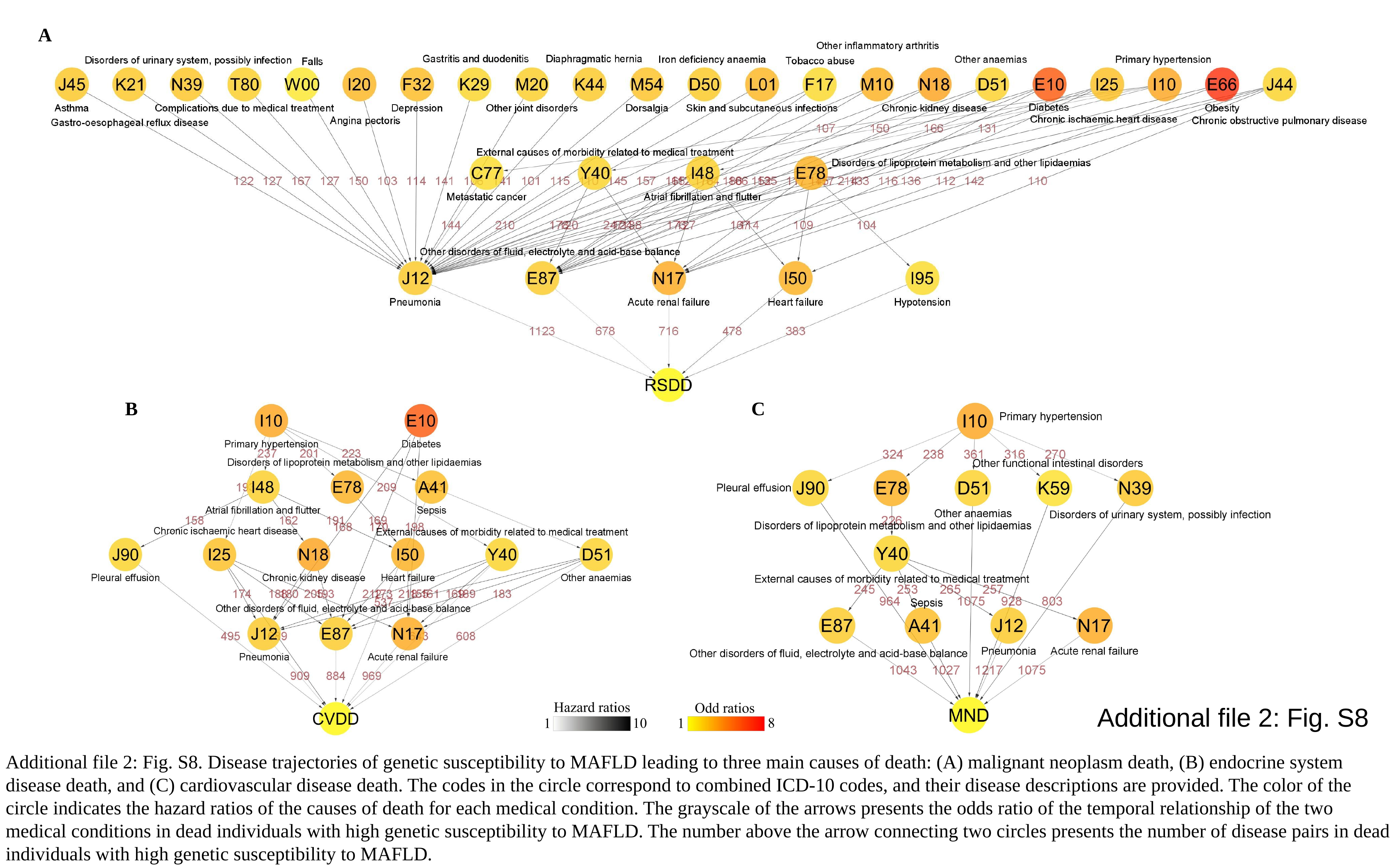

A
B
C
Additional file 2: Fig. S8
Additional file 2: Fig. S8. Disease trajectories of genetic susceptibility to MAFLD leading to three main causes of death: (A) malignant neoplasm death, (B) endocrine system disease death, and (C) cardiovascular disease death. The codes in the circle correspond to combined ICD-10 codes, and their disease descriptions are provided. The color of the circle indicates the hazard ratios of the causes of death for each medical condition. The grayscale of the arrows presents the odds ratio of the temporal relationship of the two medical conditions in dead individuals with high genetic susceptibility to MAFLD. The number above the arrow connecting two circles presents the number of disease pairs in dead individuals with high genetic susceptibility to MAFLD.

## Slide 9
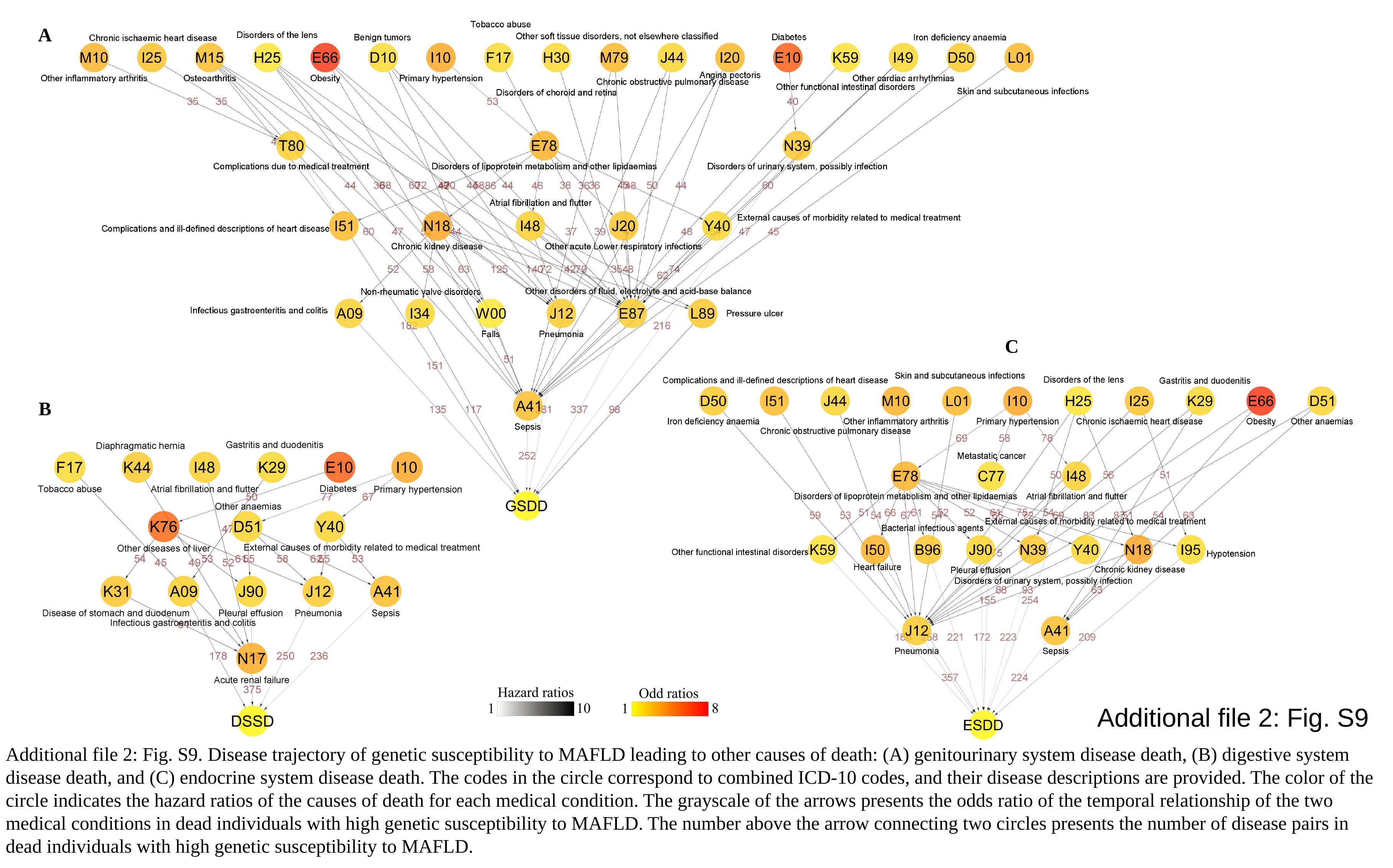

A
C
B
Additional file 2: Fig. S9
Additional file 2: Fig. S9. Disease trajectory of genetic susceptibility to MAFLD leading to other causes of death: (A) genitourinary system disease death, (B) digestive system disease death, and (C) endocrine system disease death. The codes in the circle correspond to combined ICD-10 codes, and their disease descriptions are provided. The color of the circle indicates the hazard ratios of the causes of death for each medical condition. The grayscale of the arrows presents the odds ratio of the temporal relationship of the two medical conditions in dead individuals with high genetic susceptibility to MAFLD. The number above the arrow connecting two circles presents the number of disease pairs in dead individuals with high genetic susceptibility to MAFLD.

## Slide 10
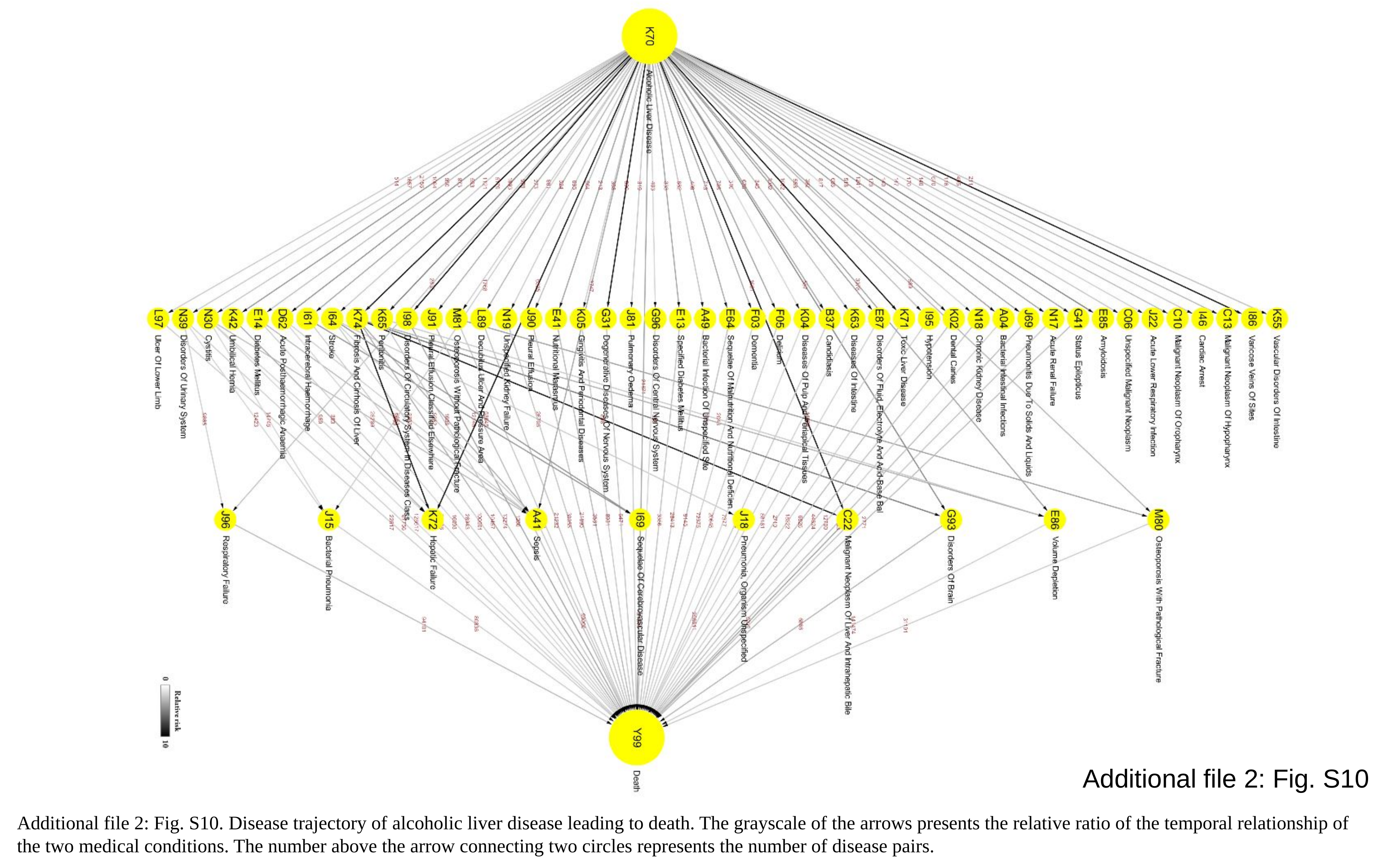

Additional file 2: Fig. S10
Additional file 2: Fig. S10. Disease trajectory of alcoholic liver disease leading to death. The grayscale of the arrows presents the relative ratio of the temporal relationship of the two medical conditions. The number above the arrow connecting two circles represents the number of disease pairs.

## Slide 11
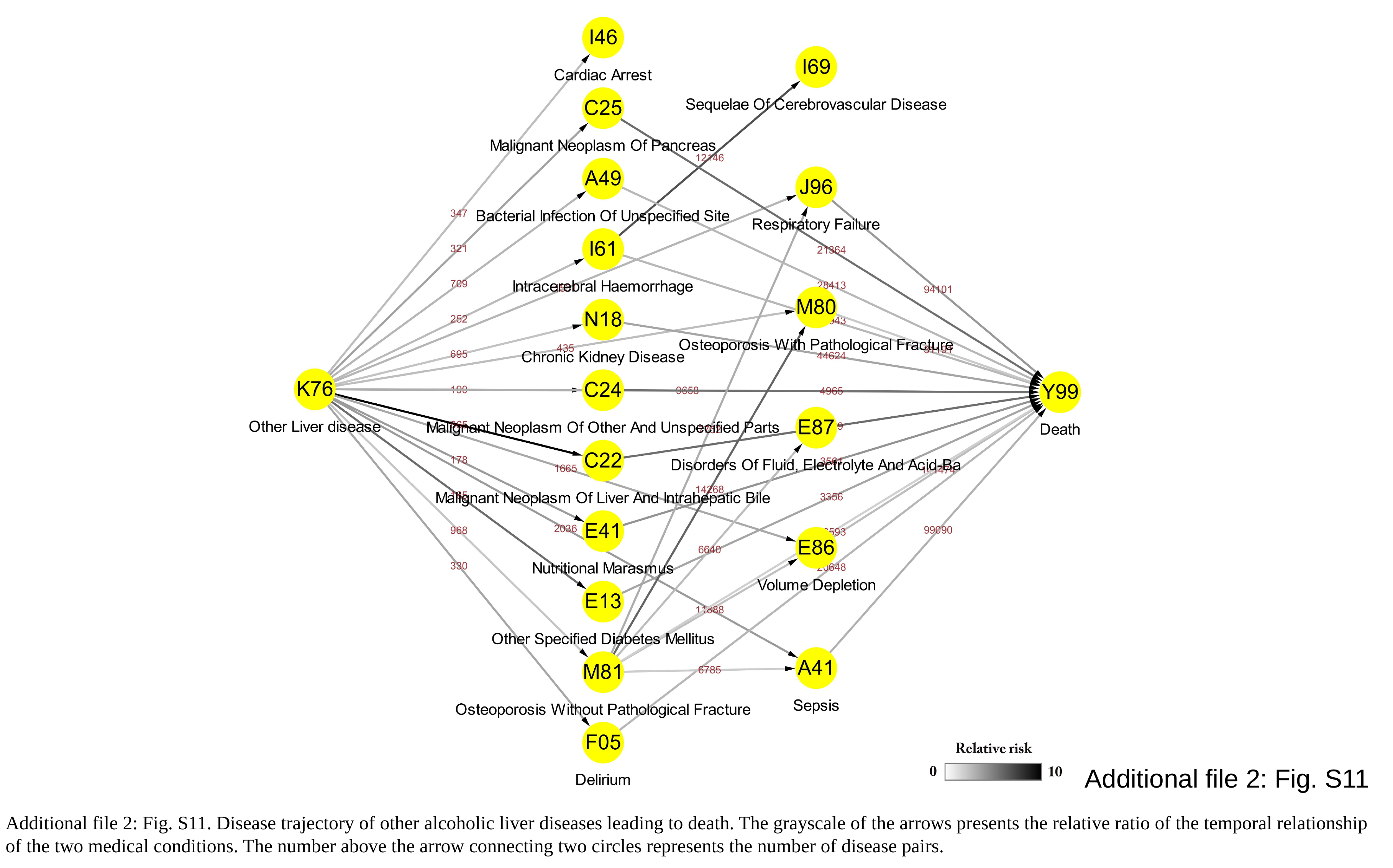

Additional file 2: Fig. S11
Additional file 2: Fig. S11. Disease trajectory of other alcoholic liver diseases leading to death. The grayscale of the arrows presents the relative ratio of the temporal relationship of the two medical conditions. The number above the arrow connecting two circles represents the number of disease pairs.
