# Supplementary material for: Multi-system diseases and death trajectory of metabolic dysfunction-associated fatty liver disease: findings from the UK Biobank
Source: BMC Med. 2023 Oct 20;21:398. doi: 10.1186/s12916-023-03080-6 (PMC10590000; doi:10.1186/s12916-023-03080-6)
Supplement: Supplementary file 3 — Additional file 3. Supplementary methods. [file 12916_2023_3080_MOESM3_ESM.docx]

**Disease trajectory analysis**

**Section 1: Temporal** **disease trajectories of MAFLD**

**Step 1:** **identifying independent associations between MAFLD and medical conditions by PheWAS**

A phenome-wide association study (PheWAS) using Cox regression was conducted to identify the disease risk of participants with MAFLD compared to age-, sex-, and Townsend deprivation index-matched controls. A total of 490 medical conditions (classifying by combined ICD-10 codes, which are shown in Supplementary Data 1) were tested in the Cox regression models.

To analyze each outcome disease in the Cox regression model, a subcohort was formed by excluding participants with a history of the outcome diseases at baseline according to self-reported medical history and ICD-10 coding. Follow-up period of the individuals in the subcohort calculated by date of death, date of the outcome disease diagnosis at first time, or date of study end (2022/2/2) minus date of study object enrollment.

To meet adequate statistical power, the analysis was limited to disease occurring in > 1% of MAFLD participants (n=1633). According to the results of Cox regression, diseases with a hazard ratio (HR) > 1 and a p < 0.05/n (the Bonferroni corrected threshold; n: number of disease categories) were retained for second-step analysis.


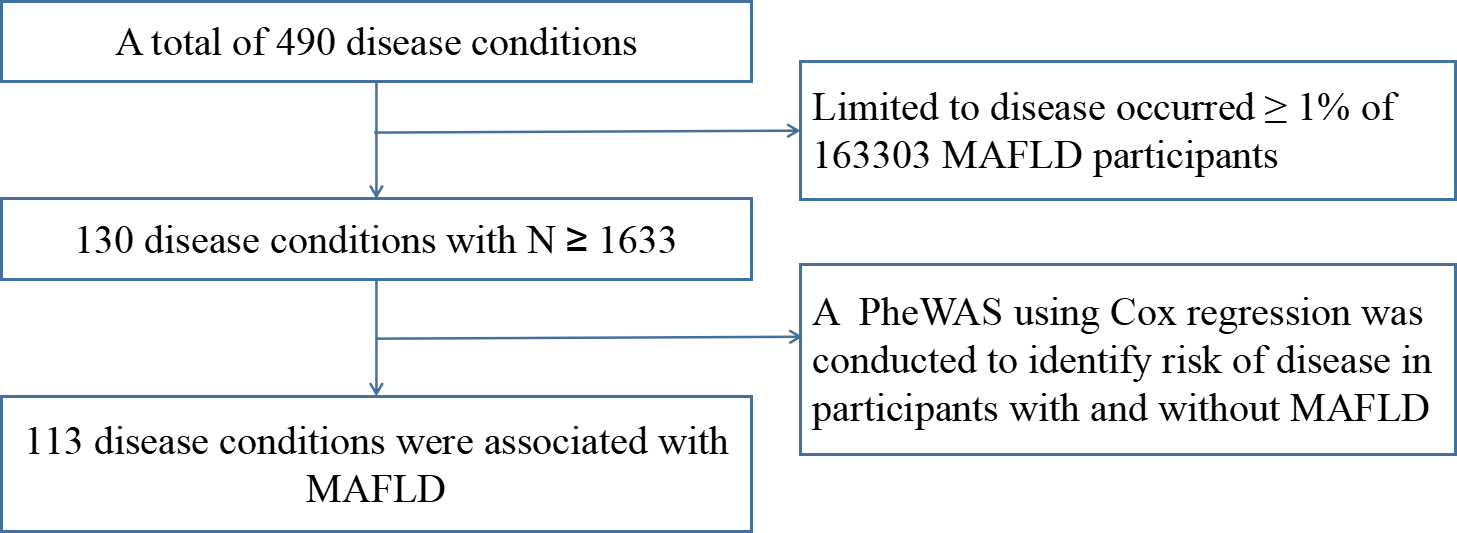


**Figure 1. Association between MAFLD and 490 medical conditions by PheWAS**

**Step 2: Identifying disease pairs with temporal order**

Assuming that all 117 diseases may be interrelated, all possible diseases 1 (D1) and 2 (D2) were paired, and the number of D1-D2 pairs was calculated as 113*(113-1). To meet adequate statistical power, pairs occurring in > 0.5% of MAFLD participants (n=816) were included. Then, to ensure the temporal order logic, we tested whether more MAFLD individuals (>50%) had a D2 diagnosis date later than that of D1 among those with both D1 and D2 diagnoses by Binomial tests, and disease pairs with P < the Bonferroni corrected threshold remained. This step can eliminate the possibility of reciprocal causation between D1 and D2.


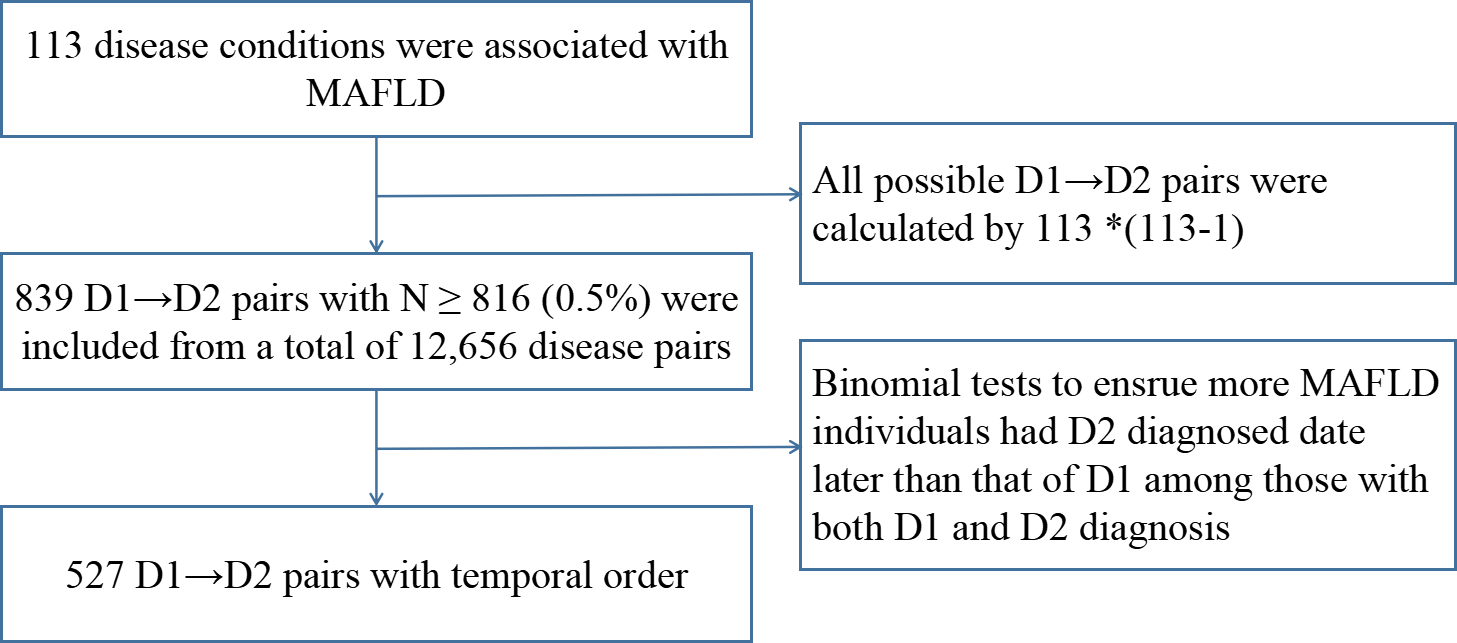


**Figure 2. Disease pairs with temporal order were selected by Binomial tests**

**Step 3: Disease pairs were confirmed by Logistic regression**

For each disease pair, a nested case‒control dataset using nearest neighbor sampling was established in participants with MAFLD. In each case‒control dataset, at most 5 controls were matched to each case based on age, sex, and Townsend deprivation index at baseline. Each case‒control dataset was formed by excluding participants with a history of D1 and D2 at the baseline date according to self-reported medical history and ICD-10 coding.

Finally, D1 was set as the exposure, D2 was set as the outcome, and a logistic regression model was used to investigate the association between D1 and D2. Disease pairs with a significantly increased risk of D2 after D1 (odds ratio (OR) > 1; P < the Bonferroni corrected threshold) were confirmed.


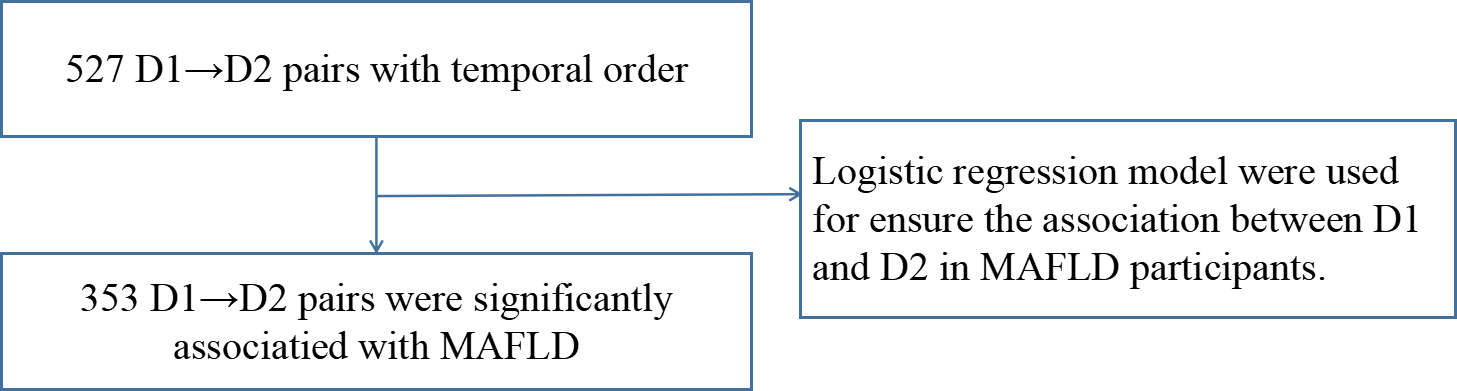


**Figure 3. Disease pairs with a significantly increased risk of D2 after D1 were confirmed**

**Section 2: Temporal disease trajectories of MAFLD leading to death**

**STEP 1: Identify the association between MAFLD and cause of death by PheWAS**

First, propensity score matching (1:5) was conducted according to age, sex, and Townsend deprivation index for dead and surviving subjects. Second, a Cox regression model was used to analyze the association between MAFLD and the cause of death by PheWAS. The follow-up period of the individuals was calculated by the date of death or the date of study end (2022/2/2) minus the date of study object enrollment.

According to the results of Cox regression, a total of 9 causes of death were found to be significantly correlated with MAFLD (HR > 1 and p < 0.05/n (the Bonferroni corrected threshold; n: number of death categories)). These causes of death were chosen for subsequent analysis.


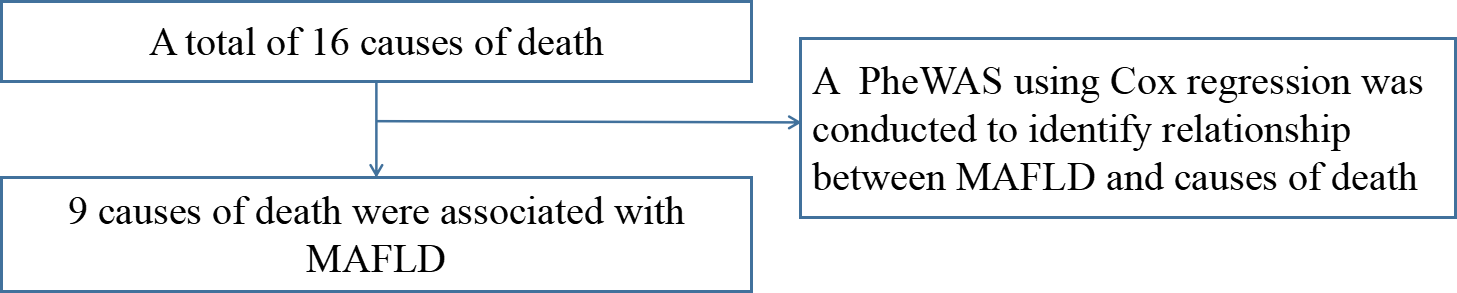


**Figure 4. Association between MAFLD and cause of death by PheWAS**

According to the results of Cox regression, diseases with a hazard ratio (HR) > 1 and a p < 0.05/n (the Bonferroni corrected threshold; n: number of disease categories) were retained for next step analysis.

**Step 2**: **Identifying the association between medical conditions and each cause of death by PheWAS**

To meet adequate statistical power, the analysis was limited to any cause of death that occurred in > 0.5% of MAFLD participants (n=816), and 7 causes of death were analyzed separately. PheWAS using Cox regression was conducted to identify the relationship between medical conditions and each cause of death in participants with MAFLD. A total of 113 medical conditions selected from Section 1 were tested in the Cox regression models.

To analyze each outcome disease in the Cox regression model, a subcohort was formed by excluding participants with a history of the outcome disease at baseline according to self-reported medical history and ICD-10 coding. The follow-up period of the individuals in the subcohort was calculated by date of death or date of study end (2022/2/2) minus date of the disease diagnosis at first time.

For each cause of death, X diseases were found to be significantly correlated with it and were chosen for subsequent analysis. To meet adequate statistical power, diseases with a hazard ratio (HR) > 1 and a p < 0.05/n (the Bonferroni corrected threshold; n: number of disease categories) were retained for next step analysis.


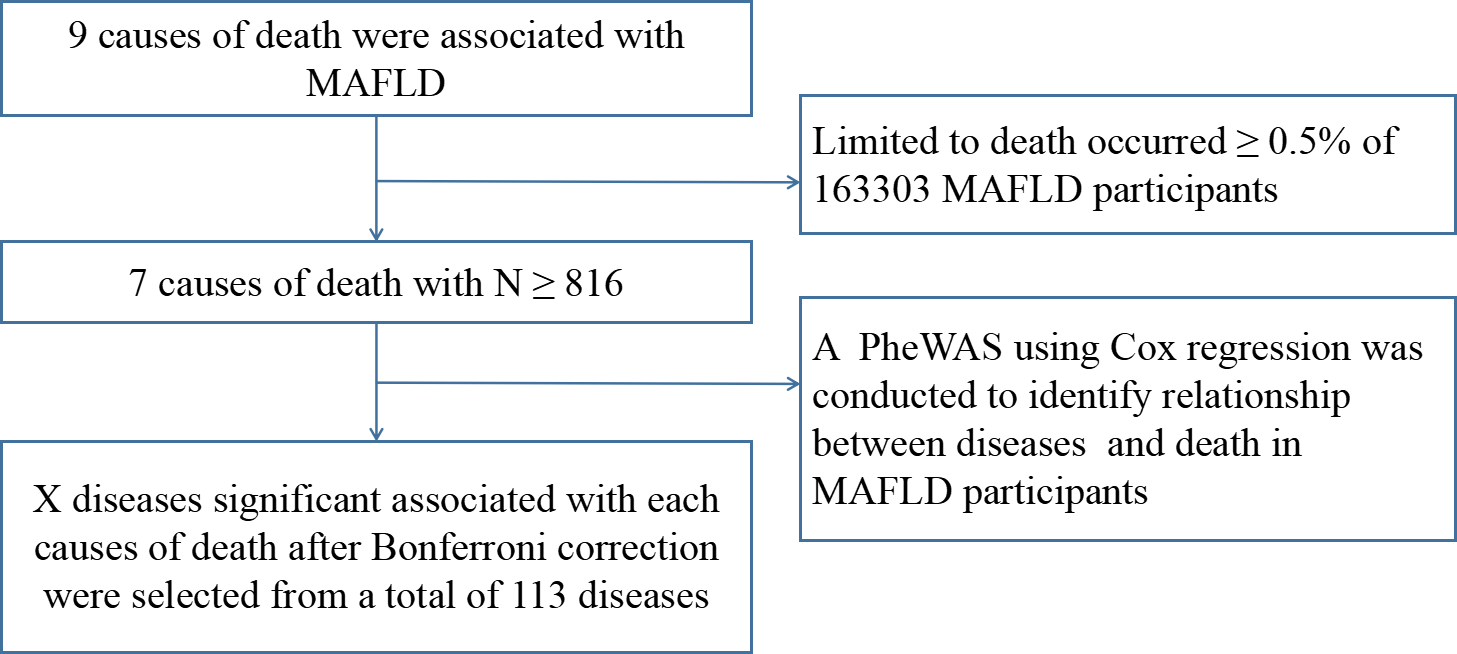


**Figure 5. Association between medical conditions and 7 causes of death by PheWAS**

**Step 3: Identifying disease pairs with temporal order leading to death**

Each cause of death was analyzed separately. For example, assume that 50 (out of 113) diseases are related to cardiovascular death. Then, all possible disease 1 (D1) and disease 2 (D2) were paired, and the number of D1-D2 pairs was calculated by 50*(50-1). To meet adequate statistical power, pairs that occurred in > 5% (the matching ratio of survival and death was 1:5) of the death participants (n=727) were included.

Then, to ensure the temporal order logic, we tested whether more dead individuals (>50%) had a D2 diagnosis date later than that of D1 among those with both D1 and D2 diagnoses by Binomial tests, and disease pairs with P < the Bonferroni corrected threshold were retained. This step can eliminate the possibility of reciprocal causation between D1 and D2. A similar analysis was conducted for the other 6 causes of death.


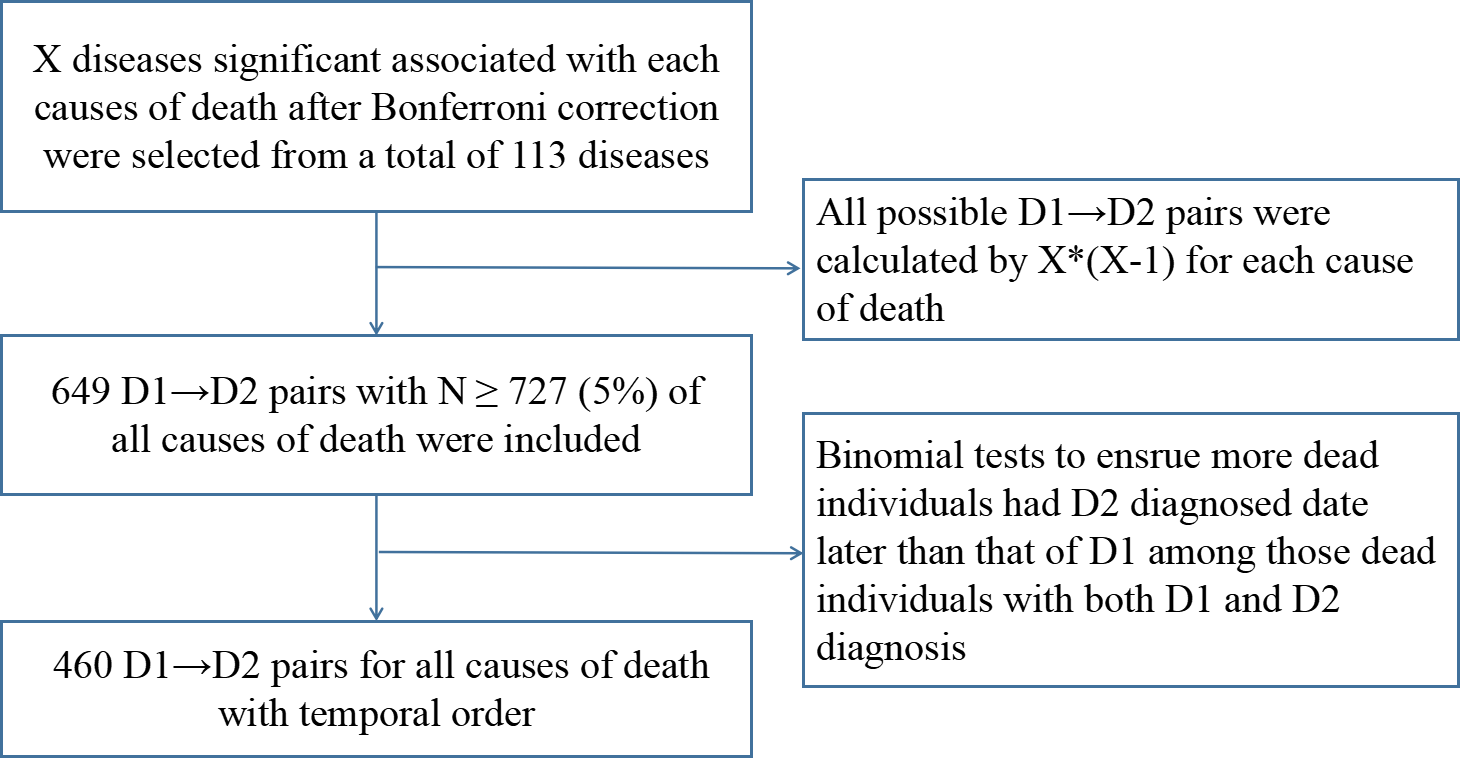


**Figure 6. Disease pairs with temporal order were selected by Binomial tests**

**Step 4: Disease pairs were confirmed by Logistic regression**

Each cause of death was analyzed separately. For each disease pair, a nested case‒control dataset using nearest neighbor sampling was established in dead participants. In each case‒control dataset, at most 5 controls were matched to each case based on age, sex, and Townsend deprivation index at baseline. Each case‒control dataset was formed by excluding participants with a history of D1 and D2 at the baseline date according to self-reported medical history and ICD-10 coding.

Finally, D1 was set as the exposure, D2 was set as the outcome, and a Logistic regression model was used to investigate the association between D1 and D2. Disease pairs with a significantly increased risk of D2 after D1 (odds ratio (OR) > 1; P < the Bonferroni corrected threshold) were confirmed.


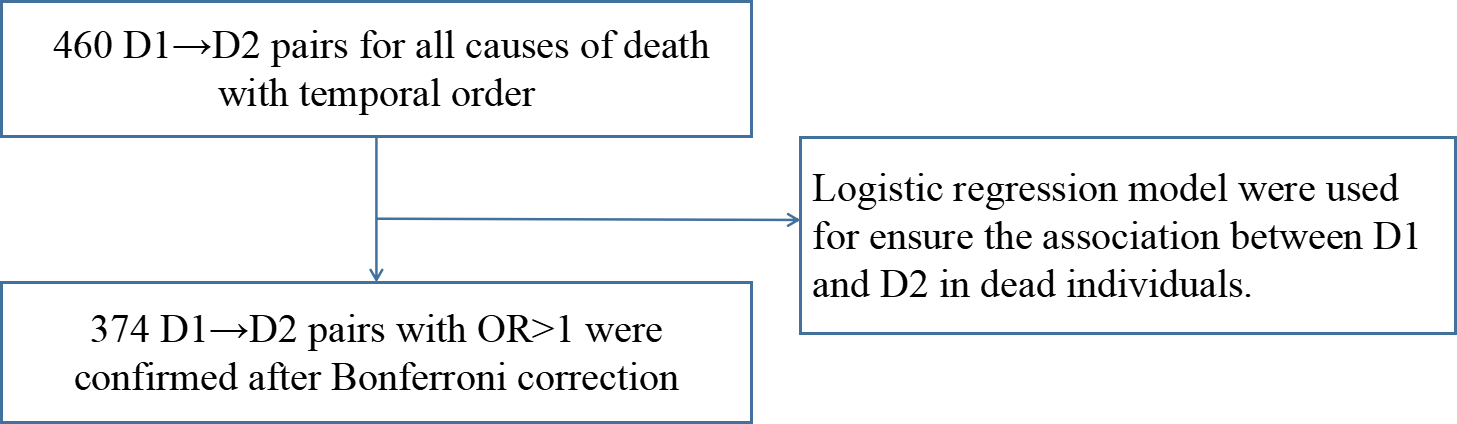


**Figure 7. Disease pairs leading to death were confirmed**

**The code is available at https://github.com/youyialex/MAFLD**
