# Supplementary material for: Multi-system diseases and death trajectory of metabolic dysfunction-associated fatty liver disease: findings from the UK Biobank
Source: BMC Med. 2023 Oct 20;21:398. doi: 10.1186/s12916-023-03080-6 (PMC10590000; doi:10.1186/s12916-023-03080-6)
Supplement: Supplementary file 4 — Additional file 4: Table S1. PheWAS of MAFLD and 490 medical conditions. Table S2. Temporal disease pairs in MAFLD individuals. Table S3. Basic characteristics of the dead and surviving participants. Table S4. PheWAS of medical conditions and 7 causes of death. Table S5. Temporal disease pairs in dead individuals with MAFLD. Table S6. PheWAS of MAFLD and 490 disease conditions in heavy drinkers and nonheavy drinkers. Table S7. PheWAS of MAFLD and 490 disease conditions in normal weight and overweight/obese individuals. Table S8. PheWAS of MAFLD and cause of death in normal weight and overweight/obese individuals. Table S9. PheWAS of MAFLD and cause of death in heavy drinkers and nonheavy drinkers. Table S10. Sensitivity analysis of the relationship between MAFLD and 490 medical conditions. Table S11. Sensitivity analysis of the relationship between MAFLD and cause of death. Table S12. PheWAS of nonfibrotic MAFLD and fibrotic MAFLD with 490 medical conditions. Table S13. PheWAS of nonfibrotic and fibrotic MAFLD with causes of death.Table S14. PheWAS of MAFLD with or without MBOAT7 rs641738 (C > T) and 490 medical conditions. Table S15. PheWAS of MAFLD with or without MBOAT7 rs641738 (C > T) and causes of death. Table S16. PheWAS of MAFLD with or without GCKR rs1260326 (C > T) and 490 medical conditions. Table S17. PheWAS of MAFLD with or without GCKR rs1260326 (C > T) and causes of death. Table S18. PheWAS of MAFLD with or without TM6SF2 rs58542926 (C > T) and 490 medical conditions. Table S19. PheWAS of MAFLD with or without TM6SF2 rs58542926 (C > T) and causes of death. Table S20. PheWAS of MAFLD with or without PNPLA3 rs738409 (C > G) and 490 medical conditions. Table S21. PheWAS of MAFLD with or without PNPLA3 rs738409 (C > G) and causes of death. Table S22. Basic characteristics of the participants with and without genetic susceptibility to MAFLD. Table 23. Basic characteristics of the dead and surviving participants with genetic susceptibility to MAFLD. Table S [file 12916_2023_3080_MOESM4_ESM.docx]

Additional file 1: Table S1. PheWAS using Cox regression was conducted to investigate the relationship between MAFLD and 490 subsequent disease conditions in males and females.

| **Medical conditions** | **Code^*^** | **Total (N = 326,606)** | | **Female (N = 122,626)** | | **Male (N = 203,980)** | |
| --- | --- | --- | --- | --- | --- | --- | --- |
|  |  | **No.^#^** | **HR (95% CI)** | **No.^#^** | **HR (95% CI)** | **No.^#^** | **HR (95% CI)** |
| **Infectious and parasitic** |  |  |  |  |  |  |  |
| Infectious gastroenteritis and colitis | A09 | 7336 | 1.40 (1.35-1.45) | 3362 | 1.48 (1.40-1.56) | 3974 | 1.34 (1.28-1.41) |
| Sepsis | A41 | 5918 | 1.51 (1.45-1.58) | 2062 | 1.68 (1.57-1.80) | 3856 | 1.44 (1.37-1.51) |
| Mycoses | B49 | 2527 | 1.29 (1.21-1.36) | 1063 | 1.39 (1.27-1.53) | 1464 | 1.22 (1.13-1.31) |
| Bacterial infectious agents | B96 | 8188 | 1.49 (1.44-1.54) | 3368 | 1.73 (1.64-1.83) | 4820 | 1.36 (1.30-1.42) |
| **Malignant neoplasms** |  |  |  |  |  |  |  |
| Colon cancer | C18 | 1920 | 1.27 (1.18-1.35) | 600 | 1.05 (0.94-1.18) | 1320 | 1.40 (1.29-1.52) |
| Metastatic cancer | C77 | 7220 | 1.21 (1.17-1.26) | 2835 | 1.20 (1.13-1.27) | 4385 | 1.22 (1.17-1.28) |
| **Benign neoplasms** |  |  |  |  |  |  |  |
| Benign tumors | D10 | 17866 | 1.29 (1.26-1.32) | 6767 | 1.32 (1.27-1.37) | 11099 | 1.27 (1.23-1.31) |
| **Blood system** |  |  |  |  |  |  |  |
| Iron deficiency anaemia | D50 | 6817 | 1.38 (1.33-1.43) | 3234 | 1.68 (1.59-1.78) | 3583 | 1.18 (1.13-1.24) |
| Other anaemias | D51 | 9594 | 1.31 (1.27-1.35) | 4215 | 1.54 (1.47-1.62) | 5379 | 1.17 (1.13-1.22) |
| **Endocrine system disease** |  |  |  |  |  |  |  |
| Hypothyroid conditions | E00 | 7738 | 1.47 (1.42-1.52) | 5434 | 1.57 (1.51-1.64) | 2304 | 1.30 (1.23-1.39) |
| Diabetes | E10 | 19609 | 4.24 (4.11-4.37) | 7661 | 5.42 (5.13-5.72) | 11948 | 3.71 (3.57-3.85) |
| Malnutrition | E40 | 3322 | 1.42 (1.35-1.50) | 1686 | 1.62 (1.50-1.75) | 1636 | 1.26 (1.17-1.35) |
| Obesity | E66 | 21512 | 8.60 (8.26-8.95) | 10851 | 8.88 (8.40-9.39) | 10661 | 8.43 (7.97-8.92) |
| Disorders of lipoprotein metabolism and other lipidaemias | E78 | 26665 | 1.77 (1.74-1.81) | 9397 | 1.89 (1.82-1.95) | 17268 | 1.72 (1.68-1.76) |
| Disorders of mineral metabolism | E83 | 2984 | 1.47 (1.39-1.55) | 1214 | 1.59 (1.45-1.74) | 1770 | 1.39 (1.30-1.50) |
| Volume depletion | E86 | 3874 | 1.43 (1.36-1.50) | 1498 | 1.61 (1.49-1.75) | 2376 | 1.33 (1.25-1.42) |
| Other disorders of fluid, electrolyte and acid-base balance | E87 | 8207 | 1.43 (1.38-1.48) | 3218 | 1.53 (1.45-1.62) | 4989 | 1.37 (1.32-1.43) |
| **Mental disorder** |  |  |  |  |  |  |  |
| Delirium due to known physiological condition | F05 | 2535 | 1.39 (1.31-1.47) | 902 | 1.60 (1.44-1.77) | 1633 | 1.29 (1.20-1.39) |
| Alcohol abuse | F10 | 3267 | 1.55 (1.47-1.64) | 657 | 1.65 (1.45-1.86) | 2610 | 1.53 (1.44-1.62) |
| Tobacco abuse | F17 | 9131 | 1.24 (1.20-1.28) | 3165 | 1.39 (1.32-1.47) | 5966 | 1.17 (1.13-1.22) |
| Depression | F32 | 9717 | 1.63 (1.58-1.68) | 5267 | 1.83 (1.75-1.91) | 4450 | 1.45 (1.39-1.52) |
| Anxiety | F40 | 7324 | 1.37 (1.32-1.42) | 4034 | 1.43 (1.36-1.50) | 3290 | 1.31 (1.24-1.38) |
| **Neural system disease** |  |  |  |  |  |  |  |
| Epilepsia | G40 | 1685 | 1.26 (1.18-1.36) | 635 | 1.42 (1.26-1.60) | 1050 | 1.19 (1.08-1.30) |
| Sleep disorder | G47 | 5120 | 3.71 (3.50-3.93) | 1587 | 4.66 (4.15-5.24) | 3533 | 3.40 (3.18-3.64) |
| Diseases in nerves, nerve roots and nerve plexa | G50 | 7795 | 1.59 (1.54-1.65) | 3681 | 1.59 (1.51-1.68) | 4114 | 1.59 (1.52-1.67) |
| Polyneuropathies | G60 | 2257 | 1.96 (1.83-2.10) | 771 | 2.23 (1.96-2.53) | 1486 | 1.84 (1.69-2.01) |
| Cerebral palsy and other paralytic syndromes | G80 | 1764 | 1.42 (1.32-1.52) | 613 | 1.65 (1.45-1.88) | 1151 | 1.32 (1.21-1.44) |
| **Eye and adnexa disease** |  |  |  |  |  |  |  |
| Disorder of eyelid | H02 | 2477 | 1.27 (1.19-1.34) | 925 | 1.16 (1.06-1.28) | 1552 | 1.34 (1.24-1.44) |
| Disorders of the lens | H25 | 16810 | 1.12 (1.10-1.15) | 7820 | 1.12 (1.08-1.15) | 8990 | 1.13 (1.10-1.16) |
| Disorders of choroid and retina | H30 | 5829 | 1.29 (1.24-1.34) | 2403 | 1.38 (1.30-1.46) | 3426 | 1.23 (1.17-1.30) |
| Visual disturbances and blindness | H53 | 3028 | 1.27 (1.20-1.34) | 1257 | 1.35 (1.24-1.47) | 1771 | 1.22 (1.14-1.30) |
| **Ear dieases** |  |  |  |  |  |  |  |
| Other disorders of the ear | H90 | 4669 | 1.18 (1.13-1.23) | 1641 | 1.25 (1.17-1.35) | 3028 | 1.14 (1.08-1.20) |
| **Cardiovascular disease** |  |  |  |  |  |  |  |
| Chronic rheumatic heart disease | I05 | 3256 | 1.32 (1.25-1.39) | 1135 | 1.47 (1.35-1.62) | 2121 | 1.25 (1.17-1.33) |
| Primary hypertension | I10 | 48720 | 1.93 (1.91-1.96) | 18445 | 2.12 (2.07-2.17) | 30275 | 1.83 (1.80-1.86) |
| Angina pectoris | I20 | 9207 | 1.69 (1.63-1.75) | 2770 | 1.91 (1.79-2.03) | 6437 | 1.61 (1.55-1.67) |
| Acute myocardial infarction | I21 | 5041 | 1.55 (1.48-1.62) | 1110 | 1.60 (1.45-1.75) | 3931 | 1.54 (1.47-1.62) |
| Other ischemic heart disease | I22 | 1760 | 1.57 (1.46-1.70) | 409 | 1.46 (1.25-1.70) | 1351 | 1.61 (1.48-1.76) |
| Chronic ischaemic heart disease | I25 | 15177 | 1.59 (1.55-1.63) | 4230 | 1.82 (1.74-1.92) | 10947 | 1.51 (1.47-1.56) |
| Embolism and thrombosis | I26 | 5561 | 1.48 (1.42-1.55) | 2124 | 1.80 (1.68-1.94) | 3437 | 1.34 (1.27-1.41) |
| Non-rheumatic valve disorders | I34 | 4858 | 1.34 (1.28-1.39) | 1605 | 1.48 (1.37-1.60) | 3253 | 1.28 (1.21-1.34) |
| Atrial fibrillation and flutter | I48 | 12662 | 1.45 (1.41-1.49) | 3773 | 1.64 (1.56-1.73) | 8889 | 1.39 (1.34-1.43) |
| Other cardiac arrhythmias | I49 | 9066 | 1.35 (1.30-1.39) | 2595 | 1.39 (1.31-1.47) | 6471 | 1.33 (1.28-1.38) |
| Heart failure | I50 | 6928 | 1.91 (1.84-1.99) | 2210 | 2.29 (2.12-2.47) | 4718 | 1.77 (1.69-1.86) |
| Complications and ill-defined descriptions of heart disease | I51 | 6566 | 1.70 (1.63-1.77) | 2044 | 2.04 (1.89-2.20) | 4522 | 1.58 (1.51-1.65) |
| Stroke | I60 | 4278 | 1.30 (1.24-1.36) | 1355 | 1.43 (1.32-1.55) | 2923 | 1.24 (1.18-1.31) |
| Other cerebrovascular diseases | I65 | 4012 | 1.37 (1.31-1.44) | 1350 | 1.41 (1.30-1.53) | 2662 | 1.35 (1.27-1.43) |
| Aneurysm and dissection | I71 | 1856 | 1.34 (1.25-1.44) | 294 | 1.21 (1.02-1.43) | 1562 | 1.37 (1.27-1.48) |
| Peripheral vascular disease | I73 | 2716 | 1.23 (1.16-1.30) | 850 | 1.15 (1.04-1.27) | 1866 | 1.26 (1.18-1.35) |
| Varicose veins of lower extremities | I83 | 2465 | 1.19 (1.12-1.26) | 1073 | 1.22 (1.12-1.33) | 1392 | 1.17 (1.08-1.26) |
| Hypotension | I95 | 5918 | 1.25 (1.20-1.30) | 2100 | 1.29 (1.21-1.38) | 3818 | 1.23 (1.17-1.29) |
| **Respiratory system disease** |  |  |  |  |  |  |  |
| Pneumonia | J12 | 10625 | 1.46 (1.42-1.50) | 3723 | 1.63 (1.55-1.72) | 6902 | 1.38 (1.33-1.43) |
| Other acute lower respiratory infections | J20 | 5923 | 1.62 (1.56-1.69) | 2335 | 1.81 (1.70-1.94) | 3588 | 1.51 (1.44-1.60) |
| Chronic obstructive pulmonary disease | J44 | 7598 | 1.43 (1.38-1.48) | 2810 | 1.48 (1.39-1.56) | 4788 | 1.41 (1.35-1.47) |
| Asthma | J45 | 11842 | 1.46 (1.42-1.50) | 5713 | 1.72 (1.65-1.79) | 6129 | 1.28 (1.24-1.33) |
| Pleural effusion | J90 | 5442 | 1.29 (1.24-1.35) | 1883 | 1.38 (1.29-1.48) | 3559 | 1.25 (1.19-1.31) |
| Other diseases of the respiratory system | J95 | 4666 | 1.33 (1.28-1.39) | 1779 | 1.40 (1.30-1.50) | 2887 | 1.30 (1.23-1.37) |
| Respiratory failure | J96 | 3334 | 1.62 (1.53-1.71) | 1203 | 1.92 (1.75-2.12) | 2131 | 1.49 (1.39-1.59) |
| **Digestive system disease** |  |  |  |  |  |  |  |
| Disease of hard tissue of teeth | K00 | 1798 | 1.30 (1.21-1.39) | 740 | 1.45 (1.29-1.62) | 1058 | 1.21 (1.11-1.33) |
| Oesophagitis | K20 | 3850 | 1.25 (1.19-1.31) | 1533 | 1.45 (1.34-1.57) | 2317 | 1.15 (1.08-1.22) |
| Gastro-oesophageal reflux disease | K21 | 15589 | 1.39 (1.36-1.42) | 7187 | 1.60 (1.54-1.66) | 8402 | 1.25 (1.21-1.29) |
| Other diseases of oesophagus | K22 | 5536 | 1.24 (1.19-1.29) | 1927 | 1.39 (1.30-1.49) | 3609 | 1.17 (1.11-1.23) |
| Gastric ulcer | K25 | 2387 | 1.42 (1.33-1.51) | 981 | 1.48 (1.34-1.63) | 1406 | 1.38 (1.27-1.49) |
| Gastritis and duodenitis | K29 | 13686 | 1.26 (1.23-1.29) | 5883 | 1.34 (1.29-1.40) | 7803 | 1.20 (1.16-1.24) |
| Disease of stomach and duodenum | K31 | 5681 | 1.37 (1.32-1.43) | 2695 | 1.48 (1.40-1.57) | 2986 | 1.28 (1.21-1.35) |
| Umbilical hernia | K42 | 2656 | 2.75 (2.56-2.96) | 668 | 4.21 (3.54-5.00) | 1988 | 2.47 (2.28-2.68) |
| Ventral hernia | K43 | 1994 | 1.99 (1.85-2.15) | 891 | 2.50 (2.22-2.83) | 1103 | 1.71 (1.55-1.88) |
| Diaphragmatic hernia | K44 | 13407 | 1.31 (1.27-1.34) | 6451 | 1.58 (1.52-1.64) | 6956 | 1.13 (1.09-1.17) |
| Noninfective gastroenteritis and colitis | K52 | 5150 | 1.28 (1.23-1.34) | 2415 | 1.39 (1.31-1.48) | 2735 | 1.20 (1.14-1.27) |
| Diverticular disease of intestine | K57 | 20017 | 1.40 (1.37-1.43) | 8152 | 1.43 (1.38-1.47) | 11865 | 1.39 (1.35-1.43) |
| Irritable bowel syndrome | K58 | 3010 | 1.27 (1.21-1.34) | 1936 | 1.41 (1.32-1.51) | 1074 | 1.09 (1.00-1.18) |
| Other functional intestinal disorders | K59 | 8857 | 1.23 (1.20-1.27) | 3666 | 1.27 (1.21-1.33) | 5191 | 1.21 (1.16-1.26) |
| Other diseases of anus and rectum | K62 | 8721 | 1.22 (1.18-1.26) | 3011 | 1.27 (1.20-1.34) | 5710 | 1.20 (1.15-1.24) |
| Other diseases of intestine | K63 | 11385 | 1.39 (1.35-1.43) | 3821 | 1.35 (1.29-1.42) | 7564 | 1.41 (1.36-1.45) |
| Haemorrhoids and perianal venous thrombosis | K64 | 6169 | 1.11 (1.08-1.16) | 2367 | 1.17 (1.10-1.24) | 3802 | 1.08 (1.04-1.14) |
| Other disorders of peritoneum | K66 | 2837 | 1.60 (1.51-1.70) | 1370 | 1.92 (1.76-2.10) | 1467 | 1.39 (1.28-1.50) |
| Other diseases of liver | K76 | 5167 | 2.67 (2.54-2.81) | 2258 | 3.05 (2.81-3.31) | 2909 | 2.44 (2.28-2.60) |
| Cholelithiasis | K80 | 7589 | 2.19 (2.11-2.28) | 4205 | 2.56 (2.42-2.71) | 3384 | 1.87 (1.77-1.98) |
| Disease of gallbladder and biliary tract | K82 | 2841 | 1.84 (1.73-1.95) | 1424 | 2.01 (1.83-2.19) | 1417 | 1.69 (1.55-1.84) |
| Other disease of digestive system | K92 | 6861 | 1.28 (1.24-1.33) | 2510 | 1.31 (1.23-1.39) | 4351 | 1.26 (1.21-1.32) |
| **Skin and subcutaneous disease** |  |  |  |  |  |  |  |
| Skin and subcutaneous infections | L01 | 6610 | 1.93 (1.85-2.01) | 2488 | 2.31 (2.15-2.47) | 4122 | 1.76 (1.67-1.85) |
| Dermatitis | L20 | 2716 | 1.40 (1.32-1.48) | 1144 | 1.59 (1.45-1.75) | 1572 | 1.28 (1.19-1.38) |
| Papulosquamous disorders | L40 | 2079 | 1.55 (1.45-1.66) | 832 | 1.66 (1.48-1.85) | 1247 | 1.49 (1.37-1.63) |
| Pressure ulcer | L89 | 1868 | 1.54 (1.43-1.65) | 700 | 1.98 (1.74-2.25) | 1168 | 1.35 (1.24-1.48) |
| Disorder of the skin and subcutaneous tissue | L98 | 2651 | 1.15 (1.09-1.22) | 962 | 1.23 (1.12-1.35) | 1689 | 1.12 (1.04-1.20) |
| **Musculoskeletal and connective tissue disease** |  |  |  |  |  |  |  |
| Autoimmune arthritis | M05 | 3037 | 1.53 (1.45-1.62) | 1733 | 1.68 (1.56-1.82) | 1304 | 1.37 (1.26-1.48) |
| Other inflammatory arthritis | M10 | 15579 | 1.92 (1.87-1.98) | 6538 | 1.86 (1.78-1.93) | 9041 | 1.98 (1.91-2.05) |
| Osteoarthritis | M15 | 28395 | 1.64 (1.61-1.67) | 13789 | 1.74 (1.69-1.79) | 14606 | 1.57 (1.53-1.61) |
| Other joint disorders | M20 | 13659 | 1.37 (1.34-1.41) | 6341 | 1.40 (1.35-1.45) | 7318 | 1.36 (1.31-1.40) |
| Systemic connective tissue disorders | M30 | 2191 | 1.26 (1.18-1.34) | 1276 | 1.28 (1.18-1.40) | 915 | 1.22 (1.11-1.34) |
| Deforming dorsopathies | M40 | 2305 | 1.31 (1.23-1.39) | 1314 | 1.31 (1.21-1.42) | 991 | 1.31 (1.19-1.44) |
| Spondylosis | M47 | 6311 | 1.54 (1.48-1.60) | 3071 | 1.50 (1.42-1.58) | 3240 | 1.58 (1.50-1.67) |
| Spondylopathy | M48 | 4091 | 1.67 (1.59-1.76) | 1768 | 1.66 (1.54-1.79) | 2323 | 1.68 (1.58-1.80) |
| Other intervertebral disc disorders | M51 | 4502 | 1.58 (1.51-1.66) | 2068 | 1.65 (1.54-1.77) | 2434 | 1.53 (1.44-1.63) |
| Dorsalgia | M54 | 8760 | 1.54 (1.49-1.60) | 4162 | 1.60 (1.52-1.68) | 4598 | 1.50 (1.43-1.57) |
| Disorders of synovium and tendons | M65 | 3639 | 1.34 (1.28-1.41) | 1677 | 1.34 (1.24-1.44) | 1962 | 1.35 (1.26-1.44) |
| Shoulder lesion | M75 | 4103 | 1.39 (1.33-1.46) | 1697 | 1.50 (1.39-1.61) | 2406 | 1.33 (1.25-1.41) |
| Other soft tissue disorders, not elsewhere classified | M79 | 7864 | 1.75 (1.69-1.82) | 4048 | 1.97 (1.87-2.08) | 3816 | 1.57 (1.50-1.66) |
| **Genitourinary system disease** |  |  |  |  |  |  |  |
| Obstructive and reflux uropathy | N13 | 2170 | 1.31 (1.23-1.39) | 654 | 1.56 (1.38-1.77) | 1516 | 1.22 (1.13-1.31) |
| Acute renal failure | N17 | 9834 | 1.90 (1.84-1.96) | 3304 | 2.27 (2.14-2.42) | 6530 | 1.75 (1.68-1.82) |
| Chronic kidney disease | N18 | 9060 | 2.01 (1.94-2.08) | 3804 | 2.23 (2.11-2.36) | 5256 | 1.87 (1.79-1.96) |
| Urolithiasis | N20 | 2924 | 1.53 (1.45-1.62) | 727 | 1.81 (1.60-2.04) | 2197 | 1.46 (1.37-1.56) |
| Other disorders of kidney and ureter | N29 | 2810 | 1.56 (1.47-1.65) | 881 | 1.74 (1.56-1.94) | 1929 | 1.48 (1.38-1.59) |
| Bladder disorder | N32 | 4485 | 1.17 (1.12-1.22) | 1189 | 1.52 (1.39-1.66) | 3296 | 1.08 (1.03-1.14) |
| Disorders of urinary system, possibly infection | N39 | 9880 | 1.49 (1.45-1.54) | 4699 | 1.64 (1.57-1.72) | 5181 | 1.38 (1.32-1.44) |
| Female genital prolapse | N81 | 2547 | 1.21 (1.14-1.28) | 2546 | 1.22 (1.15-1.29) | - | - |
| Polyp of female genital tract | N84 | 1916 | 1.75 (1.63-1.89) | 1916 | 1.77 (1.64-1.90) | - | - |
| Menopausal and perimenopausal disorders | N95 | 1876 | 1.49 (1.39-1.60) | 1876 | 1.50 (1.40-1.61) | - | - |
| **Unnatural cause** |  |  |  |  |  |  |  |
| Injuries due to external causes | S00 | 14888 | 1.06 (1.04-1.09) | 6187 | 1.09 (1.05-1.13) | 8701 | 1.05 (1.01-1.08) |
| Complications due to medical treatment | T80 | 11766 | 1.42 (1.38-1.46) | 4605 | 1.54 (1.47-1.61) | 7161 | 1.35 (1.31-1.40) |
| Falls | W00 | 10293 | 1.14 (1.11-1.17) | 4858 | 1.11 (1.07-1.16) | 5435 | 1.16 (1.12-1.21) |
| Other external causes of accidental injury | W20 | 5349 | 1.17 (1.12-1.22) | 1838 | 1.29 (1.20-1.38) | 3511 | 1.11 (1.06-1.17) |
| External causes of morbidity related to medical treatment | Y40 | 15399 | 1.38 (1.35-1.41) | 5822 | 1.47 (1.41-1.53) | 9577 | 1.33 (1.29-1.37) |
| External causes of morbidity related to other conditions | Y95 | 2479 | 1.46 (1.37-1.55) | 814 | 1.77 (1.58-1.98) | 1665 | 1.34 (1.25-1.45) |

A total of 113 disease conditions were confirmed to be significantly associated with MAFLD after Bonferroni correction. Significantly higher HRs (95%CIs without overlap) were marked in red in males or females . HR, hazard ratio; CI, confidence interval. MAFLD, Metabolic dysfunction-associated fatty liver disease.

^*^Combined ICD-10 codes derived from the original ICD-10 code and are displayed in the Additional file 1.

^#^Number of the participants diagnosed with the corresponding diseases.

Additional file 1: Table S2. Temporal disease pairs with a significantly increased risk of disease 2 (D2) after disease 1 (D1) in MAFLD individuals.

| **D1→D2 code^*^** | **D1 description** | **D2 description** | **No.^#^** | **OR (95% CI)** | **Percentage^&^** |
| --- | --- | --- | --- | --- | --- |
| C77→A41 | Metastatic cancer | Sepsis | 999 | 4.63 (3.99-5.36) | 11.30% |
| C77→D51 | Metastatic cancer | Other anaemias | 871 | 4.92 (4.19-5.78) | 11.27% |
| C77→E87 | Metastatic cancer | Other disorders of fluid, electrolyte and acid-base balance | 827 | 4.47 (3.81-5.24) | 14.95% |
| C77→J12 | Metastatic cancer | Pneumonia | 999 | 4.83 (4.16-5.61) | 14.29% |
| C77→K59 | Metastatic cancer | Other functional intestinal disorders | 844 | 4.35 (3.72-5.09) | 14.67% |
| C77→N17 | Metastatic cancer | Acute renal failure | 941 | 5.44 (4.63-6.40) | 14.99% |
| C77→Y40 | Metastatic cancer | External causes of morbidity related to medical treatment | 1335 | 5.88 (5.11-6.77) | 11.27% |
| D10→A09 | Benign tumors | Infectious gastroenteritis and colitis | 874 | 1.79 (1.60-2.00) | 13.54% |
| D10→A41 | Benign tumors | Sepsis | 853 | 1.50 (1.35-1.67) | 3.78% |
| D10→B96 | Benign tumors | Bacterial infectious agents | 839 | 1.61 (1.44-1.79) | 6.65% |
| D10→C77 | Benign tumors | Metastatic cancer | 939 | 1.63 (1.47-1.81) | 6.34% |
| D10→D51 | Benign tumors | Other anaemias | 1044 | 1.67 (1.52-1.85) | 13.88% |
| D10→E66 | Benign tumors | Obesity | 1714 | 2.05 (1.89-2.23) | 16.49% |
| D10→E87 | Benign tumors | Other disorders of fluid, electrolyte and acid-base balance | 1076 | 1.70 (1.54-1.88) | 5.87% |
| D10→H25 | Benign tumors | Disorders of the lens | 1243 | 1.74 (1.59-1.91) | 2.89% |
| D10→J12 | Benign tumors | Pneumonia | 1236 | 1.76 (1.61-1.93) | 4.29% |
| D10→K59 | Benign tumors | Other functional intestinal disorders | 948 | 1.80 (1.62-2.00) | 12.94% |
| D10→K64 | Benign tumors | Haemorrhoids and perianal venous thrombosis | 889 | 1.76 (1.58-1.96) | 35.41% |
| D10→M10 | Benign tumors | Other inflammatory arthritis | 1335 | 1.85 (1.69-2.02) | 12.77% |
| D10→N17 | Benign tumors | Acute renal failure | 1266 | 1.68 (1.54-1.84) | 5.97% |
| D10→N18 | Benign tumors | Chronic kidney disease | 970 | 1.76 (1.58-1.95) | 11.23% |
| D10→N39 | Benign tumors | Disorders of urinary system, possibly infection | 954 | 1.64 (1.48-1.82) | 5.84% |
| D10→S00 | Benign tumors | Injuries due to external causes | 1099 | 1.74 (1.58-1.92) | 3.47% |
| D10→T80 | Benign tumors | Complications due to medical treatment | 1248 | 1.78 (1.62-1.95) | 6.99% |
| D10→W00 | Benign tumors | Falls | 818 | 1.58 (1.42-1.77) | 3.34% |
| D10→Y40 | Benign tumors | External causes of morbidity related to medical treatment | 1648 | 1.88 (1.73-2.04) | 6.65% |
| D51→A41 | Other anaemias | Sepsis | 972 | 3.20 (2.81-3.64) | 14.20% |
| D51→E87 | Other anaemias | Other disorders of fluid, electrolyte and acid-base balance | 1189 | 3.60 (3.19-4.07) | 18.09% |
| D51→J12 | Other anaemias | Pneumonia | 1214 | 3.42 (3.04-3.85) | 14.44% |
| D51→K59 | Other anaemias | Other functional intestinal disorders | 863 | 3.13 (2.73-3.58) | 12.70% |
| D51→N17 | Other anaemias | Acute renal failure | 1259 | 3.84 (3.40-4.33) | 18.94% |
| E00→E66 | Hypothyroid conditions | Obesity | 955 | 3.33 (2.92-3.80) | 30.40% |
| E00→M15 | Hypothyroid conditions | Osteoarthritis | 892 | 3.69 (3.20-4.25) | 36.51% |
| E10→A09 | Diabetes | Infectious gastroenteritis and colitis | 1199 | 1.53 (1.40-1.67) | 15.89% |
| E10→A41 | Diabetes | Sepsis | 1163 | 1.55 (1.41-1.70) | 12.34% |
| E10→B96 | Diabetes | Bacterial infectious agents | 1242 | 1.51 (1.39-1.65) | 12.39% |
| E10→C77 | Diabetes | Metastatic cancer | 918 | 1.52 (1.37-1.68) | 16.28% |
| E10→D10 | Diabetes | Benign tumors | 1490 | 1.76 (1.61-1.91) | 20.53% |
| E10→D50 | Diabetes | Iron deficiency anaemia | 1206 | 1.50 (1.37-1.64) | 17.56% |
| E10→D51 | Diabetes | Other anaemias | 1637 | 1.53 (1.42-1.66) | 14.23% |
| E10→E66 | Diabetes | Obesity | 2367 | 1.94 (1.81-2.08) | 29.18% |
| E10→E78 | Diabetes | Disorders of lipoprotein metabolism and other lipidaemias | 2829 | 2.39 (2.23-2.55) | 36.50% |
| E10→E87 | Diabetes | Other disorders of fluid, electrolyte and acid-base balance | 1655 | 1.54 (1.42-1.66) | 15.21% |
| E10→F32 | Diabetes | Depression | 967 | 1.62 (1.46-1.79) | 25.09% |
| E10→H25 | Diabetes | Disorders of the lens | 1815 | 1.69 (1.57-1.83) | 26.78% |
| E10→H30 | Diabetes | Disorders of choroid and retina | 1002 | 1.45 (1.31-1.60) | 27.09% |
| E10→I20 | Diabetes | Angina pectoris | 1012 | 1.69 (1.52-1.87) | 27.73% |
| E10→I25 | Diabetes | Chronic ischaemic heart disease | 1685 | 1.92 (1.77-2.08) | 28.49% |
| E10→I48 | Diabetes | Atrial fibrillation and flutter | 1426 | 1.61 (1.48-1.75) | 25.26% |
| E10→I49 | Diabetes | Other cardiac arrhythmias | 1210 | 1.51 (1.38-1.66) | 20.39% |
| E10→I50 | Diabetes | Heart failure | 1182 | 1.47 (1.34-1.61) | 17.20% |
| E10→I51 | Diabetes | Complications and ill-defined descriptions of heart disease | 1082 | 1.40 (1.28-1.54) | 15.92% |
| E10→I95 | Diabetes | Hypotension | 1004 | 1.41 (1.28-1.56) | 13.40% |
| E10→J12 | Diabetes | Pneumonia | 1736 | 1.55 (1.44-1.67) | 15.78% |
| E10→J20 | Diabetes | Other acute Lower respiratory infections | 834 | 1.44 (1.30-1.60) | 15.48% |
| E10→J44 | Diabetes | Chronic obstructive pulmonary disease | 883 | 1.36 (1.23-1.51) | 27.93% |
| E10→J90 | Diabetes | Pleural effusion | 895 | 1.37 (1.24-1.52) | 11.04% |
| E10→K21 | Diabetes | Gastro-oesophageal reflux disease | 1385 | 1.56 (1.43-1.70) | 20.18% |
| E10→K29 | Diabetes | Gastritis and duodenitis | 1516 | 1.55 (1.43-1.68) | 15.08% |
| E10→K44 | Diabetes | Diaphragmatic hernia | 1153 | 1.66 (1.51-1.82) | 17.16% |
| E10→K57 | Diabetes | Diverticular disease of intestine | 1841 | 1.74 (1.61-1.87) | 19.91% |
| E10→K59 | Diabetes | Other functional intestinal disorders | 1285 | 1.54 (1.41-1.67) | 12.91% |
| E10→K63 | Diabetes | Other diseases of intestine | 1134 | 1.64 (1.49-1.80) | 15.02% |
| E10→K76 | Diabetes | Other diseases of liver | 936 | 1.54 (1.39-1.71) | 21.80% |
| E10→L01 | Diabetes | Skin and subcutaneous infections | 853 | 1.46 (1.32-1.63) | 21.20% |
| E10→M10 | Diabetes | Other inflammatory arthritis | 1667 | 1.71 (1.58-1.85) | 23.73% |
| E10→M15 | Diabetes | Osteoarthritis | 2129 | 2.05 (1.90-2.21) | 28.82% |
| E10→M20 | Diabetes | Other joint disorders | 995 | 1.62 (1.46-1.79) | 21.44% |
| E10→M54 | Diabetes | Dorsalgia | 991 | 1.52 (1.37-1.68) | 16.99% |
| E10→M79 | Diabetes | Other soft tissue disorders, not elsewhere classified | 877 | 1.52 (1.37-1.69) | 17.04% |
| E10→N17 | Diabetes | Acute renal failure | 2053 | 1.65 (1.53-1.76) | 15.12% |
| E10→N18 | Diabetes | Chronic kidney disease | 1578 | 1.56 (1.44-1.68) | 22.58% |
| E10→N39 | Diabetes | Disorders of urinary system, possibly infection | 1429 | 1.59 (1.46-1.73) | 14.89% |
| E10→S00 | Diabetes | Injuries due to external causes | 1371 | 1.63 (1.50-1.78) | 23.31% |
| E10→T80 | Diabetes | Complications due to medical treatment | 1493 | 1.59 (1.46-1.72) | 9.21% |
| E10→W00 | Diabetes | Falls | 1131 | 1.56 (1.42-1.72) | 21.65% |
| E10→Y40 | Diabetes | External causes of morbidity related to medical treatment | 1989 | 1.70 (1.58-1.83) | 13.52% |
| E66→A09 | Obesity | Infectious gastroenteritis and colitis | 1008 | 1.40 (1.28-1.54) | 8.21% |
| E66→A41 | Obesity | Sepsis | 1019 | 1.50 (1.36-1.65) | 10.29% |
| E66→B96 | Obesity | Bacterial infectious agents | 1212 | 1.56 (1.42-1.70) | 8.61% |
| E66→D50 | Obesity | Iron deficiency anaemia | 970 | 1.40 (1.27-1.55) | 10.66% |
| E66→D51 | Obesity | Other anaemias | 1257 | 1.45 (1.33-1.58) | 13.96% |
| E66→E87 | Obesity | Other disorders of fluid, electrolyte and acid-base balance | 1334 | 1.46 (1.34-1.59) | 14.78% |
| E66→F40 | Obesity | Anxiety | 979 | 1.48 (1.34-1.63) | 24.30% |
| E66→I49 | Obesity | Other cardiac arrhythmias | 1068 | 1.59 (1.45-1.76) | 22.02% |
| E66→I50 | Obesity | Heart failure | 1096 | 1.55 (1.41-1.71) | 18.18% |
| E66→I51 | Obesity | Complications and ill-defined descriptions of heart disease | 1046 | 1.53 (1.39-1.68) | 21.88% |
| E66→I95 | Obesity | Hypotension | 922 | 1.42 (1.28-1.57) | 16.32% |
| E66→J12 | Obesity | Pneumonia | 1484 | 1.50 (1.39-1.63) | 14.87% |
| E66→K59 | Obesity | Other functional intestinal disorders | 1190 | 1.51 (1.38-1.65) | 13.40% |
| E66→K64 | Obesity | Haemorrhoids and perianal venous thrombosis | 903 | 1.38 (1.25-1.53) | 10.29% |
| E66→L01 | Obesity | Skin and subcutaneous infections | 904 | 1.48 (1.33-1.64) | 11.01% |
| E66→N17 | Obesity | Acute renal failure | 1625 | 1.58 (1.46-1.71) | 13.55% |
| E66→N18 | Obesity | Chronic kidney disease | 1248 | 1.65 (1.50-1.80) | 23.05% |
| E66→N39 | Obesity | Disorders of urinary system, possibly infection | 1206 | 1.63 (1.49-1.79) | 13.59% |
| E66→T80 | Obesity | Complications due to medical treatment | 1806 | 1.68 (1.56-1.81) | 15.65% |
| E66→Y40 | Obesity | External causes of morbidity related to medical treatment | 2082 | 1.79 (1.66-1.92) | 16.81% |
| E78→D10 | Disorders of lipoprotein metabolism and other lipidaemias | Benign tumors | 2128 | 1.23 (1.16-1.31) | 12.35% |
| E78→E66 | Disorders of lipoprotein metabolism and other lipidaemias | Obesity | 2831 | 1.34 (1.27-1.42) | 30.02% |
| E78→F32 | Disorders of lipoprotein metabolism and other lipidaemias | Depression | 1271 | 1.20 (1.10-1.30) | 28.30% |
| E78→H25 | Disorders of lipoprotein metabolism and other lipidaemias | Disorders of the lens | 2277 | 1.16 (1.09-1.23) | 18.51% |
| E78→I20 | Disorders of lipoprotein metabolism and other lipidaemias | Angina pectoris | 1743 | 1.18 (1.10-1.27) | 36.91% |
| E78→I25 | Disorders of lipoprotein metabolism and other lipidaemias | Chronic ischaemic heart disease | 2507 | 1.40 (1.32-1.49) | 38.41% |
| E78→I48 | Disorders of lipoprotein metabolism and other lipidaemias | Atrial fibrillation and flutter | 1989 | 1.16 (1.09-1.24) | 26.23% |
| E78→K21 | Disorders of lipoprotein metabolism and other lipidaemias | Gastro-oesophageal reflux disease | 2066 | 1.19 (1.11-1.27) | 24.53% |
| E78→K57 | Disorders of lipoprotein metabolism and other lipidaemias | Diverticular disease of intestine | 2500 | 1.18 (1.12-1.25) | 10.26% |
| E78→M10 | Disorders of lipoprotein metabolism and other lipidaemias | Other inflammatory arthritis | 2260 | 1.18 (1.11-1.25) | 25.49% |
| E78→Y40 | Disorders of lipoprotein metabolism and other lipidaemias | External causes of morbidity related to medical treatment | 2812 | 1.24 (1.18-1.32) | 14.81% |
| F17→E66 | Tobacco abuse | Obesity | 942 | 3.71 (3.23-4.26) | 29.67% |
| F17→E87 | Tobacco abuse | Other disorders of fluid, electrolyte and acid-base balance | 829 | 3.50 (3.03-4.04) | 10.88% |
| F17→J12 | Tobacco abuse | Pneumonia | 970 | 3.75 (3.27-4.30) | 16.80% |
| F17→J44 | Tobacco abuse | Chronic obstructive pulmonary disease | 858 | 3.92 (3.38-4.54) | 28.23% |
| F17→K57 | Tobacco abuse | Diverticular disease of intestine | 820 | 3.89 (3.34-4.52) | 13.02% |
| F17→N17 | Tobacco abuse | Acute renal failure | 958 | 3.33 (2.92-3.79) | 11.77% |
| F17→Y40 | Tobacco abuse | External causes of morbidity related to medical treatment | 1016 | 3.65 (3.20-4.17) | 10.82% |
| F32→F40 | Depression | Anxiety | 1094 | 3.89 (3.41-4.44) | 40.08% |
| F32→J12 | Depression | Pneumonia | 866 | 3.67 (3.18-4.24) | 14.95% |
| G50→E66 | Diseases in nerves, nerve roots and nerve plexa | Obesity | 1021 | 5.32 (4.56-6.20) | 17.55% |
| H25→E87 | Disorders of the lens | Other disorders of fluid, electrolyte and acid-base balance | 1032 | 1.23 (1.12-1.35) | 1.67% |
| H25→I49 | Disorders of the lens | Other cardiac arrhythmias | 847 | 1.24 (1.12-1.38) | 4.74% |
| H25→J12 | Disorders of the lens | Pneumonia | 1119 | 1.23 (1.13-1.34) | 0.91% |
| H25→N17 | Disorders of the lens | Acute renal failure | 1201 | 1.24 (1.14-1.35) | 0.96% |
| H25→N18 | Disorders of the lens | Chronic kidney disease | 1070 | 1.23 (1.12-1.34) | 12.35% |
| I20→D51 | Angina pectoris | Other anaemias | 818 | 2.89 (2.53-3.31) | 11.02% |
| I20→E66 | Angina pectoris | Obesity | 1131 | 4.44 (3.87-5.08) | 23.92% |
| I20→H25 | Angina pectoris | Disorders of the lens | 886 | 3.25 (2.83-3.72) | 14.22% |
| I20→I48 | Angina pectoris | Atrial fibrillation and flutter | 980 | 3.28 (2.88-3.73) | 18.53% |
| I20→I50 | Angina pectoris | Heart failure | 862 | 3.52 (3.05-4.05) | 17.85% |
| I20→I51 | Angina pectoris | Complications and ill-defined descriptions of heart disease | 844 | 3.15 (2.74-3.61) | 20.36% |
| I20→J12 | Angina pectoris | Pneumonia | 856 | 3.24 (2.82-3.72) | 9.11% |
| I20→K21 | Angina pectoris | Gastro-oesophageal reflux disease | 858 | 3.59 (3.11-4.14) | 15.18% |
| I20→K57 | Angina pectoris | Diverticular disease of intestine | 897 | 3.53 (3.07-4.06) | 8.29% |
| I20→M10 | Angina pectoris | Other inflammatory arthritis | 872 | 3.29 (2.87-3.78) | 16.72% |
| I20→N17 | Angina pectoris | Acute renal failure | 930 | 2.82 (2.49-3.20) | 8.67% |
| I20→N18 | Angina pectoris | Chronic kidney disease | 835 | 3.34 (2.90-3.85) | 15.90% |
| I20→T80 | Angina pectoris | Complications due to medical treatment | 840 | 3.28 (2.85-3.77) | 12.49% |
| I20→Y40 | Angina pectoris | External causes of morbidity related to medical treatment | 1192 | 3.49 (3.09-3.93) | 12.61% |
| I21→I20 | Acute myocardial infarction | Angina pectoris | 830 | 10.78 (8.53-13.61) | 21.34% |
| I25→B96 | Chronic ischaemic heart disease | Bacterial infectious agents | 930 | 1.81 (1.62-2.01) | 6.73% |
| I25→D50 | Chronic ischaemic heart disease | Iron deficiency anaemia | 860 | 1.68 (1.51-1.88) | 7.74% |
| I25→D51 | Chronic ischaemic heart disease | Other anaemias | 1212 | 1.80 (1.63-1.97) | 8.57% |
| I25→E66 | Chronic ischaemic heart disease | Obesity | 1667 | 2.27 (2.09-2.48) | 26.28% |
| I25→E87 | Chronic ischaemic heart disease | Other disorders of fluid, electrolyte and acid-base balance | 1290 | 1.79 (1.64-1.96) | 10.53% |
| I25→H25 | Chronic ischaemic heart disease | Disorders of the lens | 1326 | 1.89 (1.72-2.07) | 9.67% |
| I25→I20 | Chronic ischaemic heart disease | Angina pectoris | 2268 | 2.88 (2.66-3.13) | 41.25% |
| I25→I21 | Chronic ischaemic heart disease | Acute myocardial infarction | 1364 | 85.25 (52.08-139.55) | 52.71% |
| I25→I34 | Chronic ischaemic heart disease | Non-rheumatic valve disorders | 852 | 1.76 (1.57-1.97) | 32.88% |
| I25→I48 | Chronic ischaemic heart disease | Atrial fibrillation and flutter | 1651 | 2.00 (1.84-2.17) | 19.32% |
| I25→I49 | Chronic ischaemic heart disease | Other cardiac arrhythmias | 1346 | 1.85 (1.69-2.03) | 26.57% |
| I25→I50 | Chronic ischaemic heart disease | Heart failure | 1433 | 1.87 (1.71-2.04) | 24.63% |
| I25→I51 | Chronic ischaemic heart disease | Complications and ill-defined descriptions of heart disease | 1379 | 1.83 (1.68-2.00) | 29.00% |
| I25→I95 | Chronic ischaemic heart disease | Hypotension | 1106 | 1.71 (1.55-1.88) | 12.85% |
| I25→J12 | Chronic ischaemic heart disease | Pneumonia | 1364 | 1.78 (1.63-1.94) | 9.94% |
| I25→J90 | Chronic ischaemic heart disease | Pleural effusion | 957 | 1.57 (1.42-1.74) | 11.98% |
| I25→K59 | Chronic ischaemic heart disease | Other functional intestinal disorders | 996 | 1.72 (1.55-1.90) | 6.91% |
| I25→M10 | Chronic ischaemic heart disease | Other inflammatory arthritis | 1308 | 1.92 (1.75-2.11) | 15.05% |
| I25→N17 | Chronic ischaemic heart disease | Acute renal failure | 1512 | 1.82 (1.68-1.99) | 9.33% |
| I25→N18 | Chronic ischaemic heart disease | Chronic kidney disease | 1274 | 1.90 (1.73-2.09) | 16.37% |
| I25→N39 | Chronic ischaemic heart disease | Disorders of urinary system, possibly infection | 1005 | 1.95 (1.75-2.17) | 7.94% |
| I25→S00 | Chronic ischaemic heart disease | Injuries due to external causes | 1103 | 1.78 (1.61-1.97) | 9.96% |
| I25→T80 | Chronic ischaemic heart disease | Complications due to medical treatment | 1395 | 1.95 (1.78-2.14) | 9.51% |
| I25→W00 | Chronic ischaemic heart disease | Falls | 857 | 1.75 (1.57-1.96) | 9.54% |
| I25→Y40 | Chronic ischaemic heart disease | External causes of morbidity related to medical treatment | 1954 | 2.14 (1.97-2.31) | 9.99% |
| I48→A41 | Atrial fibrillation and flutter | Sepsis | 898 | 1.81 (1.62-2.02) | 21.57% |
| I48→B96 | Atrial fibrillation and flutter | Bacterial infectious agents | 914 | 1.81 (1.62-2.02) | 13.80% |
| I48→D51 | Atrial fibrillation and flutter | Other anaemias | 969 | 1.84 (1.66-2.05) | 12.81% |
| I48→E87 | Atrial fibrillation and flutter | Other disorders of fluid, electrolyte and acid-base balance | 1269 | 2.06 (1.87-2.27) | 24.32% |
| I48→I05 | Atrial fibrillation and flutter | Chronic rheumatic heart disease | 900 | 1.92 (1.72-2.15) | 25.38% |
| I48→I34 | Atrial fibrillation and flutter | Non-rheumatic valve disorders | 862 | 2.00 (1.78-2.25) | 23.57% |
| I48→I49 | Atrial fibrillation and flutter | Other cardiac arrhythmias | 1244 | 2.31 (2.09-2.55) | 28.19% |
| I48→I50 | Atrial fibrillation and flutter | Heart failure | 1609 | 2.37 (2.17-2.59) | 30.58% |
| I48→I51 | Atrial fibrillation and flutter | Complications and ill-defined descriptions of heart disease | 1415 | 2.22 (2.02-2.44) | 26.15% |
| I48→I95 | Atrial fibrillation and flutter | Hypotension | 986 | 1.94 (1.75-2.16) | 23.32% |
| I48→J12 | Atrial fibrillation and flutter | Pneumonia | 1376 | 2.25 (2.04-2.47) | 26.99% |
| I48→J90 | Atrial fibrillation and flutter | Pleural effusion | 903 | 1.93 (1.73-2.16) | 29.37% |
| I48→K59 | Atrial fibrillation and flutter | Other functional intestinal disorders | 853 | 1.84 (1.64-2.06) | 12.70% |
| I48→N17 | Atrial fibrillation and flutter | Acute renal failure | 1494 | 2.07 (1.89-2.26) | 24.25% |
| I48→N18 | Atrial fibrillation and flutter | Chronic kidney disease | 1197 | 1.97 (1.79-2.17) | 18.25% |
| I48→N39 | Atrial fibrillation and flutter | Disorders of urinary system, possibly infection | 1003 | 1.94 (1.74-2.15) | 14.78% |
| I48→S00 | Atrial fibrillation and flutter | Injuries due to external causes | 1092 | 2.12 (1.91-2.36) | 15.33% |
| I48→T80 | Atrial fibrillation and flutter | Complications due to medical treatment | 1011 | 2.13 (1.91-2.38) | 15.40% |
| I48→W00 | Atrial fibrillation and flutter | Falls | 924 | 1.94 (1.73-2.16) | 15.29% |
| I48→Y40 | Atrial fibrillation and flutter | External causes of morbidity related to medical treatment | 1339 | 2.43 (2.20-2.68) | 22.92% |
| I49→E87 | Other cardiac arrhythmias | Other disorders of fluid, electrolyte and acid-base balance | 838 | 3.05 (2.66-3.49) | 23.19% |
| I49→I50 | Other cardiac arrhythmias | Heart failure | 948 | 3.59 (3.13-4.12) | 26.90% |
| I49→I51 | Other cardiac arrhythmias | Complications and ill-defined descriptions of heart disease | 920 | 3.13 (2.74-3.57) | 31.11% |
| I49→J12 | Other cardiac arrhythmias | Pneumonia | 869 | 3.37 (2.93-3.87) | 21.33% |
| I49→N17 | Other cardiac arrhythmias | Acute renal failure | 964 | 3.29 (2.89-3.75) | 20.52% |
| I50→E87 | Heart failure | Other disorders of fluid, electrolyte and acid-base balance | 998 | 4.52 (3.90-5.22) | 24.94% |
| I50→I51 | Heart failure | Complications and ill-defined descriptions of heart disease | 1071 | 4.12 (3.60-4.72) | 22.80% |
| I50→N17 | Heart failure | Acute renal failure | 1180 | 4.80 (4.18-5.50) | 22.68% |
| I51→N17 | Complications and ill-defined descriptions of heart disease | Acute renal failure | 852 | 4.73 (4.03-5.56) | 17.50% |
| J12→A41 | Pneumonia | Sepsis | 924 | 3.07 (2.70-3.50) | 32.63% |
| J20→J12 | Other acute Lower respiratory infections | Pneumonia | 1011 | 5.71 (4.87-6.70) | 3.37% |
| J44→E87 | Chronic obstructive pulmonary disease | Other disorders of fluid, electrolyte and acid-base balance | 903 | 3.30 (2.88-3.77) | 14.03% |
| J44→J12 | Chronic obstructive pulmonary disease | Pneumonia | 1160 | 3.83 (3.37-4.34) | 19.42% |
| J44→N17 | Chronic obstructive pulmonary disease | Acute renal failure | 997 | 3.33 (2.93-3.79) | 14.04% |
| J44→Y40 | Chronic obstructive pulmonary disease | External causes of morbidity related to medical treatment | 860 | 3.71 (3.21-4.29) | 11.11% |
| J45→D10 | Asthma | Benign tumors | 993 | 3.39 (2.98-3.86) | 19.87% |
| J45→E66 | Asthma | Obesity | 1485 | 3.49 (3.13-3.88) | 29.22% |
| J45→E78 | Asthma | Disorders of lipoprotein metabolism and other lipidaemias | 1294 | 3.55 (3.16-3.99) | 30.00% |
| J45→H25 | Asthma | Disorders of the lens | 1076 | 3.23 (2.86-3.65) | 21.63% |
| J45→J12 | Asthma | Pneumonia | 949 | 2.84 (2.51-3.22) | 16.31% |
| J45→J44 | Asthma | Chronic obstructive pulmonary disease | 1103 | 3.04 (2.70-3.42) | 5.38% |
| J45→K21 | Asthma | Gastro-oesophageal reflux disease | 1144 | 2.98 (2.65-3.34) | 24.45% |
| J45→K57 | Asthma | Diverticular disease of intestine | 1243 | 3.24 (2.89-3.63) | 17.41% |
| J45→M10 | Asthma | Other inflammatory arthritis | 1115 | 2.85 (2.54-3.20) | 25.02% |
| J45→M15 | Asthma | Osteoarthritis | 1494 | 3.70 (3.31-4.13) | 31.83% |
| J45→N17 | Asthma | Acute renal failure | 864 | 2.93 (2.57-3.34) | 11.50% |
| J45→S00 | Asthma | Injuries due to external causes | 866 | 2.99 (2.61-3.41) | 21.45% |
| J45→T80 | Asthma | Complications due to medical treatment | 854 | 2.73 (2.40-3.11) | 11.84% |
| J45→Y40 | Asthma | External causes of morbidity related to medical treatment | 1176 | 2.87 (2.56-3.21) | 10.83% |
| K21→A09 | Gastro-oesophageal reflux disease | Infectious gastroenteritis and colitis | 842 | 2.12 (1.88-2.39) | 8.21% |
| K21→D51 | Gastro-oesophageal reflux disease | Other anaemias | 967 | 2.23 (1.99-2.50) | 17.06% |
| K21→E87 | Gastro-oesophageal reflux disease | Other disorders of fluid, electrolyte and acid-base balance | 943 | 1.95 (1.75-2.17) | 10.38% |
| K21→J12 | Gastro-oesophageal reflux disease | Pneumonia | 1055 | 2.08 (1.87-2.31) | 10.54% |
| K21→K59 | Gastro-oesophageal reflux disease | Other functional intestinal disorders | 976 | 2.07 (1.85-2.31) | 12.24% |
| K21→N17 | Gastro-oesophageal reflux disease | Acute renal failure | 993 | 2.14 (1.92-2.39) | 11.07% |
| K21→N18 | Gastro-oesophageal reflux disease | Chronic kidney disease | 839 | 1.89 (1.68-2.12) | 16.62% |
| K21→T80 | Gastro-oesophageal reflux disease | Complications due to medical treatment | 1058 | 2.41 (2.16-2.69) | 11.19% |
| K21→Y40 | Gastro-oesophageal reflux disease | External causes of morbidity related to medical treatment | 1380 | 2.48 (2.25-2.74) | 11.22% |
| K29→A09 | Gastritis and duodenitis | Infectious gastroenteritis and colitis | 896 | 2.30 (2.04-2.59) | 6.45% |
| K29→D51 | Gastritis and duodenitis | Other anaemias | 1027 | 2.57 (2.29-2.89) | 25.03% |
| K29→E66 | Gastritis and duodenitis | Obesity | 1445 | 2.70 (2.45-2.98) | 6.82% |
| K29→E87 | Gastritis and duodenitis | Other disorders of fluid, electrolyte and acid-base balance | 983 | 2.31 (2.06-2.59) | 4.40% |
| K29→H25 | Gastritis and duodenitis | Disorders of the lens | 1143 | 2.34 (2.10-2.60) | 0.30% |
| K29→J12 | Gastritis and duodenitis | Pneumonia | 1086 | 2.42 (2.17-2.70) | 2.83% |
| K29→K21 | Gastritis and duodenitis | Gastro-oesophageal reflux disease | 1499 | 3.25 (2.93-3.61) | 41.63% |
| K29→K59 | Gastritis and duodenitis | Other functional intestinal disorders | 908 | 2.32 (2.06-2.61) | 5.53% |
| K29→K63 | Gastritis and duodenitis | Other diseases of intestine | 980 | 2.51 (2.23-2.83) | 13.45% |
| K29→N17 | Gastritis and duodenitis | Acute renal failure | 1123 | 2.46 (2.21-2.75) | 3.62% |
| K29→N18 | Gastritis and duodenitis | Chronic kidney disease | 962 | 2.30 (2.05-2.58) | 8.87% |
| K29→Y40 | Gastritis and duodenitis | External causes of morbidity related to medical treatment | 1208 | 2.40 (2.16-2.66) | 5.24% |
| K44→D51 | Diaphragmatic hernia | Other anaemias | 966 | 2.32 (2.07-2.60) | 24.74% |
| K44→E66 | Diaphragmatic hernia | Obesity | 1407 | 2.63 (2.38-2.91) | 14.14% |
| K44→E87 | Diaphragmatic hernia | Other disorders of fluid, electrolyte and acid-base balance | 868 | 2.03 (1.81-2.28) | 4.94% |
| K44→H25 | Diaphragmatic hernia | Disorders of the lens | 1078 | 2.39 (2.14-2.67) | 3.47% |
| K44→I48 | Diaphragmatic hernia | Atrial fibrillation and flutter | 824 | 2.42 (2.13-2.74) | 8.33% |
| K44→J12 | Diaphragmatic hernia | Pneumonia | 953 | 2.36 (2.10-2.66) | 5.87% |
| K44→K21 | Diaphragmatic hernia | Gastro-oesophageal reflux disease | 1600 | 3.33 (3.01-3.69) | 48.06% |
| K44→K57 | Diaphragmatic hernia | Diverticular disease of intestine | 1563 | 2.92 (2.65-3.22) | 15.76% |
| K44→K59 | Diaphragmatic hernia | Other functional intestinal disorders | 842 | 2.13 (1.89-2.40) | 7.18% |
| K44→K63 | Diaphragmatic hernia | Other diseases of intestine | 871 | 2.22 (1.97-2.50) | 12.11% |
| K44→N17 | Diaphragmatic hernia | Acute renal failure | 954 | 2.18 (1.95-2.44) | 4.62% |
| K44→T80 | Diaphragmatic hernia | Complications due to medical treatment | 964 | 2.50 (2.22-2.81) | 3.96% |
| K44→Y40 | Diaphragmatic hernia | External causes of morbidity related to medical treatment | 1199 | 2.83 (2.54-3.17) | 6.33% |
| K57→A09 | Diverticular disease of intestine | Infectious gastroenteritis and colitis | 1019 | 1.52 (1.38-1.68) | 24.45% |
| K57→A41 | Diverticular disease of intestine | Sepsis | 867 | 1.38 (1.25-1.53) | 7.10% |
| K57→B96 | Diverticular disease of intestine | Bacterial infectious agents | 879 | 1.53 (1.38-1.70) | 7.98% |
| K57→C77 | Diverticular disease of intestine | Metastatic cancer | 823 | 1.40 (1.26-1.56) | 7.59% |
| K57→D50 | Diverticular disease of intestine | Iron deficiency anaemia | 886 | 1.65 (1.48-1.84) | 32.67% |
| K57→D51 | Diverticular disease of intestine | Other anaemias | 1196 | 1.57 (1.43-1.71) | 19.81% |
| K57→E66 | Diverticular disease of intestine | Obesity | 2001 | 1.90 (1.76-2.04) | 7.81% |
| K57→E87 | Diverticular disease of intestine | Other disorders of fluid, electrolyte and acid-base balance | 1185 | 1.55 (1.41-1.69) | 6.44% |
| K57→F40 | Diverticular disease of intestine | Anxiety | 831 | 1.59 (1.42-1.77) | 9.23% |
| K57→H25 | Diverticular disease of intestine | Disorders of the lens | 1584 | 1.70 (1.57-1.84) | 1.94% |
| K57→I49 | Diverticular disease of intestine | Other cardiac arrhythmias | 1021 | 1.47 (1.33-1.62) | 5.43% |
| K57→I50 | Diverticular disease of intestine | Heart failure | 838 | 1.49 (1.34-1.66) | 6.71% |
| K57→I51 | Diverticular disease of intestine | Complications and ill-defined descriptions of heart disease | 827 | 1.43 (1.28-1.59) | 6.00% |
| K57→I95 | Diverticular disease of intestine | Hypotension | 835 | 1.50 (1.35-1.67) | 5.99% |
| K57→J12 | Diverticular disease of intestine | Pneumonia | 1349 | 1.52 (1.40-1.66) | 5.88% |
| K57→K59 | Diverticular disease of intestine | Other functional intestinal disorders | 1085 | 1.56 (1.42-1.71) | 23.80% |
| K57→K63 | Diverticular disease of intestine | Other diseases of intestine | 1297 | 1.82 (1.66-2.00) | 54.91% |
| K57→K64 | Diverticular disease of intestine | Haemorrhoids and perianal venous thrombosis | 1183 | 1.67 (1.52-1.84) | 52.90% |
| K57→M10 | Diverticular disease of intestine | Other inflammatory arthritis | 1641 | 1.76 (1.63-1.91) | 12.08% |
| K57→M54 | Diverticular disease of intestine | Dorsalgia | 932 | 1.48 (1.34-1.64) | 7.26% |
| K57→N17 | Diverticular disease of intestine | Acute renal failure | 1391 | 1.54 (1.42-1.67) | 7.33% |
| K57→N18 | Diverticular disease of intestine | Chronic kidney disease | 1195 | 1.47 (1.34-1.60) | 11.86% |
| K57→N39 | Diverticular disease of intestine | Disorders of urinary system, possibly infection | 1102 | 1.57 (1.43-1.72) | 5.12% |
| K57→T80 | Diverticular disease of intestine | Complications due to medical treatment | 1248 | 1.65 (1.51-1.81) | 4.59% |
| K57→W00 | Diverticular disease of intestine | Falls | 993 | 1.52 (1.38-1.68) | 3.80% |
| K57→Y40 | Diverticular disease of intestine | External causes of morbidity related to medical treatment | 1675 | 1.73 (1.60-1.87) | 4.57% |
| K62→E66 | Other diseases of anus and rectum | Obesity | 943 | 4.96 (4.25-5.80) | 6.82% |
| K63→H25 | Other diseases of intestine | Disorders of the lens | 910 | 3.07 (2.70-3.51) | 0.62% |
| K63→N17 | Other diseases of intestine | Acute renal failure | 848 | 2.58 (2.27-2.93) | 4.31% |
| K63→Y40 | Other diseases of intestine | External causes of morbidity related to medical treatment | 1033 | 3.20 (2.82-3.62) | 4.56% |
| K80→Y40 | Cholelithiasis | External causes of morbidity related to medical treatment | 826 | 4.64 (3.95-5.46) | 10.32% |
| M10→A09 | Other inflammatory arthritis | Infectious gastroenteritis and colitis | 912 | 1.97 (1.76-2.20) | 10.31% |
| M10→A41 | Other inflammatory arthritis | Sepsis | 817 | 1.92 (1.71-2.16) | 10.03% |
| M10→B96 | Other inflammatory arthritis | Bacterial infectious agents | 911 | 1.91 (1.71-2.13) | 8.21% |
| M10→D51 | Other inflammatory arthritis | Other anaemias | 1129 | 2.01 (1.82-2.23) | 12.47% |
| M10→E87 | Other inflammatory arthritis | Other disorders of fluid, electrolyte and acid-base balance | 1153 | 2.00 (1.81-2.21) | 9.98% |
| M10→I49 | Other inflammatory arthritis | Other cardiac arrhythmias | 936 | 2.07 (1.85-2.32) | 16.99% |
| M10→I50 | Other inflammatory arthritis | Heart failure | 910 | 1.90 (1.70-2.12) | 11.28% |
| M10→I51 | Other inflammatory arthritis | Complications and ill-defined descriptions of heart disease | 885 | 1.81 (1.62-2.03) | 12.37% |
| M10→I95 | Other inflammatory arthritis | Hypotension | 858 | 1.87 (1.67-2.09) | 10.11% |
| M10→J12 | Other inflammatory arthritis | Pneumonia | 1271 | 2.02 (1.83-2.22) | 11.79% |
| M10→K59 | Other inflammatory arthritis | Other functional intestinal disorders | 1053 | 1.93 (1.74-2.14) | 12.38% |
| M10→N17 | Other inflammatory arthritis | Acute renal failure | 1390 | 2.10 (1.92-2.31) | 11.19% |
| M10→N18 | Other inflammatory arthritis | Chronic kidney disease | 1144 | 2.01 (1.82-2.22) | 19.53% |
| M10→N39 | Other inflammatory arthritis | Disorders of urinary system, possibly infection | 945 | 1.94 (1.74-2.16) | 11.26% |
| M10→T80 | Other inflammatory arthritis | Complications due to medical treatment | 1193 | 2.22 (2.01-2.46) | 10.32% |
| M10→W00 | Other inflammatory arthritis | Falls | 935 | 1.89 (1.70-2.11) | 13.73% |
| M10→Y40 | Other inflammatory arthritis | External causes of morbidity related to medical treatment | 1522 | 2.29 (2.09-2.50) | 10.86% |
| M15→D10 | Osteoarthritis | Benign tumors | 2038 | 1.15 (1.08-1.23) | 9.31% |
| M15→E66 | Osteoarthritis | Obesity | 3648 | 1.37 (1.30-1.44) | 36.78% |
| M15→K57 | Osteoarthritis | Diverticular disease of intestine | 2605 | 1.14 (1.07-1.20) | 7.13% |
| M15→M10 | Osteoarthritis | Other inflammatory arthritis | 3208 | 1.25 (1.18-1.31) | 16.53% |
| M15→M20 | Osteoarthritis | Other joint disorders | 2192 | 1.33 (1.24-1.41) | 48.85% |
| M15→Y40 | Osteoarthritis | External causes of morbidity related to medical treatment | 2887 | 1.20 (1.13-1.26) | 14.64% |
| M20→D10 | Other joint disorders | Benign tumors | 1027 | 2.50 (2.23-2.81) | 2.52% |
| M20→D51 | Other joint disorders | Other anaemias | 821 | 2.26 (1.99-2.55) | 6.62% |
| M20→E66 | Other joint disorders | Obesity | 1728 | 3.05 (2.78-3.36) | 28.93% |
| M20→H25 | Other joint disorders | Disorders of the lens | 1079 | 2.69 (2.40-3.02) | 1.30% |
| M20→I25 | Other joint disorders | Chronic ischaemic heart disease | 893 | 2.59 (2.29-2.93) | 8.34% |
| M20→J12 | Other joint disorders | Pneumonia | 901 | 2.55 (2.25-2.88) | 6.35% |
| M20→K21 | Other joint disorders | Gastro-oesophageal reflux disease | 1215 | 2.47 (2.23-2.75) | 11.99% |
| M20→K57 | Other joint disorders | Diverticular disease of intestine | 1297 | 2.51 (2.27-2.78) | 2.21% |
| M20→K59 | Other joint disorders | Other functional intestinal disorders | 857 | 2.62 (2.31-2.98) | 8.81% |
| M20→M10 | Other joint disorders | Other inflammatory arthritis | 1433 | 2.64 (2.39-2.91) | 18.44% |
| M20→M79 | Other joint disorders | Other soft tissue disorders, not elsewhere classified | 820 | 2.44 (2.15-2.77) | 17.27% |
| M20→N17 | Other joint disorders | Acute renal failure | 889 | 2.48 (2.19-2.80) | 7.94% |
| M20→T80 | Other joint disorders | Complications due to medical treatment | 1166 | 2.66 (2.39-2.97) | 6.53% |
| M20→Y40 | Other joint disorders | External causes of morbidity related to medical treatment | 1279 | 2.87 (2.57-3.19) | 11.53% |
| M54→M47 | Dorsalgia | Spondylosis | 861 | 4.26 (3.66-4.97) | 16.36% |
| N17→A41 | Acute renal failure | Sepsis | 1073 | 3.01 (2.67-3.39) | 39.97% |
| N17→E87 | Acute renal failure | Other disorders of fluid, electrolyte and acid-base balance | 1281 | 3.63 (3.23-4.08) | 43.13% |
| N17→I95 | Acute renal failure | Hypotension | 845 | 3.12 (2.72-3.58) | 33.39% |
| N18→E87 | Chronic kidney disease | Other disorders of fluid, electrolyte and acid-base balance | 1159 | 3.08 (2.74-3.46) | 18.47% |
| N18→J12 | Chronic kidney disease | Pneumonia | 1093 | 2.89 (2.57-3.25) | 16.50% |
| N39→A41 | Disorders of urinary system, possibly infection | Sepsis | 906 | 2.98 (2.62-3.39) | 34.07% |
| N39→B96 | Disorders of urinary system, possibly infection | Bacterial infectious agents | 853 | 3.51 (3.04-4.05) | 62.85% |
| N39→E87 | Disorders of urinary system, possibly infection | Other disorders of fluid, electrolyte and acid-base balance | 1012 | 2.72 (2.42-3.06) | 19.88% |
| N39→J12 | Disorders of urinary system, possibly infection | Pneumonia | 1075 | 3.05 (2.70-3.43) | 14.28% |
| N39→K59 | Disorders of urinary system, possibly infection | Other functional intestinal disorders | 948 | 2.69 (2.38-3.04) | 19.73% |
| S00→B96 | Injuries due to external causes | Bacterial infectious agents | 951 | 2.05 (1.83-2.29) | 11.69% |
| S00→E87 | Injuries due to external causes | Other disorders of fluid, electrolyte and acid-base balance | 1129 | 2.04 (1.84-2.26) | 15.04% |
| S00→I95 | Injuries due to external causes | Hypotension | 818 | 2.04 (1.81-2.30) | 22.02% |
| S00→J12 | Injuries due to external causes | Pneumonia | 1289 | 2.14 (1.94-2.35) | 12.65% |
| S00→K59 | Injuries due to external causes | Other functional intestinal disorders | 999 | 1.90 (1.71-2.11) | 13.55% |
| S00→N17 | Injuries due to external causes | Acute renal failure | 1210 | 2.15 (1.95-2.38) | 14.98% |
| S00→N39 | Injuries due to external causes | Disorders of urinary system, possibly infection | 1072 | 2.06 (1.86-2.29) | 11.70% |
| S00→T80 | Injuries due to external causes | Complications due to medical treatment | 1160 | 2.24 (2.02-2.49) | 7.06% |
| T80→A41 | Complications due to medical treatment | Sepsis | 852 | 2.66 (2.34-3.03) | 18.54% |
| T80→B96 | Complications due to medical treatment | Bacterial infectious agents | 981 | 3.48 (3.05-3.97) | 39.19% |
| T80→E87 | Complications due to medical treatment | Other disorders of fluid, electrolyte and acid-base balance | 989 | 2.87 (2.54-3.25) | 17.74% |
| T80→J12 | Complications due to medical treatment | Pneumonia | 1114 | 2.98 (2.65-3.35) | 15.42% |
| T80→K59 | Complications due to medical treatment | Other functional intestinal disorders | 942 | 2.62 (2.32-2.95) | 11.73% |
| T80→N17 | Complications due to medical treatment | Acute renal failure | 1153 | 2.90 (2.59-3.26) | 15.56% |
| W00→E87 | Falls | Other disorders of fluid, electrolyte and acid-base balance | 912 | 2.65 (2.34-3.00) | 14.13% |
| W00→J12 | Falls | Pneumonia | 1038 | 2.88 (2.56-3.25) | 12.71% |
| W00→K59 | Falls | Other functional intestinal disorders | 848 | 2.55 (2.25-2.90) | 13.63% |
| W00→N17 | Falls | Acute renal failure | 973 | 2.81 (2.49-3.18) | 14.81% |
| Y40→A09 | External causes of morbidity related to medical treatment | Infectious gastroenteritis and colitis | 1060 | 1.89 (1.71-2.10) | 13.17% |
| Y40→A41 | External causes of morbidity related to medical treatment | Sepsis | 1205 | 2.01 (1.82-2.21) | 24.03% |
| Y40→B96 | External causes of morbidity related to medical treatment | Bacterial infectious agents | 1283 | 2.17 (1.97-2.39) | 27.01% |
| Y40→E87 | External causes of morbidity related to medical treatment | Other disorders of fluid, electrolyte and acid-base balance | 1408 | 2.28 (2.07-2.50) | 25.56% |
| Y40→I95 | External causes of morbidity related to medical treatment | Hypotension | 1033 | 2.05 (1.84-2.28) | 29.19% |
| Y40→J12 | External causes of morbidity related to medical treatment | Pneumonia | 1564 | 2.12 (1.94-2.31) | 18.35% |
| Y40→J20 | External causes of morbidity related to medical treatment | Other acute Lower respiratory infections | 871 | 1.95 (1.74-2.18) | 14.34% |
| Y40→J90 | External causes of morbidity related to medical treatment | Pleural effusion | 901 | 1.84 (1.64-2.05) | 22.04% |
| Y40→K59 | External causes of morbidity related to medical treatment | Other functional intestinal disorders | 1271 | 2.02 (1.83-2.22) | 19.34% |
| Y40→N17 | External causes of morbidity related to medical treatment | Acute renal failure | 1541 | 2.25 (2.06-2.46) | 25.04% |

^*^Combined ICD-10 codes derived from the original ICD-10 code and are displayed in the Additional file 1.

^#^Number of the MAFLD participants experienced the corresponding temporal disease trajectories.

**^&^**Number of MAFLD individuals having the same D1 and D2 diagnosis date divided by the MAFLD individuals diagnosed with both D1 and D2. A high value indicates a strong correlation between the two diseases and may exist a reverse causal relationship.

OR, odds ratio; CI, confidence interval. MAFLD, Metabolic dysfunction-associated fatty liver disease.

Additional file 1: Table S3. Basic characteristics of the dead and surviving participants with MAFLD after propensity score matching.

| **Characteristics** | **Total**  **(N=81,938)** | **Survival**  **(N=67,402)** | **Dead**  **(N=14,536)** |
| --- | --- | --- | --- |
|  |  |  |  |
| Male, n (%) | 55106 (67.3) | 45294 (67.2) | 9812 (67.5) |
| Age (years) | 63 (58-66) | 63 (58-66) | 63 (58-66) |
| Deprivation Index | -1.67 (-3.36-1.36) | -1.70 (-3.38-1.29) | -1.65 (-3.34-1.38) |
| Alcohol intake (g/day) | 11.43 (0-27.14) | 11.43 (0-27.14) | 10 (0-28.57) |
| BMI (kg/m2) | 30.66 (28.47-33.59) | 30.6 (28.45-33.43) | 31.03 (28.6-34.35) |
| Follow-up time (years) | 12.72 (11.87-13.54) | 13 (12.31-13.69) | 8.16 (5.12-10.47) |

Data were expressed as n (%) and median (25^th^ - 75^th^). A propensity score matching was conducted according to age, sex, and Townsend deprivation index (assigned by the postcode of participants location, which reflect the level of social deprivation in which the participant lives) for subjects with and without MAFLD. The recording of alcohol intake is based on recollection and estimation of the average total amount of red wine, spirits, beer, and fruit wine consumed each week or month. BMI, body mass index; MAFLD, Metabolic dysfunction-associated fatty liver disease.

Additional file 1: Table S4. PheWAS using Cox regression was conducted to investigate the relationship between medical conditions and 7 causes of death in MAFLD individuals.

| **Medical conditions** | **Code*** | **No.#** | **HR (95% CI)** | **Causes of death** |
| --- | --- | --- | --- | --- |
| Infectious gastroenteritis and colitis | A09 | 652 | 2.22 (2.04-2.41) | CVDD |
| Sepsis | A41 | 987 | 4.19 (3.91-4.49) | CVDD |
| Mycoses | B49 | 348 | 3.27 (2.93-3.64) | CVDD |
| Bacterial infectious agents | B96 | 994 | 3.21 (3.00-3.44) | CVDD |
| Colon cancer | C18 | 130 | 1.43 (1.20-1.70) | CVDD |
| Metastatic cancer | C77 | 768 | 3.15 (2.92-3.40) | CVDD |
| Iron deficiency anaemia | D50 | 637 | 2.16 (1.99-2.35) | CVDD |
| Other anaemias | D51 | 1127 | 3.10 (2.90-3.31) | CVDD |
| Diabetes | E10 | 1275 | 1.94 (1.82-2.07) | CVDD |
| Malnutrition | E40 | 296 | 2.00 (1.78-2.25) | CVDD |
| Obesity | E66 | 962 | 1.22 (1.14-1.31) | CVDD |
| Disorders of lipoprotein metabolism and other lipidaemias | E78 | 1430 | 1.46 (1.37-1.55) | CVDD |
| Disorders of mineral metabolism | E83 | 351 | 2.78 (2.50-3.10) | CVDD |
| Volume depletion | E86 | 621 | 3.78 (3.48-4.11) | CVDD |
| Other disorders of fluid, electrolyte and acid-base balance | E87 | 1571 | 5.27 (4.98-5.59) | CVDD |
| Delirium due to known physiological condition | F05 | 508 | 3.68 (3.36-4.03) | CVDD |
| Alcohol abuse | F10 | 313 | 2.38 (2.13-2.67) | CVDD |
| Tobacco abuse | F17 | 718 | 2.30 (2.13-2.49) | CVDD |
| Depression | F32 | 558 | 1.69 (1.55-1.84) | CVDD |
| Anxiety | F40 | 367 | 1.37 (1.24-1.53) | CVDD |
| Epilepsia | G40 | 183 | 2.83 (2.44-3.27) | CVDD |
| Sleep disorder | G47 | 348 | 1.83 (1.64-2.04) | CVDD |
| Polyneuropathies | G60 | 286 | 2.85 (2.53-3.21) | CVDD |
| Cerebral palsy and other paralytic syndromes | G80 | 325 | 4.19 (3.75-4.68) | CVDD |
| Disorders of choroid and retina | H30 | 391 | 1.41 (1.27-1.56) | CVDD |
| Visual disturbances and blindness | H53 | 249 | 1.82 (1.61-2.07) | CVDD |
| Chronic rheumatic heart disease | I05 | 609 | 3.95 (3.63-4.29) | CVDD |
| Primary hypertension | I10 | 2363 | 1.88 (1.77-2.00) | CVDD |
| Angina pectoris | I20 | 805 | 2.24 (2.08-2.41) | CVDD |
| Acute myocardial infarction | I21 | 740 | 3.74 (3.46-4.04) | CVDD |
| Other ischemic heart disease | I22 | 271 | 3.45 (3.05-3.89) | CVDD |
| Chronic ischaemic heart disease | I25 | 1380 | 2.78 (2.61-2.96) | CVDD |
| Embolism and thrombosis | I26 | 744 | 3.51 (3.25-3.80) | CVDD |
| Non-rheumatic valve disorders | I34 | 724 | 3.21 (2.97-3.47) | CVDD |
| Atrial fibrillation and flutter | I48 | 1583 | 3.27 (3.09-3.47) | CVDD |
| Other cardiac arrhythmias | I49 | 1093 | 2.83 (2.65-3.02) | CVDD |
| Heart failure | I50 | 1591 | 6.21 (5.86-6.58) | CVDD |
| Complications and ill-defined descriptions of heart disease | I51 | 1036 | 3.71 (3.47-3.97) | CVDD |
| Stroke | I60 | 944 | 5.33 (4.97-5.72) | CVDD |
| Other cerebrovascular diseases | I65 | 622 | 3.20 (2.95-3.48) | CVDD |
| Aneurysm and dissection | I71 | 270 | 2.85 (2.52-3.22) | CVDD |
| Peripheral vascular disease | I73 | 515 | 4.16 (3.80-4.55) | CVDD |
| Varicose veins of lower extremities | I83 | 160 | 1.48 (1.27-1.73) | CVDD |
| Hypotension | I95 | 953 | 3.82 (3.57-4.10) | CVDD |
| Pneumonia | J12 | 1807 | 5.01 (4.74-5.30) | CVDD |
| Other acute Lower respiratory infections | J20 | 768 | 3.31 (3.07-3.57) | CVDD |
| Chronic obstructive pulmonary disease | J44 | 968 | 2.98 (2.78-3.20) | CVDD |
| Pleural effusion | J90 | 986 | 4.67 (4.36-5.00) | CVDD |
| Other diseases of the respiratory system | J95 | 558 | 2.73 (2.50-2.97) | CVDD |
| Respiratory failure | J96 | 848 | 6.48 (6.03-6.97) | CVDD |
| Oesophagitis | K20 | 210 | 1.34 (1.16-1.53) | CVDD |
| Other diseases of oesophagus | K22 | 317 | 1.31 (1.17-1.47) | CVDD |
| Gastric ulcer | K25 | 184 | 1.79 (1.55-2.08) | CVDD |
| Gastritis and duodenitis | K29 | 734 | 1.35 (1.25-1.46) | CVDD |
| Disease of stomach and duodenum | K31 | 328 | 1.39 (1.25-1.56) | CVDD |
| Noninfective gastroenteritis and colitis | K52 | 367 | 1.92 (1.72-2.13) | CVDD |
| Other functional intestinal disorders | K59 | 851 | 2.35 (2.18-2.52) | CVDD |
| Other diseases of liver | K76 | 408 | 2.07 (1.87-2.29) | CVDD |
| Cholelithiasis | K80 | 389 | 1.32 (1.20-1.47) | CVDD |
| Disease of gallbladder and biliary tract | K82 | 189 | 1.67 (1.44-1.92) | CVDD |
| Other disease of digestive system | K92 | 593 | 2.21 (2.03-2.41) | CVDD |
| Skin and subcutaneous infections | L01 | 681 | 2.72 (2.51-2.95) | CVDD |
| Dermatitis | L20 | 213 | 1.86 (1.62-2.13) | CVDD |
| Pressure ulcer | L89 | 492 | 5.96 (5.44-6.54) | CVDD |
| Autoimmune arthritis | M05 | 180 | 1.45 (1.25-1.68) | CVDD |
| Other inflammatory arthritis | M10 | 868 | 1.28 (1.19-1.38) | CVDD |
| Systemic connective tissue disorders | M30 | 146 | 1.40 (1.19-1.65) | CVDD |
| Spondylopathy | M48 | 243 | 1.32 (1.16-1.50) | CVDD |
| Dorsalgia | M54 | 485 | 1.37 (1.24-1.50) | CVDD |
| Other soft tissue disorders, not elsewhere classified | M79 | 509 | 1.67 (1.53-1.83) | CVDD |
| Obstructive and reflux uropathy | N13 | 170 | 1.86 (1.59-2.16) | CVDD |
| Acute renal failure | N17 | 1870 | 5.30 (5.02-5.60) | CVDD |
| Chronic kidney disease | N18 | 1284 | 3.24 (3.05-3.45) | CVDD |
| Other disorders of kidney and ureter | N29 | 256 | 1.95 (1.72-2.21) | CVDD |
| Disorders of urinary system, possibly infection | N39 | 1077 | 2.77 (2.59-2.96) | CVDD |
| Injuries due to external causes | S00 | 1096 | 1.96 (1.83-2.09) | CVDD |
| Complications due to medical treatment | T80 | 894 | 1.98 (1.85-2.13) | CVDD |
| Falls | W00 | 938 | 2.21 (2.06-2.37) | CVDD |
| Other external causes of accidental injury | W20 | 406 | 2.00 (1.81-2.21) | CVDD |
| External causes of morbidity related to medical treatment | Y40 | 1255 | 2.22 (2.08-2.36) | CVDD |
| External causes of morbidity related to other conditions | Y95 | 589 | 5.34 (4.90-5.81) | CVDD |
| Infectious gastroenteritis and colitis | A09 | 269 | 4.32 (3.78-4.93) | DSSD |
| Sepsis | A41 | 403 | 8.68 (7.73-9.75) | DSSD |
| Mycoses | B49 | 122 | 5.06 (4.20-6.10) | DSSD |
| Bacterial infectious agents | B96 | 305 | 4.61 (4.06-5.24) | DSSD |
| Colon cancer | C18 | 67 | 3.24 (2.54-4.15) | DSSD |
| Metastatic cancer | C77 | 245 | 4.59 (3.99-5.27) | DSSD |
| Benign tumors | D10 | 225 | 1.37 (1.19-1.58) | DSSD |
| Iron deficiency anaemia | D50 | 208 | 3.20 (2.76-3.70) | DSSD |
| Other anaemias | D51 | 401 | 5.51 (4.90-6.19) | DSSD |
| Diabetes | E10 | 327 | 2.06 (1.82-2.34) | DSSD |
| Malnutrition | E40 | 113 | 3.38 (2.79-4.10) | DSSD |
| Obesity | E66 | 253 | 1.39 (1.21-1.60) | DSSD |
| Disorders of mineral metabolism | E83 | 132 | 4.65 (3.89-5.57) | DSSD |
| Volume depletion | E86 | 232 | 6.52 (5.66-7.51) | DSSD |
| Other disorders of fluid, electrolyte and acid-base balance | E87 | 551 | 9.61 (8.62-10.70) | DSSD |
| Delirium due to known physiological condition | F05 | 139 | 4.36 (3.66-5.19) | DSSD |
| Tobacco abuse | F17 | 211 | 3.00 (2.59-3.47) | DSSD |
| Depression | F32 | 155 | 2.05 (1.73-2.42) | DSSD |
| Anxiety | F40 | 123 | 2.01 (1.67-2.42) | DSSD |
| Epilepsia | G40 | 32 | 2.09 (1.47-2.96) | DSSD |
| Sleep disorder | G47 | 78 | 1.74 (1.38-2.19) | DSSD |
| Polyneuropathies | G60 | 71 | 3.03 (2.39-3.85) | DSSD |
| Cerebral palsy and other paralytic syndromes | G80 | 39 | 2.07 (1.50-2.84) | DSSD |
| Chronic rheumatic heart disease | I05 | 84 | 2.21 (1.77-2.75) | DSSD |
| Primary hypertension | I10 | 562 | 1.68 (1.49-1.90) | DSSD |
| Angina pectoris | I20 | 136 | 1.41 (1.18-1.69) | DSSD |
| Acute myocardial infarction | I21 | 90 | 1.76 (1.42-2.18) | DSSD |
| Chronic ischaemic heart disease | I25 | 215 | 1.43 (1.24-1.66) | DSSD |
| Embolism and thrombosis | I26 | 186 | 3.76 (3.22-4.39) | DSSD |
| Non-rheumatic valve disorders | I34 | 108 | 1.91 (1.57-2.32) | DSSD |
| Atrial fibrillation and flutter | I48 | 301 | 2.34 (2.06-2.66) | DSSD |
| Other cardiac arrhythmias | I49 | 184 | 1.86 (1.59-2.17) | DSSD |
| Heart failure | I50 | 254 | 3.59 (3.13-4.11) | DSSD |
| Complications and ill-defined descriptions of heart disease | I51 | 156 | 2.19 (1.85-2.59) | DSSD |
| Stroke | I60 | 91 | 1.95 (1.58-2.42) | DSSD |
| Other cerebrovascular diseases | I65 | 106 | 2.23 (1.83-2.72) | DSSD |
| Aneurysm and dissection | I71 | 55 | 2.45 (1.87-3.21) | DSSD |
| Peripheral vascular disease | I73 | 83 | 2.74 (2.19-3.42) | DSSD |
| Hypotension | I95 | 299 | 5.50 (4.84-6.26) | DSSD |
| Pneumonia | J12 | 425 | 5.07 (4.52-5.68) | DSSD |
| Other acute Lower respiratory infections | J20 | 165 | 3.00 (2.55-3.53) | DSSD |
| Chronic obstructive pulmonary disease | J44 | 199 | 2.52 (2.17-2.92) | DSSD |
| Pleural effusion | J90 | 288 | 6.14 (5.39-7.00) | DSSD |
| Other diseases of the respiratory system | J95 | 164 | 3.51 (2.98-4.13) | DSSD |
| Respiratory failure | J96 | 212 | 6.98 (6.02-8.08) | DSSD |
| Oesophagitis | K20 | 92 | 2.58 (2.09-3.19) | DSSD |
| Gastro-oesophageal reflux disease | K21 | 214 | 1.55 (1.34-1.79) | DSSD |
| Other diseases of oesophagus | K22 | 154 | 2.91 (2.46-3.44) | DSSD |
| Gastric ulcer | K25 | 74 | 3.18 (2.51-4.01) | DSSD |
| Gastritis and duodenitis | K29 | 325 | 3.13 (2.76-3.55) | DSSD |
| Disease of stomach and duodenum | K31 | 243 | 5.09 (4.44-5.85) | DSSD |
| Umbilical hernia | K42 | 65 | 2.74 (2.14-3.51) | DSSD |
| Ventral hernia | K43 | 63 | 3.35 (2.60-4.31) | DSSD |
| Diaphragmatic hernia | K44 | 206 | 1.70 (1.47-1.98) | DSSD |
| Noninfective gastroenteritis and colitis | K52 | 129 | 3.04 (2.53-3.64) | DSSD |
| Other functional intestinal disorders | K59 | 256 | 3.18 (2.78-3.65) | DSSD |
| Other diseases of intestine | K63 | 215 | 2.06 (1.79-2.39) | DSSD |
| Other disorders of peritoneum | K66 | 65 | 2.54 (1.98-3.26) | DSSD |
| Cholelithiasis | K80 | 201 | 3.23 (2.78-3.76) | DSSD |
| Disease of gallbladder and biliary tract | K82 | 162 | 6.76 (5.74-7.97) | DSSD |
| Other disease of digestive system | K92 | 328 | 6.30 (5.56-7.13) | DSSD |
| Skin and subcutaneous infections | L01 | 199 | 3.51 (3.02-4.08) | DSSD |
| Dermatitis | L20 | 54 | 2.02 (1.54-2.65) | DSSD |
| Papulosquamous disorders | L40 | 34 | 1.74 (1.24-2.45) | DSSD |
| Pressure ulcer | L89 | 122 | 6.33 (5.26-7.63) | DSSD |
| Other soft tissue disorders, not elsewhere classified | M79 | 112 | 1.56 (1.29-1.90) | DSSD |
| Obstructive and reflux uropathy | N13 | 40 | 1.87 (1.36-2.56) | DSSD |
| Chronic kidney disease | N18 | 265 | 2.76 (2.42-3.16) | DSSD |
| Other disorders of kidney and ureter | N29 | 73 | 2.40 (1.90-3.04) | DSSD |
| Disorders of urinary system, possibly infection | N39 | 275 | 3.10 (2.71-3.54) | DSSD |
| Injuries due to external causes | S00 | 262 | 2.03 (1.77-2.32) | DSSD |
| Complications due to medical treatment | T80 | 271 | 2.71 (2.37-3.10) | DSSD |
| Falls | W00 | 229 | 2.36 (2.04-2.72) | DSSD |
| Other external causes of accidental injury | W20 | 98 | 2.09 (1.70-2.56) | DSSD |
| External causes of morbidity related to medical treatment | Y40 | 352 | 2.82 (2.49-3.18) | DSSD |
| External causes of morbidity related to other conditions | Y95 | 144 | 5.58 (4.70-6.64) | DSSD |
| Infectious gastroenteritis and colitis | A09 | 314 | 3.45 (3.06-3.89) | ESDD |
| Sepsis | A41 | 459 | 6.71 (6.04-7.45) | ESDD |
| Mycoses | B49 | 164 | 4.94 (4.21-5.79) | ESDD |
| Bacterial infectious agents | B96 | 453 | 4.95 (4.45-5.50) | ESDD |
| Colon cancer | C18 | 53 | 1.80 (1.37-2.37) | ESDD |
| Metastatic cancer | C77 | 281 | 3.82 (3.36-4.34) | ESDD |
| Iron deficiency anaemia | D50 | 313 | 3.45 (3.05-3.89) | ESDD |
| Other anaemias | D51 | 491 | 4.59 (4.14-5.09) | ESDD |
| Hypothyroid conditions | E00 | 153 | 1.60 (1.35-1.88) | ESDD |
| Diabetes | E10 | 922 | 16.07 (14.17-18.22) | ESDD |
| Malnutrition | E40 | 155 | 3.28 (2.79-3.87) | ESDD |
| Obesity | E66 | 469 | 2.05 (1.85-2.28) | ESDD |
| Disorders of lipoprotein metabolism and other lipidaemias | E78 | 573 | 2.07 (1.87-2.29) | ESDD |
| Disorders of mineral metabolism | E83 | 181 | 4.64 (3.98-5.41) | ESDD |
| Volume depletion | E86 | 290 | 5.80 (5.12-6.58) | ESDD |
| Delirium due to known physiological condition | F05 | 250 | 5.83 (5.11-6.66) | ESDD |
| Alcohol abuse | F10 | 103 | 2.38 (1.95-2.90) | ESDD |
| Tobacco abuse | F17 | 259 | 2.56 (2.25-2.92) | ESDD |
| Depression | F32 | 236 | 2.25 (1.97-2.58) | ESDD |
| Anxiety | F40 | 143 | 1.64 (1.38-1.94) | ESDD |
| Epilepsia | G40 | 64 | 3.02 (2.35-3.87) | ESDD |
| Sleep disorder | G47 | 190 | 3.18 (2.74-3.70) | ESDD |
| Diseases in nerves, nerve roots and nerve plexa | G50 | 159 | 1.64 (1.39-1.93) | ESDD |
| Polyneuropathies | G60 | 213 | 6.98 (6.05-8.04) | ESDD |
| Cerebral palsy and other paralytic syndromes | G80 | 102 | 4.01 (3.29-4.90) | ESDD |
| Disorders of the lens | H25 | 347 | 1.29 (1.14-1.45) | ESDD |
| Disorders of choroid and retina | H30 | 247 | 2.97 (2.60-3.39) | ESDD |
| Visual disturbances and blindness | H53 | 122 | 2.76 (2.30-3.31) | ESDD |
| Chronic rheumatic heart disease | I05 | 193 | 3.77 (3.25-4.38) | ESDD |
| Primary hypertension | I10 | 851 | 3.03 (2.69-3.42) | ESDD |
| Angina pectoris | I20 | 283 | 2.46 (2.16-2.79) | ESDD |
| Acute myocardial infarction | I21 | 239 | 3.62 (3.16-4.14) | ESDD |
| Other ischemic heart disease | I22 | 104 | 4.05 (3.33-4.94) | ESDD |
| Chronic ischaemic heart disease | I25 | 477 | 3.00 (2.70-3.34) | ESDD |
| Embolism and thrombosis | I26 | 247 | 3.59 (3.14-4.11) | ESDD |
| Non-rheumatic valve disorders | I34 | 250 | 3.33 (2.92-3.81) | ESDD |
| Atrial fibrillation and flutter | I48 | 565 | 3.54 (3.21-3.91) | ESDD |
| Other cardiac arrhythmias | I49 | 386 | 3.04 (2.72-3.40) | ESDD |
| Heart failure | I50 | 551 | 6.74 (6.10-7.45) | ESDD |
| Complications and ill-defined descriptions of heart disease | I51 | 344 | 3.74 (3.33-4.20) | ESDD |
| Stroke | I60 | 255 | 4.25 (3.72-4.85) | ESDD |
| Other cerebrovascular diseases | I65 | 256 | 4.15 (3.64-4.73) | ESDD |
| Aneurysm and dissection | I71 | 63 | 1.97 (1.54-2.54) | ESDD |
| Peripheral vascular disease | I73 | 213 | 5.42 (4.70-6.25) | ESDD |
| Varicose veins of lower extremities | I83 | 61 | 1.70 (1.32-2.20) | ESDD |
| Hypotension | I95 | 385 | 4.98 (4.46-5.57) | ESDD |
| Pneumonia | J12 | 743 | 7.27 (6.63-7.97) | ESDD |
| Other acute Lower respiratory infections | J20 | 327 | 4.59 (4.07-5.17) | ESDD |
| Chronic obstructive pulmonary disease | J44 | 346 | 3.32 (2.96-3.73) | ESDD |
| Asthma | J45 | 212 | 1.56 (1.36-1.81) | ESDD |
| Pleural effusion | J90 | 359 | 5.43 (4.84-6.09) | ESDD |
| Other diseases of the respiratory system | J95 | 224 | 3.45 (3.00-3.97) | ESDD |
| Disease of hard tissue of teeth | K00 | 37 | 1.77 (1.28-2.45) | ESDD |
| Oesophagitis | K20 | 97 | 1.89 (1.54-2.31) | ESDD |
| Other diseases of oesophagus | K22 | 118 | 1.49 (1.24-1.79) | ESDD |
| Gastric ulcer | K25 | 81 | 2.41 (1.93-3.01) | ESDD |
| Gastritis and duodenitis | K29 | 332 | 1.96 (1.74-2.21) | ESDD |
| Disease of stomach and duodenum | K31 | 182 | 2.43 (2.08-2.82) | ESDD |
| Umbilical hernia | K42 | 63 | 1.85 (1.44-2.38) | ESDD |
| Noninfective gastroenteritis and colitis | K52 | 149 | 2.42 (2.04-2.86) | ESDD |
| Other functional intestinal disorders | K59 | 383 | 3.47 (3.10-3.88) | ESDD |
| Other diseases of intestine | K63 | 224 | 1.43 (1.25-1.65) | ESDD |
| Other diseases of liver | K76 | 225 | 3.70 (3.22-4.25) | ESDD |
| Cholelithiasis | K80 | 170 | 1.80 (1.54-2.11) | ESDD |
| Disease of gallbladder and biliary tract | K82 | 92 | 2.53 (2.05-3.12) | ESDD |
| Other disease of digestive system | K92 | 227 | 2.63 (2.29-3.02) | ESDD |
| Skin and subcutaneous infections | L01 | 338 | 4.55 (4.04-5.12) | ESDD |
| Dermatitis | L20 | 93 | 2.48 (2.01-3.05) | ESDD |
| Autoimmune arthritis | M05 | 79 | 1.94 (1.55-2.43) | ESDD |
| Other inflammatory arthritis | M10 | 347 | 1.63 (1.45-1.83) | ESDD |
| Systemic connective tissue disorders | M30 | 56 | 1.63 (1.25-2.13) | ESDD |
| Spondylopathy | M48 | 111 | 1.84 (1.52-2.23) | ESDD |
| Dorsalgia | M54 | 206 | 1.81 (1.57-2.10) | ESDD |
| Other soft tissue disorders, not elsewhere classified | M79 | 218 | 2.25 (1.95-2.59) | ESDD |
| Obstructive and reflux uropathy | N13 | 68 | 2.29 (1.80-2.92) | ESDD |
| Acute renal failure | N17 | 863 | 13.82 (5.22-21.91) | ESDD |
| Chronic kidney disease | N18 | 616 | 5.45 (4.96-6.00) | ESDD |
| Urolithiasis | N20 | 77 | 1.98 (1.58-2.49) | ESDD |
| Other disorders of kidney and ureter | N29 | 127 | 3.01 (2.52-3.61) | ESDD |
| Disorders of urinary system, possibly infection | N39 | 454 | 3.93 (3.53-4.37) | ESDD |
| Injuries due to external causes | S00 | 440 | 2.48 (2.23-2.76) | ESDD |
| Complications due to medical treatment | T80 | 381 | 2.77 (2.47-3.10) | ESDD |
| Falls | W00 | 391 | 2.93 (2.62-3.27) | ESDD |
| Other external causes of accidental injury | W20 | 158 | 2.39 (2.03-2.81) | ESDD |
| External causes of morbidity related to medical treatment | Y40 | 504 | 2.95 (2.66-3.27) | ESDD |
| External causes of morbidity related to other conditions | Y95 | 247 | 7.26 (6.35-8.29) | ESDD |
| Infectious gastroenteritis and colitis | A09 | 250 | 4.90 (4.26-5.63) | GSDD |
| Sepsis | A41 | 434 | 5.95 (5.09-6.84) | GSDD |
| Mycoses | B49 | 116 | 5.97 (4.93-7.24) | GSDD |
| Bacterial infectious agents | B96 | 377 | 7.98 (7.05-9.04) | GSDD |
| Metastatic cancer | C77 | 174 | 4.06 (3.46-4.78) | GSDD |
| Benign tumors | D10 | 202 | 1.48 (1.27-1.72) | GSDD |
| Iron deficiency anaemia | D50 | 240 | 4.71 (4.08-5.43) | GSDD |
| Hypothyroid conditions | E00 | 120 | 2.15 (1.78-2.60) | GSDD |
| Diabetes | E10 | 278 | 2.76 (2.39-3.18) | GSDD |
| Malnutrition | E40 | 122 | 4.42 (3.67-5.34) | GSDD |
| Obesity | E66 | 266 | 1.85 (1.62-2.13) | GSDD |
| Disorders of lipoprotein metabolism and other lipidaemias | E78 | 347 | 2.03 (1.78-2.32) | GSDD |
| Disorders of mineral metabolism | E83 | 129 | 5.64 (4.70-6.77) | GSDD |
| Other disorders of fluid, electrolyte and acid-base balance | E87 | 602 | 16.71 (14.88-18.76) | GSDD |
| Delirium due to known physiological condition | F05 | 192 | 7.80 (6.68-9.10) | GSDD |
| Alcohol abuse | F10 | 64 | 2.46 (1.91-3.16) | GSDD |
| Tobacco abuse | F17 | 152 | 2.50 (2.11-2.97) | GSDD |
| Depression | F32 | 128 | 1.99 (1.66-2.39) | GSDD |
| Anxiety | F40 | 95 | 1.82 (1.48-2.24) | GSDD |
| Epilepsia | G40 | 32 | 2.50 (1.76-3.56) | GSDD |
| Sleep disorder | G47 | 97 | 2.67 (2.17-3.29) | GSDD |
| Diseases in nerves, nerve roots and nerve plexa | G50 | 91 | 1.54 (1.25-1.91) | GSDD |
| Polyneuropathies | G60 | 125 | 6.79 (5.64-8.17) | GSDD |
| Cerebral palsy and other paralytic syndromes | G80 | 55 | 3.59 (2.74-4.71) | GSDD |
| Disorders of the lens | H25 | 246 | 1.58 (1.37-1.83) | GSDD |
| Disorders of choroid and retina | H30 | 151 | 3.00 (2.53-3.56) | GSDD |
| Visual disturbances and blindness | H53 | 59 | 2.20 (1.70-2.86) | GSDD |
| Chronic rheumatic heart disease | I05 | 170 | 5.87 (4.99-6.91) | GSDD |
| Primary hypertension | I10 | 488 | 3.36 (2.86-3.96) | GSDD |
| Angina pectoris | I20 | 185 | 2.67 (2.27-3.13) | GSDD |
| Acute myocardial infarction | I21 | 145 | 3.68 (3.09-4.38) | GSDD |
| Other ischemic heart disease | I22 | 69 | 4.49 (3.52-5.73) | GSDD |
| Chronic ischaemic heart disease | I25 | 303 | 3.20 (2.79-3.66) | GSDD |
| Embolism and thrombosis | I26 | 139 | 3.37 (2.83-4.03) | GSDD |
| Non-rheumatic valve disorders | I34 | 210 | 5.03 (4.33-5.84) | GSDD |
| Atrial fibrillation and flutter | I48 | 414 | 5.02 (4.44-5.67) | GSDD |
| Other cardiac arrhythmias | I49 | 277 | 3.85 (3.37-4.41) | GSDD |
| Complications and ill-defined descriptions of heart disease | I51 | 275 | 5.39 (4.71-6.17) | GSDD |
| Stroke | I60 | 123 | 3.35 (2.78-4.04) | GSDD |
| Other cerebrovascular diseases | I65 | 165 | 4.50 (3.82-5.30) | GSDD |
| Aneurysm and dissection | I71 | 49 | 2.62 (1.97-3.49) | GSDD |
| Peripheral vascular disease | I73 | 136 | 5.81 (4.85-6.94) | GSDD |
| Varicose veins of lower extremities | I83 | 41 | 1.92 (1.41-2.63) | GSDD |
| Pneumonia | J12 | 516 | 9.47 (8.43-10.64) | GSDD |
| Other acute Lower respiratory infections | J20 | 224 | 5.43 (4.69-6.28) | GSDD |
| Chronic obstructive pulmonary disease | J44 | 226 | 3.69 (3.19-4.27) | GSDD |
| Other diseases of the respiratory system | J95 | 154 | 4.07 (3.43-4.82) | GSDD |
| Oesophagitis | K20 | 55 | 1.79 (1.36-2.34) | GSDD |
| Other diseases of oesophagus | K22 | 86 | 1.83 (1.47-2.28) | GSDD |
| Gastric ulcer | K25 | 61 | 3.10 (2.40-4.01) | GSDD |
| Gastritis and duodenitis | K29 | 201 | 2.04 (1.75-2.37) | GSDD |
| Disease of stomach and duodenum | K31 | 88 | 1.92 (1.55-2.39) | GSDD |
| Umbilical hernia | K42 | 49 | 2.42 (1.82-3.23) | GSDD |
| Ventral hernia | K43 | 31 | 1.92 (1.34-2.74) | GSDD |
| Diaphragmatic hernia | K44 | 151 | 1.44 (1.21-1.71) | GSDD |
| Noninfective gastroenteritis and colitis | K52 | 133 | 3.82 (3.19-4.58) | GSDD |
| Other functional intestinal disorders | K59 | 281 | 4.54 (3.97-5.19) | GSDD |
| Other diseases of intestine | K63 | 141 | 1.53 (1.28-1.82) | GSDD |
| Other disorders of peritoneum | K66 | 38 | 1.72 (1.24-2.37) | GSDD |
| Other diseases of liver | K76 | 130 | 3.55 (2.96-4.26) | GSDD |
| Cholelithiasis | K80 | 112 | 2.00 (1.65-2.44) | GSDD |
| Disease of gallbladder and biliary tract | K82 | 67 | 3.13 (2.44-4.00) | GSDD |
| Other disease of digestive system | K92 | 173 | 3.50 (2.98-4.12) | GSDD |
| Skin and subcutaneous infections | L01 | 237 | 5.56 (4.81-6.42) | GSDD |
| Dermatitis | L20 | 77 | 3.49 (2.77-4.40) | GSDD |
| Disorder of the skin and subcutaneous tissue | L98 | 44 | 1.86 (1.38-2.52) | GSDD |
| Autoimmune arthritis | M05 | 52 | 2.14 (1.62-2.83) | GSDD |
| Other inflammatory arthritis | M10 | 286 | 2.49 (2.17-2.84) | GSDD |
| Osteoarthritis | M15 | 293 | 1.32 (1.15-1.51) | GSDD |
| Other joint disorders | M20 | 151 | 1.49 (1.26-1.77) | GSDD |
| Systemic connective tissue disorders | M30 | 47 | 2.31 (1.72-3.09) | GSDD |
| Spondylosis | M47 | 87 | 1.58 (1.27-1.97) | GSDD |
| Spondylopathy | M48 | 70 | 1.94 (1.53-2.47) | GSDD |
| Dorsalgia | M54 | 135 | 2.01 (1.68-2.41) | GSDD |
| Other soft tissue disorders, not elsewhere classified | M79 | 172 | 3.09 (2.63-3.64) | GSDD |
| Obstructive and reflux uropathy | N13 | 124 | 7.65 (6.35-9.21) | GSDD |
| Acute renal failure | N17 | 803 | 27.65 (24.39-31.35) | GSDD |
| Urolithiasis | N20 | 61 | 2.66 (2.06-3.45) | GSDD |
| Other disorders of kidney and ureter | N29 | 146 | 6.26 (5.27-7.45) | GSDD |
| Bladder disorder | N32 | 98 | 2.44 (1.98-3.00) | GSDD |
| Disorders of urinary system, possibly infection | N39 | 399 | 6.93 (6.13-7.84) | GSDD |
| Injuries due to external causes | S00 | 298 | 2.97 (2.60-3.39) | GSDD |
| Complications due to medical treatment | T80 | 325 | 4.55 (3.99-5.18) | GSDD |
| Falls | W00 | 267 | 3.48 (3.04-3.99) | GSDD |
| Other external causes of accidental injury | W20 | 113 | 2.89 (2.38-3.51) | GSDD |
| External causes of morbidity related to medical treatment | Y40 | 398 | 4.66 (4.12-5.28) | GSDD |
| Infectious gastroenteritis and colitis | A09 | 932 | 2.55 (2.38-2.73) | MND |
| Sepsis | A41 | 1687 | 6.02 (5.70-6.36) | MND |
| Mycoses | B49 | 651 | 4.91 (4.53-5.32) | MND |
| Bacterial infectious agents | B96 | 1182 | 2.95 (2.77-3.13) | MND |
| Colon cancer | C18 | 611 | 5.71 (5.26-6.20) | MND |
| Benign tumors | D10 | 1211 | 1.40 (1.32-1.49) | MND |
| Iron deficiency anaemia | D50 | 555 | 1.44 (1.32-1.57) | MND |
| Other anaemias | D51 | 1761 | 4.00 (3.79-4.22) | MND |
| Hypothyroid conditions | E00 | 471 | 1.25 (1.14-1.37) | MND |
| Diabetes | E10 | 1453 | 1.50 (1.41-1.59) | MND |
| Malnutrition | E40 | 269 | 1.42 (1.26-1.61) | MND |
| Disorders of mineral metabolism | E83 | 814 | 5.33 (4.95-5.73) | MND |
| Volume depletion | E86 | 977 | 4.77 (4.46-5.10) | MND |
| Other disorders of fluid, electrolyte and acid-base balance | E87 | 1631 | 3.97 (3.75-4.19) | MND |
| Delirium due to known physiological condition | F05 | 398 | 2.19 (1.98-2.43) | MND |
| Alcohol abuse | F10 | 242 | 1.41 (1.24-1.60) | MND |
| Tobacco abuse | F17 | 849 | 2.13 (1.99-2.29) | MND |
| Depression | F32 | 612 | 1.44 (1.32-1.56) | MND |
| Anxiety | F40 | 454 | 1.35 (1.23-1.49) | MND |
| Epilepsia | G40 | 189 | 2.29 (1.98-2.65) | MND |
| Polyneuropathies | G60 | 264 | 2.05 (1.81-2.32) | MND |
| Cerebral palsy and other paralytic syndromes | G80 | 228 | 2.24 (1.97-2.56) | MND |
| Visual disturbances and blindness | H53 | 238 | 1.38 (1.21-1.57) | MND |
| Primary hypertension | I10 | 2919 | 1.31 (1.25-1.38) | MND |
| Embolism and thrombosis | I26 | 1277 | 4.97 (4.68-5.28) | MND |
| Atrial fibrillation and flutter | I48 | 1133 | 1.49 (1.39-1.58) | MND |
| Heart failure | I50 | 667 | 1.54 (1.42-1.66) | MND |
| Stroke | I60 | 428 | 1.68 (1.53-1.86) | MND |
| Other cerebrovascular diseases | I65 | 337 | 1.28 (1.15-1.43) | MND |
| Peripheral vascular disease | I73 | 237 | 1.41 (1.24-1.60) | MND |
| Hypotension | I95 | 856 | 2.54 (2.36-2.72) | MND |
| Pneumonia | J12 | 2132 | 4.44 (4.22-4.67) | MND |
| Other acute Lower respiratory infections | J20 | 1082 | 3.66 (3.43-3.90) | MND |
| Chronic obstructive pulmonary disease | J44 | 885 | 2.00 (1.87-2.15) | MND |
| Pleural effusion | J90 | 1546 | 5.92 (5.60-6.26) | MND |
| Other diseases of the respiratory system | J95 | 886 | 3.50 (3.27-3.76) | MND |
| Respiratory failure | J96 | 569 | 3.08 (2.83-3.36) | MND |
| Oesophagitis | K20 | 282 | 1.43 (1.27-1.61) | MND |
| Other diseases of oesophagus | K22 | 511 | 1.71 (1.57-1.87) | MND |
| Gastric ulcer | K25 | 185 | 1.42 (1.23-1.65) | MND |
| Gastritis and duodenitis | K29 | 861 | 1.24 (1.16-1.34) | MND |
| Disease of stomach and duodenum | K31 | 475 | 1.62 (1.48-1.78) | MND |
| Ventral hernia | K43 | 190 | 1.84 (1.59-2.13) | MND |
| Noninfective gastroenteritis and colitis | K52 | 615 | 2.61 (2.40-2.83) | MND |
| Other functional intestinal disorders | K59 | 1589 | 3.77 (3.57-3.99) | MND |
| Other diseases of intestine | K63 | 725 | 1.21 (1.12-1.31) | MND |
| Other disorders of peritoneum | K66 | 279 | 2.01 (1.79-2.27) | MND |
| Other diseases of liver | K76 | 589 | 2.40 (2.20-2.61) | MND |
| Cholelithiasis | K80 | 522 | 1.44 (1.31-1.57) | MND |
| Disease of gallbladder and biliary tract | K82 | 569 | 4.16 (3.82-4.53) | MND |
| Other disease of digestive system | K92 | 717 | 2.11 (1.95-2.28) | MND |
| Skin and subcutaneous infections | L01 | 586 | 1.75 (1.61-1.90) | MND |
| Dermatitis | L20 | 237 | 1.64 (1.44-1.87) | MND |
| Pressure ulcer | L89 | 478 | 4.34 (3.96-4.76) | MND |
| Dorsalgia | M54 | 717 | 1.64 (1.52-1.77) | MND |
| Other soft tissue disorders, not elsewhere classified | M79 | 633 | 1.65 (1.52-1.79) | MND |
| Obstructive and reflux uropathy | N13 | 545 | 4.92 (4.51-5.37) | MND |
| Acute renal failure | N17 | 1890 | 3.81 (3.61-4.01) | MND |
| Chronic kidney disease | N18 | 816 | 1.42 (1.32-1.53) | MND |
| Other disorders of kidney and ureter | N29 | 324 | 1.96 (1.75-2.19) | MND |
| Bladder disorder | N32 | 348 | 1.30 (1.17-1.45) | MND |
| Disorders of urinary system, possibly infection | N39 | 1335 | 2.69 (2.54-2.86) | MND |
| Injuries due to external causes | S00 | 903 | 1.17 (1.09-1.25) | MND |
| Complications due to medical treatment | T80 | 1417 | 2.58 (2.43-2.73) | MND |
| Falls | W00 | 741 | 1.29 (1.20-1.39) | MND |
| Other external causes of accidental injury | W20 | 343 | 1.30 (1.17-1.45) | MND |
| External causes of morbidity related to medical treatment | Y40 | 2282 | 3.60 (3.43-3.79) | MND |
| External causes of morbidity related to other conditions | Y95 | 575 | 3.92 (3.60-4.27) | MND |
| Infectious gastroenteritis and colitis | A09 | 492 | 2.94 (2.67-3.23) | RSDD |
| Sepsis | A41 | 864 | 7.07 (6.55-7.64) | RSDD |
| Mycoses | B49 | 323 | 5.42 (4.83-6.07) | RSDD |
| Bacterial infectious agents | B96 | 734 | 4.27 (3.93-4.63) | RSDD |
| Metastatic cancer | C77 | 634 | 5.01 (4.59-5.47) | RSDD |
| Iron deficiency anaemia | D50 | 423 | 2.45 (2.21-2.71) | RSDD |
| Other anaemias | D51 | 744 | 3.59 (3.31-3.90) | RSDD |
| Hypothyroid conditions | E00 | 252 | 1.43 (1.26-1.62) | RSDD |
| Diabetes | E10 | 807 | 1.98 (1.82-2.14) | RSDD |
| Malnutrition | E40 | 250 | 2.91 (2.56-3.31) | RSDD |
| Obesity | E66 | 627 | 1.35 (1.24-1.47) | RSDD |
| Disorders of lipoprotein metabolism and other lipidaemias | E78 | 912 | 1.51 (1.39-1.63) | RSDD |
| Disorders of mineral metabolism | E83 | 304 | 4.27 (3.80-4.80) | RSDD |
| Volume depletion | E86 | 513 | 5.64 (5.14-6.20) | RSDD |
| Other disorders of fluid, electrolyte and acid-base balance | E87 | 1168 | 7.42 (6.92-7.96) | RSDD |
| Delirium due to known physiological condition | F05 | 441 | 5.69 (5.15-6.29) | RSDD |
| Alcohol abuse | F10 | 224 | 2.93 (2.56-3.35) | RSDD |
| Tobacco abuse | F17 | 534 | 3.00 (2.74-3.29) | RSDD |
| Depression | F32 | 444 | 2.36 (2.14-2.61) | RSDD |
| Anxiety | F40 | 308 | 2.00 (1.78-2.24) | RSDD |
| Epilepsia | G40 | 132 | 3.49 (2.93-4.15) | RSDD |
| Sleep disorder | G47 | 237 | 2.11 (1.85-2.41) | RSDD |
| Polyneuropathies | G60 | 187 | 3.17 (2.74-3.67) | RSDD |
| Cerebral palsy and other paralytic syndromes | G80 | 196 | 4.28 (3.70-4.94) | RSDD |
| Disorders of choroid and retina | H30 | 225 | 1.34 (1.17-1.54) | RSDD |
| Visual disturbances and blindness | H53 | 174 | 2.15 (1.85-2.51) | RSDD |
| Other disorders of the ear | H90 | 218 | 1.60 (1.39-1.83) | RSDD |
| Chronic rheumatic heart disease | I05 | 295 | 3.15 (2.80-3.55) | RSDD |
| Primary hypertension | I10 | 1531 | 2.07 (1.91-2.24) | RSDD |
| Angina pectoris | I20 | 396 | 1.72 (1.55-1.91) | RSDD |
| Acute myocardial infarction | I21 | 269 | 2.11 (1.86-2.39) | RSDD |
| Other ischemic heart disease | I22 | 117 | 2.46 (2.05-2.96) | RSDD |
| Chronic ischaemic heart disease | I25 | 678 | 1.97 (1.81-2.15) | RSDD |
| Embolism and thrombosis | I26 | 444 | 3.56 (3.22-3.94) | RSDD |
| Non-rheumatic valve disorders | I34 | 321 | 2.27 (2.02-2.54) | RSDD |
| Atrial fibrillation and flutter | I48 | 955 | 3.21 (2.98-3.46) | RSDD |
| Other cardiac arrhythmias | I49 | 564 | 2.34 (2.14-2.56) | RSDD |
| Heart failure | I50 | 889 | 5.53 (5.12-5.97) | RSDD |
| Complications and ill-defined descriptions of heart disease | I51 | 528 | 3.06 (2.79-3.36) | RSDD |
| Stroke | I60 | 374 | 3.34 (3.00-3.71) | RSDD |
| Other cerebrovascular diseases | I65 | 385 | 3.34 (3.01-3.72) | RSDD |
| Aneurysm and dissection | I71 | 146 | 2.58 (2.18-3.04) | RSDD |
| Peripheral vascular disease | I73 | 258 | 3.44 (3.03-3.90) | RSDD |
| Hypotension | I95 | 653 | 4.58 (4.21-4.99) | RSDD |
| Pneumonia | J12 | 2020 | 16.35 (15.28-17.50) | RSDD |
| Other acute Lower respiratory infections | J20 | 701 | 5.65 (5.20-6.14) | RSDD |
| Chronic obstructive pulmonary disease | J44 | 1029 | 6.70 (6.22-7.21) | RSDD |
| Asthma | J45 | 418 | 1.76 (1.58-1.95) | RSDD |
| Pleural effusion | J90 | 729 | 6.28 (5.78-6.81) | RSDD |
| Other diseases of the respiratory system | J95 | 508 | 4.49 (4.09-4.93) | RSDD |
| Oesophagitis | K20 | 153 | 1.65 (1.40-1.94) | RSDD |
| Gastro-oesophageal reflux disease | K21 | 464 | 1.26 (1.14-1.39) | RSDD |
| Other diseases of oesophagus | K22 | 238 | 1.69 (1.48-1.92) | RSDD |
| Gastric ulcer | K25 | 119 | 1.96 (1.63-2.35) | RSDD |
| Gastritis and duodenitis | K29 | 467 | 1.47 (1.34-1.62) | RSDD |
| Disease of stomach and duodenum | K31 | 250 | 1.82 (1.60-2.07) | RSDD |
| Umbilical hernia | K42 | 95 | 1.53 (1.25-1.87) | RSDD |
| Diaphragmatic hernia | K44 | 428 | 1.33 (1.20-1.47) | RSDD |
| Noninfective gastroenteritis and colitis | K52 | 257 | 2.30 (2.02-2.61) | RSDD |
| Other functional intestinal disorders | K59 | 712 | 3.60 (3.32-3.91) | RSDD |
| Other diseases of liver | K76 | 275 | 2.40 (2.12-2.71) | RSDD |
| Cholelithiasis | K80 | 274 | 1.59 (1.40-1.79) | RSDD |
| Disease of gallbladder and biliary tract | K82 | 133 | 2.01 (1.69-2.38) | RSDD |
| Other disease of digestive system | K92 | 360 | 2.27 (2.04-2.54) | RSDD |
| Skin and subcutaneous infections | L01 | 467 | 3.22 (2.92-3.55) | RSDD |
| Dermatitis | L20 | 152 | 2.24 (1.91-2.64) | RSDD |
| Papulosquamous disorders | L40 | 88 | 1.77 (1.43-2.19) | RSDD |
| Disorder of the skin and subcutaneous tissue | L98 | 120 | 1.70 (1.42-2.04) | RSDD |
| Autoimmune arthritis | M05 | 144 | 1.97 (1.67-2.33) | RSDD |
| Other inflammatory arthritis | M10 | 586 | 1.48 (1.35-1.62) | RSDD |
| Other joint disorders | M20 | 387 | 1.27 (1.14-1.41) | RSDD |
| Systemic connective tissue disorders | M30 | 119 | 1.93 (1.61-2.32) | RSDD |
| Deforming dorsopathies | M40 | 113 | 1.77 (1.47-2.14) | RSDD |
| Spondylosis | M47 | 260 | 1.57 (1.38-1.78) | RSDD |
| Spondylopathy | M48 | 209 | 1.94 (1.68-2.23) | RSDD |
| Dorsalgia | M54 | 377 | 1.85 (1.66-2.06) | RSDD |
| Other soft tissue disorders, not elsewhere classified | M79 | 360 | 2.04 (1.83-2.28) | RSDD |
| Obstructive and reflux uropathy | N13 | 97 | 1.80 (1.47-2.21) | RSDD |
| Acute renal failure | N17 | 1308 | 6.90 (6.44-7.39) | RSDD |
| Chronic kidney disease | N18 | 745 | 3.13 (2.89-3.39) | RSDD |
| Other disorders of kidney and ureter | N29 | 160 | 2.06 (1.76-2.42) | RSDD |
| Bladder disorder | N32 | 174 | 1.37 (1.17-1.59) | RSDD |
| Disorders of urinary system, possibly infection | N39 | 770 | 3.56 (3.29-3.86) | RSDD |
| Injuries due to external causes | S00 | 826 | 2.68 (2.47-2.89) | RSDD |
| Complications due to medical treatment | T80 | 574 | 2.17 (1.98-2.37) | RSDD |
| Falls | W00 | 702 | 2.95 (2.71-3.20) | RSDD |
| Other external causes of accidental injury | W20 | 342 | 2.94 (2.63-3.29) | RSDD |
| External causes of morbidity related to medical treatment | Y40 | 872 | 2.74 (2.54-2.96) | RSDD |
| Infectious gastroenteritis and colitis | A09 | 111 | 2.02 (1.66-2.46) | UCD |
| Sepsis | A41 | 223 | 5.21 (4.50-6.05) | UCD |
| Mycoses | B49 | 59 | 2.89 (2.22-3.76) | UCD |
| Bacterial infectious agents | B96 | 222 | 4.10 (3.53-4.76) | UCD |
| Colon cancer | C18 | 41 | 2.46 (1.80-3.36) | UCD |
| Metastatic cancer | C77 | 152 | 3.11 (2.62-3.70) | UCD |
| Iron deficiency anaemia | D50 | 87 | 1.57 (1.26-1.95) | UCD |
| Other anaemias | D51 | 222 | 3.34 (2.88-3.88) | UCD |
| Diabetes | E10 | 185 | 1.37 (1.16-1.61) | UCD |
| Malnutrition | E40 | 45 | 1.65 (1.22-2.22) | UCD |
| Disorders of mineral metabolism | E83 | 58 | 2.41 (1.85-3.14) | UCD |
| Volume depletion | E86 | 107 | 3.40 (2.79-4.16) | UCD |
| Other disorders of fluid, electrolyte and acid-base balance | E87 | 278 | 4.89 (4.27-5.61) | UCD |
| Delirium due to known physiological condition | F05 | 71 | 2.69 (2.12-3.43) | UCD |
| Alcohol abuse | F10 | 70 | 2.94 (2.30-3.74) | UCD |
| Tobacco abuse | F17 | 119 | 2.05 (1.69-2.48) | UCD |
| Depression | F32 | 102 | 1.72 (1.41-2.12) | UCD |
| Anxiety | F40 | 79 | 1.65 (1.31-2.07) | UCD |
| Epilepsia | G40 | 24 | 1.99 (1.33-2.99) | UCD |
| Polyneuropathies | G60 | 41 | 2.19 (1.61-3.00) | UCD |
| Cerebral palsy and other paralytic syndromes | G80 | 41 | 2.76 (2.02-3.77) | UCD |
| Chronic rheumatic heart disease | I05 | 85 | 2.91 (2.33-3.63) | UCD |
| Acute myocardial infarction | I21 | 87 | 2.23 (1.79-2.78) | UCD |
| Other ischemic heart disease | I22 | 29 | 1.95 (1.35-2.82) | UCD |
| Chronic ischaemic heart disease | I25 | 190 | 1.73 (1.47-2.02) | UCD |
| Embolism and thrombosis | I26 | 125 | 3.09 (2.57-3.73) | UCD |
| Non-rheumatic valve disorders | I34 | 111 | 2.65 (2.18-3.23) | UCD |
| Atrial fibrillation and flutter | I48 | 208 | 2.04 (1.75-2.38) | UCD |
| Other cardiac arrhythmias | I49 | 136 | 1.76 (1.47-2.11) | UCD |
| Heart failure | I50 | 162 | 2.80 (2.37-3.31) | UCD |
| Complications and ill-defined descriptions of heart disease | I51 | 120 | 2.18 (1.80-2.63) | UCD |
| Stroke | I60 | 104 | 2.94 (2.40-3.60) | UCD |
| Other cerebrovascular diseases | I65 | 72 | 1.92 (1.51-2.44) | UCD |
| Aneurysm and dissection | I71 | 55 | 3.20 (2.44-4.20) | UCD |
| Peripheral vascular disease | I73 | 63 | 2.66 (2.06-3.43) | UCD |
| Hypotension | I95 | 173 | 3.69 (3.14-4.35) | UCD |
| Pneumonia | J12 | 323 | 4.69 (4.12-5.35) | UCD |
| Other acute Lower respiratory infections | J20 | 111 | 2.49 (2.04-3.03) | UCD |
| Chronic obstructive pulmonary disease | J44 | 134 | 2.10 (1.75-2.51) | UCD |
| Pleural effusion | J90 | 225 | 5.88 (5.07-6.82) | UCD |
| Other diseases of the respiratory system | J95 | 121 | 3.25 (2.69-3.93) | UCD |
| Respiratory failure | J96 | 163 | 6.46 (5.47-7.64) | UCD |
| Other diseases of oesophagus | K22 | 78 | 1.81 (1.44-2.28) | UCD |
| Noninfective gastroenteritis and colitis | K52 | 82 | 2.38 (1.90-2.98) | UCD |
| Other functional intestinal disorders | K59 | 138 | 2.02 (1.69-2.42) | UCD |
| Other disorders of peritoneum | K66 | 39 | 1.93 (1.40-2.66) | UCD |
| Other diseases of liver | K76 | 71 | 1.96 (1.54-2.50) | UCD |
| Disease of gallbladder and biliary tract | K82 | 39 | 1.86 (1.35-2.56) | UCD |
| Other disease of digestive system | K92 | 97 | 1.95 (1.59-2.41) | UCD |
| Skin and subcutaneous infections | L01 | 103 | 2.16 (1.76-2.65) | UCD |
| Pressure ulcer | L89 | 77 | 4.69 (3.71-5.91) | UCD |
| Obstructive and reflux uropathy | N13 | 33 | 1.93 (1.36-2.73) | UCD |
| Acute renal failure | N17 | 312 | 4.51 (3.95-5.14) | UCD |
| Chronic kidney disease | N18 | 172 | 2.23 (1.90-2.63) | UCD |
| Other disorders of kidney and ureter | N29 | 46 | 1.92 (1.43-2.58) | UCD |
| Disorders of urinary system, possibly infection | N39 | 165 | 2.21 (1.87-2.61) | UCD |
| Injuries due to external causes | S00 | 237 | 2.47 (2.14-2.86) | UCD |
| Complications due to medical treatment | T80 | 283 | 4.09 (3.56-4.70) | UCD |
| Falls | W00 | 188 | 2.49 (2.13-2.92) | UCD |
| Other external causes of accidental injury | W20 | 76 | 2.08 (1.65-2.63) | UCD |
| External causes of morbidity related to medical treatment | Y40 | 302 | 3.26 (2.85-3.73) | UCD |
| External causes of morbidity related to other conditions | Y95 | 104 | 4.85 (3.96-5.93) | UCD |

^*^Combined ICD-10 codes derived from the original ICD-10 code and are displayed in the Additional file 1.

^#^Number of the MAFLD participants dead after diagnosing corresponding medical conditions.

HR, hazards ratio; CI, confidence interval. MAFLD, metabolic dysfunction-associated fatty liver disease; MND, malignant neoplasms death; ESDD, endocrine system disease death; CVDD, cardiovascular disease death; RSDD, respiratory system disease death; DSSD, digestive system disease death; GSDD, genitourinary system disease death; UCD, unnatural cause death.

Additional file 1: Table S5. Temporal disease pairs with a significantly increased risk of disease 2 (D2) after disease 1 (D1) in dead individuals with MAFLD.

| **Causes of death** | **D1→D2 code^*^** | **D1 description** | **D2 description** | **No.^#^** | **OR (95% CI)** |
| --- | --- | --- | --- | --- | --- |
| CVDD | D51→E87 | Other anaemias | Other disorders of fluid, electrolyte and acid-base balance | 302 | 4.27 (3.34 -5.45) |
| CVDD | D51→J12 | Other anaemias | Pneumonia | 304 | 4.61 (3.58 -5.92) |
| CVDD | D51→N17 | Other anaemias | Acute renal failure | 302 | 5.38 (4.11 -7.04) |
| CVDD | E10→E87 | Diabetes | Other disorders of fluid, electrolyte and acid-base balance | 361 | 3.66 (2.97 -4.52) |
| CVDD | E10→I50 | Diabetes | Heart failure | 302 | 2.98 (2.39 -3.71) |
| CVDD | E10→J12 | Diabetes | Pneumonia | 370 | 3.61 (2.93 -4.45) |
| CVDD | E10→N17 | Diabetes | Acute renal failure | 424 | 4.01 (3.28 -4.90) |
| CVDD | E78→E87 | Disorders of lipoprotein metabolism and other lipidaemias | Other disorders of fluid, electrolyte and acid-base balance | 398 | 3.00 (2.49 -3.62) |
| CVDD | E78→I50 | Disorders of lipoprotein metabolism and other lipidaemias | Heart failure | 339 | 2.85 (2.32 -3.49) |
| CVDD | E78→J12 | Disorders of lipoprotein metabolism and other lipidaemias | Pneumonia | 409 | 3.87 (3.16 -4.74) |
| CVDD | E78→N17 | Disorders of lipoprotein metabolism and other lipidaemias | Acute renal failure | 428 | 3.40 (2.81 -4.12) |
| CVDD | I10→A41 | Primary hypertension | Sepsis | 403 | 1.47 (1.28 -1.70) |
| CVDD | I10→D51 | Primary hypertension | Other anaemias | 376 | 1.58 (1.36 -1.84) |
| CVDD | I10→E78 | Primary hypertension | Disorders of lipoprotein metabolism and other lipidaemias | 398 | 1.92 (1.65 -2.23) |
| CVDD | I10→E87 | Primary hypertension | Other disorders of fluid, electrolyte and acid-base balance | 617 | 1.69 (1.50 -1.90) |
| CVDD | I10→I25 | Primary hypertension | Chronic ischaemic heart disease | 375 | 1.46 (1.25 -1.69) |
| CVDD | I10→I50 | Primary hypertension | Heart failure | 538 | 1.28 (1.14 -1.45) |
| CVDD | I10→J12 | Primary hypertension | Pneumonia | 677 | 1.88 (1.68 -2.12) |
| CVDD | I10→J90 | Primary hypertension | Pleural effusion | 378 | 1.34 (1.16 -1.55) |
| CVDD | I10→K59 | Primary hypertension | Other functional intestinal disorders | 337 | 1.36 (1.17 -1.59) |
| CVDD | I10→N17 | Primary hypertension | Acute renal failure | 711 | 1.77 (1.58 -1.97) |
| CVDD | I10→N39 | Primary hypertension | Disorders of urinary system, possibly infection | 375 | 1.44 (1.24 -1.67) |
| CVDD | I10→S00 | Primary hypertension | Injuries due to external causes | 326 | 1.39 (1.19 -1.63) |
| CVDD | I10→Y40 | Primary hypertension | External causes of morbidity related to medical treatment | 436 | 1.76 (1.53 -2.03) |
| CVDD | I25→E87 | Chronic ischaemic heart disease | Other disorders of fluid, electrolyte and acid-base balance | 344 | 4.59 (3.61 -5.84) |
| CVDD | I25→I50 | Chronic ischaemic heart disease | Heart failure | 326 | 4.30 (3.37 -5.48) |
| CVDD | I25→J12 | Chronic ischaemic heart disease | Pneumonia | 355 | 4.59 (3.62 -5.82) |
| CVDD | I25→N17 | Chronic ischaemic heart disease | Acute renal failure | 392 | 4.52 (3.61 -5.65) |
| CVDD | I48→E87 | Atrial fibrillation and flutter | Other disorders of fluid, electrolyte and acid-base balance | 399 | 3.71 (3.03 -4.54) |
| CVDD | I48→I50 | Atrial fibrillation and flutter | Heart failure | 382 | 3.66 (2.97 -4.51) |
| CVDD | I48→J12 | Atrial fibrillation and flutter | Pneumonia | 415 | 4.76 (3.83 -5.93) |
| CVDD | I48→N17 | Atrial fibrillation and flutter | Acute renal failure | 444 | 5.15 (4.15 -6.41) |
| CVDD | I48→N18 | Atrial fibrillation and flutter | Chronic kidney disease | 318 | 3.37 (2.70 -4.20) |
| CVDD | I50→E87 | Heart failure | Other disorders of fluid, electrolyte and acid-base balance | 393 | 6.13 (4.75 -7.91) |
| CVDD | I50→J12 | Heart failure | Pneumonia | 374 | 5.81 (4.51 -7.49) |
| CVDD | I50→N17 | Heart failure | Acute renal failure | 460 | 6.97 (5.44 -8.94) |
| CVDD | N17→E87 | Acute renal failure | Other disorders of fluid, electrolyte and acid-base balance | 359 | 4.26 (3.43 -5.30) |
| CVDD | N18→E87 | Chronic kidney disease | Other disorders of fluid, electrolyte and acid-base balance | 375 | 5.11 (4.02 -6.51) |
| CVDD | N18→J12 | Chronic kidney disease | Pneumonia | 344 | 5.24 (4.06 -6.75) |
| CVDD | Y40→E87 | External causes of morbidity related to medical treatment | Other disorders of fluid, electrolyte and acid-base balance | 307 | 3.60 (2.87 -4.52) |
| CVDD | Y40→J12 | External causes of morbidity related to medical treatment | Pneumonia | 342 | 4.25 (3.38 -5.34) |
| CVDD | Y40→N17 | External causes of morbidity related to medical treatment | Acute renal failure | 315 | 4.14 (3.28 -5.22) |
| DSSD | D10→E87 | Benign tumors | Other disorders of fluid, electrolyte and acid-base balance | 75 | 5.31 (3.11 -9.06) |
| DSSD | D51→A41 | Other anaemias | Sepsis | 88 | 3.16 (2.11 -4.73) |
| DSSD | D51→B96 | Other anaemias | Bacterial infectious agents | 75 | 3.73 (2.33 -5.97) |
| DSSD | D51→E87 | Other anaemias | Other disorders of fluid, electrolyte and acid-base balance | 117 | 4.43 (2.98 -6.59) |
| DSSD | D51→J12 | Other anaemias | Pneumonia | 90 | 3.56 (2.32 -5.45) |
| DSSD | E10→A41 | Diabetes | Sepsis | 78 | 2.62 (1.76 -3.89) |
| DSSD | E10→D51 | Diabetes | Other anaemias | 71 | 2.96 (1.90 -4.62) |
| DSSD | E10→E87 | Diabetes | Other disorders of fluid, electrolyte and acid-base balance | 114 | 3.20 (2.25 -4.54) |
| DSSD | E10→J12 | Diabetes | Pneumonia | 78 | 3.58 (2.32 -5.53) |
| DSSD | E66→A41 | Obesity | Sepsis | 78 | 3.82 (2.39 -6.11) |
| DSSD | E66→E87 | Obesity | Other disorders of fluid, electrolyte and acid-base balance | 85 | 4.63 (2.82 -7.60) |
| DSSD | E66→J12 | Obesity | Pneumonia | 71 | 4.41 (2.61 -7.47) |
| DSSD | I10→A41 | Primary hypertension | Sepsis | 148 | 1.73 (1.38 -2.19) |
| DSSD | I10→E87 | Primary hypertension | Other disorders of fluid, electrolyte and acid-base balance | 201 | 1.88 (1.53 -2.30) |
| DSSD | I10→J12 | Primary hypertension | Pneumonia | 165 | 2.34 (1.85 -2.97) |
| DSSD | I10→N18 | Primary hypertension | Chronic kidney disease | 81 | 1.79 (1.30 -2.47) |
| DSSD | I10→Y40 | Primary hypertension | External causes of morbidity related to medical treatment | 128 | 1.99 (1.53 -2.58) |
| DSSD | I25→E87 | Chronic ischaemic heart disease | Other disorders of fluid, electrolyte and acid-base balance | 71 | 6.07 (3.45 -10.69) |
| DSSD | I48→E87 | Atrial fibrillation and flutter | Other disorders of fluid, electrolyte and acid-base balance | 77 | 3.38 (2.19 -5.24) |
| DSSD | K21→E87 | Gastro-oesophageal reflux disease | Other disorders of fluid, electrolyte and acid-base balance | 77 | 9.11 (4.58 -18.13) |
| DSSD | K29→A41 | Gastritis and duodenitis | Sepsis | 73 | 7.70 (3.98 -14.88) |
| DSSD | K29→E87 | Gastritis and duodenitis | Other disorders of fluid, electrolyte and acid-base balance | 115 | 11.09 (5.98 -20.56) |
| DSSD | K29→J12 | Gastritis and duodenitis | Pneumonia | 91 | 10.00 (5.22 -19.16) |
| DSSD | K31→E87 | Disease of stomach and duodenum | Other disorders of fluid, electrolyte and acid-base balance | 72 | 9.38 (4.52 -19.43) |
| DSSD | N18→E87 | Chronic kidney disease | Other disorders of fluid, electrolyte and acid-base balance | 85 | 5.82 (3.48 -9.74) |
| DSSD | S00→E87 | Injuries due to external causes | Other disorders of fluid, electrolyte and acid-base balance | 75 | 5.25 (3.08 -8.96) |
| DSSD | Y40→A41 | External causes of morbidity related to medical treatment | Sepsis | 95 | 3.48 (2.34 -5.19) |
| DSSD | Y40→E87 | External causes of morbidity related to medical treatment | Other disorders of fluid, electrolyte and acid-base balance | 92 | 3.72 (2.47 -5.61) |
| DSSD | Y40→I95 | External causes of morbidity related to medical treatment | Hypotension | 71 | 2.11 (1.41 -3.16) |
| DSSD | Y40→J12 | External causes of morbidity related to medical treatment | Pneumonia | 89 | 4.86 (3.07 -7.70) |
| ESDD | D51→A41 | Other anaemias | Sepsis | 121 | 4.24 (2.90 -6.20) |
| ESDD | D51→B96 | Other anaemias | Bacterial infectious agents | 107 | 4.76 (3.09 -7.33) |
| ESDD | D51→J12 | Other anaemias | Pneumonia | 150 | 5.59 (3.84 -8.15) |
| ESDD | E10→A09 | Diabetes | Infectious gastroenteritis and colitis | 113 | 3.03 (2.11 -4.35) |
| ESDD | E10→A41 | Diabetes | Sepsis | 203 | 3.34 (2.53 -4.40) |
| ESDD | E10→B96 | Diabetes | Bacterial infectious agents | 151 | 3.28 (2.39 -4.50) |
| ESDD | E10→C77 | Diabetes | Metastatic cancer | 134 | 3.51 (2.48 -4.97) |
| ESDD | E10→D50 | Diabetes | Iron deficiency anaemia | 104 | 3.57 (2.38 -5.35) |
| ESDD | E10→D51 | Diabetes | Other anaemias | 159 | 3.07 (2.27 -4.17) |
| ESDD | E10→E66 | Diabetes | Obesity | 141 | 3.15 (2.27 -4.37) |
| ESDD | E10→E78 | Diabetes | Disorders of lipoprotein metabolism and other lipidaemias | 136 | 3.51 (2.48 -4.97) |
| ESDD | E10→E86 | Diabetes | Volume depletion | 111 | 2.35 (1.68 -3.28) |
| ESDD | E10→F05 | Diabetes | Delirium due to known physiological condition | 115 | 2.54 (1.81 -3.58) |
| ESDD | E10→I25 | Diabetes | Chronic ischaemic heart disease | 119 | 3.28 (2.30 -4.67) |
| ESDD | E10→I26 | Diabetes | Embolism and thrombosis | 102 | 3.08 (2.12 -4.49) |
| ESDD | E10→I48 | Diabetes | Atrial fibrillation and flutter | 162 | 3.07 (2.28 -4.14) |
| ESDD | E10→I49 | Diabetes | Other cardiac arrhythmias | 128 | 2.93 (2.10 -4.10) |
| ESDD | E10→I50 | Diabetes | Heart failure | 176 | 3.13 (2.35 -4.18) |
| ESDD | E10→I51 | Diabetes | Complications and ill-defined descriptions of heart disease | 118 | 2.63 (1.87 -3.69) |
| ESDD | E10→I95 | Diabetes | Hypotension | 133 | 2.88 (2.09 -3.97) |
| ESDD | E10→J12 | Diabetes | Pneumonia | 301 | 3.44 (2.75 -4.32) |
| ESDD | E10→J20 | Diabetes | Other acute Lower respiratory infections | 120 | 3.20 (2.24 -4.56) |
| ESDD | E10→J90 | Diabetes | Pleural effusion | 147 | 2.72 (2.01 -3.68) |
| ESDD | E10→K29 | Diabetes | Gastritis and duodenitis | 103 | 2.63 (1.84 -3.77) |
| ESDD | E10→K59 | Diabetes | Other functional intestinal disorders | 158 | 3.31 (2.43 -4.51) |
| ESDD | E10→L01 | Diabetes | Skin and subcutaneous infections | 103 | 3.21 (2.18 -4.71) |
| ESDD | E10→N17 | Diabetes | Acute renal failure | 317 | 3.39 (2.72 -4.22) |
| ESDD | E10→N18 | Diabetes | Chronic kidney disease | 200 | 2.97 (2.28 -3.88) |
| ESDD | E10→N39 | Diabetes | Disorders of urinary system, possibly infection | 169 | 3.09 (2.30 -4.15) |
| ESDD | E10→S00 | Diabetes | Injuries due to external causes | 132 | 3.26 (2.31 -4.58) |
| ESDD | E10→T80 | Diabetes | Complications due to medical treatment | 118 | 2.80 (1.98 -3.94) |
| ESDD | E10→W00 | Diabetes | Falls | 121 | 3.00 (2.12 -4.24) |
| ESDD | E10→Y40 | Diabetes | External causes of morbidity related to medical treatment | 163 | 3.75 (2.73 -5.16) |
| ESDD | E66→A41 | Obesity | Sepsis | 121 | 4.61 (3.06 -6.93) |
| ESDD | E66→I50 | Obesity | Heart failure | 102 | 7.20 (4.20 -12.36) |
| ESDD | E66→J12 | Obesity | Pneumonia | 171 | 6.75 (4.54 -10.04) |
| ESDD | E66→N17 | Obesity | Acute renal failure | 175 | 7.56 (4.98 -11.47) |
| ESDD | E66→N18 | Obesity | Chronic kidney disease | 101 | 5.94 (3.61 -9.79) |
| ESDD | E78→A41 | Disorders of lipoprotein metabolism and other lipidaemias | Sepsis | 128 | 2.57 (1.88 -3.52) |
| ESDD | E78→B96 | Disorders of lipoprotein metabolism and other lipidaemias | Bacterial infectious agents | 119 | 2.98 (2.09 -4.24) |
| ESDD | E78→D51 | Disorders of lipoprotein metabolism and other lipidaemias | Other anaemias | 121 | 3.04 (2.17 -4.26) |
| ESDD | E78→I48 | Disorders of lipoprotein metabolism and other lipidaemias | Atrial fibrillation and flutter | 104 | 2.97 (2.07 -4.28) |
| ESDD | E78→I50 | Disorders of lipoprotein metabolism and other lipidaemias | Heart failure | 123 | 2.96 (2.12 -4.13) |
| ESDD | E78→I95 | Disorders of lipoprotein metabolism and other lipidaemias | Hypotension | 101 | 2.82 (1.96 -4.06) |
| ESDD | E78→J12 | Disorders of lipoprotein metabolism and other lipidaemias | Pneumonia | 192 | 4.98 (3.61 -6.86) |
| ESDD | E78→J90 | Disorders of lipoprotein metabolism and other lipidaemias | Pleural effusion | 107 | 3.18 (2.21 -4.58) |
| ESDD | E78→K59 | Disorders of lipoprotein metabolism and other lipidaemias | Other functional intestinal disorders | 116 | 3.18 (2.22 -4.56) |
| ESDD | E78→N17 | Disorders of lipoprotein metabolism and other lipidaemias | Acute renal failure | 199 | 3.68 (2.78 -4.87) |
| ESDD | E78→N18 | Disorders of lipoprotein metabolism and other lipidaemias | Chronic kidney disease | 142 | 3.08 (2.25 -4.21) |
| ESDD | E78→N39 | Disorders of lipoprotein metabolism and other lipidaemias | Disorders of urinary system, possibly infection | 102 | 3.96 (2.62 -6.00) |
| ESDD | E78→Y40 | Disorders of lipoprotein metabolism and other lipidaemias | External causes of morbidity related to medical treatment | 120 | 4.67 (3.15 -6.92) |
| ESDD | H25→J12 | Disorders of the lens | Pneumonia | 116 | 8.25 (4.91 -13.86) |
| ESDD | H25→N17 | Disorders of the lens | Acute renal failure | 157 | 7.08 (4.62 -10.86) |
| ESDD | H25→N18 | Disorders of the lens | Chronic kidney disease | 104 | 5.33 (3.35 -8.50) |
| ESDD | I10→A41 | Primary hypertension | Sepsis | 184 | 1.74 (1.41 -2.15) |
| ESDD | I10→C77 | Primary hypertension | Metastatic cancer | 111 | 2.03 (1.53 -2.68) |
| ESDD | I10→D51 | Primary hypertension | Other anaemias | 152 | 1.81 (1.42 -2.30) |
| ESDD | I10→E78 | Primary hypertension | Disorders of lipoprotein metabolism and other lipidaemias | 153 | 2.02 (1.59 -2.57) |
| ESDD | I10→I25 | Primary hypertension | Chronic ischaemic heart disease | 138 | 1.84 (1.44 -2.36) |
| ESDD | I10→I48 | Primary hypertension | Atrial fibrillation and flutter | 178 | 1.62 (1.31 -2.01) |
| ESDD | I10→I50 | Primary hypertension | Heart failure | 188 | 1.54 (1.25 -1.90) |
| ESDD | I10→J12 | Primary hypertension | Pneumonia | 288 | 2.26 (1.89 -2.69) |
| ESDD | I10→K59 | Primary hypertension | Other functional intestinal disorders | 157 | 1.58 (1.25 -1.98) |
| ESDD | I10→N17 | Primary hypertension | Acute renal failure | 323 | 2.17 (1.83 -2.56) |
| ESDD | I10→N39 | Primary hypertension | Disorders of urinary system, possibly infection | 162 | 1.77 (1.41 -2.23) |
| ESDD | I10→Y40 | Primary hypertension | External causes of morbidity related to medical treatment | 185 | 1.91 (1.54 -2.36) |
| ESDD | I20→N17 | Angina pectoris | Acute renal failure | 101 | 8.46 (4.76 -15.03) |
| ESDD | I25→I50 | Chronic ischaemic heart disease | Heart failure | 104 | 4.96 (3.20 -7.69) |
| ESDD | I25→J12 | Chronic ischaemic heart disease | Pneumonia | 155 | 5.41 (3.71 -7.88) |
| ESDD | I25→N17 | Chronic ischaemic heart disease | Acute renal failure | 154 | 4.28 (3.02 -6.07) |
| ESDD | I25→N18 | Chronic ischaemic heart disease | Chronic kidney disease | 103 | 4.42 (2.89 -6.77) |
| ESDD | I48→A41 | Atrial fibrillation and flutter | Sepsis | 106 | 4.69 (3.07 -7.17) |
| ESDD | I48→J12 | Atrial fibrillation and flutter | Pneumonia | 150 | 5.03 (3.50 -7.23) |
| ESDD | I48→N17 | Atrial fibrillation and flutter | Acute renal failure | 174 | 6.55 (4.49 -9.56) |
| ESDD | J12→A41 | Pneumonia | Sepsis | 123 | 3.02 (2.19 -4.17) |
| ESDD | J44→J12 | Chronic obstructive pulmonary disease | Pneumonia | 113 | 6.95 (4.29 -11.24) |
| ESDD | K29→J12 | Gastritis and duodenitis | Pneumonia | 117 | 6.74 (4.16 -10.91) |
| ESDD | K29→N17 | Gastritis and duodenitis | Acute renal failure | 119 | 5.46 (3.53 -8.43) |
| ESDD | M10→J12 | Other inflammatory arthritis | Pneumonia | 115 | 8.31 (4.95 -13.96) |
| ESDD | M10→N17 | Other inflammatory arthritis | Acute renal failure | 124 | 6.22 (4.00 -9.66) |
| ESDD | N17→A41 | Acute renal failure | Sepsis | 169 | 2.82 (2.15 -3.69) |
| ESDD | N17→F05 | Acute renal failure | Delirium due to known physiological condition | 107 | 1.77 (1.30 -2.42) |
| ESDD | N17→Y95 | Acute renal failure | External causes of morbidity related to other conditions | 112 | 2.37 (1.71 -3.29) |
| ESDD | N18→A41 | Chronic kidney disease | Sepsis | 145 | 4.64 (3.18 -6.75) |
| ESDD | N18→I95 | Chronic kidney disease | Hypotension | 105 | 6.53 (3.92 -10.88) |
| ESDD | N18→J12 | Chronic kidney disease | Pneumonia | 176 | 5.71 (3.96 -8.21) |
| ESDD | N18→J90 | Chronic kidney disease | Pleural effusion | 108 | 4.22 (2.78 -6.42) |
| ESDD | N18→K59 | Chronic kidney disease | Other functional intestinal disorders | 115 | 4.56 (3.00 -6.91) |
| ESDD | N18→Y95 | Chronic kidney disease | External causes of morbidity related to other conditions | 102 | 5.05 (3.16 -8.06) |
| ESDD | N39→A41 | Disorders of urinary system, possibly infection | Sepsis | 102 | 3.22 (2.22 -4.65) |
| ESDD | S00→A41 | Injuries due to external causes | Sepsis | 103 | 3.56 (2.41 -5.27) |
| ESDD | S00→J12 | Injuries due to external causes | Pneumonia | 130 | 4.14 (2.88 -5.96) |
| ESDD | T80→J12 | Complications due to medical treatment | Pneumonia | 112 | 3.76 (2.56 -5.52) |
| ESDD | Y40→A41 | External causes of morbidity related to medical treatment | Sepsis | 117 | 3.36 (2.35 -4.80) |
| ESDD | Y40→J12 | External causes of morbidity related to medical treatment | Pneumonia | 158 | 4.20 (3.03 -5.81) |
| GSDD | D10→A41 | Benign tumors | Sepsis | 66 | 7.30 (3.77 -14.14) |
| GSDD | D10→E87 | Benign tumors | Other disorders of fluid, electrolyte and acid-base balance | 84 | 6.79 (3.87 -11.89) |
| GSDD | D10→J12 | Benign tumors | Pneumonia | 73 | 5.53 (3.19 -9.59) |
| GSDD | D10→N17 | Benign tumors | Acute renal failure | 99 | 6.75 (3.99 -11.41) |
| GSDD | D50→A41 | Iron deficiency anaemia | Sepsis | 83 | 10.87 (5.27 -22.43) |
| GSDD | D50→E87 | Iron deficiency anaemia | Other disorders of fluid, electrolyte and acid-base balance | 101 | 10.70 (5.60 -20.46) |
| GSDD | D50→J12 | Iron deficiency anaemia | Pneumonia | 76 | 12.00 (5.55 -25.94) |
| GSDD | E10→A41 | Diabetes | Sepsis | 105 | 2.79 (1.94 -4.03) |
| GSDD | E10→B96 | Diabetes | Bacterial infectious agents | 75 | 3.25 (2.06 -5.14) |
| GSDD | E10→E78 | Diabetes | Disorders of lipoprotein metabolism and other lipidaemias | 62 | 6.36 (3.37 -12.02) |
| GSDD | E10→E87 | Diabetes | Other disorders of fluid, electrolyte and acid-base balance | 144 | 3.70 (2.64 -5.18) |
| GSDD | E10→I48 | Diabetes | Atrial fibrillation and flutter | 78 | 2.93 (1.94 -4.44) |
| GSDD | E10→I51 | Diabetes | Complications and ill-defined descriptions of heart disease | 61 | 3.42 (2.05 -5.70) |
| GSDD | E10→J12 | Diabetes | Pneumonia | 117 | 4.48 (3.00 -6.70) |
| GSDD | E10→K59 | Diabetes | Other functional intestinal disorders | 63 | 3.14 (1.94 -5.07) |
| GSDD | E10→N17 | Diabetes | Acute renal failure | 156 | 3.80 (2.75 -5.26) |
| GSDD | E10→N39 | Diabetes | Disorders of urinary system, possibly infection | 82 | 3.41 (2.22 -5.23) |
| GSDD | E10→T80 | Diabetes | Complications due to medical treatment | 61 | 2.46 (1.56 -3.88) |
| GSDD | E10→Y40 | Diabetes | External causes of morbidity related to medical treatment | 76 | 2.86 (1.88 -4.37) |
| GSDD | E66→A41 | Obesity | Sepsis | 90 | 5.32 (3.26 -8.68) |
| GSDD | E66→B96 | Obesity | Bacterial infectious agents | 72 | 4.41 (2.61 -7.47) |
| GSDD | E66→E87 | Obesity | Other disorders of fluid, electrolyte and acid-base balance | 112 | 4.76 (3.09 -7.33) |
| GSDD | E66→J12 | Obesity | Pneumonia | 103 | 7.73 (4.52 -13.24) |
| GSDD | E66→N17 | Obesity | Acute renal failure | 117 | 9.00 (5.28 -15.34) |
| GSDD | E66→N39 | Obesity | Disorders of urinary system, possibly infection | 67 | 5.00 (2.82 -8.88) |
| GSDD | E78→A09 | Disorders of lipoprotein metabolism and other lipidaemias | Infectious gastroenteritis and colitis | 76 | 2.17 (1.46 -3.22) |
| GSDD | E78→A41 | Disorders of lipoprotein metabolism and other lipidaemias | Sepsis | 112 | 2.91 (2.07 -4.10) |
| GSDD | E78→B96 | Disorders of lipoprotein metabolism and other lipidaemias | Bacterial infectious agents | 103 | 2.64 (1.85 -3.77) |
| GSDD | E78→E87 | Disorders of lipoprotein metabolism and other lipidaemias | Other disorders of fluid, electrolyte and acid-base balance | 169 | 3.25 (2.43 -4.34) |
| GSDD | E78→F05 | Disorders of lipoprotein metabolism and other lipidaemias | Delirium due to known physiological condition | 63 | 2.17 (1.41 -3.34) |
| GSDD | E78→I48 | Disorders of lipoprotein metabolism and other lipidaemias | Atrial fibrillation and flutter | 89 | 3.66 (2.42 -5.51) |
| GSDD | E78→I51 | Disorders of lipoprotein metabolism and other lipidaemias | Complications and ill-defined descriptions of heart disease | 71 | 2.78 (1.79 -4.31) |
| GSDD | E78→J12 | Disorders of lipoprotein metabolism and other lipidaemias | Pneumonia | 129 | 4.03 (2.82 -5.74) |
| GSDD | E78→J20 | Disorders of lipoprotein metabolism and other lipidaemias | Other acute Lower respiratory infections | 62 | 3.53 (2.12 -5.87) |
| GSDD | E78→K59 | Disorders of lipoprotein metabolism and other lipidaemias | Other functional intestinal disorders | 69 | 2.48 (1.61 -3.82) |
| GSDD | E78→N17 | Disorders of lipoprotein metabolism and other lipidaemias | Acute renal failure | 183 | 3.42 (2.58 -4.54) |
| GSDD | E78→N39 | Disorders of lipoprotein metabolism and other lipidaemias | Disorders of urinary system, possibly infection | 92 | 2.65 (1.81 -3.87) |
| GSDD | E78→T80 | Disorders of lipoprotein metabolism and other lipidaemias | Complications due to medical treatment | 80 | 2.61 (1.74 -3.89) |
| GSDD | E78→Y40 | Disorders of lipoprotein metabolism and other lipidaemias | External causes of morbidity related to medical treatment | 90 | 4.42 (2.84 -6.88) |
| GSDD | F17→E87 | Tobacco abuse | Other disorders of fluid, electrolyte and acid-base balance | 70 | 6.08 (3.30 -11.20) |
| GSDD | F17→N17 | Tobacco abuse | Acute renal failure | 83 | 7.08 (3.96 -12.65) |
| GSDD | H25→A41 | Disorders of the lens | Sepsis | 72 | 7.18 (3.82 -13.49) |
| GSDD | H25→E87 | Disorders of the lens | Other disorders of fluid, electrolyte and acid-base balance | 114 | 7.24 (4.36 -12.01) |
| GSDD | H25→I48 | Disorders of the lens | Atrial fibrillation and flutter | 69 | 6.33 (3.45 -11.64) |
| GSDD | H25→J12 | Disorders of the lens | Pneumonia | 96 | 18.17 (7.99 -41.33) |
| GSDD | H25→N17 | Disorders of the lens | Acute renal failure | 137 | 8.65 (5.23 -14.29) |
| GSDD | H25→N39 | Disorders of the lens | Disorders of urinary system, possibly infection | 65 | 8.88 (4.27 -18.43) |
| GSDD | H30→E87 | Disorders of choroid and retina | Other disorders of fluid, electrolyte and acid-base balance | 64 | 16.00 (5.83 -43.94) |
| GSDD | I10→A41 | Primary hypertension | Sepsis | 175 | 1.51 (1.23 -1.86) |
| GSDD | I10→E78 | Primary hypertension | Disorders of lipoprotein metabolism and other lipidaemias | 96 | 2.19 (1.61 -2.99) |
| GSDD | I10→E87 | Primary hypertension | Other disorders of fluid, electrolyte and acid-base balance | 234 | 1.80 (1.48 -2.18) |
| GSDD | I10→I25 | Primary hypertension | Chronic ischaemic heart disease | 75 | 1.84 (1.33 -2.55) |
| GSDD | I10→I48 | Primary hypertension | Atrial fibrillation and flutter | 140 | 1.70 (1.34 -2.17) |
| GSDD | I10→J12 | Primary hypertension | Pneumonia | 198 | 2.38 (1.92 -2.96) |
| GSDD | I10→N17 | Primary hypertension | Acute renal failure | 273 | 1.96 (1.64 -2.34) |
| GSDD | I10→S00 | Primary hypertension | Injuries due to external causes | 81 | 1.81 (1.31 -2.49) |
| GSDD | I10→Y40 | Primary hypertension | External causes of morbidity related to medical treatment | 130 | 1.91 (1.48 -2.47) |
| GSDD | I20→E87 | Angina pectoris | Other disorders of fluid, electrolyte and acid-base balance | 88 | 4.84 (2.95 -7.93) |
| GSDD | I20→J12 | Angina pectoris | Pneumonia | 61 | 6.09 (3.22 -11.52) |
| GSDD | I20→N17 | Angina pectoris | Acute renal failure | 81 | 4.83 (2.91 -8.03) |
| GSDD | I25→A41 | Chronic ischaemic heart disease | Sepsis | 89 | 4.45 (2.81 -7.07) |
| GSDD | I25→B96 | Chronic ischaemic heart disease | Bacterial infectious agents | 77 | 2.90 (1.90 -4.42) |
| GSDD | I25→E87 | Chronic ischaemic heart disease | Other disorders of fluid, electrolyte and acid-base balance | 115 | 4.86 (3.23 -7.30) |
| GSDD | I25→I51 | Chronic ischaemic heart disease | Complications and ill-defined descriptions of heart disease | 63 | 2.68 (1.69 -4.24) |
| GSDD | I25→J12 | Chronic ischaemic heart disease | Pneumonia | 105 | 5.76 (3.63 -9.16) |
| GSDD | I25→N17 | Chronic ischaemic heart disease | Acute renal failure | 137 | 4.40 (3.05 -6.35) |
| GSDD | I25→N39 | Chronic ischaemic heart disease | Disorders of urinary system, possibly infection | 66 | 4.11 (2.46 -6.88) |
| GSDD | I25→T80 | Chronic ischaemic heart disease | Complications due to medical treatment | 62 | 3.05 (1.88 -4.93) |
| GSDD | I48→A09 | Atrial fibrillation and flutter | Infectious gastroenteritis and colitis | 65 | 2.84 (1.80 -4.48) |
| GSDD | I48→A41 | Atrial fibrillation and flutter | Sepsis | 124 | 3.74 (2.63 -5.33) |
| GSDD | I48→B96 | Atrial fibrillation and flutter | Bacterial infectious agents | 91 | 3.55 (2.35 -5.36) |
| GSDD | I48→E87 | Atrial fibrillation and flutter | Other disorders of fluid, electrolyte and acid-base balance | 153 | 4.00 (2.89 -5.55) |
| GSDD | I48→F05 | Atrial fibrillation and flutter | Delirium due to known physiological condition | 65 | 2.52 (1.61 -3.93) |
| GSDD | I48→I05 | Atrial fibrillation and flutter | Chronic rheumatic heart disease | 62 | 2.14 (1.38 -3.32) |
| GSDD | I48→I51 | Atrial fibrillation and flutter | Complications and ill-defined descriptions of heart disease | 88 | 3.62 (2.34 -5.58) |
| GSDD | I48→J12 | Atrial fibrillation and flutter | Pneumonia | 133 | 4.85 (3.35 -7.02) |
| GSDD | I48→K59 | Atrial fibrillation and flutter | Other functional intestinal disorders | 67 | 3.80 (2.32 -6.22) |
| GSDD | I48→S00 | Atrial fibrillation and flutter | Injuries due to external causes | 76 | 3.91 (2.45 -6.24) |
| GSDD | I49→A41 | Other cardiac arrhythmias | Sepsis | 83 | 5.00 (3.01 -8.29) |
| GSDD | I49→E87 | Other cardiac arrhythmias | Other disorders of fluid, electrolyte and acid-base balance | 103 | 5.79 (3.56 -9.42) |
| GSDD | I51→A41 | Complications and ill-defined descriptions of heart disease | Sepsis | 61 | 9.43 (4.33 -20.55) |
| GSDD | J12→A41 | Pneumonia | Sepsis | 119 | 2.80 (2.05 -3.82) |
| GSDD | J44→A41 | Chronic obstructive pulmonary disease | Sepsis | 67 | 6.08 (3.38 -10.93) |
| GSDD | J44→E87 | Chronic obstructive pulmonary disease | Other disorders of fluid, electrolyte and acid-base balance | 88 | 5.56 (3.36 -9.18) |
| GSDD | J44→N17 | Chronic obstructive pulmonary disease | Acute renal failure | 98 | 7.93 (4.55 -13.82) |
| GSDD | K29→A41 | Gastritis and duodenitis | Sepsis | 65 | 6.60 (3.39 -12.83) |
| GSDD | K29→J12 | Gastritis and duodenitis | Pneumonia | 63 | 8.22 (4.12 -16.42) |
| GSDD | K59→A41 | Other functional intestinal disorders | Sepsis | 85 | 5.17 (3.12 -8.56) |
| GSDD | K63→N17 | Other diseases of intestine | Acute renal failure | 64 | 5.83 (3.16 -10.76) |
| GSDD | L01→A41 | Skin and subcutaneous infections | Sepsis | 74 | 8.78 (4.40 -17.49) |
| GSDD | M10→A41 | Other inflammatory arthritis | Sepsis | 96 | 6.62 (3.92 -11.21) |
| GSDD | M10→B96 | Other inflammatory arthritis | Bacterial infectious agents | 69 | 6.00 (3.26 -11.06) |
| GSDD | M10→E87 | Other inflammatory arthritis | Other disorders of fluid, electrolyte and acid-base balance | 120 | 7.33 (4.48 -12.00) |
| GSDD | M10→J12 | Other inflammatory arthritis | Pneumonia | 109 | 9.31 (5.25 -16.49) |
| GSDD | M10→N17 | Other inflammatory arthritis | Acute renal failure | 132 | 9.53 (5.60 -16.23) |
| GSDD | M10→S00 | Other inflammatory arthritis | Injuries due to external causes | 64 | 8.50 (4.09 -17.68) |
| GSDD | M15→A41 | Osteoarthritis | Sepsis | 93 | 3.60 (2.40 -5.39) |
| GSDD | M15→B96 | Osteoarthritis | Bacterial infectious agents | 67 | 4.17 (2.49 -6.97) |
| GSDD | M15→E87 | Osteoarthritis | Other disorders of fluid, electrolyte and acid-base balance | 110 | 4.71 (3.14 -7.09) |
| GSDD | M15→J12 | Osteoarthritis | Pneumonia | 106 | 4.85 (3.18 -7.39) |
| GSDD | M15→N17 | Osteoarthritis | Acute renal failure | 132 | 4.67 (3.20 -6.80) |
| GSDD | M15→W00 | Osteoarthritis | Falls | 62 | 3.67 (2.18 -6.17) |
| GSDD | M20→E87 | Other joint disorders | Other disorders of fluid, electrolyte and acid-base balance | 66 | 13.80 (5.57 -34.21) |
| GSDD | M20→N17 | Other joint disorders | Acute renal failure | 74 | 16.20 (6.57 -39.97) |
| GSDD | M79→E87 | Other soft tissue disorders, not elsewhere classified | Other disorders of fluid, electrolyte and acid-base balance | 69 | 15.00 (6.07 -37.09) |
| GSDD | N17→A41 | Acute renal failure | Sepsis | 191 | 2.85 (2.22 -3.67) |
| GSDD | N17→E87 | Acute renal failure | Other disorders of fluid, electrolyte and acid-base balance | 214 | 4.70 (3.51 -6.31) |
| GSDD | N17→F05 | Acute renal failure | Delirium due to known physiological condition | 88 | 1.96 (1.38 -2.77) |
| GSDD | N17→J12 | Acute renal failure | Pneumonia | 176 | 5.30 (3.83 -7.34) |
| GSDD | N39→A41 | Disorders of urinary system, possibly infection | Sepsis | 108 | 2.74 (1.95 -3.84) |
| GSDD | S00→A41 | Injuries due to external causes | Sepsis | 90 | 3.12 (2.11 -4.62) |
| GSDD | T80→A41 | Complications due to medical treatment | Sepsis | 106 | 3.93 (2.63 -5.87) |
| GSDD | W00→A41 | Falls | Sepsis | 84 | 3.03 (2.02 -4.55) |
| GSDD | Y40→A09 | External causes of morbidity related to medical treatment | Infectious gastroenteritis and colitis | 87 | 2.12 (1.47 -3.04) |
| GSDD | Y40→A41 | External causes of morbidity related to medical treatment | Sepsis | 133 | 3.30 (2.38 -4.57) |
| GSDD | Y40→E87 | External causes of morbidity related to medical treatment | Other disorders of fluid, electrolyte and acid-base balance | 138 | 4.44 (3.10 -6.38) |
| GSDD | Y40→J12 | External causes of morbidity related to medical treatment | Pneumonia | 125 | 4.48 (3.08 -6.54) |
| GSDD | Y40→K59 | External causes of morbidity related to medical treatment | Other functional intestinal disorders | 73 | 2.47 (1.64 -3.72) |
| MND | I10→A41 | Primary hypertension | Sepsis | 636 | 1.40 (1.25 -1.57) |
| MND | I10→D51 | Primary hypertension | Other anaemias | 610 | 1.49 (1.32 -1.68) |
| MND | I10→E87 | Primary hypertension | Other disorders of fluid, electrolyte and acid-base balance | 663 | 1.82 (1.62 -2.04) |
| MND | I10→J12 | Primary hypertension | Pneumonia | 784 | 1.83 (1.64 -2.04) |
| MND | I10→J90 | Primary hypertension | Pleural effusion | 553 | 1.31 (1.16 -1.48) |
| MND | I10→K59 | Primary hypertension | Other functional intestinal disorders | 572 | 1.35 (1.20 -1.52) |
| MND | I10→N17 | Primary hypertension | Acute renal failure | 749 | 1.90 (1.70 -2.12) |
| MND | I10→N39 | Primary hypertension | Disorders of urinary system, possibly infection | 475 | 1.38 (1.21 -1.57) |
| MND | I10→T80 | Primary hypertension | Complications due to medical treatment | 472 | 1.30 (1.14 -1.48) |
| MND | I10→Y40 | Primary hypertension | External causes of morbidity related to medical treatment | 761 | 1.57 (1.41 -1.75) |
| MND | Y40→A41 | External causes of morbidity related to medical treatment | Sepsis | 416 | 2.98 (2.47 -3.59) |
| MND | Y40→E87 | External causes of morbidity related to medical treatment | Other disorders of fluid, electrolyte and acid-base balance | 385 | 3.25 (2.67 -3.97) |
| MND | Y40→J12 | External causes of morbidity related to medical treatment | Pneumonia | 459 | 3.20 (2.67 -3.82) |
| MND | Y40→N17 | External causes of morbidity related to medical treatment | Acute renal failure | 412 | 3.87 (3.16 -4.74) |
| RSDD | B96→J12 | Bacterial infectious agents | Pneumonia | 248 | 4.47 (3.39 -5.88) |
| RSDD | C77→J12 | Metastatic cancer | Pneumonia | 231 | 2.23 (1.79 -2.76) |
| RSDD | D51→E87 | Other anaemias | Other disorders of fluid, electrolyte and acid-base balance | 213 | 3.38 (2.58 -4.42) |
| RSDD | D51→J12 | Other anaemias | Pneumonia | 312 | 3.93 (3.11 -4.97) |
| RSDD | E10→A41 | Diabetes | Sepsis | 181 | 3.25 (2.43 -4.34) |
| RSDD | E10→E87 | Diabetes | Other disorders of fluid, electrolyte and acid-base balance | 241 | 3.47 (2.70 -4.47) |
| RSDD | E10→J12 | Diabetes | Pneumonia | 386 | 3.34 (2.74 -4.07) |
| RSDD | E10→N17 | Diabetes | Acute renal failure | 270 | 3.55 (2.79 -4.51) |
| RSDD | E66→E87 | Obesity | Other disorders of fluid, electrolyte and acid-base balance | 189 | 5.49 (3.86 -7.79) |
| RSDD | E66→J12 | Obesity | Pneumonia | 288 | 6.87 (5.04 -9.36) |
| RSDD | E66→N17 | Obesity | Acute renal failure | 218 | 7.44 (5.14 -10.76) |
| RSDD | E78→A41 | Disorders of lipoprotein metabolism and other lipidaemias | Sepsis | 204 | 2.50 (1.95 -3.20) |
| RSDD | E78→E87 | Disorders of lipoprotein metabolism and other lipidaemias | Other disorders of fluid, electrolyte and acid-base balance | 296 | 3.26 (2.61 -4.08) |
| RSDD | E78→J12 | Disorders of lipoprotein metabolism and other lipidaemias | Pneumonia | 432 | 3.70 (3.04 -4.49) |
| RSDD | E78→N17 | Disorders of lipoprotein metabolism and other lipidaemias | Acute renal failure | 293 | 3.46 (2.76 -4.34) |
| RSDD | F17→J12 | Tobacco abuse | Pneumonia | 227 | 7.09 (4.95 -10.15) |
| RSDD | F32→J12 | Depression | Pneumonia | 192 | 9.18 (5.91 -14.26) |
| RSDD | I10→A41 | Primary hypertension | Sepsis | 349 | 1.56 (1.34 -1.82) |
| RSDD | I10→C77 | Primary hypertension | Metastatic cancer | 199 | 2.12 (1.71 -2.63) |
| RSDD | I10→D51 | Primary hypertension | Other anaemias | 240 | 1.66 (1.37 -2.00) |
| RSDD | I10→E78 | Primary hypertension | Disorders of lipoprotein metabolism and other lipidaemias | 253 | 2.15 (1.77 -2.61) |
| RSDD | I10→E87 | Primary hypertension | Other disorders of fluid, electrolyte and acid-base balance | 456 | 1.72 (1.50 -1.97) |
| RSDD | I10→I25 | Primary hypertension | Chronic ischaemic heart disease | 203 | 1.70 (1.38 -2.08) |
| RSDD | I10→I48 | Primary hypertension | Atrial fibrillation and flutter | 300 | 1.50 (1.27 -1.77) |
| RSDD | I10→J12 | Primary hypertension | Pneumonia | 755 | 1.86 (1.66 -2.07) |
| RSDD | I10→K59 | Primary hypertension | Other functional intestinal disorders | 298 | 1.38 (1.17 -1.62) |
| RSDD | I10→N17 | Primary hypertension | Acute renal failure | 476 | 1.86 (1.62 -2.13) |
| RSDD | I10→N39 | Primary hypertension | Disorders of urinary system, possibly infection | 262 | 1.49 (1.25 -1.78) |
| RSDD | I10→Y40 | Primary hypertension | External causes of morbidity related to medical treatment | 286 | 1.59 (1.34 -1.88) |
| RSDD | I20→J12 | Angina pectoris | Pneumonia | 196 | 7.21 (4.86 -10.71) |
| RSDD | I25→E87 | Chronic ischaemic heart disease | Other disorders of fluid, electrolyte and acid-base balance | 198 | 4.74 (3.46 -6.50) |
| RSDD | I25→J12 | Chronic ischaemic heart disease | Pneumonia | 304 | 4.97 (3.82 -6.46) |
| RSDD | I25→N17 | Chronic ischaemic heart disease | Acute renal failure | 213 | 4.45 (3.31 -6.00) |
| RSDD | I48→E87 | Atrial fibrillation and flutter | Other disorders of fluid, electrolyte and acid-base balance | 250 | 4.13 (3.17 -5.39) |
| RSDD | I48→I50 | Atrial fibrillation and flutter | Heart failure | 219 | 3.92 (2.98 -5.16) |
| RSDD | I48→J12 | Atrial fibrillation and flutter | Pneumonia | 347 | 4.70 (3.71 -5.95) |
| RSDD | I48→N17 | Atrial fibrillation and flutter | Acute renal failure | 249 | 4.73 (3.58 -6.25) |
| RSDD | I49→J12 | Other cardiac arrhythmias | Pneumonia | 186 | 8.50 (5.57 -12.97) |
| RSDD | I50→E87 | Heart failure | Other disorders of fluid, electrolyte and acid-base balance | 216 | 6.39 (4.54 -9.00) |
| RSDD | I50→N17 | Heart failure | Acute renal failure | 242 | 6.30 (4.58 -8.65) |
| RSDD | J20→J12 | Other acute Lower respiratory infections | Pneumonia | 254 | 7.00 (5.03 -9.75) |
| RSDD | J44→A41 | Chronic obstructive pulmonary disease | Sepsis | 188 | 4.23 (3.09 -5.79) |
| RSDD | J44→E87 | Chronic obstructive pulmonary disease | Other disorders of fluid, electrolyte and acid-base balance | 262 | 4.51 (3.43 -5.92) |
| RSDD | J44→I50 | Chronic obstructive pulmonary disease | Heart failure | 194 | 3.87 (2.87 -5.22) |
| RSDD | J44→J12 | Chronic obstructive pulmonary disease | Pneumonia | 382 | 7.67 (5.77 -10.18) |
| RSDD | J44→N17 | Chronic obstructive pulmonary disease | Acute renal failure | 262 | 7.10 (5.12 -9.84) |
| RSDD | J45→J12 | Asthma | Pneumonia | 216 | 25.11 (12.90 -48.89) |
| RSDD | K21→J12 | Gastro-oesophageal reflux disease | Pneumonia | 225 | 7.15 (4.97 -10.29) |
| RSDD | K29→J12 | Gastritis and duodenitis | Pneumonia | 234 | 6.35 (4.55 -8.86) |
| RSDD | K44→J12 | Diaphragmatic hernia | Pneumonia | 215 | 11.84 (7.41 -18.91) |
| RSDD | M10→J12 | Other inflammatory arthritis | Pneumonia | 286 | 7.60 (5.47 -10.57) |
| RSDD | M20→J12 | Other joint disorders | Pneumonia | 187 | 9.00 (5.79 -13.98) |
| RSDD | M54→J12 | Dorsalgia | Pneumonia | 188 | 10.37 (6.47 -16.60) |
| RSDD | N17→A41 | Acute renal failure | Sepsis | 215 | 3.27 (2.54 -4.22) |
| RSDD | N17→E87 | Acute renal failure | Other disorders of fluid, electrolyte and acid-base balance | 245 | 4.32 (3.32 -5.63) |
| RSDD | N18→E87 | Chronic kidney disease | Other disorders of fluid, electrolyte and acid-base balance | 211 | 5.80 (4.15 -8.11) |
| RSDD | N18→J12 | Chronic kidney disease | Pneumonia | 311 | 6.74 (5.01 -9.07) |
| RSDD | N39→A41 | Disorders of urinary system, possibly infection | Sepsis | 182 | 3.73 (2.77 -5.02) |
| RSDD | N39→J12 | Disorders of urinary system, possibly infection | Pneumonia | 280 | 3.91 (3.06 -5.00) |
| RSDD | S00→A41 | Injuries due to external causes | Sepsis | 183 | 3.21 (2.42 -4.25) |
| RSDD | S00→E87 | Injuries due to external causes | Other disorders of fluid, electrolyte and acid-base balance | 201 | 5.21 (3.75 -7.25) |
| RSDD | S00→J12 | Injuries due to external causes | Pneumonia | 330 | 5.28 (4.07 -6.84) |
| RSDD | S00→N17 | Injuries due to external causes | Acute renal failure | 221 | 4.84 (3.57 -6.56) |
| RSDD | T80→J12 | Complications due to medical treatment | Pneumonia | 229 | 4.73 (3.51 -6.38) |
| RSDD | W00→J12 | Falls | Pneumonia | 282 | 4.94 (3.76 -6.48) |
| RSDD | Y40→A41 | External causes of morbidity related to medical treatment | Sepsis | 187 | 3.37 (2.54 -4.48) |
| RSDD | Y40→E87 | External causes of morbidity related to medical treatment | Other disorders of fluid, electrolyte and acid-base balance | 214 | 4.42 (3.30 -5.92) |
| RSDD | Y40→J12 | External causes of morbidity related to medical treatment | Pneumonia | 373 | 3.94 (3.20 -4.87) |
| RSDD | Y40→N17 | External causes of morbidity related to medical treatment | Acute renal failure | 221 | 4.94 (3.67 -6.66) |
| UCD | D51→A41 | Other anaemias | Sepsis | 58 | 3.76 (2.21 -6.43) |
| UCD | D51→B96 | Other anaemias | Bacterial infectious agents | 55 | 5.00 (2.69 -9.29) |
| UCD | E10→N17 | Diabetes | Acute renal failure | 55 | 3.71 (2.17 -6.33) |
| UCD | I25→Y40 | Chronic ischaemic heart disease | External causes of morbidity related to medical treatment | 58 | 3.19 (1.95 -5.21) |
| UCD | T80→A41 | Complications due to medical treatment | Sepsis | 59 | 5.23 (2.89 -9.47) |

^*^Combined ICD-10 codes derived from the original ICD-10 code and are displayed in the Additional file 1.

^#^Number of the participants experienced the corresponding temporal disease trajectories leading to causes of death.

OR, odds ratio; CI, confidence interval. MAFLD, metabolic dysfunction-associated fatty liver disease; MND, malignant neoplasms death; ESDD, endocrine system disease death; CVDD, cardiovascular disease death; RSDD, respiratory system disease death; DSSD, digestive system disease death; GSDD, genitourinary system disease death; UCD, unnatural cause death.

Additional file 1: Table S6. PheWAS using Cox regression was conducted to investigate the relationship between MAFLD and 490 subsequent disease conditions in heavy drinkers and nonheavy drinkers.

| **Medical conditions** | **Code^*^** | **Total**  **(N=****326,606)** | | **Non-heavy drinker (N=256,902)** | | **Heavy drinker**  **(N=69,704)** | |
| --- | --- | --- | --- | --- | --- | --- | --- |
|  |  | **No.^#^** | **HR (95% CI)** | **No.^#^** | **HR (95% CI)** | **No.^#^** | **HR (95% CI)** |
| **Infectious and parasitic** |  |  |  |  |  |  |  |
| Infectious gastroenteritis and colitis | A09 | 7336 | 1.40 (1.35-1.45) | 5856 | 1.47 (1.42-1.53) | 1480 | 1.18 (1.09-1.28) |
| Sepsis | A41 | 5918 | 1.51 (1.45-1.58) | 4580 | 1.57 (1.50-1.64) | 1338 | 1.34 (1.23-1.46) |
| Mycoses | B49 | 2527 | 1.29 (1.21-1.36) | 1946 | 1.33 (1.25-1.42) | 581 | 1.12 (0.99-1.27) |
| Bacterial infectious agents | B96 | 8188 | 1.49 (1.44-1.54) | 6389 | 1.55 (1.49-1.61) | 1799 | 1.28 (1.19-1.38) |
| **Malignant neoplasms** |  |  |  |  |  |  |  |
| Colon cancer | C18 | 1920 | 1.27 (1.18-1.35) | 1382 | 1.25 (1.15-1.35) | 538 | 1.27 (1.10-1.45) |
| Metastatic cancer | C77 | 7220 | 1.21 (1.17-1.26) | 5348 | 1.22 (1.18-1.27) | 1872 | 1.14 (1.06-1.22) |
| **Benign neoplasms** |  |  |  |  |  |  |  |
| Benign tumors | D10 | 17866 | 1.29 (1.26-1.32) | 13301 | 1.30 (1.27-1.34) | 4565 | 1.20 (1.15-1.26) |
| **Blood system** |  |  |  |  |  |  |  |
| Iron deficiency anaemia | D50 | 6817 | 1.38 (1.33-1.43) | 5654 | 1.45 (1.40-1.51) | 1163 | 1.15 (1.05-1.26) |
| Other anaemias | D51 | 9594 | 1.31 (1.27-1.35) | 7570 | 1.36 (1.32-1.41) | 2024 | 1.15 (1.08-1.24) |
| **Endocrine system disease** |  |  |  |  |  |  |  |
| Hypothyroid conditions | E00 | 7738 | 1.47 (1.42-1.52) | 6648 | 1.57 (1.51-1.63) | 1090 | 1.18 (1.08-1.30) |
| Diabetes | E10 | 19609 | 4.24 (4.11-4.37) | 15832 | 4.33 (4.19-4.48) | 3777 | 4.20 (3.88-4.54) |
| Malnutrition | E40 | 3322 | 1.42 (1.35-1.50) | 2679 | 1.52 (1.43-1.61) | 643 | 1.11 (0.99-1.25) |
| Obesity | E66 | 21512 | 8.60 (8.26-8.95) | 17194 | 8.96 (8.58-9.37) | 4318 | 7.54 (6.85-8.28) |
| Disorders of lipoprotein metabolism and other lipidaemias | E78 | 26665 | 1.77 (1.74-1.81) | 20093 | 1.78 (1.74-1.82) | 6572 | 1.74 (1.67-1.82) |
| Disorders of mineral metabolism | E83 | 2984 | 1.47 (1.39-1.55) | 2209 | 1.48 (1.39-1.58) | 775 | 1.39 (1.23-1.56) |
| Volume depletion | E86 | 3874 | 1.43 (1.36-1.50) | 2998 | 1.46 (1.38-1.54) | 876 | 1.34 (1.20-1.49) |
| Other disorders of fluid, electrolyte and acid-base balance | E87 | 8207 | 1.43 (1.38-1.48) | 6301 | 1.49 (1.43-1.55) | 1906 | 1.23 (1.14-1.32) |
| **Mental disorder** |  |  |  |  |  |  |  |
| Delirium due to known physiological condition | F05 | 2535 | 1.39 (1.31-1.47) | 1937 | 1.41 (1.31-1.50) | 598 | 1.31 (1.15-1.50) |
| Alcohol abuse | F10 | 3267 | 1.55 (1.47-1.64) | 1124 | 1.37 (1.25-1.50) | 2143 | 1.35 (1.26-1.45) |
| Tobacco abuse | F17 | 9131 | 1.24 (1.20-1.28) | 6230 | 1.32 (1.28-1.37) | 2901 | 0.96 (0.91-1.01) |
| Depression | F32 | 9717 | 1.63 (1.58-1.68) | 7783 | 1.72 (1.66-1.78) | 1934 | 1.35 (1.25-1.45) |
| Anxiety | F40 | 7324 | 1.37 (1.32-1.42) | 5857 | 1.42 (1.36-1.47) | 1467 | 1.24 (1.14-1.35) |
| **Neural system disease** |  |  |  |  |  |  |  |
| Epilepsia | G40 | 1685 | 1.26 (1.18-1.36) | 1309 | 1.29 (1.19-1.40) | 376 | 1.19 (1.01-1.40) |
| Sleep disorder | G47 | 5120 | 3.71 (3.50-3.93) | 4026 | 3.88 (3.63-4.14) | 1094 | 3.16 (2.77-3.61) |
| Diseases in nerves, nerve roots and nerve plexa | G50 | 7795 | 1.59 (1.54-1.65) | 6099 | 1.63 (1.57-1.70) | 1696 | 1.46 (1.35-1.59) |
| Polyneuropathies | G60 | 2257 | 1.96 (1.83-2.10) | 1751 | 2.02 (1.86-2.18) | 506 | 1.77 (1.51-2.07) |
| Cerebral palsy and other paralytic syndromes | G80 | 1764 | 1.42 (1.32-1.52) | 1338 | 1.47 (1.35-1.59) | 426 | 1.22 (1.05-1.43) |
| **Eye and adnexa disease** |  |  |  |  |  |  |  |
| Disorder of eyelid | H02 | 2477 | 1.27 (1.19-1.34) | 1909 | 1.30 (1.22-1.39) | 568 | 1.13 (0.99-1.28) |
| Disorders of the lens | H25 | 16810 | 1.12 (1.10-1.15) | 13114 | 1.14 (1.11-1.17) | 3696 | 1.07 (1.02-1.13) |
| Disorders of choroid and retina | H30 | 5829 | 1.29 (1.24-1.34) | 4585 | 1.34 (1.29-1.40) | 1244 | 1.11 (1.02-1.22) |
| Visual disturbances and blindness | H53 | 3028 | 1.27 (1.20-1.34) | 2371 | 1.29 (1.21-1.37) | 657 | 1.22 (1.08-1.38) |
| **Ear dieases** |  |  |  |  |  |  |  |
| Other disorders of the ear | H90 | 4669 | 1.18 (1.13-1.23) | 3639 | 1.18 (1.13-1.24) | 1030 | 1.20 (1.09-1.32) |
| **Cardiovascular disease** |  |  |  |  |  |  |  |
| Chronic rheumatic heart disease | I05 | 3256 | 1.32 (1.25-1.39) | 2493 | 1.34 (1.27-1.42) | 763 | 1.22 (1.09-1.37) |
| Primary hypertension | I10 | 48720 | 1.93 (1.91-1.96) | 36469 | 1.96 (1.93-1.99) | 12251 | 1.81 (1.76-1.87) |
| Angina pectoris | I20 | 9207 | 1.69 (1.63-1.75) | 7159 | 1.73 (1.67-1.80) | 2048 | 1.56 (1.45-1.68) |
| Acute myocardial infarction | I21 | 5041 | 1.55 (1.48-1.62) | 3877 | 1.55 (1.48-1.63) | 1164 | 1.57 (1.42-1.73) |
| Other ischemic heart disease | I22 | 1760 | 1.57 (1.46-1.70) | 1387 | 1.64 (1.51-1.79) | 373 | 1.35 (1.14-1.59) |
| Chronic ischaemic heart disease | I25 | 15177 | 1.59 (1.55-1.63) | 11613 | 1.62 (1.57-1.67) | 3564 | 1.46 (1.38-1.54) |
| Embolism and thrombosis | I26 | 5561 | 1.48 (1.42-1.55) | 4285 | 1.55 (1.48-1.62) | 1276 | 1.26 (1.15-1.38) |
| Non-rheumatic valve disorders | I34 | 4858 | 1.34 (1.28-1.39) | 3672 | 1.35 (1.28-1.41) | 1186 | 1.28 (1.17-1.41) |
| Atrial fibrillation and flutter | I48 | 12662 | 1.45 (1.41-1.49) | 9248 | 1.45 (1.41-1.50) | 3414 | 1.40 (1.32-1.48) |
| Other cardiac arrhythmias | I49 | 9066 | 1.35 (1.30-1.39) | 6896 | 1.36 (1.32-1.41) | 2170 | 1.27 (1.19-1.36) |
| Heart failure | I50 | 6928 | 1.91 (1.84-1.99) | 5326 | 1.97 (1.88-2.06) | 1602 | 1.71 (1.56-1.86) |
| Complications and ill-defined descriptions of heart disease | I51 | 6566 | 1.70 (1.63-1.77) | 5014 | 1.74 (1.67-1.82) | 1552 | 1.53 (1.41-1.67) |
| Stroke | I60 | 4278 | 1.30 (1.24-1.36) | 3147 | 1.28 (1.22-1.35) | 1131 | 1.31 (1.19-1.44) |
| Other cerebrovascular diseases | I65 | 4012 | 1.37 (1.31-1.44) | 2998 | 1.37 (1.30-1.44) | 1014 | 1.34 (1.21-1.48) |
| Aneurysm and dissection | I71 | 1856 | 1.34 (1.25-1.44) | 1305 | 1.27 (1.17-1.37) | 551 | 1.51 (1.31-1.74) |
| Peripheral vascular disease | I73 | 2716 | 1.23 (1.16-1.30) | 1992 | 1.30 (1.21-1.38) | 724 | 0.98 (0.88-1.10) |
| Varicose veins of lower extremities | I83 | 2465 | 1.19 (1.12-1.26) | 1891 | 1.22 (1.14-1.30) | 574 | 1.09 (0.96-1.24) |
| Hypotension | I95 | 5918 | 1.25 (1.20-1.30) | 4583 | 1.28 (1.23-1.34) | 1335 | 1.13 (1.04-1.23) |
| **Respiratory system disease** |  |  |  |  |  |  |  |
| Pneumonia | J12 | 10625 | 1.46 (1.42-1.50) | 8104 | 1.51 (1.46-1.56) | 2521 | 1.27 (1.20-1.36) |
| Other acute Lower respiratory infections | J20 | 5923 | 1.62 (1.56-1.69) | 4620 | 1.66 (1.59-1.74) | 1303 | 1.48 (1.35-1.63) |
| Chronic obstructive pulmonary disease | J44 | 7598 | 1.43 (1.38-1.48) | 5546 | 1.55 (1.49-1.61) | 2052 | 1.08 (1.01-1.16) |
| Asthma | J45 | 11842 | 1.46 (1.42-1.50) | 9287 | 1.53 (1.48-1.58) | 2555 | 1.24 (1.16-1.32) |
| Pleural effusion | J90 | 5442 | 1.29 (1.24-1.35) | 4053 | 1.30 (1.24-1.36) | 1389 | 1.23 (1.13-1.34) |
| Other diseases of the respiratory system | J95 | 4666 | 1.33 (1.28-1.39) | 3559 | 1.39 (1.32-1.46) | 1107 | 1.14 (1.04-1.25) |
| Respiratory failure | J96 | 3334 | 1.62 (1.53-1.71) | 2550 | 1.76 (1.65-1.88) | 784 | 1.19 (1.07-1.33) |
| **Digestive system disease** |  |  |  |  |  |  |  |
| Disease of hard tissue of teeth | K00 | 1798 | 1.30 (1.21-1.39) | 1448 | 1.36 (1.26-1.47) | 350 | 1.13 (0.96-1.34) |
| Oesophagitis | K20 | 3850 | 1.25 (1.19-1.31) | 2895 | 1.26 (1.20-1.33) | 955 | 1.18 (1.07-1.31) |
| Gastro-oesophageal reflux disease | K21 | 15589 | 1.39 (1.36-1.42) | 12240 | 1.44 (1.40-1.48) | 3349 | 1.25 (1.18-1.32) |
| Other diseases of oesophagus | K22 | 5536 | 1.24 (1.19-1.29) | 4101 | 1.26 (1.20-1.32) | 1435 | 1.13 (1.04-1.22) |
| Gastric ulcer | K25 | 2387 | 1.42 (1.33-1.51) | 1835 | 1.44 (1.34-1.54) | 552 | 1.36 (1.18-1.56) |
| Gastritis and duodenitis | K29 | 13686 | 1.26 (1.23-1.29) | 10590 | 1.29 (1.26-1.33) | 3096 | 1.14 (1.08-1.21) |
| Disease of stomach and duodenum | K31 | 5681 | 1.37 (1.32-1.43) | 4506 | 1.39 (1.33-1.45) | 1175 | 1.34 (1.22-1.47) |
| Umbilical hernia | K42 | 2656 | 2.75 (2.56-2.96) | 1950 | 2.84 (2.61-3.09) | 706 | 2.38 (2.05-2.75) |
| Ventral hernia | K43 | 1994 | 1.99 (1.85-2.15) | 1541 | 2.08 (1.91-2.26) | 453 | 1.70 (1.44-2.00) |
| Diaphragmatic hernia | K44 | 13407 | 1.31 (1.27-1.34) | 10621 | 1.37 (1.33-1.40) | 2786 | 1.13 (1.07-1.20) |
| Noninfective gastroenteritis and colitis | K52 | 5150 | 1.28 (1.23-1.34) | 4077 | 1.33 (1.27-1.39) | 1073 | 1.15 (1.04-1.26) |
| Diverticular disease of intestine | K57 | 20017 | 1.40 (1.37-1.43) | 14911 | 1.41 (1.38-1.45) | 5106 | 1.34 (1.28-1.40) |
| Irritable bowel syndrome | K58 | 3010 | 1.27 (1.21-1.34) | 2534 | 1.35 (1.27-1.43) | 476 | 1.05 (0.91-1.20) |
| Other functional intestinal disorders | K59 | 8857 | 1.23 (1.20-1.27) | 7053 | 1.28 (1.24-1.33) | 1804 | 1.09 (1.02-1.17) |
| Other diseases of anus and rectum | K62 | 8721 | 1.22 (1.18-1.26) | 6395 | 1.22 (1.18-1.26) | 2326 | 1.18 (1.11-1.26) |
| Other diseases of intestine | K63 | 11385 | 1.39 (1.35-1.43) | 8241 | 1.38 (1.33-1.42) | 3144 | 1.35 (1.27-1.43) |
| Haemorrhoids and perianal venous thrombosis | K64 | 6169 | 1.11 (1.08-1.16) | 4762 | 1.14 (1.10-1.19) | 1407 | 1.02 (0.94-1.10) |
| Other disorders of peritoneum | K66 | 2837 | 1.60 (1.51-1.70) | 2291 | 1.71 (1.60-1.82) | 546 | 1.26 (1.10-1.45) |
| Other diseases of liver | K76 | 5167 | 2.67 (2.54-2.81) | 3935 | 2.78 (2.62-2.95) | 1232 | 2.28 (2.04-2.54) |
| Cholelithiasis | K80 | 7589 | 2.19 (2.11-2.28) | 6411 | 2.33 (2.23-2.43) | 1178 | 1.79 (1.62-1.99) |
| Disease of gallbladder and biliary tract | K82 | 2841 | 1.84 (1.73-1.95) | 2288 | 1.94 (1.81-2.08) | 553 | 1.50 (1.31-1.74) |
| Other disease of digestive system | K92 | 6861 | 1.28 (1.24-1.33) | 5200 | 1.29 (1.24-1.34) | 1661 | 1.23 (1.14-1.33) |
| **Skin and subcutaneous disease** |  |  |  |  |  |  |  |
| Skin and subcutaneous infections | L01 | 6610 | 1.93 (1.85-2.01) | 5093 | 2.01 (1.92-2.11) | 1517 | 1.65 (1.51-1.80) |
| Dermatitis | L20 | 2716 | 1.40 (1.32-1.48) | 2100 | 1.46 (1.37-1.56) | 616 | 1.17 (1.03-1.33) |
| Papulosquamous disorders | L40 | 2079 | 1.55 (1.45-1.66) | 1547 | 1.57 (1.45-1.69) | 532 | 1.47 (1.27-1.70) |
| Pressure ulcer | L89 | 1868 | 1.54 (1.43-1.65) | 1450 | 1.64 (1.51-1.78) | 418 | 1.20 (1.03-1.40) |
| Disorder of the skin and subcutaneous tissue | L98 | 2651 | 1.15 (1.09-1.22) | 2030 | 1.18 (1.11-1.25) | 621 | 1.07 (0.95-1.20) |
| **Musculoskeletal and connective tissue disease** |  |  |  |  |  |  |  |
| Autoimmune arthritis | M05 | 3037 | 1.53 (1.45-1.62) | 2463 | 1.62 (1.52-1.72) | 574 | 1.26 (1.10-1.43) |
| Other inflammatory arthritis | M10 | 15579 | 1.92 (1.87-1.98) | 11568 | 1.92 (1.87-1.98) | 4011 | 1.88 (1.77-1.98) |
| Osteoarthritis | M15 | 28395 | 1.64 (1.61-1.67) | 21927 | 1.70 (1.66-1.74) | 6468 | 1.45 (1.39-1.51) |
| Other joint disorders | M20 | 13659 | 1.37 (1.34-1.41) | 10454 | 1.42 (1.38-1.46) | 3205 | 1.21 (1.15-1.28) |
| Systemic connective tissue disorders | M30 | 2191 | 1.26 (1.18-1.34) | 1800 | 1.32 (1.23-1.41) | 391 | 1.07 (0.92-1.25) |
| Deforming dorsopathies | M40 | 2305 | 1.31 (1.23-1.39) | 1850 | 1.37 (1.28-1.46) | 455 | 1.14 (0.99-1.31) |
| Spondylosis | M47 | 6311 | 1.54 (1.48-1.60) | 4922 | 1.58 (1.52-1.66) | 1389 | 1.39 (1.28-1.52) |
| Spondylopathy | M48 | 4091 | 1.67 (1.59-1.76) | 3204 | 1.75 (1.65-1.85) | 887 | 1.43 (1.28-1.59) |
| Other intervertebral disc disorders | M51 | 4502 | 1.58 (1.51-1.66) | 3526 | 1.66 (1.57-1.75) | 976 | 1.32 (1.19-1.47) |
| Dorsalgia | M54 | 8760 | 1.54 (1.49-1.60) | 6896 | 1.60 (1.54-1.66) | 1864 | 1.39 (1.29-1.50) |
| Disorders of synovium and tendons | M65 | 3639 | 1.34 (1.28-1.41) | 2835 | 1.39 (1.31-1.47) | 804 | 1.19 (1.07-1.33) |
| Shoulder lesion | M75 | 4103 | 1.39 (1.33-1.46) | 3152 | 1.44 (1.36-1.52) | 951 | 1.24 (1.12-1.37) |
| Other soft tissue disorders, not elsewhere classified | M79 | 7864 | 1.75 (1.69-1.82) | 6336 | 1.83 (1.76-1.91) | 1528 | 1.52 (1.39-1.65) |
| **Genitourinary system disease** |  |  |  |  |  |  |  |
| Obstructive and reflux uropathy | N13 | 2170 | 1.31 (1.23-1.39) | 1711 | 1.33 (1.24-1.43) | 459 | 1.23 (1.06-1.43) |
| Acute renal failure | N17 | 9834 | 1.90 (1.84-1.96) | 7553 | 1.95 (1.88-2.03) | 2281 | 1.72 (1.60-1.85) |
| Chronic kidney disease | N18 | 9060 | 2.01 (1.94-2.08) | 7547 | 2.11 (2.03-2.19) | 1513 | 1.74 (1.59-1.91) |
| Urolithiasis | N20 | 2924 | 1.53 (1.45-1.62) | 2417 | 1.60 (1.50-1.70) | 507 | 1.37 (1.18-1.58) |
| Other disorders of kidney and ureter | N29 | 2810 | 1.56 (1.47-1.65) | 2252 | 1.63 (1.52-1.74) | 558 | 1.34 (1.17-1.54) |
| Bladder disorder | N32 | 4485 | 1.17 (1.12-1.22) | 3484 | 1.17 (1.12-1.23) | 1001 | 1.21 (1.10-1.34) |
| Disorders of urinary system, possibly infection | N39 | 9880 | 1.49 (1.45-1.54) | 7964 | 1.55 (1.50-1.61) | 1916 | 1.33 (1.23-1.43) |
| Female genital prolapse | N81 | 2547 | 1.21 (1.14-1.28) | 2305 | 1.31 (1.23-1.39) | 242 | 0.87 (0.72-1.05) |
| Polyp of female genital tract | N84 | 1916 | 1.75 (1.63-1.89) | 1672 | 1.86 (1.72-2.01) | 244 | 1.48 (1.19-1.83) |
| Menopausal and perimenopausal disorders | N95 | 1876 | 1.49 (1.39-1.60) | 1632 | 1.59 (1.47-1.71) | 244 | 1.21 (0.99-1.48) |
| **Unnatural cause** |  |  |  |  |  |  |  |
| Injuries due to external causes | S00 | 14888 | 1.06 (1.04-1.09) | 11193 | 1.08 (1.05-1.11) | 3695 | 0.97 (0.92-1.01) |
| Complications due to medical treatment | T80 | 11766 | 1.42 (1.38-1.46) | 9052 | 1.47 (1.43-1.52) | 2714 | 1.24 (1.17-1.32) |
| Falls | W00 | 10293 | 1.14 (1.11-1.17) | 7738 | 1.17 (1.13-1.20) | 2555 | 1.02 (0.96-1.08) |
| Other external causes of accidental injury | W20 | 5349 | 1.17 (1.12-1.22) | 3940 | 1.19 (1.14-1.24) | 1409 | 1.06 (0.98-1.15) |
| External causes of morbidity related to medical treatment | Y40 | 15399 | 1.38 (1.35-1.41) | 11907 | 1.42 (1.38-1.46) | 3492 | 1.26 (1.19-1.33) |
| External causes of morbidity related to other conditions | Y95 | 2479 | 1.46 (1.37-1.55) | 1879 | 1.51 (1.41-1.62) | 600 | 1.28 (1.12-1.45) |

A total of 113 disease conditions were confirmed to be significantly associated with MAFLD after Bonferroni correction. Significantly higher HRs (95%CIs without overlap) were marked in red in non-heavy drinker or heavy drinker (consuming alcohol > 30g/d for male and > 20g/d for female) . The recording of alcohol intake is based on recollection and estimation of the average total amount of red wine, spirits, beer, and fruit wine consumed each week or month.

^*^Combined ICD-10 codes derived from the original ICD-10 code and are displayed in the Additional file 1.

^#^Number of the participants diagnosed with the corresponding diseases.

HR, hazard ratio; CI, confidence interval. MAFLD, Metabolic dysfunction-associated fatty liver disease.

Additional file 1: Table S7. PheWAS using Cox regression was conducted to investigate the relationship between MAFLD and 490 subsequent disease conditions in normal weight and overweight/obese individuals.

| **Medical conditions** | **Code^*^** | **Total**  **(N =** **326,606)** | | **Normal weight**  **(N = 83,508)** | | **Over weight/obesity**  **(N = 243,098)** | |
| --- | --- | --- | --- | --- | --- | --- | --- |
|  |  | **No.^#^** | **HR (95% CI)** | **No.^#^** | **HR (95% CI)** | **No.^#^** | **HR (95% CI)** |
| **Infectious and parasitic** |  |  |  |  |  |  |  |
| Infectious gastroenteritis and colitis | A09 | 7336 | 1.40 (1.35-1.45) | 131 | 1.68 (1.41-2.01) | 7205 | 1.39 (1.33-1.46) |
| Sepsis | A41 | 5918 | 1.51 (1.45-1.58) | 93 | 1.66 (1.35-2.05) | 5825 | 1.46 (1.38-1.53) |
| Mycoses | B49 | 2527 | 1.29 (1.21-1.36) | 42 | 1.35 (0.99-1.84) | 2485 | 1.36 (1.26-1.47) |
| Bacterial infectious agents | B96 | 8188 | 1.49 (1.44-1.54) | 158 | 2.00 (1.70-2.35) | 8030 | 1.43 (1.37-1.50) |
| **Malignant neoplasms** |  |  |  |  |  |  |  |
| Colon cancer | C18 | 1920 | 1.27 (1.18-1.35) | 26 | 1.25 (0.84-1.84) | 1894 | 1.17 (1.08-1.27) |
| Metastatic cancer | C77 | 7220 | 1.21 (1.17-1.26) | 117 | 1.32 (1.09-1.58) | 7103 | 1.21 (1.16-1.26) |
| **Benign neoplasms** |  |  |  |  |  |  |  |
| Benign tumors | D10 | 17866 | 1.29 (1.26-1.32) | 272 | 1.35 (1.19-1.52) | 17594 | 1.22 (1.19-1.26) |
| **Blood system** |  |  |  |  |  |  |  |
| Iron deficiency anaemia | D50 | 6817 | 1.38 (1.33-1.43) | 97 | 1.38 (1.12-1.69) | 6720 | 1.31 (1.25-1.37) |
| Other anaemias | D51 | 9594 | 1.31 (1.27-1.35) | 158 | 1.46 (1.25-1.72) | 9436 | 1.29 (1.25-1.34) |
| **Endocrine system disease** |  |  |  |  |  |  |  |
| Hypothyroid conditions | E00 | 7738 | 1.47 (1.42-1.52) | 70 | 0.97 (0.76-1.23) | 7668 | 1.34 (1.29-1.40) |
| Diabetes | E10 | 19609 | 4.24 (4.11-4.37) | 184 | 3.54 (3.04-4.12) | 19425 | 3.34 (3.21-3.46) |
| Malnutrition | E40 | 3322 | 1.42 (1.35-1.50) | 53 | 1.47 (1.12-1.94) | 3269 | 1.46 (1.37-1.56) |
| Obesity | E66 | 21512 | 8.60 (8.26-8.95) | 17 | 1.93 (1.18-3.15) | 21495 | 4.92 (4.71-5.13) |
| Disorders of lipoprotein metabolism and other lipidaemias | E78 | 26665 | 1.77 (1.74-1.81) | 447 | 2.35 (2.14-2.59) | 26218 | 1.53 (1.50-1.57) |
| Disorders of mineral metabolism | E83 | 2984 | 1.47 (1.39-1.55) | 66 | 2.22 (1.73-2.84) | 2918 | 1.44 (1.34-1.54) |
| Volume depletion | E86 | 3874 | 1.43 (1.36-1.50) | 78 | 1.86 (1.48-2.34) | 3796 | 1.47 (1.38-1.57) |
| Other disorders of fluid, electrolyte and acid-base balance | E87 | 8207 | 1.43 (1.38-1.48) | 176 | 2.02 (1.74-2.35) | 8031 | 1.46 (1.40-1.53) |
| **Mental disorder** |  |  |  |  |  |  |  |
| Delirium due to known physiological condition | F05 | 2535 | 1.39 (1.31-1.47) | 44 | 1.63 (1.20-2.20) | 2491 | 1.38 (1.28-1.49) |
| Alcohol abuse | F10 | 3267 | 1.55 (1.47-1.64) | 145 | 4.22 (3.55-5.01) | 3122 | 1.71 (1.59-1.83) |
| Tobacco abuse | F17 | 9131 | 1.24 (1.20-1.28) | 270 | 2.14 (1.90-2.43) | 8861 | 1.46 (1.40-1.52) |
| Depression | F32 | 9717 | 1.63 (1.58-1.68) | 130 | 1.48 (1.24-1.76) | 9587 | 1.61 (1.54-1.67) |
| Anxiety | F40 | 7324 | 1.37 (1.32-1.42) | 105 | 1.31 (1.08-1.59) | 7219 | 1.38 (1.32-1.44) |
| **Neural system disease** |  |  |  |  |  |  |  |
| Epilepsia | G40 | 1685 | 1.26 (1.18-1.36) | 50 | 2.43 (1.82-3.24) | 1635 | 1.29 (1.18-1.42) |
| Sleep disorder | G47 | 5120 | 3.71 (3.50-3.93) | 40 | 2.71 (1.96-3.74) | 5080 | 2.89 (2.70-3.10) |
| Diseases in nerves, nerve roots and nerve plexa | G50 | 7795 | 1.59 (1.54-1.65) | 83 | 1.29 (1.04-1.61) | 7712 | 1.40 (1.34-1.46) |
| Polyneuropathies | G60 | 2257 | 1.96 (1.83-2.10) | 34 | 2.00 (1.41-2.83) | 2223 | 1.94 (1.77-2.12) |
| Cerebral palsy and other paralytic syndromes | G80 | 1764 | 1.42 (1.32-1.52) | 30 | 1.71 (1.18-2.46) | 1734 | 1.34 (1.23-1.47) |
| **Eye and adnexa disease** |  |  |  |  |  |  |  |
| Disorder of eyelid | H02 | 2477 | 1.27 (1.19-1.34) | 36 | 1.33 (0.96-1.86) | 2441 | 1.18 (1.10-1.26) |
| Disorders of the lens | H25 | 16810 | 1.12 (1.10-1.15) | 253 | 1.20 (1.06-1.37) | 16557 | 1.07 (1.04-1.10) |
| Disorders of choroid and retina | H30 | 5829 | 1.29 (1.24-1.34) | 77 | 1.20 (0.96-1.51) | 5752 | 1.23 (1.17-1.29) |
| Visual disturbances and blindness | H53 | 3028 | 1.27 (1.20-1.34) | 44 | 1.25 (0.93-1.69) | 2984 | 1.25 (1.17-1.33) |
| **Ear dieases** |  |  |  |  |  |  |  |
| Other disorders of the ear | H90 | 4669 | 1.18 (1.13-1.23) | 65 | 1.20 (0.93-1.53) | 4604 | 1.09 (1.04-1.15) |
| **Cardiovascular disease** |  |  |  |  |  |  |  |
| Chronic rheumatic heart disease | I05 | 3256 | 1.32 (1.25-1.39) | 51 | 1.50 (1.13-1.99) | 3205 | 1.23 (1.15-1.31) |
| Primary hypertension | I10 | 48720 | 1.93 (1.91-1.96) | 657 | 1.99 (1.84-2.15) | 48063 | 1.62 (1.59-1.65) |
| Angina pectoris | I20 | 9207 | 1.69 (1.63-1.75) | 140 | 2.03 (1.72-2.41) | 9067 | 1.45 (1.40-1.51) |
| Acute myocardial infarction | I21 | 5041 | 1.55 (1.48-1.62) | 87 | 2.00 (1.61-2.49) | 4954 | 1.40 (1.33-1.48) |
| Other ischemic heart disease | I22 | 1760 | 1.57 (1.46-1.70) | 25 | 1.66 (1.11-2.48) | 1735 | 1.44 (1.31-1.57) |
| Chronic ischaemic heart disease | I25 | 15177 | 1.59 (1.55-1.63) | 232 | 1.84 (1.61-2.09) | 14945 | 1.41 (1.37-1.46) |
| Embolism and thrombosis | I26 | 5561 | 1.48 (1.42-1.55) | 85 | 1.69 (1.36-2.11) | 5476 | 1.34 (1.28-1.41) |
| Non-rheumatic valve disorders | I34 | 4858 | 1.34 (1.28-1.39) | 55 | 1.11 (0.85-1.45) | 4803 | 1.23 (1.17-1.30) |
| Atrial fibrillation and flutter | I48 | 12662 | 1.45 (1.41-1.49) | 146 | 1.22 (1.03-1.44) | 12516 | 1.34 (1.30-1.39) |
| Other cardiac arrhythmias | I49 | 9066 | 1.35 (1.30-1.39) | 115 | 1.23 (1.02-1.48) | 8951 | 1.26 (1.21-1.31) |
| Heart failure | I50 | 6928 | 1.91 (1.84-1.99) | 93 | 1.95 (1.58-2.40) | 6835 | 1.71 (1.63-1.80) |
| Complications and ill-defined descriptions of heart disease | I51 | 6566 | 1.70 (1.63-1.77) | 78 | 1.56 (1.24-1.96) | 6488 | 1.50 (1.43-1.58) |
| Stroke | I60 | 4278 | 1.30 (1.24-1.36) | 81 | 1.65 (1.32-2.06) | 4197 | 1.29 (1.22-1.36) |
| Other cerebrovascular diseases | I65 | 4012 | 1.37 (1.31-1.44) | 86 | 2.01 (1.62-2.50) | 3926 | 1.34 (1.26-1.42) |
| Aneurysm and dissection | I71 | 1856 | 1.34 (1.25-1.44) | 38 | 1.98 (1.43-2.74) | 1818 | 1.25 (1.15-1.36) |
| Peripheral vascular disease | I73 | 2716 | 1.23 (1.16-1.30) | 73 | 1.99 (1.57-2.52) | 2643 | 1.36 (1.27-1.46) |
| Varicose veins of lower extremities | I83 | 2465 | 1.19 (1.12-1.26) | 22 | 0.80 (0.52-1.22) | 2443 | 1.07 (0.99-1.14) |
| Hypotension | I95 | 5918 | 1.25 (1.20-1.30) | 99 | 1.40 (1.14-1.71) | 5819 | 1.25 (1.20-1.31) |
| **Respiratory system disease** |  |  |  |  |  |  |  |
| Pneumonia | J12 | 10625 | 1.46 (1.42-1.50) | 205 | 1.88 (1.63-2.16) | 10420 | 1.48 (1.42-1.53) |
| Other acute Lower respiratory infections | J20 | 5923 | 1.62 (1.56-1.69) | 102 | 1.93 (1.58-2.36) | 5821 | 1.57 (1.49-1.65) |
| Chronic obstructive pulmonary disease | J44 | 7598 | 1.43 (1.38-1.48) | 178 | 2.03 (1.74-2.36) | 7420 | 1.61 (1.54-1.69) |
| Asthma | J45 | 11842 | 1.46 (1.42-1.50) | 153 | 1.30 (1.10-1.52) | 11689 | 1.39 (1.34-1.44) |
| Pleural effusion | J90 | 5442 | 1.29 (1.24-1.35) | 99 | 1.59 (1.30-1.94) | 5343 | 1.28 (1.22-1.35) |
| Other diseases of the respiratory system | J95 | 4666 | 1.33 (1.28-1.39) | 83 | 1.64 (1.32-2.04) | 4583 | 1.29 (1.22-1.36) |
| Respiratory failure | J96 | 3334 | 1.62 (1.53-1.71) | 65 | 1.97 (1.54-2.53) | 3269 | 1.74 (1.62-1.86) |
| **Digestive system disease** |  |  |  |  |  |  |  |
| Disease of hard tissue of teeth | K00 | 1798 | 1.30 (1.21-1.39) | 27 | 1.27 (0.87-1.87) | 1771 | 1.33 (1.21-1.45) |
| Oesophagitis | K20 | 3850 | 1.25 (1.19-1.31) | 78 | 1.79 (1.42-2.24) | 3772 | 1.19 (1.13-1.27) |
| Gastro-oesophageal reflux disease | K21 | 15589 | 1.39 (1.36-1.42) | 213 | 1.42 (1.24-1.63) | 15376 | 1.26 (1.22-1.29) |
| Other diseases of oesophagus | K22 | 5536 | 1.24 (1.19-1.29) | 129 | 2.14 (1.79-2.56) | 5407 | 1.13 (1.07-1.18) |
| Gastric ulcer | K25 | 2387 | 1.42 (1.33-1.51) | 40 | 1.62 (1.18-2.22) | 2347 | 1.39 (1.29-1.51) |
| Gastritis and duodenitis | K29 | 13686 | 1.26 (1.23-1.29) | 248 | 1.61 (1.42-1.83) | 13438 | 1.21 (1.17-1.25) |
| Disease of stomach and duodenum | K31 | 5681 | 1.37 (1.32-1.43) | 89 | 1.56 (1.26-1.93) | 5592 | 1.27 (1.21-1.33) |
| Umbilical hernia | K42 | 2656 | 2.75 (2.56-2.96) | 21 | 2.15 (1.39-3.35) | 2635 | 2.09 (1.92-2.28) |
| Ventral hernia | K43 | 1994 | 1.99 (1.85-2.15) | 13 | 0.99 (0.57-1.72) | 1981 | 1.78 (1.62-1.95) |
| Diaphragmatic hernia | K44 | 13407 | 1.31 (1.27-1.34) | 205 | 1.51 (1.31-1.74) | 13202 | 1.17 (1.14-1.21) |
| Noninfective gastroenteritis and colitis | K52 | 5150 | 1.28 (1.23-1.34) | 75 | 1.23 (0.98-1.55) | 5075 | 1.30 (1.24-1.37) |
| Diverticular disease of intestine | K57 | 20017 | 1.40 (1.37-1.43) | 272 | 1.42 (1.26-1.61) | 19745 | 1.27 (1.24-1.30) |
| Irritable bowel syndrome | K58 | 3010 | 1.27 (1.21-1.34) | 42 | 1.13 (0.83-1.53) | 2968 | 1.34 (1.25-1.44) |
| Other functional intestinal disorders | K59 | 8857 | 1.23 (1.20-1.27) | 130 | 1.25 (1.05-1.49) | 8727 | 1.20 (1.15-1.25) |
| Other diseases of anus and rectum | K62 | 8721 | 1.22 (1.18-1.26) | 139 | 1.32 (1.12-1.57) | 8582 | 1.19 (1.15-1.24) |
| Other diseases of intestine | K63 | 11385 | 1.39 (1.35-1.43) | 184 | 1.59 (1.37-1.85) | 11201 | 1.31 (1.27-1.36) |
| Haemorrhoids and perianal venous thrombosis | K64 | 6169 | 1.11 (1.08-1.16) | 92 | 1.14 (0.92-1.40) | 6077 | 1.10 (1.05-1.15) |
| Other disorders of peritoneum | K66 | 2837 | 1.60 (1.51-1.70) | 33 | 1.40 (0.99-1.98) | 2804 | 1.45 (1.35-1.56) |
| Other diseases of liver | K76 | 5167 | 2.67 (2.54-2.81) | 91 | 3.34 (2.69-4.14) | 5076 | 2.53 (2.37-2.70) |
| Cholelithiasis | K80 | 7589 | 2.19 (2.11-2.28) | 103 | 2.59 (2.12-3.16) | 7486 | 1.77 (1.69-1.85) |
| Disease of gallbladder and biliary tract | K82 | 2841 | 1.84 (1.73-1.95) | 54 | 2.48 (1.88-3.27) | 2787 | 1.74 (1.61-1.88) |
| Other disease of digestive system | K92 | 6861 | 1.28 (1.24-1.33) | 117 | 1.52 (1.26-1.83) | 6744 | 1.24 (1.19-1.29) |
| **Skin and subcutaneous disease** |  |  |  |  |  |  |  |
| Skin and subcutaneous infections | L01 | 6610 | 1.93 (1.85-2.01) | 74 | 1.57 (1.24-1.98) | 6536 | 1.78 (1.70-1.88) |
| Dermatitis | L20 | 2716 | 1.40 (1.32-1.48) | 40 | 1.43 (1.04-1.96) | 2676 | 1.35 (1.25-1.45) |
| Papulosquamous disorders | L40 | 2079 | 1.55 (1.45-1.66) | 22 | 1.14 (0.75-1.75) | 2057 | 1.50 (1.38-1.64) |
| Pressure ulcer | L89 | 1868 | 1.54 (1.43-1.65) | 38 | 1.87 (1.35-2.60) | 1830 | 1.73 (1.57-1.91) |
| Disorder of the skin and subcutaneous tissue | L98 | 2651 | 1.15 (1.09-1.22) | 45 | 1.32 (0.98-1.78) | 2606 | 1.14 (1.06-1.22) |
| **Musculoskeletal and connective tissue disease** |  |  |  |  |  |  |  |
| Autoimmune arthritis | M05 | 3037 | 1.53 (1.45-1.62) | 30 | 1.10 (0.76-1.58) | 3007 | 1.42 (1.32-1.52) |
| Other inflammatory arthritis | M10 | 15579 | 1.92 (1.87-1.98) | 195 | 1.93 (1.67-2.23) | 15384 | 1.63 (1.58-1.69) |
| Osteoarthritis | M15 | 28395 | 1.64 (1.61-1.67) | 272 | 1.20 (1.06-1.36) | 28123 | 1.38 (1.35-1.41) |
| Other joint disorders | M20 | 13659 | 1.37 (1.34-1.41) | 141 | 1.05 (0.89-1.25) | 13518 | 1.21 (1.18-1.25) |
| Systemic connective tissue disorders | M30 | 2191 | 1.26 (1.18-1.34) | 22 | 0.88 (0.57-1.34) | 2169 | 1.22 (1.13-1.31) |
| Deforming dorsopathies | M40 | 2305 | 1.31 (1.23-1.39) | 29 | 1.13 (0.78-1.63) | 2276 | 1.28 (1.19-1.38) |
| Spondylosis | M47 | 6311 | 1.54 (1.48-1.60) | 72 | 1.38 (1.09-1.75) | 6239 | 1.34 (1.27-1.40) |
| Spondylopathy | M48 | 4091 | 1.67 (1.59-1.76) | 49 | 1.59 (1.19-2.11) | 4042 | 1.45 (1.37-1.54) |
| Other intervertebral disc disorders | M51 | 4502 | 1.58 (1.51-1.66) | 52 | 1.37 (1.04-1.81) | 4450 | 1.42 (1.34-1.50) |
| Dorsalgia | M54 | 8760 | 1.54 (1.49-1.60) | 102 | 1.28 (1.05-1.56) | 8658 | 1.44 (1.38-1.50) |
| Disorders of synovium and tendons | M65 | 3639 | 1.34 (1.28-1.41) | 24 | 0.67 (0.45-1.00) | 3615 | 1.20 (1.13-1.28) |
| Shoulder lesion | M75 | 4103 | 1.39 (1.33-1.46) | 54 | 1.40 (1.06-1.83) | 4049 | 1.24 (1.17-1.31) |
| Other soft tissue disorders, not elsewhere classified | M79 | 7864 | 1.75 (1.69-1.82) | 72 | 1.24 (0.98-1.57) | 7792 | 1.54 (1.47-1.61) |
| **Genitourinary system disease** |  |  |  |  |  |  |  |
| Obstructive and reflux uropathy | N13 | 2170 | 1.31 (1.23-1.39) | 27 | 1.14 (0.78-1.68) | 2143 | 1.25 (1.15-1.35) |
| Acute renal failure | N17 | 9834 | 1.90 (1.84-1.96) | 161 | 2.26 (1.93-2.65) | 9673 | 1.77 (1.70-1.84) |
| Chronic kidney disease | N18 | 9060 | 2.01 (1.94-2.08) | 122 | 2.22 (1.85-2.67) | 8938 | 1.70 (1.63-1.77) |
| Urolithiasis | N20 | 2924 | 1.53 (1.45-1.62) | 40 | 1.51 (1.10-2.07) | 2884 | 1.43 (1.33-1.54) |
| Other disorders of kidney and ureter | N29 | 2810 | 1.56 (1.47-1.65) | 41 | 1.62 (1.18-2.22) | 2769 | 1.47 (1.36-1.58) |
| Bladder disorder | N32 | 4485 | 1.17 (1.12-1.22) | 67 | 1.21 (0.95-1.54) | 4418 | 1.14 (1.08-1.20) |
| Disorders of urinary system, possibly infection | N39 | 9880 | 1.49 (1.45-1.54) | 149 | 1.61 (1.37-1.90) | 9731 | 1.39 (1.33-1.44) |
| Female genital prolapse | N81 | 2547 | 1.21 (1.14-1.28) | 17 | 0.62 (0.39-1.01) | 2530 | 1.06 (0.99-1.14) |
| Polyp of female genital tract | N84 | 1916 | 1.75 (1.63-1.89) | 6 | 0.37 (0.17-0.83) | 1910 | 1.72 (1.56-1.88) |
| Menopausal and perimenopausal disorders | N95 | 1876 | 1.49 (1.39-1.60) | 9 | 0.48 (0.25-0.92) | 1867 | 1.48 (1.36-1.62) |
| **Unnatural cause** |  |  |  |  |  |  |  |
| Injuries due to external causes | S00 | 14888 | 1.06 (1.04-1.09) | 257 | 1.22 (1.08-1.38) | 14631 | 1.08 (1.05-1.12) |
| Complications due to medical treatment | T80 | 11766 | 1.42 (1.38-1.46) | 159 | 1.39 (1.18-1.62) | 11607 | 1.32 (1.28-1.37) |
| Falls | W00 | 10293 | 1.14 (1.11-1.17) | 197 | 1.43 (1.24-1.65) | 10096 | 1.16 (1.12-1.20) |
| Other external causes of accidental injury | W20 | 5349 | 1.17 (1.12-1.22) | 98 | 1.47 (1.20-1.80) | 5251 | 1.14 (1.09-1.20) |
| External causes of morbidity related to medical treatment | Y40 | 15399 | 1.38 (1.35-1.41) | 219 | 1.39 (1.21-1.59) | 15180 | 1.30 (1.26-1.34) |
| External causes of morbidity related to other conditions | Y95 | 2479 | 1.46 (1.37-1.55) | 57 | 2.17 (1.66-2.84) | 2422 | 1.50 (1.39-1.63) |

A total of 113 disease conditions were confirmed to be significantly associated with MAFLD after Bonferroni correction. Significantly higher HRs (95%CIs without overlap) between normal weight (body mass index < 25 kg/m^2^) and over weight/obesity (body mass index ≥ 25 kg/m^2^) individuals were marked in red.

^*^Combined ICD-10 codes derived from the original ICD-10 code and are displayed in the Additional file 1.

^#^Number of the participants diagnosed with the corresponding diseases.

HR, hazard ratio; CI, confidence interval. MAFLD, Metabolic dysfunction-associated fatty liver disease.

Additional file 1: Table S8. PheWAS using Cox regression was conducted to investigate the relationship between MAFLD and cause of death in normal weight and overweight/obese individuals.

| **Causes of death** | **Total**  **(N = 326,606)** | | **Normal weight**  **(N = 83,508)** | | **Over weight/obese**  **(N = 243,098)** | |
| --- | --- | --- | --- | --- | --- | --- |
|  | **No.^*^** | **HR (95% CI)** | **No.^*^** | **HR (95% CI)** | **No.^*^** | **HR (95% CI)** |
| Cardiovascular death | 6025 | 1.64 (1.58-1.71) | 124 | 2.33 (1.94-2.79) | 5901 | 1.59 (1.51-1.67) |
| Digestive system death | 1409 | 1.81 (1.66-1.98) | 58 | 4.15 (3.16-5.45) | 1351 | 2.21 (1.95-2.49) |
| Endocrine system death | 2007 | 3.23 (2.95-3.53) | 27 | 3.00 (2.02-4.45) | 1980 | 3.13 (2.79-3.52) |
| Genitourinary system death | 1203 | 2.05 (1.86-2.26) | 20 | 2.32 (1.47-3.64) | 1183 | 2.02 (1.78-2.29) |
| Infectious and parasitic death | 867 | 1.63 (1.46-1.80) | 116 | 1.99 (1.20-3.28) | 751 | 1.85 (1.62-2.13) |
| Malignant neoplasms death | 7488 | 1.26 (1.21-1.30) | 158 | 1.72 (1.46-2.01) | 7330 | 1.29 (1.23-1.35) |
| Respiratory system death | 3606 | 1.21 (1.15-1.27) | 75 | 1.47 (1.17-1.85) | 3531 | 1.39 (1.31-1.49) |
| Unnatural cause death | 1083 | 1.21 (1.11-1.32) | 29 | 2.01 (1.38-2.93) | 1054 | 1.29 (1.15-1.44) |

After Bonferroni correction, a total of 8 death causes were significant associated with MAFLD. Significantly higher HRs (95%CIs without overlap) were marked in red in normal weight (body mass index < 25 kg/m^2^) or over weight/obesity (body mass index ≥ 25 kg/m^2^) individuals.

**^*^**Number of the MAFLD participants died due to the corresponding causes, including primary and secondary causes of death.

HR, hazard ratio; CI, confidence interval. MAFLD, Metabolic dysfunction-associated fatty liver disease.

Additional file 1: Table S9. PheWAS using Cox regression was conducted to investigate the relationship between MAFLD and cause of death in heavy drinkers and/or nonheavy drinkers.

| **Causes of death** | **Total**  **(N = 326,606)** | | **Non-heavy drinking**  **(N = 256,902)** | | **Heavy drinking**  **(N = 69,704)** | |
| --- | --- | --- | --- | --- | --- | --- |
|  | **No.^*^** | **HR (95% CI)** | **No.^*^** | **HR (95% CI)** | **No.^*^** | **HR (95% CI)** |
| Cardiovascular death | 6025 | 1.64 (1.58-1.71) | 4495 | 1.70 (1.62-1.78) | 1530 | 1.43 (1.31-1.55) |
| Digestive system death | 1409 | 1.81 (1.66-1.98) | 960 | 1.84 (1.66-2.04) | 449 | 1.59 (1.35-1.86) |
| Endocrine system death | 2007 | 3.23 (2.95-3.53) | 1635 | 3.36 (3.05-3.71) | 372 | 2.85 (2.29-3.55) |
| Genitourinary system death | 1203 | 2.05 (1.86-2.26) | 965 | 2.12 (1.90-2.36) | 238 | 1.86 (1.47-2.35) |
| Infectious and parasitic death | 867 | 1.63 (1.46-1.80) | 572 | 1.62 (1.44-1.83) | 295 | 1.59 (1.27-2.00) |
| Malignant neoplasms death | 7488 | 1.26 (1.21-1.30) | 5563 | 1.29 (1.24-1.34) | 1925 | 1.10 (1.03-1.18) |
| Respiratory system death | 3606 | 1.21 (1.15-1.27) | 2667 | 1.26 (1.19-1.33) | 939 | 1.01 (0.91-1.11) |
| Unnatural cause death | 1083 | 1.21 (1.11-1.32) | 792 | 1.21 (1.09-1.34) | 291 | 1.17 (0.97-1.40) |

After Bonferroni correction, a total of 8 death causes were significant associated with MAFLD. Significantly higher HRs (95%CIs without overlap) between non-heavy drinker and heavy drinker (consuming alcohol > 30g/d for male and > 20g/d for female) were marked in red. The recording of alcohol intake is based on recollection and estimation of the average total amount of red wine, spirits, beer, and fruit wine consumed each week or month.

**^*^**Number of the MAFLD participants died due to the corresponding causes, including primary and secondary causes of death.

HR, hazard ratio; CI, confidence interval. MAFLD, Metabolic dysfunction-associated fatty liver disease.

Additional file 1: Table S10. Sensitivity analysis of the relationship between MAFLD and 490 subsequent disease conditions after excluding individuals with preexisting diagnosis-related systemic disease.

| **Medical conditions** | **Code^*^** | **Main analysis** | | **Sensitivity analysis** | |
| --- | --- | --- | --- | --- | --- |
|  |  | **No./N^#^** | **HR (95% CI)** | **No./N^#^** | **HR (95% CI)** |
| **Infectious and parasitic** |  |  |  |  |  |
| Infectious gastroenteritis and colitis | A09 | 7336/163149 | 1.40 (1.35-1.45) | 6820/157202 | 1.39 (1.34-1.44) |
| Sepsis | A41 | 5918/162810 | 1.51 (1.45-1.58) | 5506/157202 | 1.49 (1.43-1.55) |
| Mycoses | B49 | 2527/162935 | 1.29 (1.21-1.36) | 2300/157202 | 1.25 (1.18-1.33) |
| Bacterial infectious agents | B96 | 8188/160304 | 1.49 (1.44-1.54) | 7879/157202 | 1.48 (1.43-1.53) |
| **Malignant neoplasms** |  |  |  |  |  |
| Colon cancer | C18 | 1920/162740 | 1.27 (1.18-1.35) | 1770/155352 | 1.25 (1.17-1.34) |
| Metastatic cancer | C77 | 7220/162361 | 1.21 (1.17-1.26) | 6347/155352 | 1.23 (1.18-1.27) |
| **Benign neoplasms** |  |  |  |  |  |
| Benign tumors | D10 | 17866/151074 | 1.29 (1.26-1.32) | 17764/150374 | 1.29 (1.26-1.32) |
| **Blood system** |  |  |  |  |  |
| Iron deficiency anaemia | D50 | 6817/162113 | 1.38 (1.33-1.43) | 6388/158804 | 1.36 (1.31-1.41) |
| Other anaemias | D51 | 9594/160778 | 1.31 (1.27-1.35) | 9250/158804 | 1.31 (1.27-1.35) |
| **Endocrine system disease** |  |  |  |  |  |
| Hypothyroid conditions | E00 | 7738/160535 | 1.47 (1.42-1.52) | 6533/144665 | 1.45 (1.39-1.50) |
| Diabetes | E10 | 19609/156670 | 4.24 (4.11-4.37) | 17142/144665 | 4.20 (4.07-4.34) |
| Malnutrition | E40 | 3322/163169 | 1.42 (1.35-1.50) | 2398/144665 | 1.25 (1.18-1.33) |
| Obesity | E66 | 21512/160653 | 8.60 (8.26-8.95) | 18060/144665 | 8.55 (8.19-8.92) |
| Disorders of lipoprotein metabolism and other lipidaemias | E78 | 26665/154349 | 1.77 (1.74-1.81) | 23747/144665 | 1.74 (1.70-1.77) |
| Disorders of mineral metabolism | E83 | 2984/163016 | 1.47 (1.39-1.55) | 2306/144665 | 1.38 (1.30-1.47) |
| Volume depletion | E86 | 3874/163025 | 1.43 (1.36-1.50) | 2829/144665 | 1.30 (1.23-1.38) |
| Other disorders of fluid, electrolyte and acid-base balance | E87 | 8207/162784 | 1.43 (1.38-1.48) | 6028/144665 | 1.30 (1.25-1.35) |
| **Mental disorder** |  |  |  |  |  |
| Delirium due to known physiological condition | F05 | 2535/163279 | 1.39 (1.31-1.47) | 2331/158421 | 1.38 (1.29-1.47) |
| Alcohol abuse | F10 | 3267/162002 | 1.55 (1.47-1.64) | 3076/158421 | 1.54 (1.46-1.63) |
| Tobacco abuse | F17 | 9131/162892 | 1.24 (1.20-1.28) | 8369/158421 | 1.25 (1.21-1.29) |
| Depression | F32 | 9717/161211 | 1.63 (1.58-1.68) | 9195/158421 | 1.64 (1.58-1.69) |
| Anxiety | F40 | 7324/162578 | 1.37 (1.32-1.42) | 6686/158421 | 1.35 (1.30-1.40) |
| **Neural system disease** |  |  |  |  |  |
| Epilepsia | G40 | 1685/162258 | 1.26 (1.18-1.36) | 1495/152623 | 1.24 (1.15-1.34) |
| Sleep disorder | G47 | 5120/161322 | 3.71 (3.50-3.93) | 4554/152623 | 3.61 (3.39-3.83) |
| Diseases in nerves, nerve roots and nerve plexa | G50 | 7795/158231 | 1.59 (1.54-1.65) | 7332/152623 | 1.58 (1.52-1.63) |
| Polyneuropathies | G60 | 2257/162999 | 1.96 (1.83-2.10) | 1908/152623 | 1.90 (1.77-2.05) |
| Cerebral palsy and other paralytic syndromes | G80 | 1764/162806 | 1.42 (1.32-1.52) | 1492/152623 | 1.38 (1.28-1.50) |
| **Eye and adnexa disease** |  |  |  |  |  |
| Disorder of eyelid | H02 | 2477/161985 | 1.27 (1.19-1.34) | 2273/155267 | 1.28 (1.20-1.36) |
| Disorders of the lens | H25 | 16810/159905 | 1.12 (1.10-1.15) | 15849/155267 | 1.12 (1.10-1.15) |
| Disorders of choroid and retina | H30 | 5829/161808 | 1.29 (1.24-1.34) | 5352/155267 | 1.28 (1.23-1.34) |
| Visual disturbances and blindness | H53 | 3028/162578 | 1.27 (1.20-1.34) | 2633/155267 | 1.23 (1.16-1.30) |
| **Ear dieases** |  |  |  |  |  |
| Other disorders of the ear | H90 | 4669/162571 | 1.18 (1.13-1.23) | 4501/161112 | 1.18 (1.13-1.23) |
| **Cardiovascular disease** |  |  |  |  |  |
| Chronic rheumatic heart disease | I05 | 3256/163024 | 1.32 (1.25-1.39) | 1812/131094 | 1.20 (1.12-1.28) |
| Primary hypertension | I10 | 48720/142582 | 1.93 (1.91-1.96) | 43113/131094 | 1.96 (1.93-1.99) |
| Angina pectoris | I20 | 9207/157094 | 1.69 (1.63-1.75) | 5894/131094 | 1.60 (1.54-1.67) |
| Acute myocardial infarction | I21 | 5041/160758 | 1.55 (1.48-1.62) | 3488/131094 | 1.49 (1.42-1.57) |
| Other ischemic heart disease | I22 | 1760/162835 | 1.57 (1.46-1.70) | 959/131094 | 1.43 (1.30-1.58) |
| Chronic ischaemic heart disease | I25 | 15177/155301 | 1.59 (1.55-1.63) | 10589/131094 | 1.51 (1.47-1.56) |
| Embolism and thrombosis | I26 | 5561/161104 | 1.48 (1.42-1.55) | 4000/131094 | 1.44 (1.38-1.51) |
| Non-rheumatic valve disorders | I34 | 4858/162465 | 1.34 (1.28-1.39) | 2973/131094 | 1.25 (1.19-1.32) |
| Atrial fibrillation and flutter | I48 | 12662/160257 | 1.45 (1.41-1.49) | 8321/131094 | 1.34 (1.30-1.39) |
| Other cardiac arrhythmias | I49 | 9066/161401 | 1.35 (1.30-1.39) | 5654/131094 | 1.22 (1.18-1.27) |
| Heart failure | I50 | 6928/162080 | 1.91 (1.84-1.99) | 3591/131094 | 1.71 (1.62-1.80) |
| Complications and ill-defined descriptions of heart disease | I51 | 6566/162663 | 1.70 (1.63-1.77) | 3662/131094 | 1.55 (1.48-1.63) |
| Stroke | I60 | 4278/162102 | 1.30 (1.24-1.36) | 2768/131094 | 1.19 (1.13-1.26) |
| Other cerebrovascular diseases | I65 | 4012/162754 | 1.37 (1.31-1.44) | 2306/131094 | 1.20 (1.13-1.28) |
| Aneurysm and dissection | I71 | 1856/163000 | 1.34 (1.25-1.44) | 1122/131094 | 1.28 (1.18-1.40) |
| Peripheral vascular disease | I73 | 2716/162528 | 1.23 (1.16-1.30) | 1423/131094 | 1.02 (0.95-1.10) |
| Varicose veins of lower extremities | I83 | 2465/160493 | 1.19 (1.12-1.26) | 1794/131094 | 1.11 (1.04-1.18) |
| Hypotension | I95 | 5918/162756 | 1.25 (1.20-1.30) | 3501/131094 | 1.11 (1.06-1.16) |
| **Respiratory system disease** |  |  |  |  |  |
| Pneumonia | J12 | 10625/161819 | 1.46 (1.42-1.50) | 8755/148070 | 1.44 (1.40-1.49) |
| Other acute Lower respiratory infections | J20 | 5923/161465 | 1.62 (1.56-1.69) | 4897/148070 | 1.64 (1.56-1.71) |
| Chronic obstructive pulmonary disease | J44 | 7598/161973 | 1.43 (1.38-1.48) | 5697/148070 | 1.39 (1.34-1.45) |
| Asthma | J45 | 11842/156556 | 1.46 (1.42-1.50) | 10838/148070 | 1.46 (1.42-1.51) |
| Pleural effusion | J90 | 5442/162627 | 1.29 (1.24-1.35) | 4608/148070 | 1.28 (1.23-1.34) |
| Other diseases of the respiratory system | J95 | 4666/162756 | 1.33 (1.28-1.39) | 3794/148070 | 1.33 (1.27-1.40) |
| Respiratory failure | J96 | 3334/163125 | 1.62 (1.53-1.71) | 2551/148070 | 1.65 (1.55-1.75) |
| **Digestive system disease** |  |  |  |  |  |
| Disease of hard tissue of teeth | K00 | 1798/162002 | 1.30 (1.21-1.39) | 1179/121308 | 1.25 (1.15-1.36) |
| Oesophagitis | K20 | 3850/160550 | 1.25 (1.19-1.31) | 2401/121308 | 1.25 (1.17-1.32) |
| Gastro-oesophageal reflux disease | K21 | 15589/157687 | 1.39 (1.36-1.42) | 9834/121308 | 1.38 (1.33-1.42) |
| Other diseases of oesophagus | K22 | 5536/160822 | 1.24 (1.19-1.29) | 3538/121308 | 1.24 (1.18-1.30) |
| Gastric ulcer | K25 | 2387/162140 | 1.42 (1.33-1.51) | 1445/121308 | 1.41 (1.31-1.53) |
| Gastritis and duodenitis | K29 | 13686/155701 | 1.26 (1.23-1.29) | 8921/121308 | 1.23 (1.19-1.27) |
| Disease of stomach and duodenum | K31 | 5681/162117 | 1.37 (1.32-1.43) | 3011/121308 | 1.32 (1.25-1.39) |
| Umbilical hernia | K42 | 2656/161773 | 2.75 (2.56-2.96) | 1823/121308 | 2.87 (2.63-3.13) |
| Ventral hernia | K43 | 1994/162307 | 1.99 (1.85-2.15) | 1060/121308 | 1.88 (1.70-2.07) |
| Diaphragmatic hernia | K44 | 13407/155900 | 1.31 (1.27-1.34) | 8890/121308 | 1.32 (1.28-1.36) |
| Noninfective gastroenteritis and colitis | K52 | 5150/159177 | 1.28 (1.23-1.34) | 3180/121308 | 1.22 (1.16-1.28) |
| Diverticular disease of intestine | K57 | 20017/158363 | 1.40 (1.37-1.43) | 13474/121308 | 1.40 (1.36-1.43) |
| Irritable bowel syndrome | K58 | 3010/162235 | 1.27 (1.21-1.34) | 1579/121308 | 1.20 (1.11-1.29) |
| Other functional intestinal disorders | K59 | 8857/161616 | 1.23 (1.20-1.27) | 5533/121308 | 1.19 (1.15-1.24) |
| Other diseases of anus and rectum | K62 | 8721/157065 | 1.22 (1.18-1.26) | 5869/121308 | 1.18 (1.14-1.23) |
| Other diseases of intestine | K63 | 11385/161152 | 1.39 (1.35-1.43) | 7339/121308 | 1.39 (1.35-1.44) |
| Haemorrhoids and perianal venous thrombosis | K64 | 6169/163303 | 1.11 (1.08-1.16) | 3880/121308 | 1.06 (1.02-1.11) |
| Other disorders of peritoneum | K66 | 2837/162728 | 1.60 (1.51-1.70) | 1774/121308 | 1.65 (1.54-1.78) |
| Other diseases of liver | K76 | 5167/162616 | 2.67 (2.54-2.81) | 3118/121308 | 2.64 (2.48-2.82) |
| Cholelithiasis | K80 | 7589/158857 | 2.19 (2.11-2.28) | 5090/121308 | 2.26 (2.15-2.37) |
| Disease of gallbladder and biliary tract | K82 | 2841/162529 | 1.84 (1.73-1.95) | 1789/121308 | 1.83 (1.70-1.98) |
| Other disease of digestive system | K92 | 6861/161874 | 1.28 (1.24-1.33) | 4214/121308 | 1.24 (1.19-1.30) |
| **Skin and subcutaneous disease** |  |  |  |  |  |
| Skin and subcutaneous infections | L01 | 6610/159887 | 1.93 (1.85-2.01) | 6095/152260 | 1.92 (1.84-2.01) |
| Dermatitis | L20 | 2716/162642 | 1.40 (1.32-1.48) | 2459/152260 | 1.39 (1.31-1.48) |
| Papulosquamous disorders | L40 | 2079/162545 | 1.55 (1.45-1.66) | 1869/152260 | 1.53 (1.42-1.64) |
| Pressure ulcer | L89 | 1868/163257 | 1.54 (1.43-1.65) | 1620/152260 | 1.51 (1.39-1.62) |
| Disorder of the skin and subcutaneous tissue | L98 | 2651/161972 | 1.15 (1.09-1.22) | 2363/152260 | 1.13 (1.07-1.20) |
| **Musculoskeletal and connective tissue disease** |  |  |  |  |  |
| Autoimmune arthritis | M05 | 3037/162250 | 1.53 (1.45-1.62) | 1955/134328 | 1.42 (1.33-1.52) |
| Other inflammatory arthritis | M10 | 15579/160552 | 1.92 (1.87-1.98) | 10919/134328 | 1.89 (1.83-1.95) |
| Osteoarthritis | M15 | 28395/153373 | 1.64 (1.61-1.67) | 22127/134328 | 1.62 (1.58-1.65) |
| Other joint disorders | M20 | 13659/154075 | 1.37 (1.34-1.41) | 10263/134328 | 1.32 (1.29-1.36) |
| Systemic connective tissue disorders | M30 | 2191/162832 | 1.26 (1.18-1.34) | 1542/134328 | 1.22 (1.13-1.31) |
| Deforming dorsopathies | M40 | 2305/162876 | 1.31 (1.23-1.39) | 1319/134328 | 1.16 (1.08-1.26) |
| Spondylosis | M47 | 6311/161812 | 1.54 (1.48-1.60) | 3855/134328 | 1.41 (1.35-1.48) |
| Spondylopathy | M48 | 4091/162604 | 1.67 (1.59-1.76) | 2553/134328 | 1.61 (1.51-1.71) |
| Other intervertebral disc disorders | M51 | 4502/161517 | 1.58 (1.51-1.66) | 2868/134328 | 1.49 (1.40-1.57) |
| Dorsalgia | M54 | 8760/159333 | 1.54 (1.49-1.60) | 6104/134328 | 1.47 (1.41-1.52) |
| Disorders of synovium and tendons | M65 | 3639/161060 | 1.34 (1.28-1.41) | 2499/134328 | 1.29 (1.22-1.37) |
| Shoulder lesion | M75 | 4103/161327 | 1.39 (1.33-1.46) | 2761/134328 | 1.35 (1.27-1.42) |
| Other soft tissue disorders, not elsewhere classified | M79 | 7864/159848 | 1.75 (1.69-1.82) | 5453/134328 | 1.67 (1.60-1.75) |
| **Genitourinary system disease** |  |  |  |  |  |
| Obstructive and reflux uropathy | N13 | 2170/162786 | 1.31 (1.23-1.39) | 1636/137732 | 1.27 (1.18-1.37) |
| Acute renal failure | N17 | 9834/162926 | 1.90 (1.84-1.96) | 7719/137732 | 1.88 (1.81-1.95) |
| Chronic kidney disease | N18 | 9060/162981 | 2.01 (1.94-2.08) | 6787/137732 | 1.98 (1.90-2.06) |
| Urolithiasis | N20 | 2924/161006 | 1.53 (1.45-1.62) | 2367/137732 | 1.52 (1.43-1.62) |
| Other disorders of kidney and ureter | N29 | 2810/162789 | 1.56 (1.47-1.65) | 2118/137732 | 1.55 (1.45-1.66) |
| Bladder disorder | N32 | 4485/161276 | 1.17 (1.12-1.22) | 3349/137732 | 1.13 (1.08-1.19) |
| Disorders of urinary system, possibly infection | N39 | 9880/159007 | 1.49 (1.45-1.54) | 7640/137732 | 1.46 (1.41-1.51) |
| Female genital prolapse | N81 | 2547/160936 | 1.21 (1.14-1.28) | 1853/137732 | 1.15 (1.07-1.23) |
| Polyp of female genital tract | N84 | 1916/160791 | 1.75 (1.63-1.89) | 1520/137732 | 1.74 (1.60-1.88) |
| Menopausal and perimenopausal disorders | N95 | 1876/160763 | 1.49 (1.39-1.60) | 1391/137732 | 1.48 (1.37-1.61) |
| **Unnatural cause** |  |  |  |  |  |
| Injuries due to external causes | S00 | 14888/153893 | 1.06 (1.04-1.09) | 13458/145044 | 1.04 (1.02-1.07) |
| Complications due to medical treatment | T80 | 11766/157629 | 1.42 (1.38-1.46) | 10367/145044 | 1.42 (1.38-1.47) |
| Falls | W00 | 10293/159257 | 1.14 (1.11-1.17) | 8776/145044 | 1.11 (1.08-1.15) |
| Other external causes of accidental injury | W20 | 5349/159265 | 1.17 (1.12-1.22) | 4566/145044 | 1.16 (1.11-1.21) |
| External causes of morbidity related to medical treatment | Y40 | 15399/157448 | 1.38 (1.35-1.41) | 13707/145044 | 1.38 (1.34-1.41) |
| External causes of morbidity related to other conditions | Y95 | 2479/163260 | 1.46 (1.37-1.55) | 1982/145044 | 1.43 (1.34-1.53) |

A total of 113 disease conditions were confirmed to be significantly associated with MAFLD after Bonferroni correction. Significantly higher HRs (95%CIs without overlap) between normal weight (body mass index < 25 kg/m^2^) and over weight/obesity (body mass index ≥ 25 kg/m^2^) individuals were marked in red.

^*^Combined ICD-10 codes derived from the original ICD-10 code and are displayed in the Additional file 1.

^#^NO. indicates the number of the MAFLD participants diagnosed with the corresponding diseases. N indicates the number of the MAFLD participants included to this analysis.

HR, hazard ratio; CI, confidence interval. MAFLD, Metabolic dysfunction-associated fatty liver disease.

Additional file 1: Table S11. Sensitivity analysis of the relationship between MAFLD and cause of death after excluding individuals with preexisting diagnosis-related systemic disease.

| **Causes of death** | **Main analysis** | | **Sensitivity analysis** | |
| --- | --- | --- | --- | --- |
|  | **No./N^#^** | **HR (95% CI)** | **No./N^#^** | **HR (95% CI)** |
| Cardiovascular death | 6025/163303 | 1.64 (1.58-1.71) | 3156/131094 | 1.51 (1.43-1.59) |
| Digestive system death | 1409/163303 | 1.81 (1.66-1.98) | 835/121308 | 2.05 (1.82-2.30) |
| Endocrine system death | 2007/163303 | 3.23 (2.95-3.53) | 1018/144665 | 3.03 (2.69-3.42) |
| Genitourinary system death | 1203/163303 | 2.05 (1.86-2.26) | 812/137732 | 2.01 (1.79-2.26) |
| Infectious and parasitic death | 867/163303 | 1.63 (1.46-1.80) | 649/146851 | 1.65 (1.48-1.82) |
| Malignant neoplasms death | 7488/163303 | 1.26 (1.21-1.30) | 6182/155352 | 1.27 (1.22-1.31) |
| Respiratory system death | 3606/163303 | 1.21 (1.15-1.27) | 2738/148070 | 1.24 (1.17-1.31) |
| Unnatural cause death | 1083/163303 | 1.21 (1.11-1.32) | 847/145044 | 1.21 (1.10-1.34) |

After Bonferroni correction, a total of 8 death causes were significant associated with MAFLD. Significantly higher HRs (95%CIs without overlap) between non-heavy drinker and heavy drinker (consuming alcohol > 30g/d for male and > 20g/d for female) were marked in red. The recording of alcohol intake is based on recollection and estimation of the average total amount of red wine, spirits, beer, and fruit wine consumed each week or month.

^#^NO. indicates the number of the MAFLD participants dead duo to the corresponding causes. N indicates the number of the MAFLD participants included to this analysis.

HR, hazard ratio; CI, confidence interval. MAFLD, Metabolic dysfunction-associated fatty liver disease.

Additional file 1: Table S12. PheWAS using Cox regression was conducted to investigate the association of nonfibrotic MAFLD and fibrotic MAFLD with 490 subsequent disease conditions.

| **Medical conditions** | **Code^*^** | **Non-Fibrotic MAFLD vs.**  **Non-MAFLD (N = 266,772)** | | **Fibrotic MAFLD vs.**  **Non-MAFLD (N = 275,966)** | |
| --- | --- | --- | --- | --- | --- |
|  |  | **No.^#^** | **HR (95% CI)** | **No.^#^** | **HR (95% CI)** |
| **Infectious and parasitic** |  |  |  |  |  |
| Infectious gastroenteritis and colitis | A09 | 2997 | 1.27(1.21-1.34) | 4333 | 1.71(1.63-1.79) |
| Sepsis | A41 | 2007 | 1.28(1.20-1.36) | 3883 | 2.32(2.19-2.45) |
| Mycoses | B49 | 952 | 1.11(1.02-1.21) | 1586 | 1.72(1.59-1.86) |
| Bacterial infectious agents | B96 | 3009 | 1.31(1.24-1.38) | 4974 | 2.02(1.93-2.11) |
| **Malignant neoplasms** |  |  |  |  |  |
| Colon cancer | C18 | 737 | 1.19(1.08-1.32) | 1222 | 1.83(1.67-2.00) |
| Metastatic cancer | C77 | 2964 | 1.15(1.09-1.20) | 4399 | 1.57(1.50-1.65) |
| **Benign neoplasms** |  |  |  |  |  |
| Benign tumors | D10 | 8080 | 1.25(1.21-1.29) | 9709 | 1.39(1.35-1.43) |
| **Blood system** |  |  |  |  |  |
| Iron deficiency anaemia | D50 | 2578 | 1.20(1.13-1.27) | 4234 | 1.83(1.75-1.93) |
| Other anaemias | D51 | 3416 | 1.16(1.10-1.21) | 6316 | 2.01(1.93-2.09) |
| **Endocrine system disease** |  |  |  |  |  |
| Hypothyroid conditions | E00 | 3311 | 1.44(1.37-1.52) | 4456 | 1.80(1.71-1.88) |
| Diabetes | E10 | 6043 | 4.28(4.06-4.51) | 11351 | 7.99(7.60-8.40) |
| Malnutrition | E40 | 1252 | 1.25(1.16-1.36) | 2064 | 1.94(1.81-2.08) |
| Obesity | E66 | 8281 | 8.11(7.65-8.59) | 10116 | 9.27(8.75-9.82) |
| Disorders of lipoprotein metabolism and other lipidaemias | E78 | 11289 | 1.79(1.73-1.84) | 15611 | 2.39(2.33-2.45) |
| Disorders of mineral metabolism | E83 | 1097 | 1.26(1.15-1.37) | 1842 | 1.98(1.83-2.13) |
| Volume depletion | E86 | 1323 | 1.22(1.13-1.32) | 2644 | 2.28(2.13-2.44) |
| Other disorders of fluid, electrolyte and acid-base balance | E87 | 2769 | 1.19(1.13-1.25) | 5439 | 2.20(2.10-2.30) |
| **Mental disorder** |  |  |  |  |  |
| Delirium due to known physiological condition | F05 | 693 | 1.02(0.92-1.13) | 1931 | 2.69(2.48-2.92) |
| Alcohol abuse | F10 | 1378 | 1.34(1.24-1.45) | 1633 | 1.46(1.36-1.57) |
| Tobacco abuse | F17 | 4321 | 1.19(1.14-1.24) | 4548 | 1.14(1.10-1.19) |
| Depression | F32 | 4547 | 1.60(1.53-1.67) | 4734 | 1.53(1.46-1.59) |
| Anxiety | F40 | 3467 | 1.31(1.25-1.38) | 3640 | 1.28(1.22-1.34) |
| **Neural system disease** |  |  |  |  |  |
| Epilepsia | G40 | 717 | 1.18(1.06-1.31) | 948 | 1.43(1.30-1.58) |
| Sleep disorder | G47 | 1918 | 3.11(2.86-3.38) | 2449 | 3.67(3.38-3.98) |
| Diseases in nerves, nerve roots and nerve plexa | G50 | 3464 | 1.56(1.49-1.64) | 4026 | 1.67(1.59-1.75) |
| Polyneuropathies | G60 | 665 | 1.39(1.24-1.55) | 1455 | 2.83(2.57-3.12) |
| Cerebral palsy and other paralytic syndromes | G80 | 615 | 1.25(1.12-1.40) | 1120 | 2.12(1.92-2.34) |
| **Eye and adnexa disease** |  |  |  |  |  |
| Disorder of eyelid | H02 | 994 | 1.21(1.11-1.33) | 1599 | 1.80(1.67-1.95) |
| Disorders of the lens | H25 | 6065 | 1.01(0.98-1.05) | 11772 | 1.91(1.86-1.97) |
| Disorders of choroid and retina | H30 | 1971 | 1.10(1.03-1.17) | 4127 | 2.17(2.06-2.28) |
| Visual disturbances and blindness | H53 | 1235 | 1.17(1.08-1.26) | 1865 | 1.64(1.53-1.76) |
| **Ear dieases** |  |  |  |  |  |
| Other disorders of the ear | H90 | 1843 | 1.10(1.03-1.17) | 3222 | 1.80(1.71-1.91) |
| **Cardiovascular disease** |  |  |  |  |  |
| Chronic rheumatic heart disease | I05 | 1020 | 1.13(1.03-1.23) | 2461 | 2.55(2.38-2.74) |
| Primary hypertension | I10 | 20965 | 1.85(1.81-1.89) | 27305 | 2.46(2.42-2.51) |
| Angina pectoris | I20 | 3617 | 1.63(1.55-1.72) | 5575 | 2.39(2.28-2.50) |
| Acute myocardial infarction | I21 | 2042 | 1.49(1.39-1.59) | 2859 | 1.95(1.83-2.07) |
| Other ischemic heart disease | I22 | 666 | 1.48(1.32-1.66) | 1087 | 2.23(2.02-2.48) |
| Chronic ischaemic heart disease | I25 | 5907 | 1.52(1.46-1.58) | 9357 | 2.34(2.26-2.42) |
| Embolism and thrombosis | I26 | 2104 | 1.36(1.27-1.44) | 3473 | 2.09(1.98-2.21) |
| Non-rheumatic valve disorders | I34 | 1566 | 1.15(1.07-1.23) | 3506 | 2.42(2.28-2.56) |
| Atrial fibrillation and flutter | I48 | 4168 | 1.26(1.21-1.32) | 9155 | 2.67(2.57-2.77) |
| Other cardiac arrhythmias | I49 | 3218 | 1.22(1.17-1.29) | 6350 | 2.28(2.19-2.38) |
| Heart failure | I50 | 1943 | 1.54(1.44-1.64) | 4752 | 3.55(3.35-3.76) |
| Complications and ill-defined descriptions of heart disease | I51 | 2131 | 1.48(1.39-1.58) | 4426 | 2.90(2.74-3.06) |
| Stroke | I60 | 1575 | 1.23(1.15-1.32) | 2846 | 2.07(1.95-2.20) |
| Other cerebrovascular diseases | I65 | 1381 | 1.21(1.12-1.30) | 2697 | 2.21(2.07-2.36) |
| Aneurysm and dissection | I71 | 634 | 1.28(1.15-1.44) | 1396 | 2.63(2.39-2.90) |
| Peripheral vascular disease | I73 | 833 | 0.93(0.85-1.01) | 1919 | 1.99(1.84-2.14) |
| Varicose veins of lower extremities | I83 | 907 | 1.06(0.97-1.16) | 1685 | 1.83(1.69-1.98) |
| Hypotension | I95 | 1979 | 1.07(1.01-1.13) | 4227 | 2.14(2.04-2.26) |
| **Respiratory system disease** |  |  |  |  |  |
| Pneumonia | J12 | 3827 | 1.27(1.21-1.33) | 6684 | 2.08(2.00-2.17) |
| Other acute Lower respiratory infections | J20 | 2135 | 1.45(1.36-1.54) | 3477 | 2.18(2.06-2.31) |
| Chronic obstructive pulmonary disease | J44 | 2824 | 1.17(1.11-1.23) | 4592 | 1.76(1.68-1.85) |
| Asthma | J45 | 5684 | 1.41(1.36-1.47) | 5680 | 1.28(1.24-1.33) |
| Pleural effusion | J90 | 1896 | 1.11(1.05-1.19) | 3741 | 2.05(1.95-2.17) |
| Other diseases of the respiratory system | J95 | 1786 | 1.15(1.08-1.23) | 2901 | 1.75(1.65-1.85) |
| Respiratory failure | J96 | 1136 | 1.29(1.19-1.40) | 2111 | 2.23(2.08-2.40) |
| **Digestive system disease** |  |  |  |  |  |
| Disease of hard tissue of teeth | K00 | 844 | 1.27(1.15-1.40) | 909 | 1.25(1.13-1.37) |
| Oesophagitis | K20 | 1778 | 1.24(1.16-1.33) | 2134 | 1.37(1.28-1.46) |
| Gastro-oesophageal reflux disease | K21 | 7217 | 1.36(1.31-1.40) | 8364 | 1.45(1.41-1.50) |
| Other diseases of oesophagus | K22 | 2491 | 1.23(1.16-1.30) | 3190 | 1.45(1.38-1.53) |
| Gastric ulcer | K25 | 934 | 1.30(1.19-1.43) | 1429 | 1.84(1.69-2.00) |
| Gastritis and duodenitis | K29 | 5842 | 1.18(1.14-1.22) | 7930 | 1.49(1.44-1.54) |
| Disease of stomach and duodenum | K31 | 2376 | 1.28(1.20-1.35) | 3351 | 1.67(1.58-1.76) |
| Umbilical hernia | K42 | 1116 | 2.51(2.26-2.78) | 1236 | 2.56(2.31-2.83) |
| Ventral hernia | K43 | 827 | 1.93(1.73-2.15) | 1033 | 2.21(1.99-2.46) |
| Diaphragmatic hernia | K44 | 6177 | 1.32(1.27-1.37) | 7491 | 1.48(1.43-1.53) |
| Noninfective gastroenteritis and colitis | K52 | 2185 | 1.18(1.11-1.25) | 2929 | 1.45(1.37-1.53) |
| Diverticular disease of intestine | K57 | 8996 | 1.39(1.35-1.44) | 11250 | 1.64(1.59-1.69) |
| Irritable bowel syndrome | K58 | 1524 | 1.31(1.22-1.41) | 1465 | 1.16(1.08-1.25) |
| Other functional intestinal disorders | K59 | 3349 | 1.12(1.07-1.18) | 5763 | 1.81(1.73-1.88) |
| Other diseases of anus and rectum | K62 | 4097 | 1.21(1.16-1.26) | 4663 | 1.27(1.22-1.33) |
| Other diseases of intestine | K63 | 5050 | 1.34(1.28-1.39) | 6304 | 1.55(1.49-1.61) |
| Haemorrhoids and perianal venous thrombosis | K64 | 2915 | 1.11(1.05-1.16) | 3438 | 1.23(1.17-1.29) |
| Other disorders of peritoneum | K66 | 1298 | 1.59(1.47-1.73) | 1465 | 1.66(1.53-1.80) |
| Other diseases of liver | K76 | 2038 | 2.46(2.28-2.66) | 2617 | 2.94(2.74-3.16) |
| Cholelithiasis | K80 | 3350 | 2.13(2.02-2.26) | 3544 | 2.07(1.96-2.19) |
| Disease of gallbladder and biliary tract | K82 | 1213 | 1.79(1.64-1.96) | 1518 | 2.07(1.90-2.25) |
| Other disease of digestive system | K92 | 2861 | 1.21(1.15-1.28) | 3985 | 1.57(1.49-1.64) |
| **Skin and subcutaneous disease** |  |  |  |  |  |
| Skin and subcutaneous infections | L01 | 2354 | 1.59(1.50-1.69) | 3762 | 2.36(2.23-2.50) |
| Dermatitis | L20 | 1140 | 1.31(1.20-1.42) | 1587 | 1.69(1.57-1.83) |
| Papulosquamous disorders | L40 | 943 | 1.52(1.38-1.67) | 1115 | 1.66(1.51-1.82) |
| Pressure ulcer | L89 | 543 | 1.20(1.07-1.36) | 1361 | 2.83(2.56-3.12) |
| Disorder of the skin and subcutaneous tissue | L98 | 1110 | 1.10(1.01-1.19) | 1790 | 1.64(1.53-1.77) |
| **Musculoskeletal and connective tissue disease** |  |  |  |  |  |
| Autoimmune arthritis | M05 | 1286 | 1.48(1.36-1.61) | 1682 | 1.79(1.65-1.93) |
| Other inflammatory arthritis | M10 | 6161 | 1.78(1.72-1.85) | 9031 | 2.46(2.37-2.55) |
| Osteoarthritis | M15 | 11792 | 1.51(1.47-1.55) | 16301 | 2.01(1.96-2.06) |
| Other joint disorders | M20 | 6134 | 1.33(1.28-1.37) | 7224 | 1.43(1.38-1.48) |
| Systemic connective tissue disorders | M30 | 863 | 1.17(1.06-1.28) | 1444 | 1.81(1.67-1.97) |
| Deforming dorsopathies | M40 | 953 | 1.21(1.10-1.32) | 1338 | 1.57(1.45-1.71) |
| Spondylosis | M47 | 2615 | 1.43(1.35-1.51) | 3650 | 1.85(1.76-1.95) |
| Spondylopathy | M48 | 1620 | 1.62(1.50-1.74) | 2409 | 2.23(2.08-2.39) |
| Other intervertebral disc disorders | M51 | 2022 | 1.57(1.47-1.67) | 2281 | 1.62(1.52-1.73) |
| Dorsalgia | M54 | 3734 | 1.45(1.38-1.52) | 4658 | 1.66(1.59-1.74) |
| Disorders of synovium and tendons | M65 | 1638 | 1.30(1.21-1.39) | 1868 | 1.36(1.27-1.45) |
| Shoulder lesion | M75 | 1939 | 1.38(1.30-1.48) | 2104 | 1.37(1.29-1.46) |
| Other soft tissue disorders, not elsewhere classified | M79 | 3157 | 1.61(1.52-1.69) | 4223 | 1.99(1.89-2.09) |
| **Genitourinary system disease** |  |  |  |  |  |
| Obstructive and reflux uropathy | N13 | 879 | 1.20(1.09-1.31) | 1366 | 1.72(1.58-1.88) |
| Acute renal failure | N17 | 3010 | 1.54(1.46-1.63) | 6358 | 3.08(2.94-3.23) |
| Chronic kidney disease | N18 | 2640 | 1.68(1.58-1.78) | 6194 | 3.74(3.55-3.93) |
| Urolithiasis | N20 | 1270 | 1.49(1.37-1.62) | 1516 | 1.64(1.51-1.77) |
| Other disorders of kidney and ureter | N29 | 1053 | 1.42(1.30-1.55) | 1750 | 2.19(2.02-2.38) |
| Bladder disorder | N32 | 1773 | 1.16(1.08-1.23) | 2993 | 1.81(1.71-1.92) |
| Disorders of urinary system, possibly infection | N39 | 3663 | 1.34(1.28-1.40) | 6020 | 2.05(1.97-2.14) |
| Female genital prolapse | N81 | 1264 | 1.23(1.14-1.33) | 1239 | 1.10(1.02-1.19) |
| Polyp of female genital tract | N84 | 867 | 1.58(1.42-1.74) | 821 | 1.36(1.22-1.50) |
| Menopausal and perimenopausal disorders | N95 | 821 | 1.39(1.26-1.54) | 849 | 1.32(1.20-1.46) |
| **Unnatural cause** |  |  |  |  |  |
| Injuries due to external causes | S00 | 6320 | 1.00(0.96-1.03) | 8945 | 1.31(1.27-1.35) |
| Complications due to medical treatment | T80 | 4654 | 1.30(1.25-1.36) | 7035 | 1.84(1.77-1.91) |
| Falls | W00 | 3950 | 1.01(0.97-1.06) | 6474 | 1.55(1.49-1.61) |
| Other external causes of accidental injury | W20 | 2383 | 1.11(1.05-1.18) | 2910 | 1.25(1.19-1.32) |
| External causes of morbidity related to medical treatment | Y40 | 6063 | 1.27(1.22-1.32) | 9395 | 1.85(1.79-1.91) |
| External causes of morbidity related to other conditions | Y95 | 814 | 1.19(1.08-1.31) | 1727 | 2.37(2.18-2.57) |

A total of 113 disease conditions were confirmed to be significantly associated with MAFLD after Bonferroni correction. Compared with individuals with non-MAFLD, significantly higher HRs (95%CIs without overlap) for non-fibrotic MAFLD or fibrotic MAFLD were marked in red.

^*^Combined ICD-10 codes derived from the original ICD-10 code and are displayed in the Additional file 1.

^#^Number of the participants diagnosed with the corresponding diseases.

HR, hazard ratio; CI, confidence interval. MAFLD, Metabolic dysfunction-associated fatty liver disease.

Additional file 1: Table S13. PheWAS using Cox regression was conducted to investigate the relationship of nonfibrotic and fibrotic MAFLD with causes of death.

| **Causes of death** | **Non-Fibrotic MAFLD vs.**  **Non-MAFLD (N = 266,772)** | | **Fibrotic MAFLD vs.**  **Non-MAFLD (N = 275,966)** | |
| --- | --- | --- | --- | --- |
|  | **No.^*^** | **HR (95% CI)** | **No.^*^** | **HR (95% CI)** |
| Cardiovascular death | 1642 | 1.32 (1.23-1.42) | 4142 | 3.12 (2.94-3.31) |
| Digestive system death | 391 | 1.44 (1.24-1.66) | 953 | 3.26 (2.88-3.70) |
| Endocrine system death | 350 | 2.24 (1.88-2.68) | 1392 | 8.38 (7.20-9.76) |
| Genitourinary system death | 243 | 1.37 (1.14-1.65) | 849 | 4.52 (3.89-5.25) |
| Infectious and parasitic death | 672 | 1.62 (1.44-1.83) | 395 | 1.59 (1.27-2.00) |
| Malignant neoplasms death | 2746 | 1.14 (1.08-1.20) | 4896 | 1.88 (1.79-1.96) |
| Respiratory system death | 1072 | 0.94 (0.86-1.01) | 2625 | 2.14 (2.01-2.29) |
| Unnatural cause death | 327 | 0.97 (0.84-1.12) | 772 | 2.10 (1.86-2.37) |

After Bonferroni correction, a total of 8 death causes were significant associated with MAFLD. Compared with individuals with non-MAFLD, significantly higher HRs (95%CIs without overlap) for non-fibrotic MAFLD or fibrotic MAFLD were marked in red.

**^*^**Number of the participants died due to the corresponding causes, including primary and secondary causes of death.

HR, hazard ratio; CI, confidence interval. MAFLD, Metabolic dysfunction-associated fatty liver disease.

Additional file 1: Table S14. PheWAS using Cox regression was conducted to investigate the association of MAFLD with or without MBOAT7 rs641738 (C>T) and subsequent 490 disease conditions.

| **Medical conditions** | **Code^*^** | **MAFLD without** **MBOAT7 rs641738** **(C>T) vs.**  **Non-MAFLD (N = 246,515)** | | **MAFLD with MBOAT7**  **rs641738 (C>T) vs.**  **Non-MAFLD (N = 243,394)** | |
| --- | --- | --- | --- | --- | --- |
|  |  | **No.^#^** | **HR (95% CI)** | **No.^#^** | **HR (95% CI)** |
| **Infectious and parasitic** |  |  |  |  |  |
| Infectious gastroenteritis and colitis | A09 | 3685 | 1.38 (1.32-1.44) | 3651 | 1.43 (1.37-1.49) |
| Sepsis | A41 | 2899 | 1.45 (1.38-1.52) | 3019 | 1.58 (1.51-1.66) |
| Mycoses | B49 | 1271 | 1.27 (1.18-1.36) | 1256 | 1.31 (1.22-1.40) |
| Bacterial infectious agents | B96 | 3958 | 1.41 (1.35-1.47) | 4230 | 1.57 (1.51-1.64) |
| **Malignant neoplasms** |  |  |  |  |  |
| Colon cancer | C18 | 951 | 1.23 (1.13-1.33) | 969 | 1.31 (1.20-1.41) |
| Metastatic cancer | C77 | 3671 | 1.21 (1.16-1.26) | 3549 | 1.22 (1.17-1.27) |
| **Benign neoplasms** |  |  |  |  |  |
| Benign tumors | D10 | 8999 | 1.27 (1.24-1.30) | 8867 | 1.31 (1.27-1.34) |
| **Blood system** |  |  |  |  |  |
| Iron deficiency anaemia | D50 | 3337 | 1.32 (1.26-1.38) | 3480 | 1.44 (1.38-1.50) |
| Other anaemias | D51 | 4749 | 1.27 (1.22-1.32) | 4845 | 1.35 (1.30-1.40) |
| **Endocrine system disease** |  |  |  |  |  |
| Hypothyroid conditions | E00 | 3903 | 1.45 (1.39-1.51) | 3835 | 1.49 (1.43-1.55) |
| Diabetes | E10 | 9379 | 3.94 (3.81-4.08) | 10230 | 4.55 (4.40-4.71) |
| Malnutrition | E40 | 1628 | 1.36 (1.28-1.45) | 1694 | 1.48 (1.39-1.57) |
| Obesity | E66 | 10261 | 7.99 (7.66-8.33) | 11251 | 9.26 (8.88-9.65) |
| Disorders of lipoprotein metabolism and other lipidaemias | E78 | 13338 | 1.73 (1.69-1.77) | 13327 | 1.82 (1.78-1.86) |
| Disorders of mineral metabolism | E83 | 1403 | 1.35 (1.26-1.44) | 1581 | 1.59 (1.49-1.70) |
| Volume depletion | E86 | 1932 | 1.39 (1.32-1.48) | 1942 | 1.46 (1.38-1.55) |
| Other disorders of fluid, electrolyte and acid-base balance | E87 | 4000 | 1.37 (1.31-1.42) | 4207 | 1.50 (1.44-1.56) |
| **Mental disorder** |  |  |  |  |  |
| Delirium due to known physiological condition | F05 | 1225 | 1.31 (1.22-1.41) | 1310 | 1.46 (1.36-1.57) |
| Alcohol abuse | F10 | 1474 | 1.37 (1.28-1.46) | 1793 | 1.74 (1.63-1.85) |
| Tobacco abuse | F17 | 4516 | 1.20 (1.16-1.25) | 4615 | 1.28 (1.23-1.33) |
| Depression | F32 | 4803 | 1.58 (1.52-1.64) | 4914 | 1.69 (1.63-1.75) |
| Anxiety | F40 | 3604 | 1.32 (1.27-1.38) | 3720 | 1.42 (1.37-1.48) |
| **Neural system disease** |  |  |  |  |  |
| Epilepsia | G40 | 848 | 1.25 (1.14-1.36) | 837 | 1.28 (1.18-1.40) |
| Sleep disorder | G47 | 2381 | 3.37 (3.16-3.60) | 2739 | 4.06 (3.81-4.33) |
| Diseases in nerves, nerve roots and nerve plexa | G50 | 3916 | 1.57 (1.50-1.63) | 3879 | 1.62 (1.55-1.69) |
| Polyneuropathies | G60 | 1060 | 1.80 (1.66-1.96) | 1197 | 2.12 (1.96-2.30) |
| Cerebral palsy and other paralytic syndromes | G80 | 871 | 1.37 (1.26-1.49) | 893 | 1.47 (1.35-1.60) |
| **Eye and adnexa disease** |  |  |  |  |  |
| Disorder of eyelid | H02 | 1282 | 1.28 (1.20-1.38) | 1195 | 1.25 (1.16-1.34) |
| Disorders of the lens | H25 | 8440 | 1.10 (1.07-1.13) | 8370 | 1.14 (1.11-1.17) |
| Disorders of choroid and retina | H30 | 2924 | 1.27 (1.21-1.33) | 2905 | 1.31 (1.25-1.37) |
| Visual disturbances and blindness | H53 | 1480 | 1.21 (1.14-1.30) | 1548 | 1.32 (1.24-1.41) |
| **Ear dieases** |  |  |  |  |  |
| Other disorders of the ear | H90 | 2291 | 1.13 (1.08-1.19) | 2378 | 1.23 (1.17-1.29) |
| **Cardiovascular disease** |  |  |  |  |  |
| Chronic rheumatic heart disease | I05 | 1595 | 1.26 (1.19-1.35) | 1661 | 1.37 (1.29-1.46) |
| Primary hypertension | I10 | 24363 | 1.87 (1.84-1.91) | 24357 | 2.00 (1.96-2.03) |
| Angina pectoris | I20 | 4515 | 1.62 (1.56-1.68) | 4692 | 1.76 (1.70-1.83) |
| Acute myocardial infarction | I21 | 2519 | 1.52 (1.44-1.60) | 2522 | 1.58 (1.50-1.67) |
| Other ischemic heart disease | I22 | 853 | 1.49 (1.37-1.63) | 907 | 1.66 (1.52-1.81) |
| Chronic ischaemic heart disease | I25 | 7404 | 1.51 (1.47-1.56) | 7773 | 1.66 (1.61-1.71) |
| Embolism and thrombosis | I26 | 2689 | 1.41 (1.34-1.48) | 2872 | 1.57 (1.49-1.65) |
| Non-rheumatic valve disorders | I34 | 2386 | 1.28 (1.22-1.35) | 2472 | 1.39 (1.32-1.46) |
| Atrial fibrillation and flutter | I48 | 6314 | 1.42 (1.37-1.46) | 6348 | 1.49 (1.44-1.54) |
| Other cardiac arrhythmias | I49 | 4417 | 1.28 (1.24-1.33) | 4649 | 1.41 (1.36-1.46) |
| Heart failure | I50 | 3360 | 1.81 (1.73-1.90) | 3568 | 2.01 (1.92-2.11) |
| Complications and ill-defined descriptions of heart disease | I51 | 3200 | 1.62 (1.55-1.70) | 3366 | 1.78 (1.70-1.86) |
| Stroke | I60 | 2110 | 1.25 (1.19-1.32) | 2168 | 1.34 (1.27-1.42) |
| Other cerebrovascular diseases | I65 | 2004 | 1.34 (1.26-1.42) | 2008 | 1.40 (1.32-1.48) |
| Aneurysm and dissection | I71 | 903 | 1.28 (1.17-1.39) | 953 | 1.40 (1.29-1.53) |
| Peripheral vascular disease | I73 | 1324 | 1.17 (1.09-1.25) | 1392 | 1.28 (1.20-1.37) |
| Varicose veins of lower extremities | I83 | 1239 | 1.17 (1.09-1.26) | 1226 | 1.21 (1.13-1.30) |
| Hypotension | I95 | 2912 | 1.20 (1.15-1.26) | 3006 | 1.30 (1.24-1.36) |
| **Respiratory system disease** |  |  |  |  |  |
| Pneumonia | J12 | 5230 | 1.41 (1.36-1.46) | 5395 | 1.51 (1.46-1.57) |
| Other acute Lower respiratory infections | J20 | 2914 | 1.56 (1.49-1.64) | 3009 | 1.68 (1.60-1.76) |
| Chronic obstructive pulmonary disease | J44 | 3730 | 1.38 (1.32-1.44) | 3868 | 1.49 (1.43-1.56) |
| Asthma | J45 | 6023 | 1.46 (1.41-1.50) | 5819 | 1.47 (1.42-1.52) |
| Pleural effusion | J90 | 2715 | 1.26 (1.20-1.33) | 2727 | 1.32 (1.26-1.39) |
| Other diseases of the respiratory system | J95 | 2306 | 1.29 (1.22-1.36) | 2360 | 1.38 (1.31-1.45) |
| Respiratory failure | J96 | 1598 | 1.52 (1.42-1.62) | 1736 | 1.72 (1.62-1.84) |
| **Digestive system disease** |  |  |  |  |  |
| Disease of hard tissue of teeth | K00 | 901 | 1.28 (1.17-1.39) | 897 | 1.32 (1.22-1.44) |
| Oesophagitis | K20 | 1955 | 1.25 (1.18-1.32) | 1895 | 1.26 (1.19-1.33) |
| Gastro-oesophageal reflux disease | K21 | 7934 | 1.39 (1.35-1.43) | 7655 | 1.39 (1.35-1.44) |
| Other diseases of oesophagus | K22 | 2815 | 1.23 (1.18-1.29) | 2721 | 1.24 (1.18-1.30) |
| Gastric ulcer | K25 | 1182 | 1.38 (1.28-1.48) | 1205 | 1.46 (1.36-1.57) |
| Gastritis and duodenitis | K29 | 6863 | 1.23 (1.20-1.27) | 6823 | 1.28 (1.24-1.32) |
| Disease of stomach and duodenum | K31 | 2759 | 1.30 (1.24-1.37) | 2922 | 1.44 (1.37-1.51) |
| Umbilical hernia | K42 | 1294 | 2.63 (2.42-2.85) | 1362 | 2.89 (2.66-3.13) |
| Ventral hernia | K43 | 968 | 1.90 (1.74-2.07) | 1026 | 2.09 (1.92-2.28) |
| Diaphragmatic hernia | K44 | 6864 | 1.31 (1.27-1.35) | 6543 | 1.30 (1.26-1.34) |
| Noninfective gastroenteritis and colitis | K52 | 2592 | 1.26 (1.20-1.33) | 2558 | 1.30 (1.24-1.37) |
| Diverticular disease of intestine | K57 | 10079 | 1.38 (1.35-1.42) | 9938 | 1.43 (1.39-1.46) |
| Irritable bowel syndrome | K58 | 1524 | 1.26 (1.18-1.35) | 1486 | 1.28 (1.20-1.37) |
| Other functional intestinal disorders | K59 | 4440 | 1.21 (1.17-1.26) | 4417 | 1.26 (1.21-1.30) |
| Other diseases of anus and rectum | K62 | 4338 | 1.19 (1.15-1.24) | 4383 | 1.25 (1.21-1.30) |
| Other diseases of intestine | K63 | 5618 | 1.34 (1.29-1.39) | 5767 | 1.44 (1.39-1.49) |
| Haemorrhoids and perianal venous thrombosis | K64 | 3198 | 1.13 (1.08-1.18) | 2971 | 1.10 (1.05-1.15) |
| Other disorders of peritoneum | K66 | 1428 | 1.58 (1.47-1.69) | 1409 | 1.62 (1.51-1.74) |
| Other diseases of liver | K76 | 2236 | 2.26 (2.13-2.40) | 2931 | 3.10 (2.93-3.28) |
| Cholelithiasis | K80 | 3794 | 2.14 (2.05-2.24) | 3795 | 2.24 (2.14-2.35) |
| Disease of gallbladder and biliary tract | K82 | 1423 | 1.80 (1.68-1.94) | 1418 | 1.87 (1.74-2.01) |
| Other disease of digestive system | K92 | 3396 | 1.24 (1.19-1.30) | 3465 | 1.32 (1.27-1.38) |
| **Skin and subcutaneous disease** |  |  |  |  |  |
| Skin and subcutaneous infections | L01 | 3235 | 1.85 (1.76-1.94) | 3375 | 2.02 (1.93-2.12) |
| Dermatitis | L20 | 1365 | 1.37 (1.28-1.47) | 1351 | 1.42 (1.32-1.52) |
| Papulosquamous disorders | L40 | 1029 | 1.51 (1.39-1.63) | 1050 | 1.60 (1.48-1.74) |
| Pressure ulcer | L89 | 904 | 1.45 (1.34-1.59) | 964 | 1.62 (1.49-1.76) |
| Disorder of the skin and subcutaneous tissue | L98 | 1336 | 1.14 (1.07-1.22) | 1315 | 1.17 (1.09-1.25) |
| **Musculoskeletal and connective tissue disease** |  |  |  |  |  |
| Autoimmune arthritis | M05 | 1530 | 1.51 (1.41-1.61) | 1507 | 1.55 (1.45-1.66) |
| Other inflammatory arthritis | M10 | 7681 | 1.86 (1.80-1.91) | 7898 | 2.00 (1.94-2.06) |
| Osteoarthritis | M15 | 14269 | 1.61 (1.58-1.65) | 14126 | 1.67 (1.64-1.71) |
| Other joint disorders | M20 | 6935 | 1.37 (1.33-1.41) | 6724 | 1.38 (1.34-1.43) |
| Systemic connective tissue disorders | M30 | 1103 | 1.24 (1.15-1.34) | 1088 | 1.27 (1.18-1.37) |
| Deforming dorsopathies | M40 | 1211 | 1.35 (1.25-1.45) | 1094 | 1.27 (1.18-1.37) |
| Spondylosis | M47 | 3165 | 1.51 (1.44-1.58) | 3146 | 1.57 (1.50-1.64) |
| Spondylopathy | M48 | 2025 | 1.62 (1.53-1.72) | 2066 | 1.73 (1.63-1.83) |
| Other intervertebral disc disorders | M51 | 2280 | 1.57 (1.48-1.66) | 2222 | 1.59 (1.51-1.68) |
| Dorsalgia | M54 | 4330 | 1.49 (1.44-1.55) | 4430 | 1.60 (1.54-1.66) |
| Disorders of synovium and tendons | M65 | 1850 | 1.34 (1.26-1.42) | 1789 | 1.35 (1.27-1.43) |
| Shoulder lesion | M75 | 2048 | 1.36 (1.29-1.44) | 2055 | 1.43 (1.35-1.51) |
| Other soft tissue disorders, not elsewhere classified | M79 | 3881 | 1.69 (1.62-1.77) | 3983 | 1.82 (1.74-1.90) |
| **Genitourinary system disease** |  |  |  |  |  |
| Obstructive and reflux uropathy | N13 | 1058 | 1.25 (1.15-1.35) | 1112 | 1.37 (1.27-1.47) |
| Acute renal failure | N17 | 4694 | 1.77 (1.70-1.84) | 5140 | 2.03 (1.95-2.11) |
| Chronic kidney disease | N18 | 4469 | 1.94 (1.86-2.02) | 4591 | 2.08 (2.00-2.17) |
| Urolithiasis | N20 | 1432 | 1.47 (1.37-1.57) | 1492 | 1.60 (1.49-1.71) |
| Other disorders of kidney and ureter | N29 | 1405 | 1.52 (1.42-1.63) | 1405 | 1.59 (1.48-1.70) |
| Bladder disorder | N32 | 2303 | 1.18 (1.12-1.24) | 2182 | 1.16 (1.10-1.23) |
| Disorders of urinary system, possibly infection | N39 | 4850 | 1.43 (1.38-1.49) | 5030 | 1.55 (1.50-1.61) |
| Female genital prolapse | N81 | 1356 | 1.26 (1.18-1.35) | 1191 | 1.15 (1.08-1.24) |
| Polyp of female genital tract | N84 | 935 | 1.68 (1.54-1.83) | 981 | 1.83 (1.68-2.00) |
| Menopausal and perimenopausal disorders | N95 | 943 | 1.47 (1.35-1.60) | 933 | 1.51 (1.39-1.65) |
| **Unnatural cause** |  |  |  |  |  |
| Injuries due to external causes | S00 | 7488 | 1.05 (1.02-1.07) | 7400 | 1.08 (1.05-1.11) |
| Complications due to medical treatment | T80 | 5819 | 1.38 (1.33-1.42) | 5947 | 1.47 (1.42-1.52) |
| Falls | W00 | 5112 | 1.11 (1.07-1.14) | 5181 | 1.17 (1.13-1.21) |
| Other external causes of accidental injury | W20 | 2689 | 1.15 (1.10-1.21) | 2660 | 1.19 (1.13-1.25) |
| External causes of morbidity related to medical treatment | Y40 | 7662 | 1.34 (1.30-1.38) | 7737 | 1.42 (1.38-1.46) |
| External causes of morbidity related to other conditions | Y95 | 1221 | 1.41 (1.31-1.51) | 1258 | 1.51 (1.41-1.63) |

A total of 113 disease conditions were confirmed to be significantly associated with MAFLD after Bonferroni correction. Compared with individuals with non-MAFLD, significantly higher HRs (95%CIs without overlap) for MAFLD with or without MBOAT7 rs641738 (C>T) were marked in red.

^*^Combined ICD-10 codes derived from the original ICD-10 code and are displayed in the Additional file 1.

^#^Number of the participants diagnosed with the corresponding diseases.

HR, hazard ratio; CI, confidence interval. MAFLD, Metabolic dysfunction-associated fatty liver disease.

Additional file 1: Table S15. PheWAS using Cox regression was conducted to investigate the relationship of MAFLD with or without MBOAT7 rs641738 (C>T) and causes of death.

| **Causes of death** | **MAFLD without MBOAT7 rs641738 (C>T) vs.**  **Non-MAFLD (N = 246,515)** | | **MAFLD with MBOAT7**  **rs641738 (C>T) vs.**  **Non-MAFLD (N = 243,394)** | |
| --- | --- | --- | --- | --- |
|  | **No.^*^** | **HR (95% CI)** | **No.^*^** | **HR (95% CI)** |
| Cardiovascular death | 2892 | 1.54 (1.47-1.62) | 3133 | 1.74 (1.66-1.83) |
| Digestive system death | 534 | 1.35 (1.21-1.50) | 875 | 2.30 (2.09-2.53) |
| Endocrine system death | 918 | 2.89 (2.61-3.20) | 1089 | 3.58 (3.24-3.95) |
| Genitourinary system death | 579 | 1.93 (1.72-2.16) | 624 | 2.17 (1.94-2.43) |
| Infectious and parasitic death | 495 | 2.05 (1.86-2.26) | 587 | 2.26 (2.21-2.30) |
| Malignant neoplasms death | 3760 | 1.24 (1.19-1.29) | 3728 | 1.28 (1.23-1.33) |
| Respiratory system death | 1760 | 1.15 (1.09-1.22) | 1846 | 1.26 (1.19-1.34) |
| Unnatural cause death | 559 | 1.22 (1.10-1.36) | 524 | 1.20 (1.07-1.33) |

After Bonferroni correction, a total of 8 death causes were significant associated with MAFLD. Compared with individuals with non-MAFLD, significantly higher HRs (95%CIs without overlap) for MAFLD with or without MBOAT7 rs641738 (C>T) were marked in red.

**^*^**Number of the participants died due to the corresponding causes, including primary and secondary causes of death.

HR, hazard ratio; CI, confidence interval. MAFLD, Metabolic dysfunction-associated fatty liver disease.

Additional file 1: Table S16. PheWAS using Cox regression was conducted to investigate the association of MAFLD with or without GCKR rs1260326 (C>T) and 490 subsequent disease conditions.

| **Medical conditions** | **Code^*^** | **MAFLD without GCKR rs1260326 vs. Non-MAFLD (N = 202,282)** | | **MAFLD with GCKR rs1260326**  **vs. Non-MAFLD (N = 280,627)** | |
| --- | --- | --- | --- | --- | --- |
|  |  | **No.^#^** | **HR (95% CI)** | **No.^#^** | **HR (95% CI)** |
| **Infectious and parasitic** |  |  |  |  |  |
| Infectious gastroenteritis and colitis | A09 | 2019 | 1.37 (1.30-1.44) | 5317 | 1.42 (1.36-1.47) |
| Sepsis | A41 | 1536 | 1.39 (1.31-1.48) | 4382 | 1.56 (1.50-1.63) |
| Mycoses | B49 | 657 | 1.18 (1.08-1.29) | 1870 | 1.33 (1.24-1.41) |
| Bacterial infectious agents | B96 | 2093 | 1.35 (1.28-1.42) | 6095 | 1.54 (1.49-1.60) |
| **Malignant neoplasms** |  |  |  |  |  |
| Colon cancer | C18 | 504 | 1.18 (1.07-1.30) | 1416 | 1.30 (1.21-1.40) |
| Metastatic cancer | C77 | 1964 | 1.17 (1.11-1.23) | 5256 | 1.23 (1.19-1.28) |
| **Benign neoplasms** |  |  |  |  |  |
| Benign tumors | D10 | 4926 | 1.26 (1.22-1.30) | 12940 | 1.30 (1.27-1.33) |
| **Blood system** |  |  |  |  |  |
| Iron deficiency anaemia | D50 | 1798 | 1.28 (1.22-1.36) | 5019 | 1.41 (1.36-1.47) |
| Other anaemias | D51 | 2500 | 1.21 (1.15-1.26) | 7094 | 1.35 (1.31-1.40) |
| **Endocrine system disease** |  |  |  |  |  |
| Hypothyroid conditions | E00 | 2137 | 1.44 (1.37-1.51) | 5601 | 1.48 (1.43-1.54) |
| Diabetes | E10 | 5045 | 3.83 (3.69-3.99) | 14564 | 4.40 (4.26-4.54) |
| Malnutrition | E40 | 837 | 1.27 (1.17-1.37) | 2485 | 1.48 (1.40-1.56) |
| Obesity | E66 | 5465 | 7.68 (7.34-8.04) | 16047 | 8.97 (8.61-9.34) |
| Disorders of lipoprotein metabolism and other lipidaemias | E78 | 7312 | 1.72 (1.67-1.76) | 19353 | 1.80 (1.76-1.84) |
| Disorders of mineral metabolism | E83 | 744 | 1.29 (1.19-1.41) | 2240 | 1.53 (1.45-1.63) |
| Volume depletion | E86 | 1012 | 1.32 (1.23-1.42) | 2862 | 1.47 (1.39-1.55) |
| Other disorders of fluid, electrolyte and acid-base balance | E87 | 2145 | 1.32 (1.26-1.39) | 6062 | 1.47 (1.42-1.53) |
| **Mental disorder** |  |  |  |  |  |
| Delirium due to known physiological condition | F05 | 670 | 1.30 (1.19-1.42) | 1865 | 1.42 (1.33-1.52) |
| Alcohol abuse | F10 | 816 | 1.37 (1.26-1.49) | 2451 | 1.62 (1.53-1.72) |
| Tobacco abuse | F17 | 2470 | 1.19 (1.13-1.24) | 6661 | 1.26 (1.22-1.30) |
| Depression | F32 | 2678 | 1.59 (1.52-1.67) | 7039 | 1.65 (1.59-1.70) |
| Anxiety | F40 | 1936 | 1.28 (1.22-1.35) | 5388 | 1.41 (1.35-1.46) |
| **Neural system disease** |  |  |  |  |  |
| Epilepsia | G40 | 480 | 1.28 (1.15-1.42) | 1205 | 1.26 (1.16-1.36) |
| Sleep disorder | G47 | 1288 | 3.30 (3.06-3.56) | 3832 | 3.87 (3.64-4.11) |
| Diseases in nerves, nerve roots and nerve plexa | G50 | 2155 | 1.56 (1.48-1.64) | 5640 | 1.60 (1.54-1.67) |
| Polyneuropathies | G60 | 586 | 1.80 (1.63-1.99) | 1671 | 2.02 (1.88-2.18) |
| Cerebral palsy and other paralytic syndromes | G80 | 493 | 1.40 (1.26-1.56) | 1271 | 1.42 (1.32-1.54) |
| **Eye and adnexa disease** |  |  |  |  |  |
| Disorder of eyelid | H02 | 701 | 1.27 (1.17-1.38) | 1776 | 1.26 (1.19-1.35) |
| Disorders of the lens | H25 | 4617 | 1.09 (1.06-1.13) | 12193 | 1.14 (1.11-1.16) |
| Disorders of choroid and retina | H30 | 1570 | 1.23 (1.16-1.30) | 4259 | 1.31 (1.26-1.37) |
| Visual disturbances and blindness | H53 | 814 | 1.21 (1.12-1.31) | 2214 | 1.29 (1.22-1.37) |
| **Ear dieases** |  |  |  |  |  |
| Other disorders of the ear | H90 | 1273 | 1.14 (1.07-1.21) | 3396 | 1.19 (1.14-1.25) |
| **Cardiovascular disease** |  |  |  |  |  |
| Chronic rheumatic heart disease | I05 | 900 | 1.29 (1.20-1.39) | 2356 | 1.33 (1.26-1.40) |
| Primary hypertension | I10 | 13353 | 1.86 (1.82-1.90) | 35367 | 1.96 (1.93-1.99) |
| Angina pectoris | I20 | 2551 | 1.66 (1.58-1.74) | 6656 | 1.70 (1.64-1.76) |
| Acute myocardial infarction | I21 | 1408 | 1.53 (1.44-1.63) | 3633 | 1.56 (1.48-1.63) |
| Other ischemic heart disease | I22 | 491 | 1.56 (1.40-1.73) | 1269 | 1.58 (1.46-1.71) |
| Chronic ischaemic heart disease | I25 | 4154 | 1.53 (1.48-1.59) | 11023 | 1.61 (1.56-1.65) |
| Embolism and thrombosis | I26 | 1478 | 1.40 (1.32-1.48) | 4083 | 1.52 (1.45-1.59) |
| Non-rheumatic valve disorders | I34 | 1289 | 1.26 (1.18-1.34) | 3569 | 1.37 (1.31-1.43) |
| Atrial fibrillation and flutter | I48 | 3431 | 1.39 (1.34-1.45) | 9231 | 1.48 (1.43-1.52) |
| Other cardiac arrhythmias | I49 | 2488 | 1.31 (1.25-1.37) | 6578 | 1.36 (1.32-1.41) |
| Heart failure | I50 | 1855 | 1.81 (1.71-1.91) | 5073 | 1.95 (1.87-2.03) |
| Complications and ill-defined descriptions of heart disease | I51 | 1758 | 1.61 (1.52-1.70) | 4808 | 1.73 (1.66-1.81) |
| Stroke | I60 | 1185 | 1.27 (1.19-1.36) | 3093 | 1.31 (1.24-1.37) |
| Other cerebrovascular diseases | I65 | 1109 | 1.34 (1.25-1.44) | 2903 | 1.38 (1.31-1.45) |
| Aneurysm and dissection | I71 | 514 | 1.31 (1.19-1.45) | 1342 | 1.35 (1.25-1.45) |
| Peripheral vascular disease | I73 | 697 | 1.11 (1.02-1.21) | 2019 | 1.27 (1.20-1.35) |
| Varicose veins of lower extremities | I83 | 685 | 1.17 (1.07-1.28) | 1780 | 1.20 (1.12-1.28) |
| Hypotension | I95 | 1604 | 1.20 (1.13-1.27) | 4314 | 1.27 (1.22-1.32) |
| **Respiratory system disease** |  |  |  |  |  |
| Pneumonia | J12 | 2851 | 1.38 (1.33-1.45) | 7774 | 1.49 (1.44-1.54) |
| Other acute Lower respiratory infections | J20 | 1630 | 1.58 (1.49-1.67) | 4293 | 1.64 (1.57-1.71) |
| Chronic obstructive pulmonary disease | J44 | 2061 | 1.38 (1.31-1.45) | 5537 | 1.46 (1.40-1.51) |
| Asthma | J45 | 3147 | 1.37 (1.32-1.43) | 8695 | 1.49 (1.45-1.54) |
| Pleural effusion | J90 | 1453 | 1.22 (1.15-1.30) | 3989 | 1.32 (1.26-1.38) |
| Other diseases of the respiratory system | J95 | 1246 | 1.26 (1.18-1.35) | 3420 | 1.36 (1.30-1.43) |
| Respiratory failure | J96 | 849 | 1.46 (1.35-1.58) | 2485 | 1.68 (1.59-1.78) |
| **Digestive system disease** |  |  |  |  |  |
| Disease of hard tissue of teeth | K00 | 471 | 1.21 (1.09-1.34) | 1327 | 1.34 (1.24-1.44) |
| Oesophagitis | K20 | 1032 | 1.19 (1.11-1.27) | 2818 | 1.28 (1.21-1.34) |
| Gastro-oesophageal reflux disease | K21 | 4393 | 1.39 (1.34-1.44) | 11196 | 1.39 (1.35-1.43) |
| Other diseases of oesophagus | K22 | 1590 | 1.26 (1.19-1.33) | 3946 | 1.23 (1.18-1.28) |
| Gastric ulcer | K25 | 630 | 1.33 (1.21-1.45) | 1757 | 1.45 (1.36-1.55) |
| Gastritis and duodenitis | K29 | 3736 | 1.21 (1.17-1.26) | 9950 | 1.27 (1.24-1.31) |
| Disease of stomach and duodenum | K31 | 1443 | 1.23 (1.16-1.31) | 4238 | 1.42 (1.37-1.49) |
| Umbilical hernia | K42 | 695 | 2.55 (2.32-2.81) | 1961 | 2.83 (2.62-3.06) |
| Ventral hernia | K43 | 497 | 1.76 (1.58-1.96) | 1497 | 2.08 (1.92-2.26) |
| Diaphragmatic hernia | K44 | 3837 | 1.33 (1.28-1.38) | 9570 | 1.30 (1.26-1.34) |
| Noninfective gastroenteritis and colitis | K52 | 1347 | 1.19 (1.12-1.26) | 3803 | 1.32 (1.26-1.38) |
| Diverticular disease of intestine | K57 | 5488 | 1.36 (1.32-1.40) | 14529 | 1.42 (1.39-1.45) |
| Irritable bowel syndrome | K58 | 851 | 1.27 (1.18-1.38) | 2159 | 1.27 (1.20-1.35) |
| Other functional intestinal disorders | K59 | 2455 | 1.21 (1.16-1.27) | 6402 | 1.24 (1.20-1.28) |
| Other diseases of anus and rectum | K62 | 2446 | 1.22 (1.16-1.27) | 6275 | 1.22 (1.18-1.27) |
| Other diseases of intestine | K63 | 3120 | 1.35 (1.29-1.40) | 8265 | 1.40 (1.36-1.45) |
| Haemorrhoids and perianal venous thrombosis | K64 | 1816 | 1.16 (1.10-1.23) | 4353 | 1.10 (1.05-1.14) |
| Other disorders of peritoneum | K66 | 746 | 1.49 (1.37-1.62) | 2091 | 1.64 (1.54-1.75) |
| Other diseases of liver | K76 | 1161 | 2.12 (1.97-2.28) | 4006 | 2.89 (2.74-3.05) |
| Cholelithiasis | K80 | 2009 | 2.05 (1.94-2.17) | 5580 | 2.25 (2.15-2.34) |
| Disease of gallbladder and biliary tract | K82 | 731 | 1.67 (1.53-1.83) | 2110 | 1.90 (1.78-2.03) |
| Other disease of digestive system | K92 | 1869 | 1.24 (1.17-1.30) | 4992 | 1.30 (1.25-1.35) |
| **Skin and subcutaneous disease** |  |  |  |  |  |
| Skin and subcutaneous infections | L01 | 1769 | 1.83 (1.72-1.93) | 4841 | 1.97 (1.89-2.06) |
| Dermatitis | L20 | 745 | 1.36 (1.25-1.48) | 1971 | 1.41 (1.33-1.50) |
| Papulosquamous disorders | L40 | 564 | 1.49 (1.35-1.65) | 1515 | 1.58 (1.47-1.70) |
| Pressure ulcer | L89 | 475 | 1.38 (1.24-1.54) | 1393 | 1.60 (1.48-1.72) |
| Disorder of the skin and subcutaneous tissue | L98 | 744 | 1.15 (1.06-1.25) | 1907 | 1.16 (1.09-1.23) |
| **Musculoskeletal and connective tissue disease** |  |  |  |  |  |
| Autoimmune arthritis | M05 | 824 | 1.47 (1.36-1.59) | 2213 | 1.55 (1.46-1.65) |
| Other inflammatory arthritis | M10 | 4192 | 1.83 (1.76-1.90) | 11387 | 1.96 (1.91-2.02) |
| Osteoarthritis | M15 | 7770 | 1.59 (1.54-1.63) | 20625 | 1.67 (1.63-1.70) |
| Other joint disorders | M20 | 3735 | 1.33 (1.28-1.38) | 9924 | 1.39 (1.35-1.43) |
| Systemic connective tissue disorders | M30 | 588 | 1.19 (1.09-1.31) | 1603 | 1.28 (1.20-1.37) |
| Deforming dorsopathies | M40 | 679 | 1.37 (1.25-1.49) | 1626 | 1.29 (1.20-1.38) |
| Spondylosis | M47 | 1735 | 1.50 (1.42-1.59) | 4576 | 1.56 (1.49-1.62) |
| Spondylopathy | M48 | 1120 | 1.62 (1.51-1.74) | 2971 | 1.69 (1.61-1.79) |
| Other intervertebral disc disorders | M51 | 1287 | 1.60 (1.50-1.71) | 3215 | 1.57 (1.50-1.65) |
| Dorsalgia | M54 | 2410 | 1.50 (1.43-1.58) | 6350 | 1.56 (1.51-1.62) |
| Disorders of synovium and tendons | M65 | 992 | 1.30 (1.21-1.39) | 2647 | 1.36 (1.29-1.43) |
| Shoulder lesion | M75 | 1089 | 1.31 (1.22-1.41) | 3014 | 1.43 (1.36-1.50) |
| Other soft tissue disorders, not elsewhere classified | M79 | 2158 | 1.70 (1.62-1.79) | 5706 | 1.77 (1.71-1.84) |
| **Genitourinary system disease** |  |  |  |  |  |
| Obstructive and reflux uropathy | N13 | 562 | 1.20 (1.09-1.32) | 1608 | 1.35 (1.26-1.44) |
| Acute renal failure | N17 | 2478 | 1.69 (1.61-1.77) | 7356 | 1.98 (1.91-2.05) |
| Chronic kidney disease | N18 | 2407 | 1.89 (1.80-1.98) | 6653 | 2.05 (1.98-2.13) |
| Urolithiasis | N20 | 791 | 1.47 (1.35-1.60) | 2133 | 1.56 (1.46-1.66) |
| Other disorders of kidney and ureter | N29 | 773 | 1.52 (1.40-1.65) | 2037 | 1.57 (1.48-1.67) |
| Bladder disorder | N32 | 1204 | 1.11 (1.04-1.19) | 3281 | 1.19 (1.14-1.25) |
| Disorders of urinary system, possibly infection | N39 | 2643 | 1.41 (1.35-1.48) | 7237 | 1.52 (1.47-1.57) |
| Female genital prolapse | N81 | 735 | 1.24 (1.14-1.35) | 1812 | 1.20 (1.13-1.28) |
| Polyp of female genital tract | N84 | 481 | 1.56 (1.40-1.73) | 1435 | 1.83 (1.69-1.98) |
| Menopausal and perimenopausal disorders | N95 | 514 | 1.45 (1.31-1.60) | 1362 | 1.51 (1.40-1.63) |
| **Unnatural cause** |  |  |  |  |  |
| Injuries due to external causes | S00 | 4056 | 1.02 (0.99-1.06) | 10832 | 1.08 (1.05-1.10) |
| Complications due to medical treatment | T80 | 3181 | 1.36 (1.31-1.42) | 8585 | 1.45 (1.40-1.49) |
| Falls | W00 | 2779 | 1.09 (1.04-1.13) | 7514 | 1.16 (1.12-1.19) |
| Other external causes of accidental injury | W20 | 1444 | 1.12 (1.05-1.18) | 3905 | 1.19 (1.14-1.24) |
| External causes of morbidity related to medical treatment | Y40 | 4255 | 1.35 (1.30-1.40) | 11144 | 1.39 (1.36-1.43) |
| External causes of morbidity related to other conditions | Y95 | 657 | 1.37 (1.25-1.50) | 1822 | 1.49 (1.40-1.60) |

A total of 113 disease conditions were confirmed to be significantly associated with MAFLD after Bonferroni correction. Compared with individuals with non-MAFLD, significantly higher HRs (95%CIs without overlap) for MAFLD with or without GCKR rs1260326 (C>T) were marked in red.

^*^Combined ICD-10 codes derived from the original ICD-10 code and are displayed in the Additional file 1.

^#^Number of the participants diagnosed with the corresponding diseases.

HR, hazard ratio; CI, confidence interval. MAFLD, Metabolic dysfunction-associated fatty liver disease.

Additional file 1: Table S17. PheWAS using Cox regression was conducted to investigate the relationship of MAFLD with or without GCKR rs1260326 (C>T) and causes of death.

| **Causes of death** | **MAFLD without GCKR rs1260326 vs. Non-MAFLD (N = 202,282)** | | **MAFLD with GCKR rs1260326**  **vs. Non-MAFLD (N = 280,627)** | |
| --- | --- | --- | --- | --- |
|  | **No.^*^** | **HR (95% CI)** | **No.^*^** | **HR (95% CI)** |
| Cardiovascular death | 1573 | 1.52(1.43-1.61) | 4452 | 1.69(1.62-1.77) |
| Digestive system death | 272 | 1.24(1.08-1.42) | 1137 | 2.04(1.86-2.23) |
| Endocrine system death | 489 | 2.78(2.47-3.13) | 1518 | 3.40(3.10-3.73) |
| Genitourinary system death | 309 | 1.86(1.62-2.14) | 894 | 2.12(1.91-2.35) |
| Infectious and parasitic death | 465 | 2.02(1.83-2.23) | 693 | 2.81(2.66-2.98) |
| Malignant neoplasms death | 1986 | 1.18(1.12-1.24) | 5502 | 1.29(1.24-1.33) |
| Respiratory system death | 968 | 1.15(1.07-1.23) | 2638 | 1.23(1.17-1.30) |
| Unnatural cause death | 283 | 1.12(0.98-1.28) | 800 | 1.25(1.13-1.37) |

After Bonferroni correction, a total of 8 death causes were significant associated with MAFLD. Compared with individuals with non-MAFLD, significantly higher HRs (95%CIs without overlap) for MAFLD with or without GCKR rs1260326 (C>T) were marked in red.

**^*^**Number of the participants died due to the corresponding causes, including primary and secondary causes of death.

HR, hazard ratio; CI, confidence interval. MAFLD, Metabolic dysfunction-associated fatty liver disease.

Additional file 1: Table S18. PheWAS using Cox regression was conducted to investigate the association of MAFLD with or without TM6SF2 rs58542926 (C>T) and subsequent 490 disease conditions.

| **Medical conditions** | **Code^*^** | **MAFLD without TM6SF2 rs58542926 vs.**  **Non-MAFLD (N = 298,639)** | | **MAFLD with TM6SF2 rs58542926 vs.**  **Non-MAFLD (N = 191,270)** | |
| --- | --- | --- | --- | --- | --- |
|  |  | **No.^#^** | **HR (95% CI)** | **No.^#^** | **HR (95% CI)** |
| **Infectious and parasitic** |  |  |  |  |  |
| Infectious gastroenteritis and colitis | A09 | 6057 | 1.40 (1.35-1.45) | 1279 | 1.43 (1.35-1.52) |
| Sepsis | A41 | 4877 | 1.51 (1.44-1.57) | 1041 | 1.56 (1.46-1.67) |
| Mycoses | B49 | 2080 | 1.28 (1.20-1.36) | 447 | 1.33 (1.20-1.47) |
| Bacterial infectious agents | B96 | 6755 | 1.48 (1.43-1.53) | 1433 | 1.52 (1.44-1.61) |
| **Malignant neoplasms** |  |  |  |  |  |
| Colon cancer | C18 | 1576 | 1.25 (1.17-1.35) | 344 | 1.33 (1.18-1.49) |
| Metastatic cancer | C77 | 5960 | 1.21 (1.17-1.25) | 1260 | 1.24 (1.17-1.32) |
| **Benign neoplasms** |  |  |  |  |  |
| Benign tumors | D10 | 14758 | 1.28 (1.25-1.31) | 3108 | 1.31 (1.26-1.36) |
| **Blood system** |  |  |  |  |  |
| Iron deficiency anaemia | D50 | 5596 | 1.36 (1.31-1.41) | 1221 | 1.44 (1.36-1.54) |
| Other anaemias | D51 | 7879 | 1.30 (1.26-1.34) | 1715 | 1.37 (1.30-1.44) |
| **Endocrine system disease** |  |  |  |  |  |
| Hypothyroid conditions | E00 | 6364 | 1.46 (1.40-1.51) | 1374 | 1.53 (1.44-1.62) |
| Diabetes | E10 | 16000 | 4.17 (4.04-4.30) | 3609 | 4.59 (4.40-4.79) |
| Malnutrition | E40 | 2744 | 1.41 (1.34-1.49) | 578 | 1.45 (1.32-1.58) |
| Obesity | E66 | 17553 | 8.45 (8.11-8.80) | 3959 | 9.36 (8.92-9.83) |
| Disorders of lipoprotein metabolism and other lipidaemias | E78 | 21884 | 1.75 (1.72-1.79) | 4781 | 1.87 (1.81-1.93) |
| Disorders of mineral metabolism | E83 | 2459 | 1.46 (1.37-1.55) | 525 | 1.51 (1.37-1.66) |
| Volume depletion | E86 | 3156 | 1.40 (1.33-1.48) | 718 | 1.55 (1.43-1.68) |
| Other disorders of fluid, electrolyte and acid-base balance | E87 | 6757 | 1.42 (1.37-1.47) | 1450 | 1.48 (1.40-1.57) |
| **Mental disorder** |  |  |  |  |  |
| Delirium due to known physiological condition | F05 | 2085 | 1.37 (1.29-1.46) | 450 | 1.44 (1.30-1.60) |
| Alcohol abuse | F10 | 2652 | 1.52 (1.43-1.61) | 615 | 1.71 (1.56-1.87) |
| Tobacco abuse | F17 | 7563 | 1.24 (1.20-1.28) | 1568 | 1.24 (1.18-1.31) |
| Depression | F32 | 7924 | 1.60 (1.55-1.66) | 1793 | 1.77 (1.68-1.86) |
| Anxiety | F40 | 6017 | 1.36 (1.31-1.41) | 1307 | 1.43 (1.35-1.52) |
| **Neural system disease** |  |  |  |  |  |
| Epilepsia | G40 | 1376 | 1.24 (1.15-1.34) | 309 | 1.36 (1.20-1.54) |
| Sleep disorder | G47 | 4175 | 3.65 (3.43-3.87) | 945 | 4.02 (3.70-4.36) |
| Diseases in nerves, nerve roots and nerve plexa | G50 | 6459 | 1.59 (1.53-1.65) | 1336 | 1.60 (1.51-1.70) |
| Polyneuropathies | G60 | 1841 | 1.93 (1.79-2.07) | 416 | 2.12 (1.89-2.37) |
| Cerebral palsy and other paralytic syndromes | G80 | 1447 | 1.40 (1.30-1.51) | 317 | 1.49 (1.32-1.68) |
| **Eye and adnexa disease** |  |  |  |  |  |
| Disorder of eyelid | H02 | 2056 | 1.27 (1.19-1.35) | 421 | 1.26 (1.13-1.40) |
| Disorders of the lens | H25 | 13864 | 1.12 (1.09-1.14) | 2946 | 1.15 (1.11-1.20) |
| Disorders of choroid and retina | H30 | 4807 | 1.28 (1.23-1.34) | 1022 | 1.32 (1.24-1.41) |
| Visual disturbances and blindness | H53 | 2504 | 1.26 (1.20-1.34) | 524 | 1.28 (1.17-1.41) |
| **Ear dieases** |  |  |  |  |  |
| Other disorders of the ear | H90 | 3860 | 1.18 (1.12-1.23) | 809 | 1.20 (1.11-1.29) |
| **Cardiovascular disease** |  |  |  |  |  |
| Chronic rheumatic heart disease | I05 | 2684 | 1.31 (1.24-1.38) | 572 | 1.35 (1.24-1.48) |
| Primary hypertension | I10 | 40132 | 1.92 (1.89-1.95) | 8588 | 2.02 (1.97-2.07) |
| Angina pectoris | I20 | 7588 | 1.68 (1.62-1.74) | 1619 | 1.74 (1.65-1.84) |
| Acute myocardial infarction | I21 | 4213 | 1.56 (1.49-1.64) | 828 | 1.49 (1.38-1.61) |
| Other ischemic heart disease | I22 | 1456 | 1.57 (1.45-1.70) | 304 | 1.59 (1.40-1.81) |
| Chronic ischaemic heart disease | I25 | 12504 | 1.57 (1.53-1.62) | 2673 | 1.64 (1.57-1.71) |
| Embolism and thrombosis | I26 | 4553 | 1.47 (1.40-1.53) | 1008 | 1.58 (1.47-1.69) |
| Non-rheumatic valve disorders | I34 | 4040 | 1.34 (1.28-1.40) | 818 | 1.31 (1.22-1.42) |
| Atrial fibrillation and flutter | I48 | 10450 | 1.45 (1.41-1.49) | 2212 | 1.49 (1.42-1.56) |
| Other cardiac arrhythmias | I49 | 7452 | 1.33 (1.29-1.38) | 1614 | 1.40 (1.33-1.48) |
| Heart failure | I50 | 5733 | 1.91 (1.83-1.99) | 1195 | 1.93 (1.81-2.06) |
| Complications and ill-defined descriptions of heart disease | I51 | 5414 | 1.69 (1.62-1.76) | 1152 | 1.74 (1.63-1.86) |
| Stroke | I60 | 3513 | 1.28 (1.22-1.35) | 765 | 1.36 (1.25-1.47) |
| Other cerebrovascular diseases | I65 | 3320 | 1.37 (1.30-1.44) | 692 | 1.38 (1.27-1.50) |
| Aneurysm and dissection | I71 | 1539 | 1.34 (1.25-1.44) | 317 | 1.34 (1.18-1.51) |
| Peripheral vascular disease | I73 | 2244 | 1.22 (1.15-1.29) | 472 | 1.25 (1.13-1.38) |
| Varicose veins of lower extremities | I83 | 2038 | 1.19 (1.12-1.26) | 427 | 1.21 (1.09-1.34) |
| Hypotension | I95 | 4883 | 1.24 (1.19-1.29) | 1035 | 1.28 (1.20-1.37) |
| **Respiratory system disease** |  |  |  |  |  |
| Pneumonia | J12 | 8791 | 1.46 (1.41-1.50) | 1834 | 1.47 (1.40-1.55) |
| Other acute Lower respiratory infections | J20 | 4850 | 1.60 (1.53-1.67) | 1073 | 1.72 (1.61-1.84) |
| Chronic obstructive pulmonary disease | J44 | 6206 | 1.41 (1.36-1.46) | 1392 | 1.54 (1.45-1.63) |
| Asthma | J45 | 9737 | 1.45 (1.41-1.49) | 2105 | 1.52 (1.45-1.59) |
| Pleural effusion | J90 | 4519 | 1.30 (1.24-1.35) | 923 | 1.28 (1.19-1.38) |
| Other diseases of the respiratory system | J95 | 3821 | 1.32 (1.26-1.38) | 845 | 1.41 (1.31-1.52) |
| Respiratory failure | J96 | 2780 | 1.63 (1.54-1.72) | 554 | 1.57 (1.43-1.73) |
| **Digestive system disease** |  |  |  |  |  |
| Disease of hard tissue of teeth | K00 | 1531 | 1.34 (1.24-1.44) | 267 | 1.13 (0.99-1.29) |
| Oesophagitis | K20 | 3219 | 1.26 (1.20-1.33) | 631 | 1.20 (1.10-1.31) |
| Gastro-oesophageal reflux disease | K21 | 12915 | 1.39 (1.36-1.43) | 2674 | 1.39 (1.34-1.45) |
| Other diseases of oesophagus | K22 | 4568 | 1.23 (1.18-1.28) | 968 | 1.26 (1.18-1.36) |
| Gastric ulcer | K25 | 1999 | 1.43 (1.34-1.53) | 388 | 1.35 (1.21-1.50) |
| Gastritis and duodenitis | K29 | 11312 | 1.25 (1.22-1.29) | 2374 | 1.27 (1.22-1.33) |
| Disease of stomach and duodenum | K31 | 4656 | 1.35 (1.30-1.41) | 1025 | 1.45 (1.35-1.55) |
| Umbilical hernia | K42 | 2197 | 2.75 (2.55-2.96) | 459 | 2.79 (2.49-3.11) |
| Ventral hernia | K43 | 1608 | 1.94 (1.79-2.10) | 386 | 2.26 (2.01-2.54) |
| Diaphragmatic hernia | K44 | 11169 | 1.31 (1.28-1.35) | 2238 | 1.27 (1.22-1.33) |
| Noninfective gastroenteritis and colitis | K52 | 4228 | 1.27 (1.22-1.33) | 922 | 1.34 (1.25-1.44) |
| Diverticular disease of intestine | K57 | 16529 | 1.40 (1.37-1.43) | 3488 | 1.43 (1.38-1.49) |
| Irritable bowel syndrome | K58 | 2449 | 1.25 (1.18-1.32) | 561 | 1.39 (1.27-1.52) |
| Other functional intestinal disorders | K59 | 7281 | 1.22 (1.18-1.26) | 1576 | 1.28 (1.22-1.36) |
| Other diseases of anus and rectum | K62 | 7242 | 1.22 (1.18-1.26) | 1479 | 1.21 (1.15-1.28) |
| Other diseases of intestine | K63 | 9455 | 1.39 (1.35-1.43) | 1930 | 1.38 (1.31-1.44) |
| Haemorrhoids and perianal venous thrombosis | K64 | 5116 | 1.12 (1.07-1.16) | 1053 | 1.11 (1.04-1.19) |
| Other disorders of peritoneum | K66 | 2339 | 1.59 (1.50-1.69) | 498 | 1.64 (1.49-1.82) |
| Other diseases of liver | K76 | 4101 | 2.55 (2.42-2.70) | 1066 | 3.24 (3.00-3.49) |
| Cholelithiasis | K80 | 6252 | 2.18 (2.09-2.27) | 1337 | 2.26 (2.12-2.41) |
| Disease of gallbladder and biliary tract | K82 | 2315 | 1.80 (1.69-1.92) | 526 | 1.99 (1.80-2.20) |
| Other disease of digestive system | K92 | 5692 | 1.28 (1.23-1.33) | 1169 | 1.27 (1.20-1.36) |
| **Skin and subcutaneous disease** |  |  |  |  |  |
| Skin and subcutaneous infections | L01 | 5457 | 1.92 (1.84-2.01) | 1153 | 1.97 (1.85-2.11) |
| Dermatitis | L20 | 2224 | 1.38 (1.30-1.46) | 492 | 1.48 (1.34-1.63) |
| Papulosquamous disorders | L40 | 1705 | 1.54 (1.43-1.65) | 374 | 1.64 (1.46-1.84) |
| Pressure ulcer | L89 | 1524 | 1.51 (1.40-1.63) | 344 | 1.66 (1.47-1.87) |
| Disorder of the skin and subcutaneous tissue | L98 | 2216 | 1.16 (1.10-1.23) | 435 | 1.11 (1.00-1.23) |
| **Musculoskeletal and connective tissue disease** |  |  |  |  |  |
| Autoimmune arthritis | M05 | 2469 | 1.50 (1.41-1.59) | 568 | 1.67 (1.53-1.84) |
| Other inflammatory arthritis | M10 | 12768 | 1.90 (1.85-1.95) | 2811 | 2.04 (1.95-2.13) |
| Osteoarthritis | M15 | 23368 | 1.63 (1.60-1.66) | 5027 | 1.71 (1.66-1.77) |
| Other joint disorders | M20 | 11286 | 1.37 (1.33-1.41) | 2373 | 1.40 (1.34-1.46) |
| Systemic connective tissue disorders | M30 | 1761 | 1.22 (1.14-1.30) | 430 | 1.44 (1.30-1.60) |
| Deforming dorsopathies | M40 | 1890 | 1.30 (1.21-1.38) | 415 | 1.38 (1.24-1.54) |
| Spondylosis | M47 | 5163 | 1.52 (1.46-1.58) | 1148 | 1.64 (1.54-1.75) |
| Spondylopathy | M48 | 3359 | 1.66 (1.57-1.75) | 732 | 1.75 (1.61-1.90) |
| Other intervertebral disc disorders | M51 | 3739 | 1.58 (1.51-1.66) | 763 | 1.57 (1.45-1.70) |
| Dorsalgia | M54 | 7181 | 1.53 (1.47-1.58) | 1579 | 1.63 (1.54-1.72) |
| Disorders of synovium and tendons | M65 | 3003 | 1.34 (1.27-1.41) | 636 | 1.37 (1.26-1.50) |
| Shoulder lesion | M75 | 3384 | 1.39 (1.32-1.46) | 719 | 1.43 (1.32-1.55) |
| Other soft tissue disorders, not elsewhere classified | M79 | 6482 | 1.74 (1.68-1.81) | 1382 | 1.80 (1.70-1.92) |
| **Genitourinary system disease** |  |  |  |  |  |
| Obstructive and reflux uropathy | N13 | 1781 | 1.29 (1.21-1.38) | 389 | 1.37 (1.23-1.53) |
| Acute renal failure | N17 | 8105 | 1.89 (1.82-1.95) | 1729 | 1.96 (1.85-2.06) |
| Chronic kidney disease | N18 | 7444 | 1.99 (1.92-2.06) | 1616 | 2.10 (1.98-2.22) |
| Urolithiasis | N20 | 2408 | 1.52 (1.43-1.62) | 516 | 1.58 (1.44-1.74) |
| Other disorders of kidney and ureter | N29 | 2297 | 1.53 (1.44-1.63) | 513 | 1.66 (1.51-1.83) |
| Bladder disorder | N32 | 3718 | 1.17 (1.12-1.22) | 767 | 1.17 (1.08-1.27) |
| Disorders of urinary system, possibly infection | N39 | 8153 | 1.48 (1.44-1.53) | 1727 | 1.52 (1.45-1.61) |
| Female genital prolapse | N81 | 2133 | 1.22 (1.15-1.30) | 414 | 1.15 (1.03-1.28) |
| Polyp of female genital tract | N84 | 1566 | 1.73 (1.60-1.87) | 350 | 1.87 (1.66-2.11) |
| Menopausal and perimenopausal disorders | N95 | 1530 | 1.47 (1.36-1.58) | 346 | 1.61 (1.43-1.81) |
| **Unnatural cause** |  |  |  |  |  |
| Injuries due to external causes | S00 | 12300 | 1.06 (1.03-1.08) | 2588 | 1.08 (1.04-1.13) |
| Complications due to medical treatment | T80 | 9701 | 1.41 (1.37-1.46) | 2065 | 1.46 (1.39-1.53) |
| Falls | W00 | 8457 | 1.13 (1.09-1.16) | 1836 | 1.19 (1.13-1.25) |
| Other external causes of accidental injury | W20 | 4449 | 1.17 (1.13-1.22) | 900 | 1.15 (1.07-1.23) |
| External causes of morbidity related to medical treatment | Y40 | 12752 | 1.38 (1.34-1.41) | 2647 | 1.39 (1.33-1.45) |
| External causes of morbidity related to other conditions | Y95 | 2044 | 1.45 (1.36-1.55) | 435 | 1.50 (1.35-1.66) |

A total of 113 disease conditions were confirmed to be significantly associated with MAFLD after Bonferroni correction. Compared with individuals with non-MAFLD, significantly higher HRs (95%CIs without overlap) for MAFLD with or without TM6SF2 rs58542926 (C>T) were marked in red.

^*^Combined ICD-10 codes derived from the original ICD-10 code and are displayed in the Additional file 1.

^#^Number of the participants diagnosed with the corresponding diseases.

HR, hazard ratio; CI, confidence interval. MAFLD, Metabolic dysfunction-associated fatty liver disease.

Additional file 1: Table S19. PheWAS using Cox regression was conducted to investigate the relationship of MAFLD with or without TM6SF2 rs58542926 (C>T) and causes of death.

| **Causes of death** | **MAFLD without TM6SF2 rs58542926 vs.**  **Non-MAFLD (N = 298,639)** | | **MAFLD with TM6SF2**  **rs58542926 vs.**  **Non-MAFLD (N = 191,270)** | |
| --- | --- | --- | --- | --- |
|  | **No.^*^** | **HR (95% CI)** | **No.^*^** | **HR (95% CI)** |
| Cardiovascular death | 4938 | 1.62 (1.55-1.69) | 1087 | 1.73 (1.62-1.85) |
| Digestive system death | 1118 | 1.73 (1.58-1.90) | 291 | 2.19 (1.91-2.51) |
| Endocrine system death | 1641 | 3.18 (2.90-3.49) | 366 | 3.44 (3.03-3.92) |
| Genitourinary system death | 1002 | 2.06 (1.86-2.28) | 201 | 2.00 (1.71-2.35) |
| Infectious and parasitic death | 858 | 1.34 (1.25-1.44) | 389 | 1.82 (1.73-1.92) |
| Malignant neoplasms death | 6181 | 1.25 (1.21-1.30) | 1307 | 1.28 (1.21-1.36) |
| Respiratory system death | 2932 | 1.18 (1.12-1.24) | 674 | 1.32 (1.21-1.44) |
| Unnatural cause death | 892 | 1.20 (1.10-1.32) | 191 | 1.25 (1.07-1.46) |

After Bonferroni correction, a total of 8 death causes were significant associated with MAFLD. Compared with individuals with non-MAFLD, significantly higher HRs (95%CIs without overlap) for MAFLD with or without TM6SF2 rs58542926 (C>T) were marked in red.

**^*^**Number of the participants died due to the corresponding causes, including primary and secondary causes of death.

HR, hazard ratio; CI, confidence interval. MAFLD, Metabolic dysfunction-associated fatty liver disease.

Additional file 1: Table S20. PheWAS using Cox regression was conducted to investigate the association of MAFLD with or without PNPLA3 rs738409 (C>G) and 490 subsequent disease conditions.

| **Medical conditions** | **Code^*^** | **MAFLD without PNPLA3 rs738409 vs. Non-MAFLD (N = 208,224)** | | **MAFLD without PNPLA3 rs738409 vs. Non-MAFLD (N = 281,685)** | |
| --- | --- | --- | --- | --- | --- |
|  |  | **No.^#^** | **HR (95% CI)** | **No.^#^** | **HR (95% CI)** |
| **Infectious and parasitic** |  |  |  |  |  |
| Infectious gastroenteritis and colitis | A09 | 1935 | 1.34 (1.27-1.41) | 5401 | 1.42 (1.37-1.48) |
| Sepsis | A41 | 1505 | 1.40 (1.31-1.48) | 4413 | 1.56 (1.49-1.63) |
| Mycoses | B49 | 643 | 1.19 (1.09-1.30) | 1884 | 1.32 (1.24-1.41) |
| Bacterial infectious agents | B96 | 2079 | 1.37 (1.30-1.44) | 6109 | 1.53 (1.48-1.59) |
| **Malignant neoplasms** |  |  |  |  |  |
| Colon cancer | C18 | 491 | 1.17 (1.06-1.30) | 1429 | 1.30 (1.21-1.40) |
| Metastatic cancer | C77 | 1939 | 1.18 (1.12-1.24) | 5281 | 1.23 (1.18-1.27) |
| **Benign neoplasms** |  |  |  |  |  |
| Benign tumors | D10 | 4817 | 1.25 (1.21-1.30) | 13049 | 1.30 (1.27-1.33) |
| **Blood system** |  |  |  |  |  |
| Iron deficiency anaemia | D50 | 1738 | 1.27 (1.20-1.34) | 5079 | 1.42 (1.36-1.47) |
| Other anaemias | D51 | 2488 | 1.23 (1.18-1.29) | 7106 | 1.34 (1.30-1.39) |
| **Endocrine system disease** |  |  |  |  |  |
| Hypothyroid conditions | E00 | 2075 | 1.43 (1.36-1.50) | 5663 | 1.48 (1.43-1.54) |
| Diabetes | E10 | 4918 | 3.82 (3.67-3.97) | 14691 | 4.40 (4.26-4.54) |
| Malnutrition | E40 | 845 | 1.31 (1.21-1.41) | 2477 | 1.46 (1.38-1.55) |
| Obesity | E66 | 5402 | 7.78 (7.43-8.14) | 16110 | 8.92 (8.57-9.29) |
| Disorders of lipoprotein metabolism and other lipidaemias | E78 | 7134 | 1.71 (1.67-1.76) | 19531 | 1.80 (1.76-1.84) |
| Disorders of mineral metabolism | E83 | 735 | 1.31 (1.20-1.42) | 2249 | 1.53 (1.44-1.62) |
| Volume depletion | E86 | 1030 | 1.38 (1.28-1.48) | 2844 | 1.45 (1.37-1.53) |
| Other disorders of fluid, electrolyte and acid-base balance | E87 | 2038 | 1.29 (1.22-1.35) | 6169 | 1.49 (1.43-1.54) |
| **Mental disorder** |  |  |  |  |  |
| Delirium due to known physiological condition | F05 | 637 | 1.26 (1.15-1.38) | 1898 | 1.43 (1.34-1.53) |
| Alcohol abuse | F10 | 773 | 1.33 (1.22-1.44) | 2494 | 1.63 (1.54-1.73) |
| Tobacco abuse | F17 | 2453 | 1.21 (1.15-1.26) | 6678 | 1.25 (1.21-1.29) |
| Depression | F32 | 2568 | 1.56 (1.49-1.64) | 7149 | 1.66 (1.60-1.72) |
| Anxiety | F40 | 1932 | 1.31 (1.24-1.38) | 5392 | 1.39 (1.34-1.45) |
| **Neural system disease** |  |  |  |  |  |
| Epilepsia | G40 | 459 | 1.25 (1.12-1.39) | 1226 | 1.27 (1.17-1.37) |
| Sleep disorder | G47 | 1248 | 3.27 (3.03-3.53) | 3872 | 3.88 (3.65-4.12) |
| Diseases in nerves, nerve roots and nerve plexa | G50 | 2055 | 1.52 (1.44-1.60) | 5740 | 1.62 (1.56-1.68) |
| Polyneuropathies | G60 | 552 | 1.74 (1.57-1.92) | 1705 | 2.04 (1.90-2.20) |
| Cerebral palsy and other paralytic syndromes | G80 | 499 | 1.45 (1.31-1.61) | 1265 | 1.40 (1.30-1.52) |
| **Eye and adnexa disease** |  |  |  |  |  |
| Disorder of eyelid | H02 | 674 | 1.25 (1.15-1.36) | 1803 | 1.27 (1.19-1.36) |
| Disorders of the lens | H25 | 4551 | 1.10 (1.07-1.14) | 12259 | 1.13 (1.10-1.16) |
| Disorders of choroid and retina | H30 | 1543 | 1.24 (1.17-1.31) | 4286 | 1.31 (1.26-1.36) |
| Visual disturbances and blindness | H53 | 828 | 1.26 (1.16-1.36) | 2200 | 1.27 (1.20-1.35) |
| **Ear dieases** |  |  |  |  |  |
| Other disorders of the ear | H90 | 1220 | 1.12 (1.05-1.19) | 3449 | 1.20 (1.15-1.26) |
| **Cardiovascular disease** |  |  |  |  |  |
| Chronic rheumatic heart disease | I05 | 819 | 1.20 (1.11-1.30) | 2437 | 1.36 (1.29-1.44) |
| Primary hypertension | I10 | 13061 | 1.86 (1.82-1.89) | 35659 | 1.96 (1.93-1.99) |
| Angina pectoris | I20 | 2460 | 1.63 (1.56-1.71) | 6747 | 1.71 (1.65-1.77) |
| Acute myocardial infarction | I21 | 1334 | 1.49 (1.40-1.58) | 3707 | 1.57 (1.50-1.65) |
| Other ischemic heart disease | I22 | 452 | 1.47 (1.31-1.64) | 1308 | 1.62 (1.49-1.75) |
| Chronic ischaemic heart disease | I25 | 4068 | 1.54 (1.48-1.60) | 11109 | 1.60 (1.56-1.65) |
| Embolism and thrombosis | I26 | 1453 | 1.41 (1.32-1.49) | 4108 | 1.51 (1.45-1.58) |
| Non-rheumatic valve disorders | I34 | 1259 | 1.25 (1.18-1.34) | 3599 | 1.37 (1.30-1.43) |
| Atrial fibrillation and flutter | I48 | 3380 | 1.40 (1.35-1.46) | 9282 | 1.47 (1.43-1.52) |
| Other cardiac arrhythmias | I49 | 2413 | 1.30 (1.24-1.36) | 6653 | 1.36 (1.32-1.41) |
| Heart failure | I50 | 1754 | 1.75 (1.65-1.85) | 5174 | 1.97 (1.89-2.06) |
| Complications and ill-defined descriptions of heart disease | I51 | 1699 | 1.59 (1.50-1.68) | 4867 | 1.74 (1.67-1.81) |
| Stroke | I60 | 1187 | 1.31 (1.22-1.39) | 3091 | 1.29 (1.23-1.36) |
| Other cerebrovascular diseases | I65 | 1089 | 1.35 (1.26-1.44) | 2923 | 1.38 (1.31-1.45) |
| Aneurysm and dissection | I71 | 502 | 1.31 (1.19-1.45) | 1354 | 1.35 (1.25-1.45) |
| Peripheral vascular disease | I73 | 709 | 1.16 (1.07-1.26) | 2007 | 1.25 (1.18-1.33) |
| Varicose veins of lower extremities | I83 | 646 | 1.13 (1.03-1.23) | 1819 | 1.21 (1.14-1.29) |
| Hypotension | I95 | 1533 | 1.17 (1.11-1.24) | 4385 | 1.28 (1.23-1.33) |
| **Respiratory system disease** |  |  |  |  |  |
| Pneumonia | J12 | 2791 | 1.39 (1.33-1.45) | 7834 | 1.49 (1.44-1.53) |
| Other acute Lower respiratory infections | J20 | 1593 | 1.58 (1.49-1.67) | 4330 | 1.64 (1.57-1.71) |
| Chronic obstructive pulmonary disease | J44 | 2017 | 1.38 (1.31-1.45) | 5581 | 1.45 (1.40-1.51) |
| Asthma | J45 | 3183 | 1.42 (1.37-1.48) | 8659 | 1.47 (1.43-1.52) |
| Pleural effusion | J90 | 1421 | 1.22 (1.15-1.30) | 4021 | 1.32 (1.26-1.38) |
| Other diseases of the respiratory system | J95 | 1266 | 1.31 (1.23-1.40) | 3400 | 1.34 (1.28-1.41) |
| Respiratory failure | J96 | 788 | 1.39 (1.28-1.51) | 2546 | 1.71 (1.61-1.81) |
| **Digestive system disease** |  |  |  |  |  |
| Disease of hard tissue of teeth | K00 | 464 | 1.22 (1.10-1.35) | 1334 | 1.33 (1.24-1.44) |
| Oesophagitis | K20 | 987 | 1.16 (1.08-1.25) | 2863 | 1.29 (1.22-1.35) |
| Gastro-oesophageal reflux disease | K21 | 4303 | 1.39 (1.34-1.44) | 11286 | 1.39 (1.35-1.43) |
| Other diseases of oesophagus | K22 | 1492 | 1.21 (1.14-1.28) | 4044 | 1.25 (1.20-1.30) |
| Gastric ulcer | K25 | 636 | 1.37 (1.25-1.50) | 1751 | 1.44 (1.34-1.53) |
| Gastritis and duodenitis | K29 | 3605 | 1.20 (1.15-1.24) | 10081 | 1.28 (1.25-1.32) |
| Disease of stomach and duodenum | K31 | 1396 | 1.22 (1.15-1.30) | 4285 | 1.43 (1.37-1.49) |
| Umbilical hernia | K42 | 673 | 2.53 (2.29-2.79) | 1983 | 2.84 (2.63-3.06) |
| Ventral hernia | K43 | 514 | 1.86 (1.68-2.07) | 1480 | 2.04 (1.89-2.21) |
| Diaphragmatic hernia | K44 | 3733 | 1.32 (1.27-1.37) | 9674 | 1.30 (1.27-1.34) |
| Noninfective gastroenteritis and colitis | K52 | 1321 | 1.19 (1.12-1.27) | 3829 | 1.32 (1.26-1.38) |
| Diverticular disease of intestine | K57 | 5375 | 1.36 (1.32-1.41) | 14642 | 1.42 (1.39-1.45) |
| Irritable bowel syndrome | K58 | 828 | 1.27 (1.17-1.37) | 2182 | 1.27 (1.20-1.35) |
| Other functional intestinal disorders | K59 | 2340 | 1.18 (1.13-1.24) | 6517 | 1.25 (1.21-1.30) |
| Other diseases of anus and rectum | K62 | 2382 | 1.21 (1.15-1.27) | 6339 | 1.23 (1.19-1.27) |
| Other diseases of intestine | K63 | 2986 | 1.32 (1.26-1.37) | 8399 | 1.41 (1.37-1.46) |
| Haemorrhoids and perianal venous thrombosis | K64 | 1737 | 1.14 (1.08-1.20) | 4432 | 1.11 (1.06-1.15) |
| Other disorders of peritoneum | K66 | 745 | 1.52 (1.40-1.66) | 2092 | 1.63 (1.53-1.74) |
| Other diseases of liver | K76 | 1088 | 2.03 (1.89-2.19) | 4079 | 2.91 (2.76-3.08) |
| Cholelithiasis | K80 | 2002 | 2.09 (1.98-2.21) | 5587 | 2.23 (2.14-2.32) |
| Disease of gallbladder and biliary tract | K82 | 718 | 1.68 (1.54-1.84) | 2123 | 1.89 (1.78-2.02) |
| Other disease of digestive system | K92 | 1848 | 1.25 (1.19-1.32) | 5013 | 1.29 (1.24-1.34) |
| **Skin and subcutaneous disease** |  |  |  |  |  |
| Skin and subcutaneous infections | L01 | 1745 | 1.84 (1.74-1.95) | 4865 | 1.96 (1.88-2.05) |
| Dermatitis | L20 | 726 | 1.35 (1.24-1.47) | 1990 | 1.41 (1.33-1.50) |
| Papulosquamous disorders | L40 | 564 | 1.53 (1.39-1.69) | 1515 | 1.56 (1.45-1.68) |
| Pressure ulcer | L89 | 454 | 1.35 (1.21-1.51) | 1414 | 1.61 (1.49-1.73) |
| Disorder of the skin and subcutaneous tissue | L98 | 741 | 1.17 (1.08-1.27) | 1910 | 1.15 (1.08-1.22) |
| **Musculoskeletal and connective tissue disease** |  |  |  |  |  |
| Autoimmune arthritis | M05 | 777 | 1.42 (1.31-1.54) | 2260 | 1.57 (1.48-1.67) |
| Other inflammatory arthritis | M10 | 4099 | 1.83 (1.76-1.90) | 11480 | 1.96 (1.91-2.02) |
| Osteoarthritis | M15 | 7594 | 1.58 (1.54-1.63) | 20801 | 1.67 (1.63-1.70) |
| Other joint disorders | M20 | 3681 | 1.34 (1.29-1.39) | 9978 | 1.39 (1.35-1.43) |
| Systemic connective tissue disorders | M30 | 585 | 1.22 (1.11-1.34) | 1606 | 1.27 (1.19-1.36) |
| Deforming dorsopathies | M40 | 641 | 1.32 (1.21-1.45) | 1664 | 1.31 (1.22-1.40) |
| Spondylosis | M47 | 1684 | 1.49 (1.41-1.58) | 4627 | 1.56 (1.49-1.63) |
| Spondylopathy | M48 | 1088 | 1.61 (1.50-1.73) | 3003 | 1.70 (1.61-1.79) |
| Other intervertebral disc disorders | M51 | 1227 | 1.56 (1.46-1.67) | 3275 | 1.59 (1.51-1.67) |
| Dorsalgia | M54 | 2334 | 1.49 (1.42-1.56) | 6426 | 1.57 (1.51-1.62) |
| Disorders of synovium and tendons | M65 | 969 | 1.30 (1.20-1.39) | 2670 | 1.36 (1.29-1.43) |
| Shoulder lesion | M75 | 1085 | 1.34 (1.25-1.43) | 3018 | 1.42 (1.35-1.49) |
| Other soft tissue disorders, not elsewhere classified | M79 | 2000 | 1.61 (1.53-1.70) | 5864 | 1.81 (1.74-1.88) |
| **Genitourinary system disease** |  |  |  |  |  |
| Obstructive and reflux uropathy | N13 | 549 | 1.20 (1.09-1.32) | 1621 | 1.35 (1.26-1.44) |
| Acute renal failure | N17 | 2479 | 1.73 (1.65-1.82) | 7355 | 1.96 (1.89-2.03) |
| Chronic kidney disease | N18 | 2407 | 1.93 (1.84-2.03) | 6653 | 2.03 (1.96-2.11) |
| Urolithiasis | N20 | 784 | 1.49 (1.37-1.62) | 2140 | 1.55 (1.46-1.65) |
| Other disorders of kidney and ureter | N29 | 733 | 1.47 (1.35-1.60) | 2077 | 1.59 (1.49-1.69) |
| Bladder disorder | N32 | 1242 | 1.18 (1.10-1.25) | 3243 | 1.17 (1.11-1.22) |
| Disorders of urinary system, possibly infection | N39 | 2581 | 1.41 (1.35-1.48) | 7299 | 1.52 (1.47-1.57) |
| Female genital prolapse | N81 | 768 | 1.33 (1.22-1.44) | 1779 | 1.17 (1.10-1.24) |
| Polyp of female genital tract | N84 | 499 | 1.66 (1.49-1.84) | 1417 | 1.79 (1.65-1.94) |
| Menopausal and perimenopausal disorders | N95 | 500 | 1.44 (1.30-1.60) | 1376 | 1.51 (1.40-1.63) |
| **Unnatural cause** |  |  |  |  |  |
| Injuries due to external causes | S00 | 3911 | 1.01 (0.97-1.05) | 10977 | 1.08 (1.06-1.11) |
| Complications due to medical treatment | T80 | 3136 | 1.37 (1.32-1.43) | 8630 | 1.44 (1.40-1.49) |
| Falls | W00 | 2738 | 1.10 (1.05-1.14) | 7555 | 1.15 (1.12-1.19) |
| Other external causes of accidental injury | W20 | 1409 | 1.12 (1.05-1.18) | 3940 | 1.19 (1.14-1.24) |
| External causes of morbidity related to medical treatment | Y40 | 4120 | 1.33 (1.29-1.38) | 11279 | 1.40 (1.36-1.43) |
| External causes of morbidity related to other conditions | Y95 | 632 | 1.35 (1.23-1.48) | 1847 | 1.50 (1.41-1.60) |

A total of 113 disease conditions were confirmed to be significantly associated with MAFLD after Bonferroni correction. Compared with individuals with non-MAFLD, significantly higher HRs (95%CIs without overlap) for MAFLD with or without PNPLA3 rs738409 (C>G) were marked in red.

^*^Combined ICD-10 codes derived from the original ICD-10 code and are displayed in the Additional file 1.

^#^Number of the participants diagnosed with the corresponding diseases.

HR, hazard ratio; CI, confidence interval. MAFLD, Metabolic dysfunction-associated fatty liver disease.

Additional file 1: Table S21. PheWAS using Cox regression was conducted to investigate the relationship of MAFLD with or without PNPLA3 rs738409 (C>G) and causes of death.

| **Causes of death** | **MAFLD without PNPLA3 rs738409 vs. Non-MAFLD (N = 266,772)** | | **MAFLD with PNPLA3 rs738409 vs. Non-MAFLD (N = 275,966)** | |
| --- | --- | --- | --- | --- |
|  | **No.^*^** | **HR (95% CI)** | **No.^*^** | **HR (95% CI)** |
| Cardiovascular death | 1510 | 1.49 (1.41-1.58) | 4515 | 1.70 (1.63-1.77) |
| Digestive system death | 264 | 1.23 (1.07-1.42) | 1145 | 2.03 (1.86-2.23) |
| Endocrine system death | 489 | 2.85 (2.53-3.21) | 1518 | 3.37 (3.07-3.70) |
| Genitourinary system death | 289 | 1.78 (1.55-2.05) | 914 | 2.15 (1.94-2.38) |
| Infectious and parasitic death | 316 | 1.61 (1.55-1.68) | 752 | 2.04 (1.85-2.25) |
| Malignant neoplasms death | 2019 | 1.23 (1.17-1.29) | 5469 | 1.27 (1.22-1.31) |
| Respiratory system death | 967 | 1.17 (1.09-1.26) | 2639 | 1.22 (1.16-1.28) |
| Unnatural cause death | 288 | 1.17 (1.02-1.33) | 795 | 1.23 (1.12-1.35) |

After Bonferroni correction, a total of 8 death causes were significant associated with MAFLD. Compared with individuals with non-MAFLD, significantly higher HRs (95%CIs without overlap) for MAFLD with or without PNPLA3 rs738409 (C>G) were marked in red.

**^*^**Number of the participants died due to the corresponding causes, including primary and secondary causes of death.

HR, hazard ratio; CI, confidence interval. MAFLD, Metabolic dysfunction-associated fatty liver disease.

Additional file 1: Table S22. Basic characteristics of the participants with and without genetic susceptibility to MAFLD after propensity score matching.

| **Characteristics** | **Total**  **(N=206,060)** | **Without genetic susceptibility to MAFLD**  **(N=103,030)** | **With genetic susceptibility to MAFLD (N=103,030)** |
| --- | --- | --- | --- |
|  |  |  |  |
| Male, n (%) | 86213 (41.8) | 43089 (41.8) | 43124 (41.9) |
| Age (years) | 59 (51-64) | 59 (51-64) | 58 (51-63) |
| Deprivation Index | -2.02 (-3.58-0.77) | -2.04 (-3.60-0.72) | -1.99 (-3.57-0.78) |
| Alcohol intake (g/day) | 8.57 (0-20.00) | 8.57 (0.66-18.57) | 8.57 (0-20.00) |
| BMI (kg/m2) | 27.09 (23.89-30.76) | 24.04 (22.16-26.32) | 30.17 (27.69-33.44) |
| Follow-up time (years) | 12.93 (12.2-13.65) | 12.92 (12.21-13.64) | 12.94 (12.19-13.65) |
| MAFLD, n (%) | 84859 (41.2) | 12038 (11.7) | 72821 (70.7) |

Data were expressed as n (%) and median (25^th^ - 75^th^). A propensity score matching was conducted according to age, sex, and Townsend deprivation index (assigned by the postcode of participants location, which reflect the level of social deprivation in which the participant lives) for subjects with and without MAFLD. The recording of alcohol intake is based on recollection and estimation of the average total amount of red wine, spirits, beer, and fruit wine consumed each week or month. BMI, body mass index; MAFLD, Metabolic dysfunction-associated fatty liver disease.

Additional file 1: Table S23. Basic characteristics of the dead and surviving participants with genetic susceptibility to MAFLD after propensity score matching.

| **Characteristics** | **Total**  **(N=47,405)** | **Survival**  **(N=39,166)** | **Dead**  **(N=8,239)** |
| --- | --- | --- | --- |
|  |  |  |  |
| Male, n (%) | 47405 (47.4) | 18525 (47.3) | 3929 (47.7) |
| Age (years) | 63 (58-66) | 62 (58-66) | 63 (58-66) |
| Deprivation Index | -1.59 (-3.32-1.45) | -1.61 (-3.33-1.42) | -1.54 (-3.30-1.51) |
| Alcohol intake (g/day) | 8.57 (0-21.43) | 8.57 (0-21.43) | 7.14 (0-22.86) |
| BMI (kg/m2) | 29.99 (27.53-33.22) | 29.93 (27.52-33.05) | 30.32 (27.58-34.11) |
| Follow-up time (years) | 12.73 (11.87-13.54) | 13.01 (12.32-13.69) | 8.19 (5.2-10.5) |

Data were expressed as n (%) and median (25^th^ - 75^th^). A propensity score matching was conducted according to age, sex, and Townsend deprivation index (assigned by the postcode of participants location, which reflect the level of social deprivation in which the participant lives) for subjects with and without MAFLD. The recording of alcohol intake is based on recollection and estimation of the average total amount of red wine, spirits, beer, and fruit wine consumed each week or month. BMI, body mass index; MAFLD, Metabolic dysfunction-associated fatty liver disease.

Additional file 1: Table S24. PheWAS using Cox regression was conducted to investigate the relationship between genetic susceptibility to MAFLD and 490 subsequent disease conditions in males and females.

| **Medical conditions** | **Code^*^** | **All (N = 103,030)** | | **Female (N = 59,906)** | | **Male (N = 43,124)** | |
| --- | --- | --- | --- | --- | --- | --- | --- |
|  |  | **No.^#^** | **HR (95% CI)** | **No.^#^** | **HR (95% CI)** | **No.^#^** | **HR (95% CI)** |
| **Infectious and parasitic** |  |  |  |  |  |  |  |
| Infectious gastroenteritis and colitis | A09 | 4664 | 1.40 (1.34-1.47) | 3073 | 1.50 (1.42-1.58) | 1591 | 1.25 (1.16-1.35) |
| Sepsis | A41 | 3510 | 1.65 (1.57-1.75) | 1921 | 1.85 (1.72-2.00) | 1589 | 1.46 (1.36-1.58) |
| Mycoses | B49 | 1570 | 1.33 (1.24-1.44) | 964 | 1.40 (1.27-1.54) | 606 | 1.24 (1.10-1.39) |
| Bacterial infectious agents | B96 | 4986 | 1.52 (1.46-1.59) | 3071 | 1.75 (1.65-1.85) | 1915 | 1.26 (1.18-1.34) |
| **Malignant neoplasms** |  |  |  |  |  |  |  |
| Colon cancer | C18 | 1099 | 1.23 (1.12-1.34) | 572 | 1.13 (1.00-1.27) | 527 | 1.36 (1.19-1.55) |
| Breast cancer | C50 | 2501 | 1.16 (1.09-1.22) | 2488 | 1.16 (1.10-1.23) | 13 | 0.77 (0.37-1.58) |
| Metastatic cancer | C77 | 4512 | 1.23 (1.17-1.28) | 2697 | 1.22 (1.16-1.30) | 1815 | 1.23 (1.15-1.32) |
| **Benign neoplasms** |  |  |  |  |  |  |  |
| Benign tumors | D10 | 10869 | 1.28 (1.24-1.31) | 6330 | 1.29 (1.24-1.34) | 4539 | 1.26 (1.20-1.31) |
| **Blood system** |  |  |  |  |  |  |  |
| Iron deficiency anaemia | D50 | 4442 | 1.48 (1.41-1.55) | 2949 | 1.72 (1.63-1.83) | 1493 | 1.15 (1.07-1.24) |
| Other anaemias | D51 | 6133 | 1.36 (1.31-1.42) | 3862 | 1.54 (1.46-1.62) | 2271 | 1.14 (1.08-1.22) |
| **Endocrine system disease** |  |  |  |  |  |  |  |
| Hypothyroid conditions | E00 | 5947 | 1.52 (1.46-1.58) | 4991 | 1.59 (1.52-1.66) | 956 | 1.30 (1.18-1.43) |
| Diabetes | E10 | 10544 | 3.62 (3.48-3.77) | 6135 | 5.02 (4.73-5.33) | 4409 | 2.59 (2.46-2.74) |
| Malnutrition | E40 | 2180 | 1.43 (1.34-1.53) | 1527 | 1.60 (1.48-1.73) | 653 | 1.15 (1.02-1.28) |
| Obesity | E66 | 12207 | 5.99 (5.72-6.26) | 8444 | 8.52 (7.99-9.07) | 3763 | 3.56 (3.33-3.81) |
| Disorders of lipoprotein metabolism and other lipidaemias | E78 | 15124 | 1.79 (1.75-1.84) | 8419 | 2.02 (1.94-2.09) | 6705 | 1.57 (1.51-1.63) |
| Disorders of mineral metabolism | E83 | 1874 | 1.60 (1.48-1.72) | 1142 | 1.71 (1.56-1.88) | 732 | 1.44 (1.29-1.61) |
| Volume depletion | E86 | 2323 | 1.44 (1.35-1.53) | 1335 | 1.58 (1.45-1.73) | 988 | 1.28 (1.16-1.40) |
| Other disorders of fluid, electrolyte and acid-base balance | E87 | 5075 | 1.46 (1.40-1.52) | 2986 | 1.53 (1.45-1.62) | 2089 | 1.36 (1.27-1.45) |
| **Mental disorder** |  |  |  |  |  |  |  |
| Delirium due to known physiological condition | F05 | 1487 | 1.44 (1.33-1.56) | 810 | 1.49 (1.34-1.66) | 677 | 1.39 (1.23-1.56) |
| Alcohol abuse | F10 | 1716 | 1.60 (1.48-1.72) | 617 | 1.72 (1.51-1.96) | 1099 | 1.53 (1.39-1.68) |
| Tobacco abuse | F17 | 5372 | 1.27 (1.22-1.32) | 2964 | 1.50 (1.42-1.59) | 2408 | 1.06 (1.00-1.12) |
| Depression | F32 | 6529 | 1.65 (1.59-1.72) | 4736 | 1.85 (1.76-1.94) | 1793 | 1.30 (1.21-1.39) |
| Anxiety | F40 | 5058 | 1.34 (1.29-1.40) | 3740 | 1.40 (1.34-1.47) | 1318 | 1.20 (1.11-1.30) |
| **Neural system disease** |  |  |  |  |  |  |  |
| Sleep disorder | G47 | 2563 | 2.71 (2.52-2.92) | 1276 | 3.66 (3.26-4.12) | 1287 | 2.16 (1.96-2.38) |
| Diseases in nerves, nerve roots and nerve plexa | G50 | 4987 | 1.52 (1.45-1.58) | 3365 | 1.58 (1.50-1.67) | 1622 | 1.40 (1.30-1.51) |
| Polyneuropathies | G60 | 1277 | 1.80 (1.64-1.97) | 670 | 2.03 (1.78-2.32) | 607 | 1.60 (1.41-1.82) |
| Cerebral palsy and other paralytic syndromes | G80 | 1033 | 1.43 (1.30-1.58) | 558 | 1.67 (1.46-1.91) | 475 | 1.22 (1.07-1.40) |
| **Eye and adnexa disease** |  |  |  |  |  |  |  |
| Disorder of eyelid | H02 | 1468 | 1.24 (1.15-1.34) | 855 | 1.18 (1.07-1.30) | 613 | 1.35 (1.20-1.53) |
| Disorders of the lens | H25 | 10994 | 1.13 (1.10-1.16) | 7393 | 1.13 (1.09-1.17) | 3601 | 1.12 (1.07-1.18) |
| Disorders of choroid and retina | H30 | 3539 | 1.29 (1.22-1.35) | 2164 | 1.31 (1.23-1.40) | 1375 | 1.25 (1.16-1.35) |
| Visual disturbances and blindness | H53 | 1872 | 1.28 (1.20-1.37) | 1150 | 1.35 (1.24-1.47) | 722 | 1.19 (1.07-1.32) |
| **Ear dieases** |  |  |  |  |  |  |  |
| Other disorders of the ear | H90 | 2723 | 1.20 (1.14-1.27) | 1520 | 1.29 (1.20-1.39) | 1203 | 1.10 (1.02-1.20) |
| **Cardiovascular disease** |  |  |  |  |  |  |  |
| Chronic rheumatic heart disease | I05 | 1913 | 1.34 (1.25-1.43) | 1037 | 1.47 (1.34-1.62) | 876 | 1.21 (1.10-1.33) |
| Primary hypertension | I10 | 28476 | 1.91 (1.88-1.95) | 16576 | 2.15 (2.10-2.21) | 11900 | 1.64 (1.59-1.68) |
| Angina pectoris | I20 | 4949 | 1.70 (1.62-1.78) | 2422 | 1.95 (1.82-2.08) | 2527 | 1.51 (1.42-1.61) |
| Acute myocardial infarction | I21 | 2577 | 1.57 (1.47-1.67) | 1004 | 1.73 (1.56-1.92) | 1573 | 1.48 (1.37-1.60) |
| Chronic ischaemic heart disease | I25 | 7983 | 1.60 (1.54-1.66) | 3732 | 1.89 (1.79-2.00) | 4251 | 1.41 (1.35-1.48) |
| Embolism and thrombosis | I26 | 3385 | 1.58 (1.49-1.66) | 1961 | 1.91 (1.77-2.05) | 1424 | 1.27 (1.17-1.37) |
| Non-rheumatic valve disorders | I34 | 2757 | 1.32 (1.25-1.40) | 1439 | 1.45 (1.34-1.58) | 1318 | 1.20 (1.11-1.30) |
| Atrial fibrillation and flutter | I48 | 6838 | 1.40 (1.35-1.46) | 3352 | 1.56 (1.48-1.65) | 3486 | 1.28 (1.22-1.35) |
| Other cardiac arrhythmias | I49 | 4936 | 1.30 (1.24-1.35) | 2371 | 1.35 (1.27-1.44) | 2565 | 1.25 (1.18-1.32) |
| Heart failure | I50 | 3692 | 1.79 (1.70-1.89) | 1870 | 2.11 (1.94-2.28) | 1822 | 1.55 (1.44-1.67) |
| Complications and ill-defined descriptions of heart disease | I51 | 3539 | 1.69 (1.60-1.78) | 1737 | 2.02 (1.86-2.19) | 1802 | 1.45 (1.35-1.56) |
| Stroke | I60 | 2366 | 1.26 (1.19-1.34) | 1229 | 1.40 (1.29-1.53) | 1137 | 1.14 (1.04-1.24) |
| Other cerebrovascular diseases | I65 | 2332 | 1.35 (1.27-1.44) | 1270 | 1.45 (1.33-1.58) | 1062 | 1.25 (1.14-1.37) |
| Varicose veins of lower extremities | I83 | 1550 | 1.21 (1.12-1.30) | 984 | 1.22 (1.11-1.34) | 566 | 1.18 (1.04-1.33) |
| Hypotension | I95 | 3516 | 1.24 (1.18-1.31) | 1927 | 1.24 (1.16-1.32) | 1589 | 1.25 (1.16-1.35) |
| **Respiratory system disease** |  |  |  |  |  |  |  |
| Pneumonia | J12 | 6133 | 1.47 (1.41-1.53) | 3369 | 1.63 (1.55-1.72) | 2764 | 1.31 (1.23-1.38) |
| Other acute Lower respiratory infections | J20 | 3443 | 1.56 (1.48-1.64) | 2032 | 1.72 (1.60-1.85) | 1411 | 1.36 (1.26-1.48) |
| Chronic obstructive pulmonary disease | J44 | 4402 | 1.35 (1.29-1.41) | 2531 | 1.49 (1.40-1.58) | 1871 | 1.20 (1.12-1.28) |
| Asthma | J45 | 7699 | 1.50 (1.45-1.55) | 5202 | 1.72 (1.65-1.80) | 2497 | 1.18 (1.12-1.25) |
| Pleural effusion | J90 | 3204 | 1.35 (1.28-1.42) | 1747 | 1.46 (1.36-1.57) | 1457 | 1.24 (1.15-1.34) |
| Other diseases of the respiratory system | J95 | 2807 | 1.41 (1.33-1.49) | 1645 | 1.55 (1.44-1.68) | 1162 | 1.24 (1.14-1.35) |
| Respiratory failure | J96 | 1991 | 1.76 (1.64-1.89) | 1101 | 2.11 (1.91-2.35) | 890 | 1.46 (1.32-1.62) |
| **Digestive system disease** |  |  |  |  |  |  |  |
| Disease of hard tissue of teeth | K00 | 1105 | 1.31 (1.20-1.43) | 673 | 1.45 (1.29-1.63) | 432 | 1.13 (0.99-1.30) |
| Oesophagitis | K20 | 2402 | 1.36 (1.28-1.45) | 1451 | 1.55 (1.43-1.68) | 951 | 1.15 (1.05-1.26) |
| Gastro-oesophageal reflux disease | K21 | 10002 | 1.49 (1.44-1.53) | 6622 | 1.67 (1.61-1.74) | 3380 | 1.22 (1.16-1.29) |
| Other diseases of oesophagus | K22 | 3259 | 1.30 (1.24-1.37) | 1817 | 1.46 (1.36-1.57) | 1442 | 1.14 (1.06-1.23) |
| Gastric ulcer | K25 | 1468 | 1.48 (1.37-1.61) | 915 | 1.59 (1.44-1.77) | 553 | 1.33 (1.17-1.51) |
| Gastritis and duodenitis | K29 | 8756 | 1.31 (1.27-1.36) | 5559 | 1.39 (1.34-1.45) | 3197 | 1.20 (1.14-1.26) |
| Disease of stomach and duodenum | K31 | 3879 | 1.56 (1.49-1.64) | 2574 | 1.63 (1.53-1.73) | 1305 | 1.45 (1.33-1.58) |
| Umbilical hernia | K42 | 1306 | 2.50 (2.26-2.76) | 536 | 4.18 (3.45-5.06) | 770 | 1.95 (1.73-2.20) |
| Ventral hernia | K43 | 1195 | 1.91 (1.73-2.10) | 762 | 2.27 (2.00-2.58) | 433 | 1.49 (1.29-1.73) |
| Diaphragmatic hernia | K44 | 8851 | 1.47 (1.42-1.52) | 6008 | 1.70 (1.64-1.78) | 2843 | 1.14 (1.08-1.20) |
| Noninfective gastroenteritis and colitis | K52 | 3359 | 1.29 (1.23-1.36) | 2237 | 1.37 (1.28-1.45) | 1122 | 1.17 (1.08-1.28) |
| Diverticular disease of intestine | K57 | 12336 | 1.44 (1.40-1.48) | 7648 | 1.51 (1.46-1.57) | 4688 | 1.33 (1.28-1.39) |
| Irritable bowel syndrome | K58 | 2255 | 1.27 (1.19-1.35) | 1794 | 1.31 (1.22-1.41) | 461 | 1.14 (0.99-1.30) |
| Other functional intestinal disorders | K59 | 5526 | 1.18 (1.14-1.23) | 3433 | 1.24 (1.18-1.30) | 2093 | 1.11 (1.04-1.18) |
| Other diseases of anus and rectum | K62 | 5085 | 1.18 (1.14-1.23) | 2774 | 1.21 (1.15-1.28) | 2311 | 1.15 (1.08-1.22) |
| Other diseases of intestine | K63 | 6577 | 1.34 (1.29-1.39) | 3557 | 1.34 (1.28-1.41) | 3020 | 1.34 (1.27-1.41) |
| Other disorders of peritoneum | K66 | 1789 | 1.75 (1.62-1.89) | 1183 | 2.13 (1.93-2.36) | 606 | 1.30 (1.15-1.46) |
| Other diseases of liver | K76 | 3267 | 2.90 (2.71-3.11) | 2063 | 3.33 (3.04-3.64) | 1204 | 2.38 (2.15-2.64) |
| Cholelithiasis | K80 | 5084 | 2.50 (2.38-2.63) | 3747 | 2.94 (2.76-3.13) | 1337 | 1.78 (1.62-1.94) |
| Disease of gallbladder and biliary tract | K82 | 1866 | 2.01 (1.86-2.18) | 1293 | 2.27 (2.06-2.50) | 573 | 1.60 (1.40-1.83) |
| Other disease of digestive system | K92 | 4097 | 1.27 (1.22-1.33) | 2320 | 1.33 (1.25-1.41) | 1777 | 1.21 (1.13-1.30) |
| **Skin and subcutaneous disease** |  |  |  |  |  |  |  |
| Skin and subcutaneous infections | L01 | 3695 | 1.71 (1.63-1.81) | 2124 | 2.03 (1.88-2.18) | 1571 | 1.41 (1.31-1.53) |
| Dermatitis | L20 | 1697 | 1.35 (1.25-1.45) | 1019 | 1.43 (1.30-1.57) | 678 | 1.25 (1.11-1.40) |
| Papulosquamous disorders | L40 | 1253 | 1.46 (1.34-1.60) | 723 | 1.46 (1.30-1.63) | 530 | 1.47 (1.29-1.68) |
| Follicular cyst of the skin and subcutaneous tissue | L72 | 1137 | 1.21 (1.11-1.31) | 659 | 1.21 (1.08-1.35) | 478 | 1.21 (1.06-1.38) |
| Pressure ulcer | L89 | 1096 | 1.52 (1.39-1.67) | 620 | 1.80 (1.57-2.05) | 476 | 1.27 (1.11-1.45) |
| **Musculoskeletal and connective tissue disease** |  |  |  |  |  |  |  |
| Autoimmune arthritis | M05 | 2053 | 1.49 (1.39-1.60) | 1566 | 1.63 (1.50-1.76) | 487 | 1.19 (1.04-1.35) |
| Other inflammatory arthritis | M10 | 9186 | 1.76 (1.71-1.82) | 5742 | 1.83 (1.75-1.91) | 3444 | 1.67 (1.58-1.76) |
| Osteoarthritis | M15 | 18276 | 1.64 (1.60-1.68) | 12506 | 1.75 (1.70-1.80) | 5770 | 1.47 (1.41-1.53) |
| Other joint disorders | M20 | 8853 | 1.39 (1.34-1.43) | 5887 | 1.44 (1.39-1.50) | 2966 | 1.29 (1.22-1.36) |
| Systemic connective tissue disorders | M30 | 1542 | 1.22 (1.14-1.32) | 1200 | 1.27 (1.17-1.38) | 342 | 1.09 (0.94-1.27) |
| Deforming dorsopathies | M40 | 1569 | 1.22 (1.14-1.32) | 1204 | 1.31 (1.20-1.42) | 365 | 1.01 (0.88-1.17) |
| Spondylosis | M47 | 4079 | 1.52 (1.45-1.60) | 2829 | 1.61 (1.52-1.71) | 1250 | 1.36 (1.25-1.48) |
| Spondylopathy | M48 | 2552 | 1.61 (1.51-1.71) | 1649 | 1.72 (1.58-1.86) | 903 | 1.45 (1.31-1.61) |
| Other intervertebral disc disorders | M51 | 2753 | 1.51 (1.42-1.60) | 1806 | 1.61 (1.49-1.73) | 947 | 1.36 (1.23-1.50) |
| Dorsalgia | M54 | 5606 | 1.55 (1.49-1.62) | 3790 | 1.67 (1.59-1.76) | 1816 | 1.35 (1.26-1.45) |
| Disorders of synovium and tendons | M65 | 2298 | 1.34 (1.26-1.43) | 1542 | 1.39 (1.29-1.50) | 756 | 1.26 (1.14-1.41) |
| Shoulder lesion | M75 | 2469 | 1.40 (1.31-1.48) | 1552 | 1.53 (1.42-1.66) | 917 | 1.21 (1.10-1.34) |
| Other soft tissue disorders, not elsewhere classified | M79 | 5060 | 1.73 (1.65-1.81) | 3585 | 2.02 (1.91-2.13) | 1475 | 1.29 (1.20-1.39) |
| **Genitourinary system disease** |  |  |  |  |  |  |  |
| Obstructive and reflux uropathy | N13 | 1264 | 1.39 (1.28-1.51) | 614 | 1.63 (1.44-1.86) | 650 | 1.22 (1.08-1.36) |
| Acute renal failure | N17 | 5442 | 1.88 (1.80-1.97) | 2884 | 2.31 (2.16-2.47) | 2558 | 1.56 (1.46-1.66) |
| Chronic kidney disease | N18 | 5266 | 1.93 (1.85-2.02) | 3213 | 2.22 (2.08-2.36) | 2053 | 1.61 (1.50-1.73) |
| Urolithiasis | N20 | 1512 | 1.48 (1.37-1.60) | 628 | 1.67 (1.47-1.89) | 884 | 1.37 (1.24-1.52) |
| Other disorders of kidney and ureter | N29 | 1598 | 1.60 (1.48-1.73) | 798 | 1.77 (1.58-1.99) | 800 | 1.46 (1.31-1.63) |
| Bladder disorder | N32 | 2459 | 1.22 (1.15-1.30) | 1058 | 1.48 (1.35-1.63) | 1401 | 1.08 (1.00-1.17) |
| Disorders of urinary system, possibly infection | N39 | 6327 | 1.52 (1.46-1.58) | 4220 | 1.63 (1.55-1.71) | 2107 | 1.34 (1.26-1.43) |
| Female genital prolapse | N81 | 2479 | 1.32 (1.25-1.41) | 2479 | 1.34 (1.26-1.42) |  |  |
| Polyp of female genital tract | N84 | 1754 | 1.73 (1.60-1.86) | 1754 | 1.74 (1.61-1.88) |  |  |
| Menopausal and perimenopausal disorders | N95 | 1727 | 1.45 (1.34-1.56) | 1727 | 1.45 (1.35-1.57) |  |  |
| **Unnatural cause** |  |  |  |  |  |  |  |
| Complications due to medical treatment | T80 | 7023 | 1.39 (1.34-1.44) | 4164 | 1.52 (1.45-1.59) | 2859 | 1.23 (1.17-1.30) |
| Falls | W00 | 6828 | 1.09 (1.05-1.12) | 4610 | 1.08 (1.04-1.12) | 2218 | 1.10 (1.04-1.17) |
| Other external causes of accidental injury | W20 | 3266 | 1.21 (1.15-1.28) | 1739 | 1.31 (1.22-1.41) | 1527 | 1.12 (1.04-1.20) |
| External causes of morbidity related to medical treatment | Y40 | 9120 | 1.35 (1.31-1.40) | 5333 | 1.47 (1.41-1.53) | 3787 | 1.22 (1.16-1.27) |
| External causes of morbidity related to other conditions | Y95 | 1414 | 1.62 (1.49-1.77) | 756 | 1.97 (1.74-2.23) | 658 | 1.35 (1.20-1.52) |

A total of 108 disease conditions were confirmed that significant associated with genetic susceptibility to MAFLD after Bonferroni correction. Significantly higher HRs (95%CIs without overlap) were marked in red in males or females . HR, hazard ratio; CI, confidence interval. MAFLD, Metabolic dysfunction-associated fatty liver disease.

^*^Combined ICD-10 codes originates from the original ICD-10 code and are displayed in the Additional file 1.

^#^Number of the participants diagnosed with the corresponding diseases.

Additional file 1: Table S25. PheWAS using Cox regression was conducted to investigate the relationship between genetic susceptibility to MAFLD and cause of death in males and/or females.

| **Causes of death** | **Total (N = 103,030)** | | **Female (N = 59,906)** | | **Male (N = 43,124)** | |
| --- | --- | --- | --- | --- | --- | --- |
|  | **No.^*^** | **HR (95% CI)** | **No.^*^** | **HR (95% CI)** | **No.^*^** | **HR (95% CI)** |
| Cardiovascular death | 3096 | 1.56 (1.48-1.65) | 1363 | 1.80 (1.65-1.97) | 1733 | 1.41 (1.31-1.52) |
| Digestive system death | 886 | 2.43 (2.15-2.74) | 443 | 2.88 (2.40-3.46) | 443 | 2.10 (1.78-2.47) |
| Endocrine system death | 985 | 2.62 (2.32-2.95) | 462 | 3.26 (2.70-3.93) | 523 | 2.23 (1.91-2.59) |
| Genitourinary system death | 674 | 1.99 (1.75-2.27) | 345 | 2.70 (2.20-3.30) | 329 | 1.56 (1.31-1.85) |
| Malignant neoplasms death | 4404 | 1.25 (1.19-1.30) | 2417 | 1.31 (1.24-1.40) | 1987 | 1.17 (1.10-1.25) |
| Respiratory system death | 1986 | 1.20 (1.13-1.28) | 980 | 1.49 (1.35-1.65) | 1006 | 1.01 (0.93-1.10) |

After Bonferroni correction, a total of 7 death causes were significant associated with high genetic susceptibility to MAFLD. Significantly higher HRs (95%CIs without overlap) were marked in red in normal weight (body mass index < 25 kg/m^2^) or over weight/obesity (body mass index ≥ 25 kg/m^2^) individuals.

**^*^**Number of the participants died due to the corresponding causes, including primary and secondary causes of death.

HR, hazard ratio; CI, confidence interval. MAFLD, Metabolic dysfunction-associated fatty liver disease.

Additional file 1: Table S26. Temporal disease pairs with a significantly increased risk of disease 2 (D2) after disease 1 (D1) in individuals with high genetic susceptibility to MAFLD.

| **D1→D2 code^*^** | **D1 description** | **D2 description** | **No.^#^** | **OR (95% CI)** | **Percentage^&^** |
| --- | --- | --- | --- | --- | --- |
| C77→A41 | Metastatic cancer | Sepsis | 646 | 3.89 (3.28-4.62) | 10.60% |
| C77→D51 | Metastatic cancer | Other anaemias | 568 | 4.21 (3.49-5.08) | 11.68% |
| C77→E87 | Metastatic cancer | Other disorders of fluid, electrolyte and acid-base balance | 566 | 4.26 (3.52-5.14) | 15.99% |
| C77→J12 | Metastatic cancer | Pneumonia | 630 | 4.20 (3.51-5.02) | 13.29% |
| C77→K59 | Metastatic cancer | Other functional intestinal disorders | 519 | 3.79 (3.14-4.57) | 14.51% |
| C77→N17 | Metastatic cancer | Acute renal failure | 571 | 4.80 (3.94-5.85) | 14.16% |
| C77→Y40 | Metastatic cancer | External causes of morbidity related to medical treatment | 835 | 5.22 (4.41-6.18) | 11.20% |
| D10→A09 | Benign tumors | Infectious gastroenteritis and colitis | 517 | 1.87 (1.62-2.17) | 13.26% |
| D10→C77 | Benign tumors | Metastatic cancer | 554 | 1.75 (1.53-2.01) | 6.79% |
| D10→D51 | Benign tumors | Other anaemias | 641 | 1.78 (1.56-2.02) | 13.55% |
| D10→E66 | Benign tumors | Obesity | 956 | 2.12 (1.89-2.37) | 17.62% |
| D10→E87 | Benign tumors | Other disorders of fluid, electrolyte and acid-base balance | 637 | 1.81 (1.59-2.06) | 6.00% |
| D10→H25 | Benign tumors | Disorders of the lens | 765 | 1.99 (1.76-2.25) | 2.85% |
| D10→I48 | Benign tumors | Atrial fibrillation and flutter | 572 | 1.79 (1.56-2.06) | 8.45% |
| D10→J12 | Benign tumors | Pneumonia | 680 | 1.77 (1.56-2.00) | 5.19% |
| D10→K59 | Benign tumors | Other functional intestinal disorders | 550 | 1.80 (1.56-2.07) | 13.05% |
| D10→M10 | Benign tumors | Other inflammatory arthritis | 772 | 2.10 (1.85-2.38) | 12.56% |
| D10→N17 | Benign tumors | Acute renal failure | 698 | 1.83 (1.62-2.08) | 6.06% |
| D10→N18 | Benign tumors | Chronic kidney disease | 555 | 2.02 (1.75-2.33) | 12.90% |
| D10→N39 | Benign tumors | Disorders of urinary system, possibly infection | 586 | 1.68 (1.47-1.92) | 7.19% |
| D10→T80 | Benign tumors | Complications due to medical treatment | 716 | 1.86 (1.64-2.10) | 8.37% |
| D10→Y40 | Benign tumors | External causes of morbidity related to medical treatment | 942 | 2.01 (1.80-2.24) | 8.45% |
| D51→A41 | Other anaemias | Sepsis | 570 | 3.52 (2.96-4.19) | 14.76% |
| D51→E87 | Other anaemias | Other disorders of fluid, electrolyte and acid-base balance | 760 | 3.55 (3.05-4.13) | 18.85% |
| D51→J12 | Other anaemias | Pneumonia | 716 | 3.69 (3.15-4.33) | 16.01% |
| D51→K59 | Other anaemias | Other functional intestinal disorders | 543 | 3.31 (2.78-3.94) | 12.87% |
| D51→N17 | Other anaemias | Acute renal failure | 785 | 3.79 (3.25-4.42) | 17.33% |
| E00→E66 | Hypothyroid conditions | Obesity | 672 | 3.33 (2.84-3.89) | 31.48% |
| E00→E78 | Hypothyroid conditions | Disorders of lipoprotein metabolism and other lipidaemias | 566 | 3.56 (2.99-4.24) | 41.23% |
| E00→H25 | Hypothyroid conditions | Disorders of the lens | 546 | 2.92 (2.47-3.45) | 21.40% |
| E00→K21 | Hypothyroid conditions | Gastro-oesophageal reflux disease | 516 | 3.19 (2.67-3.80) | 22.28% |
| E00→K57 | Hypothyroid conditions | Diverticular disease of intestine | 573 | 2.92 (2.49-3.44) | 13.48% |
| E00→M15 | Hypothyroid conditions | Osteoarthritis | 724 | 3.98 (3.38-4.68) | 36.98% |
| E00→Y40 | Hypothyroid conditions | External causes of morbidity related to medical treatment | 563 | 3.22 (2.72-3.81) | 14.10% |
| E10→A09 | Diabetes | Infectious gastroenteritis and colitis | 683 | 1.67 (1.48-1.89) | 14.91% |
| E10→A41 | Diabetes | Sepsis | 589 | 1.77 (1.55-2.02) | 12.09% |
| E10→B96 | Diabetes | Bacterial infectious agents | 671 | 1.87 (1.65-2.13) | 11.39% |
| E10→D10 | Diabetes | Benign tumors | 778 | 1.96 (1.74-2.21) | 20.46% |
| E10→D50 | Diabetes | Iron deficiency anaemia | 699 | 1.70 (1.51-1.92) | 17.54% |
| E10→D51 | Diabetes | Other anaemias | 930 | 1.71 (1.54-1.90) | 14.28% |
| E10→E66 | Diabetes | Obesity | 1236 | 2.31 (2.09-2.56) | 29.66% |
| E10→E78 | Diabetes | Disorders of lipoprotein metabolism and other lipidaemias | 1436 | 2.48 (2.25-2.73) | 36.91% |
| E10→E87 | Diabetes | Other disorders of fluid, electrolyte and acid-base balance | 870 | 1.80 (1.61-2.02) | 15.45% |
| E10→F32 | Diabetes | Depression | 546 | 1.84 (1.60-2.12) | 24.22% |
| E10→H25 | Diabetes | Disorders of the lens | 987 | 1.94 (1.74-2.16) | 27.20% |
| E10→I25 | Diabetes | Chronic ischaemic heart disease | 858 | 1.98 (1.77-2.22) | 27.39% |
| E10→I48 | Diabetes | Atrial fibrillation and flutter | 684 | 1.89 (1.66-2.15) | 24.64% |
| E10→I49 | Diabetes | Other cardiac arrhythmias | 579 | 1.69 (1.48-1.93) | 20.69% |
| E10→I50 | Diabetes | Heart failure | 572 | 1.60 (1.40-1.82) | 17.80% |
| E10→I51 | Diabetes | Complications and ill-defined descriptions of heart disease | 537 | 1.71 (1.49-1.97) | 16.55% |
| E10→J12 | Diabetes | Pneumonia | 880 | 1.72 (1.54-1.92) | 17.03% |
| E10→K21 | Diabetes | Gastro-oesophageal reflux disease | 758 | 1.86 (1.65-2.10) | 19.85% |
| E10→K29 | Diabetes | Gastritis and duodenitis | 828 | 1.80 (1.61-2.02) | 14.87% |
| E10→K44 | Diabetes | Diaphragmatic hernia | 644 | 2.01 (1.75-2.29) | 17.24% |
| E10→K57 | Diabetes | Diverticular disease of intestine | 1001 | 2.00 (1.80-2.23) | 20.31% |
| E10→K59 | Diabetes | Other functional intestinal disorders | 653 | 1.81 (1.59-2.06) | 13.77% |
| E10→K63 | Diabetes | Other diseases of intestine | 590 | 1.76 (1.54-2.01) | 14.08% |
| E10→K76 | Diabetes | Other diseases of liver | 529 | 1.78 (1.54-2.05) | 20.64% |
| E10→M10 | Diabetes | Other inflammatory arthritis | 850 | 1.90 (1.70-2.13) | 24.05% |
| E10→M15 | Diabetes | Osteoarthritis | 1178 | 2.50 (2.24-2.78) | 27.67% |
| E10→M20 | Diabetes | Other joint disorders | 551 | 1.94 (1.68-2.24) | 21.22% |
| E10→M54 | Diabetes | Dorsalgia | 548 | 1.79 (1.55-2.05) | 15.05% |
| E10→N17 | Diabetes | Acute renal failure | 1034 | 1.86 (1.68-2.06) | 15.43% |
| E10→N18 | Diabetes | Chronic kidney disease | 840 | 1.88 (1.68-2.11) | 22.93% |
| E10→N39 | Diabetes | Disorders of urinary system, possibly infection | 764 | 1.87 (1.66-2.11) | 14.88% |
| E10→T80 | Diabetes | Complications due to medical treatment | 778 | 1.83 (1.62-2.06) | 8.24% |
| E10→W00 | Diabetes | Falls | 653 | 1.77 (1.56-2.02) | 21.99% |
| E10→Y40 | Diabetes | External causes of morbidity related to medical treatment | 998 | 1.84 (1.66-2.04) | 13.49% |
| E66→A09 | Obesity | Infectious gastroenteritis and colitis | 579 | 1.54 (1.35-1.75) | 7.82% |
| E66→A41 | Obesity | Sepsis | 536 | 1.71 (1.49-1.96) | 9.79% |
| E66→B96 | Obesity | Bacterial infectious agents | 650 | 1.65 (1.46-1.87) | 8.90% |
| E66→D50 | Obesity | Iron deficiency anaemia | 584 | 1.67 (1.47-1.91) | 10.17% |
| E66→D51 | Obesity | Other anaemias | 746 | 1.68 (1.50-1.89) | 13.23% |
| E66→E87 | Obesity | Other disorders of fluid, electrolyte and acid-base balance | 737 | 1.69 (1.50-1.90) | 15.21% |
| E66→F40 | Obesity | Anxiety | 585 | 1.65 (1.44-1.88) | 24.48% |
| E66→I50 | Obesity | Heart failure | 583 | 1.75 (1.53-2.00) | 18.13% |
| E66→I51 | Obesity | Complications and ill-defined descriptions of heart disease | 533 | 1.76 (1.53-2.03) | 23.60% |
| E66→J12 | Obesity | Pneumonia | 771 | 1.72 (1.54-1.94) | 15.32% |
| E66→K59 | Obesity | Other functional intestinal disorders | 664 | 1.66 (1.47-1.88) | 11.83% |
| E66→N17 | Obesity | Acute renal failure | 822 | 1.71 (1.53-1.91) | 13.29% |
| E66→N18 | Obesity | Chronic kidney disease | 668 | 1.88 (1.65-2.14) | 23.58% |
| E66→T80 | Obesity | Complications due to medical treatment | 995 | 1.91 (1.71-2.12) | 14.74% |
| E66→Y40 | Obesity | External causes of morbidity related to medical treatment | 1130 | 1.96 (1.77-2.17) | 15.52% |
| E78→E66 | Disorders of lipoprotein metabolism and other lipidaemias | Obesity | 1489 | 1.42 (1.32-1.54) | 30.52% |
| E78→F32 | Disorders of lipoprotein metabolism and other lipidaemias | Depression | 782 | 1.35 (1.21-1.50) | 28.60% |
| E78→H25 | Disorders of lipoprotein metabolism and other lipidaemias | Disorders of the lens | 1371 | 1.29 (1.19-1.40) | 18.86% |
| E78→I20 | Disorders of lipoprotein metabolism and other lipidaemias | Angina pectoris | 931 | 1.31 (1.19-1.45) | 37.15% |
| E78→I25 | Disorders of lipoprotein metabolism and other lipidaemias | Chronic ischaemic heart disease | 1336 | 1.46 (1.34-1.59) | 37.63% |
| E78→K21 | Disorders of lipoprotein metabolism and other lipidaemias | Gastro-oesophageal reflux disease | 1193 | 1.31 (1.20-1.42) | 23.75% |
| E78→K29 | Disorders of lipoprotein metabolism and other lipidaemias | Gastritis and duodenitis | 1091 | 1.29 (1.17-1.41) | 6.07% |
| E78→K57 | Disorders of lipoprotein metabolism and other lipidaemias | Diverticular disease of intestine | 1392 | 1.30 (1.20-1.41) | 10.18% |
| E78→K63 | Disorders of lipoprotein metabolism and other lipidaemias | Other diseases of intestine | 819 | 1.25 (1.13-1.39) | 5.03% |
| E78→M10 | Disorders of lipoprotein metabolism and other lipidaemias | Other inflammatory arthritis | 1276 | 1.29 (1.19-1.41) | 24.23% |
| E78→Y40 | Disorders of lipoprotein metabolism and other lipidaemias | External causes of morbidity related to medical treatment | 1523 | 1.31 (1.21-1.41) | 13.89% |
| F17→E87 | Tobacco abuse | Other disorders of fluid, electrolyte and acid-base balance | 524 | 4.06 (3.35-4.92) | 12.67% |
| F17→J12 | Tobacco abuse | Pneumonia | 548 | 4.03 (3.34-4.86) | 17.54% |
| F17→J44 | Tobacco abuse | Chronic obstructive pulmonary disease | 527 | 3.96 (3.28-4.79) | 29.43% |
| F17→N17 | Tobacco abuse | Acute renal failure | 521 | 4.01 (3.31-4.86) | 12.26% |
| F17→Y40 | Tobacco abuse | External causes of morbidity related to medical treatment | 580 | 3.65 (3.06-4.35) | 11.90% |
| F32→F40 | Depression | Anxiety | 728 | 3.91 (3.33-4.60) | 39.63% |
| F32→J12 | Depression | Pneumonia | 526 | 3.55 (2.96-4.27) | 15.04% |
| G50→E66 | Diseases in nerves, nerve roots and nerve plexa | Obesity | 612 | 4.74 (3.92-5.74) | 16.77% |
| I20→E66 | Angina pectoris | Obesity | 544 | 4.12 (3.41-4.98) | 23.84% |
| I20→Y40 | Angina pectoris | External causes of morbidity related to medical treatment | 615 | 3.75 (3.16-4.45) | 11.96% |
| I21→I25 | Acute myocardial infarction | Chronic ischaemic heart disease | 699 | 49.93 (29.42-84.74) | 52.77% |
| I25→D51 | Chronic ischaemic heart disease | Other anaemias | 676 | 2.11 (1.85-2.41) | 8.85% |
| I25→E66 | Chronic ischaemic heart disease | Obesity | 801 | 2.30 (2.03-2.61) | 25.86% |
| I25→E87 | Chronic ischaemic heart disease | Other disorders of fluid, electrolyte and acid-base balance | 705 | 2.06 (1.81-2.34) | 11.83% |
| I25→H25 | Chronic ischaemic heart disease | Disorders of the lens | 743 | 2.27 (2.00-2.59) | 11.61% |
| I25→I20 | Chronic ischaemic heart disease | Angina pectoris | 1166 | 3.48 (3.08-3.93) | 40.42% |
| I25→I49 | Chronic ischaemic heart disease | Other cardiac arrhythmias | 635 | 2.08 (1.82-2.39) | 28.39% |
| I25→I50 | Chronic ischaemic heart disease | Heart failure | 688 | 2.10 (1.84-2.40) | 24.46% |
| I25→I51 | Chronic ischaemic heart disease | Complications and ill-defined descriptions of heart disease | 666 | 1.93 (1.70-2.20) | 29.14% |
| I25→I95 | Chronic ischaemic heart disease | Hypotension | 591 | 1.81 (1.58-2.07) | 12.99% |
| I25→J12 | Chronic ischaemic heart disease | Pneumonia | 701 | 2.06 (1.81-2.34) | 10.46% |
| I25→K59 | Chronic ischaemic heart disease | Other functional intestinal disorders | 526 | 2.04 (1.76-2.37) | 6.42% |
| I25→N17 | Chronic ischaemic heart disease | Acute renal failure | 791 | 2.24 (1.98-2.54) | 9.65% |
| I25→N18 | Chronic ischaemic heart disease | Chronic kidney disease | 673 | 2.20 (1.92-2.52) | 16.00% |
| I25→N39 | Chronic ischaemic heart disease | Disorders of urinary system, possibly infection | 542 | 2.19 (1.88-2.54) | 8.65% |
| I25→T80 | Chronic ischaemic heart disease | Complications due to medical treatment | 713 | 2.06 (1.81-2.34) | 9.65% |
| I25→W00 | Chronic ischaemic heart disease | Falls | 523 | 2.30 (1.97-2.69) | 10.11% |
| I25→Y40 | Chronic ischaemic heart disease | External causes of morbidity related to medical treatment | 994 | 2.24 (2.01-2.51) | 9.53% |
| I48→E87 | Atrial fibrillation and flutter | Other disorders of fluid, electrolyte and acid-base balance | 713 | 2.38 (2.08-2.72) | 26.01% |
| I48→I49 | Atrial fibrillation and flutter | Other cardiac arrhythmias | 637 | 2.61 (2.25-3.03) | 28.24% |
| I48→I50 | Atrial fibrillation and flutter | Heart failure | 831 | 2.77 (2.43-3.16) | 30.69% |
| I48→I51 | Atrial fibrillation and flutter | Complications and ill-defined descriptions of heart disease | 722 | 2.47 (2.16-2.83) | 26.46% |
| I48→I95 | Atrial fibrillation and flutter | Hypotension | 557 | 2.22 (1.91-2.58) | 22.64% |
| I48→J12 | Atrial fibrillation and flutter | Pneumonia | 739 | 2.42 (2.12-2.77) | 27.93% |
| I48→N17 | Atrial fibrillation and flutter | Acute renal failure | 796 | 2.41 (2.12-2.74) | 23.66% |
| I48→N18 | Atrial fibrillation and flutter | Chronic kidney disease | 617 | 2.29 (1.98-2.64) | 19.12% |
| I48→N39 | Atrial fibrillation and flutter | Disorders of urinary system, possibly infection | 538 | 2.32 (1.99-2.70) | 13.82% |
| I48→W00 | Atrial fibrillation and flutter | Falls | 543 | 2.22 (1.91-2.58) | 16.29% |
| I50→E87 | Heart failure | Other disorders of fluid, electrolyte and acid-base balance | 540 | 5.09 (4.14-6.27) | 25.12% |
| I50→N17 | Heart failure | Acute renal failure | 585 | 6.09 (4.91-7.56) | 23.50% |
| J20→J12 | Other acute Lower respiratory infections | Pneumonia | 596 | 6.27 (5.05-7.79) | 4.07% |
| J44→E87 | Chronic obstructive pulmonary disease | Other disorders of fluid, electrolyte and acid-base balance | 521 | 3.43 (2.86-4.11) | 15.40% |
| J44→J12 | Chronic obstructive pulmonary disease | Pneumonia | 625 | 4.92 (4.07-5.96) | 21.45% |
| J44→N17 | Chronic obstructive pulmonary disease | Acute renal failure | 534 | 3.24 (2.72-3.85) | 15.22% |
| J45→D10 | Asthma | Benign tumors | 633 | 3.39 (2.88-3.98) | 21.13% |
| J45→E66 | Asthma | Obesity | 905 | 2.98 (2.61-3.39) | 28.99% |
| J45→E78 | Asthma | Disorders of lipoprotein metabolism and other lipidaemias | 784 | 3.50 (3.02-4.06) | 29.38% |
| J45→F32 | Asthma | Depression | 532 | 3.11 (2.62-3.70) | 27.27% |
| J45→H25 | Asthma | Disorders of the lens | 709 | 3.02 (2.60-3.50) | 22.15% |
| J45→J12 | Asthma | Pneumonia | 567 | 2.83 (2.41-3.33) | 16.16% |
| J45→J44 | Asthma | Chronic obstructive pulmonary disease | 616 | 3.38 (2.87-3.99) | 5.82% |
| J45→K21 | Asthma | Gastro-oesophageal reflux disease | 747 | 3.17 (2.73-3.66) | 24.33% |
| J45→K29 | Asthma | Gastritis and duodenitis | 538 | 3.28 (2.75-3.91) | 15.38% |
| J45→K44 | Asthma | Diaphragmatic hernia | 573 | 3.24 (2.74-3.83) | 20.03% |
| J45→K57 | Asthma | Diverticular disease of intestine | 794 | 3.21 (2.79-3.71) | 17.82% |
| J45→M10 | Asthma | Other inflammatory arthritis | 729 | 2.85 (2.47-3.28) | 25.17% |
| J45→M15 | Asthma | Osteoarthritis | 1024 | 3.36 (2.95-3.82) | 31.78% |
| J45→T80 | Asthma | Complications due to medical treatment | 525 | 2.81 (2.38-3.32) | 12.44% |
| J45→Y40 | Asthma | External causes of morbidity related to medical treatment | 732 | 2.96 (2.57-3.42) | 11.59% |
| K21→A09 | Gastro-oesophageal reflux disease | Infectious gastroenteritis and colitis | 556 | 1.99 (1.73-2.30) | 7.48% |
| K21→D50 | Gastro-oesophageal reflux disease | Iron deficiency anaemia | 521 | 2.25 (1.92-2.62) | 16.14% |
| K21→D51 | Gastro-oesophageal reflux disease | Other anaemias | 631 | 2.25 (1.95-2.58) | 15.66% |
| K21→E87 | Gastro-oesophageal reflux disease | Other disorders of fluid, electrolyte and acid-base balance | 603 | 2.28 (1.98-2.64) | 10.31% |
| K21→F40 | Gastro-oesophageal reflux disease | Anxiety | 550 | 2.24 (1.92-2.60) | 22.31% |
| K21→H25 | Gastro-oesophageal reflux disease | Disorders of the lens | 794 | 2.26 (1.99-2.56) | 11.21% |
| K21→J12 | Gastro-oesophageal reflux disease | Pneumonia | 616 | 2.01 (1.75-2.30) | 9.99% |
| K21→K59 | Gastro-oesophageal reflux disease | Other functional intestinal disorders | 599 | 2.16 (1.88-2.49) | 12.17% |
| K21→N17 | Gastro-oesophageal reflux disease | Acute renal failure | 593 | 2.02 (1.75-2.32) | 10.49% |
| K21→N18 | Gastro-oesophageal reflux disease | Chronic kidney disease | 534 | 2.02 (1.75-2.34) | 16.52% |
| K29→A09 | Gastritis and duodenitis | Infectious gastroenteritis and colitis | 560 | 2.15 (1.86-2.50) | 5.33% |
| K29→D50 | Gastritis and duodenitis | Iron deficiency anaemia | 533 | 2.51 (2.14-2.95) | 37.69% |
| K29→D51 | Gastritis and duodenitis | Other anaemias | 662 | 2.54 (2.20-2.93) | 24.53% |
| K29→E66 | Gastritis and duodenitis | Obesity | 857 | 2.59 (2.28-2.94) | 6.52% |
| K29→E87 | Gastritis and duodenitis | Other disorders of fluid, electrolyte and acid-base balance | 633 | 2.32 (2.01-2.67) | 4.17% |
| K29→H25 | Gastritis and duodenitis | Disorders of the lens | 751 | 2.59 (2.26-2.97) | 0.15% |
| K29→J12 | Gastritis and duodenitis | Pneumonia | 659 | 2.26 (1.97-2.60) | 3.38% |
| K29→K21 | Gastritis and duodenitis | Gastro-oesophageal reflux disease | 965 | 3.29 (2.89-3.75) | 41.43% |
| K29→K59 | Gastritis and duodenitis | Other functional intestinal disorders | 542 | 2.11 (1.82-2.45) | 6.07% |
| K29→N17 | Gastritis and duodenitis | Acute renal failure | 672 | 2.39 (2.08-2.75) | 3.47% |
| K29→N18 | Gastritis and duodenitis | Chronic kidney disease | 600 | 2.37 (2.05-2.75) | 8.38% |
| K29→W00 | Gastritis and duodenitis | Falls | 539 | 2.29 (1.97-2.67) | 1.37% |
| K29→Y40 | Gastritis and duodenitis | External causes of morbidity related to medical treatment | 760 | 2.59 (2.27-2.97) | 5.05% |
| K44→D50 | Diaphragmatic hernia | Iron deficiency anaemia | 542 | 2.47 (2.12-2.90) | 34.67% |
| K44→D51 | Diaphragmatic hernia | Other anaemias | 613 | 2.28 (1.97-2.63) | 24.94% |
| K44→E66 | Diaphragmatic hernia | Obesity | 844 | 2.50 (2.20-2.83) | 13.69% |
| K44→E87 | Diaphragmatic hernia | Other disorders of fluid, electrolyte and acid-base balance | 563 | 2.13 (1.84-2.47) | 5.22% |
| K44→H25 | Diaphragmatic hernia | Disorders of the lens | 738 | 2.28 (2.00-2.60) | 2.84% |
| K44→J12 | Diaphragmatic hernia | Pneumonia | 605 | 2.15 (1.87-2.48) | 5.09% |
| K44→K21 | Diaphragmatic hernia | Gastro-oesophageal reflux disease | 1078 | 3.26 (2.88-3.68) | 47.16% |
| K44→K59 | Diaphragmatic hernia | Other functional intestinal disorders | 524 | 2.16 (1.85-2.51) | 7.82% |
| K44→N17 | Diaphragmatic hernia | Acute renal failure | 582 | 2.09 (1.81-2.41) | 4.75% |
| K44→N18 | Diaphragmatic hernia | Chronic kidney disease | 526 | 2.18 (1.87-2.54) | 11.15% |
| K44→T80 | Diaphragmatic hernia | Complications due to medical treatment | 563 | 2.34 (2.01-2.72) | 3.83% |
| K44→Y40 | Diaphragmatic hernia | External causes of morbidity related to medical treatment | 721 | 2.44 (2.13-2.79) | 5.78% |
| K57→A09 | Diverticular disease of intestine | Infectious gastroenteritis and colitis | 641 | 1.56 (1.38-1.77) | 25.61% |
| K57→B96 | Diverticular disease of intestine | Bacterial infectious agents | 527 | 1.51 (1.32-1.72) | 8.48% |
| K57→D51 | Diverticular disease of intestine | Other anaemias | 703 | 1.63 (1.45-1.84) | 19.25% |
| K57→E87 | Diverticular disease of intestine | Other disorders of fluid, electrolyte and acid-base balance | 716 | 1.54 (1.37-1.73) | 7.16% |
| K57→F40 | Diverticular disease of intestine | Anxiety | 557 | 1.62 (1.42-1.85) | 9.86% |
| K57→H25 | Diverticular disease of intestine | Disorders of the lens | 1062 | 1.78 (1.61-1.97) | 1.79% |
| K57→I49 | Diverticular disease of intestine | Other cardiac arrhythmias | 546 | 1.59 (1.39-1.82) | 5.22% |
| K57→J12 | Diverticular disease of intestine | Pneumonia | 765 | 1.59 (1.42-1.78) | 6.46% |
| K57→K59 | Diverticular disease of intestine | Other functional intestinal disorders | 606 | 1.57 (1.38-1.78) | 26.23% |
| K57→K63 | Diverticular disease of intestine | Other diseases of intestine | 738 | 1.86 (1.65-2.10) | 54.86% |
| K57→N17 | Diverticular disease of intestine | Acute renal failure | 759 | 1.56 (1.39-1.75) | 8.65% |
| K57→N18 | Diverticular disease of intestine | Chronic kidney disease | 677 | 1.49 (1.32-1.68) | 12.01% |
| K57→N39 | Diverticular disease of intestine | Disorders of urinary system, possibly infection | 663 | 1.55 (1.37-1.75) | 4.75% |
| K57→Y40 | Diverticular disease of intestine | External causes of morbidity related to medical treatment | 962 | 1.76 (1.59-1.96) | 4.62% |
| K63→H25 | Other diseases of intestine | Disorders of the lens | 528 | 3.45 (2.88-4.13) | 0.53% |
| K80→Y40 | Cholelithiasis | External causes of morbidity related to medical treatment | 538 | 4.72 (3.86-5.78) | 9.74% |
| M10→A09 | Other inflammatory arthritis | Infectious gastroenteritis and colitis | 534 | 2.10 (1.81-2.44) | 10.23% |
| M10→B96 | Other inflammatory arthritis | Bacterial infectious agents | 529 | 2.12 (1.83-2.47) | 7.79% |
| M10→D51 | Other inflammatory arthritis | Other anaemias | 676 | 2.05 (1.80-2.34) | 12.43% |
| M10→E87 | Other inflammatory arthritis | Other disorders of fluid, electrolyte and acid-base balance | 687 | 2.13 (1.87-2.44) | 9.55% |
| M10→J12 | Other inflammatory arthritis | Pneumonia | 706 | 2.16 (1.89-2.46) | 11.63% |
| M10→K59 | Other inflammatory arthritis | Other functional intestinal disorders | 618 | 2.22 (1.93-2.56) | 11.97% |
| M10→N17 | Other inflammatory arthritis | Acute renal failure | 761 | 2.27 (2.00-2.58) | 9.05% |
| M10→N18 | Other inflammatory arthritis | Chronic kidney disease | 636 | 2.03 (1.77-2.33) | 20.31% |
| M10→W00 | Other inflammatory arthritis | Falls | 604 | 2.01 (1.75-2.30) | 14.62% |
| M15→E66 | Osteoarthritis | Obesity | 2120 | 1.35 (1.26-1.44) | 37.80% |
| M15→M10 | Osteoarthritis | Other inflammatory arthritis | 2021 | 1.21 (1.14-1.30) | 14.77% |
| M15→M20 | Osteoarthritis | Other joint disorders | 1356 | 1.28 (1.18-1.39) | 49.02% |
| M20→D10 | Other joint disorders | Benign tumors | 634 | 2.36 (2.04-2.72) | 3.68% |
| M20→D51 | Other joint disorders | Other anaemias | 523 | 2.43 (2.08-2.85) | 6.62% |
| M20→E66 | Other joint disorders | Obesity | 995 | 2.76 (2.44-3.11) | 30.05% |
| M20→F32 | Other joint disorders | Depression | 546 | 2.24 (1.92-2.60) | 20.58% |
| M20→H25 | Other joint disorders | Disorders of the lens | 755 | 2.59 (2.26-2.96) | 1.98% |
| M20→J12 | Other joint disorders | Pneumonia | 550 | 2.25 (1.94-2.62) | 7.01% |
| M20→K21 | Other joint disorders | Gastro-oesophageal reflux disease | 785 | 2.43 (2.14-2.77) | 12.30% |
| M20→K57 | Other joint disorders | Diverticular disease of intestine | 861 | 2.33 (2.07-2.64) | 2.63% |
| M20→K59 | Other joint disorders | Other functional intestinal disorders | 530 | 2.55 (2.17-2.99) | 11.05% |
| M20→M10 | Other joint disorders | Other inflammatory arthritis | 918 | 2.62 (2.32-2.97) | 17.33% |
| M20→M79 | Other joint disorders | Other soft tissue disorders, not elsewhere classified | 545 | 2.32 (1.99-2.70) | 17.49% |
| M20→N17 | Other joint disorders | Acute renal failure | 518 | 2.33 (1.99-2.73) | 7.49% |
| M20→T80 | Other joint disorders | Complications due to medical treatment | 705 | 2.33 (2.03-2.66) | 5.94% |
| M20→Y40 | Other joint disorders | External causes of morbidity related to medical treatment | 771 | 2.54 (2.22-2.90) | 11.08% |
| M54→M47 | Dorsalgia | Spondylosis | 552 | 4.15 (3.43-5.02) | 18.28% |
| N17→A41 | Acute renal failure | Sepsis | 568 | 3.59 (3.01-4.29) | 42.21% |
| N18→E87 | Chronic kidney disease | Other disorders of fluid, electrolyte and acid-base balance | 653 | 3.04 (2.60-3.54) | 19.63% |
| N18→J12 | Chronic kidney disease | Pneumonia | 604 | 3.28 (2.78-3.87) | 16.86% |
| N39→A41 | Disorders of urinary system, possibly infection | Sepsis | 527 | 3.29 (2.76-3.93) | 33.47% |
| N39→E87 | Disorders of urinary system, possibly infection | Other disorders of fluid, electrolyte and acid-base balance | 615 | 2.78 (2.39-3.25) | 21.15% |
| N39→J12 | Disorders of urinary system, possibly infection | Pneumonia | 659 | 3.01 (2.58-3.51) | 14.70% |
| N39→K59 | Disorders of urinary system, possibly infection | Other functional intestinal disorders | 618 | 2.99 (2.55-3.49) | 18.96% |
| T80→B96 | Complications due to medical treatment | Bacterial infectious agents | 586 | 3.20 (2.71-3.78) | 38.67% |
| T80→E87 | Complications due to medical treatment | Other disorders of fluid, electrolyte and acid-base balance | 597 | 2.82 (2.41-3.29) | 17.87% |
| T80→J12 | Complications due to medical treatment | Pneumonia | 642 | 2.88 (2.47-3.35) | 13.58% |
| T80→K59 | Complications due to medical treatment | Other functional intestinal disorders | 549 | 2.74 (2.33-3.23) | 11.42% |
| T80→N17 | Complications due to medical treatment | Acute renal failure | 646 | 3.12 (2.67-3.65) | 15.31% |
| W00→E87 | Falls | Other disorders of fluid, electrolyte and acid-base balance | 598 | 2.74 (2.35-3.20) | 13.19% |
| W00→J12 | Falls | Pneumonia | 638 | 2.76 (2.38-3.21) | 11.89% |
| W00→K59 | Falls | Other functional intestinal disorders | 556 | 2.90 (2.46-3.41) | 15.02% |
| W00→N17 | Falls | Acute renal failure | 592 | 2.79 (2.39-3.27) | 14.13% |
| Y40→A09 | External causes of morbidity related to medical treatment | Infectious gastroenteritis and colitis | 663 | 2.08 (1.82-2.38) | 13.02% |
| Y40→A41 | External causes of morbidity related to medical treatment | Sepsis | 712 | 1.94 (1.71-2.20) | 23.97% |
| Y40→B96 | External causes of morbidity related to medical treatment | Bacterial infectious agents | 768 | 2.38 (2.09-2.71) | 26.20% |
| Y40→E87 | External causes of morbidity related to medical treatment | Other disorders of fluid, electrolyte and acid-base balance | 856 | 2.25 (1.99-2.53) | 25.93% |
| Y40→I95 | External causes of morbidity related to medical treatment | Hypotension | 584 | 2.06 (1.78-2.37) | 30.50% |
| Y40→J12 | External causes of morbidity related to medical treatment | Pneumonia | 936 | 2.23 (1.99-2.51) | 16.21% |
| Y40→K59 | External causes of morbidity related to medical treatment | Other functional intestinal disorders | 745 | 2.08 (1.83-2.36) | 19.73% |
| Y40→N17 | External causes of morbidity related to medical treatment | Acute renal failure | 913 | 2.38 (2.12-2.69) | 22.78% |

^*^Combined ICD-10 codes originates from the original ICD-10 code and are displayed in the Additional file 1.

^#^Number of the participants experienced the corresponding temporal disease trajectories.

**^&^**Number of individuals with high genetic susceptibility to MAFLD having the same D1 and D2 diagnosis date divided by the high genetic risk individuals diagnosed with both D1 and D2. A high value indicates a strong correlation between the two diseases and may exist a reverse causal relationship.

OR, odds ratio; CI, confidence interval. MAFLD, Metabolic dysfunction-associated fatty liver disease.

Additional file 1: Table S27. PheWAS using Cox regression was conducted to investigate the relationship between medical conditions and 7 causes of death in individuals with genetic susceptibility to MAFLD.

| **Medical conditions** | **Code*** | **No.#** | **HR (95% CI)** | **Causes of death** |
| --- | --- | --- | --- | --- |
| Infectious gastroenteritis and colitis | A09 | 330 | 2.16 (1.92-2.42) | CVDD |
| Sepsis | A41 | 545 | 4.87 (4.44-5.34) | CVDD |
| Mycoses | B49 | 181 | 3.37 (2.90-3.92) | CVDD |
| Bacterial infectious agents | B96 | 525 | 3.40 (3.09-3.73) | CVDD |
| Colon cancer | C18 | 69 | 1.68 (1.33-2.14) | CVDD |
| Metastatic cancer | C77 | 411 | 3.19 (2.87-3.54) | CVDD |
| Iron deficiency anaemia | D50 | 348 | 2.32 (2.07-2.59) | CVDD |
| Other anaemias | D51 | 608 | 3.25 (2.97-3.56) | CVDD |
| Hypothyroid conditions | E00 | 242 | 1.35 (1.19-1.54) | CVDD |
| Diabetes | E10 | 613 | 2.12 (1.94-2.32) | CVDD |
| Malnutrition | E40 | 154 | 2.00 (1.70-2.35) | CVDD |
| Obesity | E66 | 472 | 1.32 (1.20-1.46) | CVDD |
| Disorders of lipoprotein metabolism and other lipidaemias | E78 | 740 | 1.66 (1.52-1.80) | CVDD |
| Disorders of mineral metabolism | E83 | 196 | 2.95 (2.55-3.41) | CVDD |
| Volume depletion | E86 | 347 | 4.21 (3.76-4.70) | CVDD |
| Other disorders of fluid, electrolyte and acid-base balance | E87 | 897 | 6.17 (5.70-6.67) | CVDD |
| Delirium due to known physiological condition | F05 | 292 | 4.37 (3.87-4.93) | CVDD |
| Alcohol abuse | F10 | 163 | 2.91 (2.49-3.41) | CVDD |
| Tobacco abuse | F17 | 368 | 2.40 (2.15-2.68) | CVDD |
| Depression | F32 | 335 | 1.87 (1.67-2.10) | CVDD |
| Anxiety | F40 | 216 | 1.41 (1.23-1.62) | CVDD |
| Sleep disorder | G47 | 145 | 1.84 (1.56-2.18) | CVDD |
| Polyneuropathies | G60 | 143 | 2.98 (2.52-3.53) | CVDD |
| Cerebral palsy and other paralytic syndromes | G80 | 168 | 4.53 (3.88-5.29) | CVDD |
| Disorders of choroid and retina | H30 | 194 | 1.41 (1.22-1.63) | CVDD |
| Visual disturbances and blindness | H53 | 136 | 1.94 (1.64-2.31) | CVDD |
| Chronic rheumatic heart disease | I05 | 332 | 4.40 (3.93-4.93) | CVDD |
| Primary hypertension | I10 | 1233 | 2.14 (1.97-2.33) | CVDD |
| Angina pectoris | I20 | 378 | 2.32 (2.08-2.59) | CVDD |
| Acute myocardial infarction | I21 | 357 | 4.27 (3.83-4.77) | CVDD |
| Chronic ischaemic heart disease | I25 | 695 | 3.09 (2.83-3.37) | CVDD |
| Embolism and thrombosis | I26 | 404 | 3.74 (3.37-4.15) | CVDD |
| Non-rheumatic valve disorders | I34 | 380 | 3.61 (3.24-4.02) | CVDD |
| Atrial fibrillation and flutter | I48 | 805 | 3.74 (3.44-4.05) | CVDD |
| Other cardiac arrhythmias | I49 | 532 | 3.04 (2.77-3.34) | CVDD |
| Heart failure | I50 | 797 | 6.90 (6.36-7.49) | CVDD |
| Complications and ill-defined descriptions of heart disease | I51 | 498 | 3.87 (3.52-4.27) | CVDD |
| Stroke | I60 | 500 | 6.11 (5.55-6.72) | CVDD |
| Other cerebrovascular diseases | I65 | 324 | 3.50 (3.12-3.92) | CVDD |
| Hypotension | I95 | 537 | 4.53 (4.13-4.98) | CVDD |
| Pneumonia | J12 | 917 | 5.34 (4.94-5.77) | CVDD |
| Other acute Lower respiratory infections | J20 | 389 | 3.51 (3.16-3.91) | CVDD |
| Chronic obstructive pulmonary disease | J44 | 479 | 3.13 (2.84-3.45) | CVDD |
| Pleural effusion | J90 | 496 | 4.88 (4.43-5.38) | CVDD |
| Other diseases of the respiratory system | J95 | 296 | 3.01 (2.67-3.40) | CVDD |
| Respiratory failure | J96 | 454 | 6.87 (6.22-7.60) | CVDD |
| Oesophagitis | K20 | 114 | 1.42 (1.18-1.71) | CVDD |
| Other diseases of oesophagus | K22 | 157 | 1.39 (1.19-1.64) | CVDD |
| Gastric ulcer | K25 | 104 | 2.05 (1.69-2.50) | CVDD |
| Gastritis and duodenitis | K29 | 418 | 1.53 (1.38-1.70) | CVDD |
| Disease of stomach and duodenum | K31 | 186 | 1.39 (1.20-1.61) | CVDD |
| Umbilical hernia | K42 | 70 | 1.66 (1.31-2.11) | CVDD |
| Ventral hernia | K43 | 65 | 1.53 (1.20-1.95) | CVDD |
| Noninfective gastroenteritis and colitis | K52 | 189 | 1.88 (1.63-2.18) | CVDD |
| Other functional intestinal disorders | K59 | 448 | 2.42 (2.19-2.68) | CVDD |
| Other diseases of liver | K76 | 260 | 2.58 (2.27-2.93) | CVDD |
| Cholelithiasis | K80 | 207 | 1.31 (1.14-1.51) | CVDD |
| Disease of gallbladder and biliary tract | K82 | 115 | 1.95 (1.62-2.35) | CVDD |
| Other disease of digestive system | K92 | 305 | 2.35 (2.09-2.65) | CVDD |
| Skin and subcutaneous infections | L01 | 351 | 3.01 (2.69-3.36) | CVDD |
| Dermatitis | L20 | 120 | 2.04 (1.70-2.44) | CVDD |
| Pressure ulcer | L89 | 256 | 6.27 (5.51-7.13) | CVDD |
| Other inflammatory arthritis | M10 | 470 | 1.44 (1.30-1.59) | CVDD |
| Systemic connective tissue disorders | M30 | 90 | 1.56 (1.26-1.92) | CVDD |
| Spondylopathy | M48 | 162 | 1.77 (1.51-2.08) | CVDD |
| Dorsalgia | M54 | 256 | 1.40 (1.23-1.59) | CVDD |
| Other soft tissue disorders, not elsewhere classified | M79 | 268 | 1.73 (1.53-1.96) | CVDD |
| Obstructive and reflux uropathy | N13 | 91 | 2.21 (1.80-2.73) | CVDD |
| Acute renal failure | N17 | 981 | 6.16 (5.71-6.65) | CVDD |
| Chronic kidney disease | N18 | 660 | 3.50 (3.21-3.82) | CVDD |
| Other disorders of kidney and ureter | N29 | 138 | 2.26 (1.90-2.68) | CVDD |
| Disorders of urinary system, possibly infection | N39 | 577 | 2.86 (2.61-3.14) | CVDD |
| Complications due to medical treatment | T80 | 461 | 2.13 (1.92-2.35) | CVDD |
| Falls | W00 | 487 | 2.11 (1.91-2.32) | CVDD |
| Other external causes of accidental injury | W20 | 243 | 2.37 (2.07-2.70) | CVDD |
| External causes of morbidity related to medical treatment | Y40 | 643 | 2.40 (2.20-2.62) | CVDD |
| External causes of morbidity related to other conditions | Y95 | 317 | 6.11 (5.44-6.87) | CVDD |
| Infectious gastroenteritis and colitis | A09 | 178 | 4.53 (3.84-5.34) | DSSD |
| Sepsis | A41 | 244 | 8.68 (7.48-10.07) | DSSD |
| Mycoses | B49 | 71 | 4.70 (3.69-6.00) | DSSD |
| Bacterial infectious agents | B96 | 188 | 4.67 (3.97-5.50) | DSSD |
| Colon cancer | C18 | 39 | 3.38 (2.45-4.67) | DSSD |
| Metastatic cancer | C77 | 152 | 4.29 (3.60-5.12) | DSSD |
| Iron deficiency anaemia | D50 | 141 | 3.55 (2.96-4.26) | DSSD |
| Other anaemias | D51 | 261 | 5.85 (5.05-6.78) | DSSD |
| Diabetes | E10 | 176 | 2.05 (1.74-2.43) | DSSD |
| Malnutrition | E40 | 78 | 3.69 (2.92-4.65) | DSSD |
| Disorders of mineral metabolism | E83 | 93 | 5.09 (4.10-6.31) | DSSD |
| Volume depletion | E86 | 140 | 6.30 (5.26-7.56) | DSSD |
| Delirium due to known physiological condition | F05 | 85 | 4.41 (3.52-5.51) | DSSD |
| Tobacco abuse | F17 | 149 | 3.59 (3.01-4.28) | DSSD |
| Depression | F32 | 116 | 2.35 (1.93-2.86) | DSSD |
| Anxiety | F40 | 88 | 2.07 (1.66-2.58) | DSSD |
| Sleep disorder | G47 | 40 | 1.75 (1.28-2.41) | DSSD |
| Polyneuropathies | G60 | 46 | 3.38 (2.51-4.55) | DSSD |
| Cerebral palsy and other paralytic syndromes | G80 | 25 | 2.27 (1.52-3.38) | DSSD |
| Chronic rheumatic heart disease | I05 | 44 | 1.90 (1.40-2.57) | DSSD |
| Primary hypertension | I10 | 325 | 1.59 (1.36-1.85) | DSSD |
| Acute myocardial infarction | I21 | 48 | 1.82 (1.36-2.44) | DSSD |
| Chronic ischaemic heart disease | I25 | 123 | 1.49 (1.23-1.80) | DSSD |
| Embolism and thrombosis | I26 | 127 | 4.15 (3.44-5.01) | DSSD |
| Non-rheumatic valve disorders | I34 | 74 | 2.28 (1.80-2.89) | DSSD |
| Atrial fibrillation and flutter | I48 | 173 | 2.43 (2.06-2.87) | DSSD |
| Other cardiac arrhythmias | I49 | 106 | 1.94 (1.58-2.38) | DSSD |
| Heart failure | I50 | 173 | 4.57 (3.87-5.40) | DSSD |
| Complications and ill-defined descriptions of heart disease | I51 | 86 | 2.14 (1.71-2.67) | DSSD |
| Stroke | I60 | 48 | 1.79 (1.34-2.39) | DSSD |
| Other cerebrovascular diseases | I65 | 61 | 2.19 (1.69-2.84) | DSSD |
| Hypotension | I95 | 189 | 5.79 (4.93-6.81) | DSSD |
| Pneumonia | J12 | 253 | 5.11 (4.41-5.92) | DSSD |
| Other acute Lower respiratory infections | J20 | 103 | 3.22 (2.62-3.96) | DSSD |
| Chronic obstructive pulmonary disease | J44 | 116 | 2.53 (2.08-3.08) | DSSD |
| Pleural effusion | J90 | 191 | 7.06 (6.01-8.30) | DSSD |
| Other diseases of the respiratory system | J95 | 104 | 3.78 (3.08-4.64) | DSSD |
| Respiratory failure | J96 | 145 | 7.74 (6.47-9.25) | DSSD |
| Oesophagitis | K20 | 57 | 2.55 (1.95-3.33) | DSSD |
| Gastro-oesophageal reflux disease | K21 | 142 | 1.67 (1.39-2.00) | DSSD |
| Other diseases of oesophagus | K22 | 93 | 3.07 (2.48-3.81) | DSSD |
| Gastric ulcer | K25 | 55 | 3.95 (3.01-5.20) | DSSD |
| Gastritis and duodenitis | K29 | 201 | 3.15 (2.68-3.70) | DSSD |
| Disease of stomach and duodenum | K31 | 169 | 5.21 (4.40-6.16) | DSSD |
| Umbilical hernia | K42 | 38 | 3.24 (2.34-4.49) | DSSD |
| Ventral hernia | K43 | 44 | 3.74 (2.76-5.06) | DSSD |
| Diaphragmatic hernia | K44 | 141 | 1.87 (1.56-2.24) | DSSD |
| Noninfective gastroenteritis and colitis | K52 | 78 | 2.86 (2.27-3.61) | DSSD |
| Other functional intestinal disorders | K59 | 154 | 3.06 (2.57-3.64) | DSSD |
| Other diseases of anus and rectum | K62 | 70 | 1.56 (1.23-2.00) | DSSD |
| Other diseases of intestine | K63 | 130 | 2.13 (1.77-2.57) | DSSD |
| Other disorders of peritoneum | K66 | 45 | 2.76 (2.04-3.72) | DSSD |
| Other diseases of liver | K76 | 282 | 14.16 (12.26-16.34) | DSSD |
| Cholelithiasis | K80 | 122 | 2.96 (2.44-3.59) | DSSD |
| Disease of gallbladder and biliary tract | K82 | 99 | 6.41 (5.20-7.90) | DSSD |
| Other disease of digestive system | K92 | 200 | 6.47 (5.52-7.58) | DSSD |
| Skin and subcutaneous infections | L01 | 134 | 4.20 (3.49-5.05) | DSSD |
| Dermatitis | L20 | 36 | 2.13 (1.53-2.97) | DSSD |
| Pressure ulcer | L89 | 84 | 7.21 (5.75-9.03) | DSSD |
| Other soft tissue disorders, not elsewhere classified | M79 | 76 | 1.71 (1.35-2.16) | DSSD |
| Obstructive and reflux uropathy | N13 | 24 | 2.03 (1.35-3.05) | DSSD |
| Acute renal failure | N17 | 386 | 10.23 (8.94-11.69) | DSSD |
| Chronic kidney disease | N18 | 169 | 3.04 (2.57-3.60) | DSSD |
| Other disorders of kidney and ureter | N29 | 40 | 2.29 (1.67-3.15) | DSSD |
| Disorders of urinary system, possibly infection | N39 | 184 | 3.29 (2.79-3.87) | DSSD |
| Complications due to medical treatment | T80 | 180 | 3.17 (2.69-3.75) | DSSD |
| Falls | W00 | 158 | 2.52 (2.12-3.00) | DSSD |
| Other external causes of accidental injury | W20 | 78 | 2.69 (2.13-3.40) | DSSD |
| External causes of morbidity related to medical treatment | Y40 | 224 | 3.11 (2.66-3.63) | DSSD |
| External causes of morbidity related to other conditions | Y95 | 93 | 6.20 (5.00-7.69) | DSSD |
| Infectious gastroenteritis and colitis | A09 | 170 | 3.83 (3.24-4.52) | ESDD |
| Sepsis | A41 | 227 | 7.22 (6.22-8.39) | ESDD |
| Mycoses | B49 | 91 | 5.73 (4.61-7.11) | ESDD |
| Bacterial infectious agents | B96 | 221 | 5.09 (4.37-5.92) | ESDD |
| Metastatic cancer | C77 | 157 | 4.24 (3.56-5.03) | ESDD |
| Iron deficiency anaemia | D50 | 161 | 3.69 (3.11-4.37) | ESDD |
| Other anaemias | D51 | 256 | 4.95 (4.28-5.72) | ESDD |
| Hypothyroid conditions | E00 | 99 | 1.83 (1.49-2.26) | ESDD |
| Malnutrition | E40 | 64 | 2.69 (2.09-3.47) | ESDD |
| Obesity | E66 | 231 | 2.41 (2.08-2.80) | ESDD |
| Disorders of lipoprotein metabolism and other lipidaemias | E78 | 283 | 2.43 (2.10-2.82) | ESDD |
| Disorders of mineral metabolism | E83 | 94 | 4.75 (3.84-5.88) | ESDD |
| Volume depletion | E86 | 158 | 6.64 (5.60-7.88) | ESDD |
| Delirium due to known physiological condition | F05 | 127 | 6.32 (5.24-7.62) | ESDD |
| Alcohol abuse | F10 | 50 | 2.84 (2.13-3.77) | ESDD |
| Tobacco abuse | F17 | 119 | 2.48 (2.05-3.00) | ESDD |
| Depression | F32 | 126 | 2.31 (1.92-2.79) | ESDD |
| Anxiety | F40 | 76 | 1.58 (1.25-2.00) | ESDD |
| Sleep disorder | G47 | 81 | 3.44 (2.74-4.32) | ESDD |
| Polyneuropathies | G60 | 96 | 6.83 (5.54-8.44) | ESDD |
| Cerebral palsy and other paralytic syndromes | G80 | 50 | 4.27 (3.22-5.68) | ESDD |
| Disorders of the lens | H25 | 191 | 1.43 (1.22-1.68) | ESDD |
| Disorders of choroid and retina | H30 | 122 | 3.15 (2.61-3.82) | ESDD |
| Visual disturbances and blindness | H53 | 60 | 2.77 (2.13-3.59) | ESDD |
| Other disorders of the ear | H90 | 55 | 1.60 (1.22-2.10) | ESDD |
| Chronic rheumatic heart disease | I05 | 94 | 3.88 (3.14-4.80) | ESDD |
| Primary hypertension | I10 | 396 | 2.91 (2.47-3.43) | ESDD |
| Angina pectoris | I20 | 130 | 2.61 (2.16-3.14) | ESDD |
| Acute myocardial infarction | I21 | 108 | 4.08 (3.34-4.99) | ESDD |
| Chronic ischaemic heart disease | I25 | 221 | 3.16 (2.71-3.70) | ESDD |
| Embolism and thrombosis | I26 | 130 | 3.88 (3.22-4.66) | ESDD |
| Non-rheumatic valve disorders | I34 | 136 | 4.15 (3.46-4.98) | ESDD |
| Atrial fibrillation and flutter | I48 | 257 | 3.71 (3.21-4.28) | ESDD |
| Other cardiac arrhythmias | I49 | 182 | 3.31 (2.82-3.89) | ESDD |
| Heart failure | I50 | 261 | 7.33 (6.35-8.46) | ESDD |
| Complications and ill-defined descriptions of heart disease | I51 | 157 | 3.86 (3.25-4.58) | ESDD |
| Stroke | I60 | 112 | 4.11 (3.37-5.00) | ESDD |
| Other cerebrovascular diseases | I65 | 108 | 3.74 (3.06-4.57) | ESDD |
| Varicose veins of lower extremities | I83 | 31 | 1.78 (1.24-2.55) | ESDD |
| Hypotension | I95 | 213 | 6.11 (5.25-7.11) | ESDD |
| Pneumonia | J12 | 363 | 7.84 (6.87-8.95) | ESDD |
| Other acute Lower respiratory infections | J20 | 151 | 4.59 (3.85-5.46) | ESDD |
| Chronic obstructive pulmonary disease | J44 | 150 | 3.14 (2.64-3.74) | ESDD |
| Asthma | J45 | 103 | 1.51 (1.23-1.86) | ESDD |
| Pleural effusion | J90 | 172 | 5.63 (4.77-6.65) | ESDD |
| Other diseases of the respiratory system | J95 | 120 | 4.07 (3.36-4.92) | ESDD |
| Oesophagitis | K20 | 47 | 1.87 (1.39-2.50) | ESDD |
| Gastric ulcer | K25 | 43 | 2.70 (1.99-3.67) | ESDD |
| Gastritis and duodenitis | K29 | 188 | 2.36 (2.01-2.77) | ESDD |
| Disease of stomach and duodenum | K31 | 96 | 2.36 (1.91-2.91) | ESDD |
| Umbilical hernia | K42 | 35 | 2.70 (1.92-3.78) | ESDD |
| Noninfective gastroenteritis and colitis | K52 | 78 | 2.53 (2.00-3.18) | ESDD |
| Other functional intestinal disorders | K59 | 189 | 3.50 (2.98-4.10) | ESDD |
| Other diseases of liver | K76 | 123 | 4.14 (3.42-5.00) | ESDD |
| Cholelithiasis | K80 | 87 | 1.79 (1.43-2.23) | ESDD |
| Disease of gallbladder and biliary tract | K82 | 53 | 2.93 (2.22-3.87) | ESDD |
| Other disease of digestive system | K92 | 119 | 3.00 (2.48-3.64) | ESDD |
| Skin and subcutaneous infections | L01 | 162 | 4.90 (4.13-5.81) | ESDD |
| Dermatitis | L20 | 46 | 2.49 (1.85-3.35) | ESDD |
| Papulosquamous disorders | L40 | 25 | 1.91 (1.28-2.83) | ESDD |
| Autoimmune arthritis | M05 | 46 | 2.15 (1.60-2.89) | ESDD |
| Other inflammatory arthritis | M10 | 170 | 1.73 (1.47-2.05) | ESDD |
| Systemic connective tissue disorders | M30 | 33 | 1.82 (1.28-2.57) | ESDD |
| Spondylosis | M47 | 70 | 1.53 (1.20-1.95) | ESDD |
| Spondylopathy | M48 | 59 | 2.06 (1.58-2.68) | ESDD |
| Dorsalgia | M54 | 105 | 1.88 (1.53-2.30) | ESDD |
| Other soft tissue disorders, not elsewhere classified | M79 | 106 | 2.27 (1.85-2.78) | ESDD |
| Obstructive and reflux uropathy | N13 | 36 | 2.84 (2.04-3.97) | ESDD |
| Chronic kidney disease | N18 | 312 | 6.18 (5.40-7.08) | ESDD |
| Urolithiasis | N20 | 34 | 2.29 (1.62-3.22) | ESDD |
| Other disorders of kidney and ureter | N29 | 65 | 3.47 (2.70-4.46) | ESDD |
| Disorders of urinary system, possibly infection | N39 | 223 | 3.88 (3.34-4.52) | ESDD |
| Complications due to medical treatment | T80 | 179 | 2.87 (2.43-3.38) | ESDD |
| Falls | W00 | 186 | 2.68 (2.28-3.15) | ESDD |
| Other external causes of accidental injury | W20 | 87 | 2.74 (2.20-3.41) | ESDD |
| External causes of morbidity related to medical treatment | Y40 | 257 | 3.46 (2.99-4.00) | ESDD |
| Infectious gastroenteritis and colitis | A09 | 135 | 4.62 (3.82-5.58) | GSDD |
| Sepsis | A41 | 257 | 15.39 (13.14-18.02) | GSDD |
| Mycoses | B49 | 70 | 6.57 (5.13-8.42) | GSDD |
| Metastatic cancer | C77 | 99 | 3.93 (3.17-4.88) | GSDD |
| Benign tumors | D10 | 121 | 1.69 (1.39-2.07) | GSDD |
| Iron deficiency anaemia | D50 | 128 | 4.55 (3.75-5.53) | GSDD |
| Hypothyroid conditions | E00 | 71 | 1.95 (1.53-2.50) | GSDD |
| Diabetes | E10 | 145 | 2.98 (2.45-3.62) | GSDD |
| Malnutrition | E40 | 65 | 4.14 (3.21-5.35) | GSDD |
| Obesity | E66 | 149 | 2.12 (1.77-2.55) | GSDD |
| Disorders of lipoprotein metabolism and other lipidaemias | E78 | 187 | 2.18 (1.83-2.60) | GSDD |
| Disorders of mineral metabolism | E83 | 77 | 5.86 (4.62-7.43) | GSDD |
| Other disorders of fluid, electrolyte and acid-base balance | E87 | 341 | 17.42 (14.91-20.35) | GSDD |
| Alcohol abuse | F10 | 35 | 2.89 (2.06-4.07) | GSDD |
| Tobacco abuse | F17 | 84 | 2.57 (2.05-3.23) | GSDD |
| Depression | F32 | 83 | 2.19 (1.74-2.76) | GSDD |
| Anxiety | F40 | 53 | 1.61 (1.22-2.14) | GSDD |
| Sleep disorder | G47 | 53 | 3.28 (2.48-4.35) | GSDD |
| Diseases in nerves, nerve roots and nerve plexa | G50 | 57 | 1.76 (1.34-2.31) | GSDD |
| Polyneuropathies | G60 | 66 | 6.86 (5.32-8.85) | GSDD |
| Cerebral palsy and other paralytic syndromes | G80 | 33 | 4.15 (2.93-5.89) | GSDD |
| Disorders of the lens | H25 | 154 | 1.78 (1.49-2.14) | GSDD |
| Disorders of choroid and retina | H30 | 83 | 3.07 (2.44-3.87) | GSDD |
| Visual disturbances and blindness | H53 | 40 | 2.69 (1.95-3.70) | GSDD |
| Chronic rheumatic heart disease | I05 | 92 | 5.84 (4.68-7.28) | GSDD |
| Primary hypertension | I10 | 268 | 3.66 (2.95-4.54) | GSDD |
| Angina pectoris | I20 | 106 | 3.19 (2.59-3.94) | GSDD |
| Acute myocardial infarction | I21 | 73 | 4.02 (3.15-5.13) | GSDD |
| Chronic ischaemic heart disease | I25 | 173 | 3.79 (3.17-4.54) | GSDD |
| Embolism and thrombosis | I26 | 84 | 3.67 (2.92-4.61) | GSDD |
| Non-rheumatic valve disorders | I34 | 117 | 5.51 (4.51-6.73) | GSDD |
| Atrial fibrillation and flutter | I48 | 228 | 5.68 (4.82-6.69) | GSDD |
| Other cardiac arrhythmias | I49 | 146 | 4.06 (3.38-4.89) | GSDD |
| Complications and ill-defined descriptions of heart disease | I51 | 151 | 5.85 (4.88-7.01) | GSDD |
| Stroke | I60 | 65 | 3.46 (2.67-4.46) | GSDD |
| Other cerebrovascular diseases | I65 | 88 | 4.57 (3.65-5.73) | GSDD |
| Varicose veins of lower extremities | I83 | 27 | 2.27 (1.54-3.33) | GSDD |
| Pneumonia | J12 | 285 | 10.09 (8.63-11.80) | GSDD |
| Other acute Lower respiratory infections | J20 | 133 | 6.27 (5.18-7.59) | GSDD |
| Chronic obstructive pulmonary disease | J44 | 119 | 3.79 (3.10-4.62) | GSDD |
| Other diseases of the respiratory system | J95 | 84 | 4.22 (3.36-5.31) | GSDD |
| Other diseases of oesophagus | K22 | 42 | 1.74 (1.27-2.38) | GSDD |
| Gastric ulcer | K25 | 30 | 2.81 (1.95-4.05) | GSDD |
| Gastritis and duodenitis | K29 | 116 | 2.12 (1.73-2.60) | GSDD |
| Disease of stomach and duodenum | K31 | 52 | 1.85 (1.39-2.45) | GSDD |
| Umbilical hernia | K42 | 31 | 3.53 (2.46-5.06) | GSDD |
| Noninfective gastroenteritis and colitis | K52 | 74 | 3.68 (2.89-4.69) | GSDD |
| Other functional intestinal disorders | K59 | 146 | 4.11 (3.42-4.95) | GSDD |
| Other diseases of intestine | K63 | 78 | 1.62 (1.28-2.06) | GSDD |
| Other disorders of peritoneum | K66 | 25 | 2.00 (1.34-2.98) | GSDD |
| Other diseases of liver | K76 | 78 | 3.83 (3.02-4.85) | GSDD |
| Cholelithiasis | K80 | 69 | 2.12 (1.66-2.73) | GSDD |
| Disease of gallbladder and biliary tract | K82 | 45 | 3.72 (2.75-5.03) | GSDD |
| Other disease of digestive system | K92 | 102 | 3.95 (3.20-4.88) | GSDD |
| Skin and subcutaneous infections | L01 | 133 | 6.09 (5.03-7.37) | GSDD |
| Dermatitis | L20 | 45 | 3.64 (2.69-4.93) | GSDD |
| Papulosquamous disorders | L40 | 25 | 2.83 (1.90-4.22) | GSDD |
| Pressure ulcer | L89 | 99 | 4483.06 (2526.70-7954.16) | GSDD |
| Autoimmune arthritis | M05 | 34 | 2.33 (1.65-3.29) | GSDD |
| Other inflammatory arthritis | M10 | 158 | 2.55 (2.13-3.06) | GSDD |
| Osteoarthritis | M15 | 179 | 1.47 (1.23-1.75) | GSDD |
| Other joint disorders | M20 | 94 | 1.62 (1.30-2.02) | GSDD |
| Systemic connective tissue disorders | M30 | 32 | 2.63 (1.84-3.75) | GSDD |
| Deforming dorsopathies | M40 | 28 | 2.29 (1.57-3.34) | GSDD |
| Spondylopathy | M48 | 42 | 2.15 (1.57-2.93) | GSDD |
| Dorsalgia | M54 | 77 | 2.07 (1.63-2.62) | GSDD |
| Other soft tissue disorders, not elsewhere classified | M79 | 103 | 3.44 (2.78-4.25) | GSDD |
| Obstructive and reflux uropathy | N13 | 60 | 7.52 (5.76-9.80) | GSDD |
| Chronic kidney disease | N18 | 392 | 21.70 (18.45-25.53) | GSDD |
| Urolithiasis | N20 | 24 | 2.41 (1.60-3.62) | GSDD |
| Other disorders of kidney and ureter | N29 | 84 | 7.18 (5.71-9.03) | GSDD |
| Bladder disorder | N32 | 49 | 2.52 (1.89-3.38) | GSDD |
| Disorders of urinary system, possibly infection | N39 | 217 | 6.48 (5.49-7.64) | GSDD |
| Complications due to medical treatment | T80 | 182 | 4.84 (4.06-5.76) | GSDD |
| Falls | W00 | 156 | 3.48 (2.91-4.17) | GSDD |
| Other external causes of accidental injury | W20 | 70 | 3.25 (2.54-4.17) | GSDD |
| External causes of morbidity related to medical treatment | Y40 | 222 | 5.06 (4.28-5.97) | GSDD |
| Infectious gastroenteritis and colitis | A09 | 577 | 2.67 (2.45-2.91) | MND |
| Sepsis | A41 | 1037 | 6.70 (6.24-7.18) | MND |
| Mycoses | B49 | 396 | 5.13 (4.62-5.69) | MND |
| Bacterial infectious agents | B96 | 730 | 3.17 (2.93-3.44) | MND |
| Colon cancer | C18 | 356 | 6.37 (5.71-7.10) | MND |
| Breast cancer | C50 | 297 | 2.89 (2.57-3.25) | MND |
| Benign tumors | D10 | 673 | 1.38 (1.27-1.50) | MND |
| Iron deficiency anaemia | D50 | 333 | 1.47 (1.31-1.64) | MND |
| Other anaemias | D51 | 1077 | 4.15 (3.87-4.45) | MND |
| Hypothyroid conditions | E00 | 357 | 1.38 (1.24-1.54) | MND |
| Diabetes | E10 | 749 | 1.55 (1.43-1.67) | MND |
| Malnutrition | E40 | 163 | 1.45 (1.24-1.69) | MND |
| Disorders of lipoprotein metabolism and other lipidaemias | E78 | 902 | 1.18 (1.10-1.27) | MND |
| Disorders of mineral metabolism | E83 | 516 | 5.58 (5.09-6.12) | MND |
| Volume depletion | E86 | 566 | 4.73 (4.33-5.16) | MND |
| Other disorders of fluid, electrolyte and acid-base balance | E87 | 1050 | 4.45 (4.15-4.77) | MND |
| Delirium due to known physiological condition | F05 | 219 | 2.13 (1.86-2.44) | MND |
| Tobacco abuse | F17 | 468 | 2.08 (1.89-2.28) | MND |
| Depression | F32 | 387 | 1.44 (1.30-1.60) | MND |
| Anxiety | F40 | 296 | 1.34 (1.19-1.51) | MND |
| Polyneuropathies | G60 | 164 | 2.34 (2.00-2.73) | MND |
| Cerebral palsy and other paralytic syndromes | G80 | 142 | 2.54 (2.15-3.01) | MND |
| Primary hypertension | I10 | 1628 | 1.31 (1.23-1.40) | MND |
| Embolism and thrombosis | I26 | 773 | 5.11 (4.73-5.52) | MND |
| Atrial fibrillation and flutter | I48 | 624 | 1.60 (1.47-1.75) | MND |
| Heart failure | I50 | 339 | 1.51 (1.35-1.69) | MND |
| Stroke | I60 | 244 | 1.79 (1.58-2.04) | MND |
| Other cerebrovascular diseases | I65 | 196 | 1.36 (1.17-1.56) | MND |
| Hypotension | I95 | 524 | 2.79 (2.55-3.06) | MND |
| Pneumonia | J12 | 1225 | 4.61 (4.31-4.92) | MND |
| Other acute Lower respiratory infections | J20 | 615 | 3.76 (3.45-4.09) | MND |
| Chronic obstructive pulmonary disease | J44 | 489 | 2.04 (1.86-2.24) | MND |
| Pleural effusion | J90 | 964 | 6.79 (6.32-7.29) | MND |
| Other diseases of the respiratory system | J95 | 544 | 3.90 (3.57-4.27) | MND |
| Respiratory failure | J96 | 328 | 3.02 (2.70-3.38) | MND |
| Other diseases of oesophagus | K22 | 267 | 1.66 (1.47-1.88) | MND |
| Gastric ulcer | K25 | 105 | 1.42 (1.17-1.72) | MND |
| Gastritis and duodenitis | K29 | 512 | 1.25 (1.14-1.37) | MND |
| Disease of stomach and duodenum | K31 | 288 | 1.50 (1.33-1.69) | MND |
| Ventral hernia | K43 | 117 | 1.93 (1.61-2.32) | MND |
| Noninfective gastroenteritis and colitis | K52 | 380 | 2.70 (2.43-2.99) | MND |
| Other functional intestinal disorders | K59 | 929 | 3.77 (3.50-4.05) | MND |
| Other disorders of peritoneum | K66 | 182 | 2.19 (1.89-2.54) | MND |
| Other diseases of liver | K76 | 361 | 2.46 (2.21-2.74) | MND |
| Cholelithiasis | K80 | 330 | 1.49 (1.33-1.66) | MND |
| Disease of gallbladder and biliary tract | K82 | 351 | 4.29 (3.84-4.78) | MND |
| Other disease of digestive system | K92 | 431 | 2.28 (2.06-2.52) | MND |
| Skin and subcutaneous infections | L01 | 347 | 1.95 (1.74-2.17) | MND |
| Dermatitis | L20 | 149 | 1.75 (1.48-2.06) | MND |
| Pressure ulcer | L89 | 283 | 4.48 (3.97-5.06) | MND |
| Spondylopathy | M48 | 177 | 1.34 (1.15-1.55) | MND |
| Dorsalgia | M54 | 413 | 1.58 (1.43-1.75) | MND |
| Other soft tissue disorders, not elsewhere classified | M79 | 384 | 1.71 (1.54-1.90) | MND |
| Obstructive and reflux uropathy | N13 | 338 | 5.90 (5.28-6.60) | MND |
| Acute renal failure | N17 | 1083 | 4.11 (3.83-4.40) | MND |
| Chronic kidney disease | N18 | 480 | 1.53 (1.39-1.68) | MND |
| Other disorders of kidney and ureter | N29 | 184 | 2.07 (1.78-2.40) | MND |
| Bladder disorder | N32 | 182 | 1.35 (1.17-1.57) | MND |
| Disorders of urinary system, possibly infection | N39 | 804 | 2.72 (2.52-2.94) | MND |
| Complications due to medical treatment | T80 | 785 | 2.56 (2.37-2.77) | MND |
| Falls | W00 | 454 | 1.27 (1.15-1.40) | MND |
| Other external causes of accidental injury | W20 | 221 | 1.43 (1.25-1.64) | MND |
| External causes of morbidity related to medical treatment | Y40 | 1324 | 3.86 (3.62-4.12) | MND |
| External causes of morbidity related to other conditions | Y95 | 315 | 3.90 (3.48-4.37) | MND |
| Infectious gastroenteritis and colitis | A09 | 280 | 3.02 (2.67-3.43) | RSDD |
| Mycoses | B49 | 188 | 5.89 (5.07-6.85) | RSDD |
| Bacterial infectious agents | B96 | 435 | 4.82 (4.33-5.37) | RSDD |
| Metastatic cancer | C77 | 345 | 4.77 (4.24-5.37) | RSDD |
| Iron deficiency anaemia | D50 | 263 | 2.83 (2.48-3.22) | RSDD |
| Other anaemias | D51 | 438 | 3.89 (3.50-4.33) | RSDD |
| Hypothyroid conditions | E00 | 178 | 1.59 (1.36-1.86) | RSDD |
| Diabetes | E10 | 397 | 2.07 (1.85-2.31) | RSDD |
| Malnutrition | E40 | 144 | 3.02 (2.55-3.58) | RSDD |
| Obesity | E66 | 339 | 1.51 (1.34-1.70) | RSDD |
| Disorders of lipoprotein metabolism and other lipidaemias | E78 | 483 | 1.62 (1.46-1.80) | RSDD |
| Disorders of mineral metabolism | E83 | 172 | 4.27 (3.65-5.00) | RSDD |
| Volume depletion | E86 | 309 | 6.36 (5.63-7.19) | RSDD |
| Other disorders of fluid, electrolyte and acid-base balance | E87 | 685 | 8.25 (7.51-9.05) | RSDD |
| Delirium due to known physiological condition | F05 | 256 | 6.34 (5.56-7.23) | RSDD |
| Alcohol abuse | F10 | 124 | 3.55 (2.96-4.26) | RSDD |
| Tobacco abuse | F17 | 322 | 3.51 (3.12-3.96) | RSDD |
| Depression | F32 | 270 | 2.47 (2.17-2.81) | RSDD |
| Anxiety | F40 | 196 | 2.08 (1.79-2.41) | RSDD |
| Sleep disorder | G47 | 111 | 2.23 (1.84-2.70) | RSDD |
| Polyneuropathies | G60 | 103 | 3.43 (2.81-4.18) | RSDD |
| Cerebral palsy and other paralytic syndromes | G80 | 102 | 4.32 (3.54-5.28) | RSDD |
| Disorders of choroid and retina | H30 | 126 | 1.42 (1.19-1.70) | RSDD |
| Visual disturbances and blindness | H53 | 92 | 2.06 (1.67-2.54) | RSDD |
| Other disorders of the ear | H90 | 111 | 1.61 (1.33-1.95) | RSDD |
| Chronic rheumatic heart disease | I05 | 158 | 3.19 (2.71-3.75) | RSDD |
| Primary hypertension | I10 | 817 | 2.02 (1.82-2.24) | RSDD |
| Angina pectoris | I20 | 209 | 1.91 (1.66-2.21) | RSDD |
| Acute myocardial infarction | I21 | 146 | 2.56 (2.16-3.03) | RSDD |
| Chronic ischaemic heart disease | I25 | 363 | 2.22 (1.98-2.49) | RSDD |
| Embolism and thrombosis | I26 | 253 | 3.71 (3.25-4.24) | RSDD |
| Non-rheumatic valve disorders | I34 | 181 | 2.56 (2.19-2.98) | RSDD |
| Atrial fibrillation and flutter | I48 | 508 | 3.56 (3.21-3.94) | RSDD |
| Other cardiac arrhythmias | I49 | 306 | 2.64 (2.34-2.99) | RSDD |
| Heart failure | I50 | 480 | 6.25 (5.64-6.93) | RSDD |
| Complications and ill-defined descriptions of heart disease | I51 | 295 | 3.53 (3.12-3.99) | RSDD |
| Stroke | I60 | 192 | 3.40 (2.93-3.95) | RSDD |
| Other cerebrovascular diseases | I65 | 199 | 3.35 (2.90-3.88) | RSDD |
| Hypotension | I95 | 388 | 5.36 (4.79-5.99) | RSDD |
| Pneumonia | J12 | 1139 | 18.93 (17.27-20.76) | RSDD |
| Other acute Lower respiratory infections | J20 | 379 | 5.99 (5.35-6.70) | RSDD |
| Chronic obstructive pulmonary disease | J44 | 561 | 7.27 (6.58-8.03) | RSDD |
| Asthma | J45 | 244 | 1.83 (1.59-2.09) | RSDD |
| Pleural effusion | J90 | 402 | 6.78 (6.07-7.57) | RSDD |
| Other diseases of the respiratory system | J95 | 307 | 5.35 (4.74-6.05) | RSDD |
| Oesophagitis | K20 | 88 | 1.73 (1.40-2.14) | RSDD |
| Gastro-oesophageal reflux disease | K21 | 272 | 1.36 (1.20-1.55) | RSDD |
| Other diseases of oesophagus | K22 | 131 | 1.86 (1.56-2.22) | RSDD |
| Gastric ulcer | K25 | 63 | 1.96 (1.52-2.51) | RSDD |
| Gastritis and duodenitis | K29 | 283 | 1.66 (1.46-1.89) | RSDD |
| Disease of stomach and duodenum | K31 | 160 | 1.92 (1.63-2.26) | RSDD |
| Umbilical hernia | K42 | 45 | 1.68 (1.25-2.26) | RSDD |
| Diaphragmatic hernia | K44 | 267 | 1.50 (1.32-1.71) | RSDD |
| Noninfective gastroenteritis and colitis | K52 | 137 | 2.19 (1.84-2.61) | RSDD |
| Other functional intestinal disorders | K59 | 401 | 3.73 (3.34-4.17) | RSDD |
| Other diseases of liver | K76 | 183 | 2.92 (2.51-3.40) | RSDD |
| Cholelithiasis | K80 | 170 | 1.72 (1.47-2.01) | RSDD |
| Disease of gallbladder and biliary tract | K82 | 94 | 2.56 (2.08-3.14) | RSDD |
| Other disease of digestive system | K92 | 188 | 2.28 (1.96-2.65) | RSDD |
| Skin and subcutaneous infections | L01 | 273 | 3.84 (3.38-4.36) | RSDD |
| Dermatitis | L20 | 91 | 2.45 (1.98-3.02) | RSDD |
| Papulosquamous disorders | L40 | 57 | 2.16 (1.66-2.80) | RSDD |
| Autoimmune arthritis | M05 | 84 | 1.93 (1.55-2.40) | RSDD |
| Other inflammatory arthritis | M10 | 315 | 1.53 (1.35-1.72) | RSDD |
| Other joint disorders | M20 | 242 | 1.40 (1.22-1.60) | RSDD |
| Systemic connective tissue disorders | M30 | 78 | 2.15 (1.71-2.69) | RSDD |
| Deforming dorsopathies | M40 | 70 | 1.92 (1.51-2.44) | RSDD |
| Spondylosis | M47 | 143 | 1.55 (1.31-1.84) | RSDD |
| Spondylopathy | M48 | 116 | 2.01 (1.66-2.42) | RSDD |
| Dorsalgia | M54 | 210 | 1.88 (1.63-2.17) | RSDD |
| Other soft tissue disorders, not elsewhere classified | M79 | 197 | 2.05 (1.77-2.37) | RSDD |
| Acute renal failure | N17 | 724 | 7.87 (7.18-8.63) | RSDD |
| Chronic kidney disease | N18 | 413 | 3.40 (3.05-3.79) | RSDD |
| Other disorders of kidney and ureter | N29 | 79 | 2.03 (1.62-2.54) | RSDD |
| Disorders of urinary system, possibly infection | N39 | 442 | 3.70 (3.33-4.12) | RSDD |
| Complications due to medical treatment | T80 | 325 | 2.40 (2.13-2.71) | RSDD |
| Falls | W00 | 390 | 2.83 (2.53-3.16) | RSDD |
| Other external causes of accidental injury | W20 | 202 | 3.19 (2.75-3.69) | RSDD |
| External causes of morbidity related to medical treatment | Y40 | 491 | 3.06 (2.76-3.39) | RSDD |

^*^Combined ICD-10 codes originates from the original ICD-10 code and are displayed in the Additional file 1.

^#^Number of the participants with genetic susceptibility to MAFLD dead after diagnosing corresponding medical conditions.

HR, hazards ratio; CI, confidence interval. MAFLD, metabolic dysfunction-associated fatty liver disease; MND, malignant neoplasms death; ESDD, endocrine system disease death; CVDD, cardiovascular disease death; RSDD, respiratory system disease death; DSSD, digestive system disease death; GSDD, genitourinary system disease death.

Additional file 1: Table S28. Temporal disease pairs with a significantly increased risk of disease 2 (D2) after disease 1 (D1) in dead individuals with genetic susceptibility to MAFLD.

| **Causes of death** | **D1→D2 code^*^** | **D1 description** | **D2 description** | **No.^#^** | **OR (95% CI)** | **Percentage^&^** |
| --- | --- | --- | --- | --- | --- | --- |
| CVDD | D51→E87 | Other anaemias | Other disorders of fluid, electrolyte and acid-base balance | 189 | 4.26 (3.13-5.80) | 14.24% |
| CVDD | D51→J12 | Other anaemias | Pneumonia | 161 | 4.56 (3.23-6.45) | 9.89% |
| CVDD | D51→N17 | Other anaemias | Acute renal failure | 183 | 5.30 (3.78-7.43) | 15.91% |
| CVDD | E10→E87 | Diabetes | Other disorders of fluid, electrolyte and acid-base balance | 170 | 4.87 (3.45-6.88) | 13.60% |
| CVDD | E10→J12 | Diabetes | Pneumonia | 168 | 4.56 (3.25-6.40) | 14.85% |
| CVDD | E10→N17 | Diabetes | Acute renal failure | 198 | 5.55 (3.96-7.77) | 11.58% |
| CVDD | E78→E87 | Disorders of lipoprotein metabolism and other lipidaemias | Other disorders of fluid, electrolyte and acid-base balance | 223 | 3.23 (2.49-4.18) | 12.50% |
| CVDD | E78→I50 | Disorders of lipoprotein metabolism and other lipidaemias | Heart failure | 169 | 2.79 (2.10-3.73) | 14.45% |
| CVDD | E78→J12 | Disorders of lipoprotein metabolism and other lipidaemias | Pneumonia | 214 | 4.26 (3.20-5.67) | 8.99% |
| CVDD | E78→N17 | Disorders of lipoprotein metabolism and other lipidaemias | Acute renal failure | 217 | 3.72 (2.83-4.90) | 13.86% |
| CVDD | I10→A41 | Primary hypertension | Sepsis | 223 | 1.54 (1.27-1.87) | 8.33% |
| CVDD | I10→D51 | Primary hypertension | Other anaemias | 223 | 1.64 (1.34-2.00) | 10.25% |
| CVDD | I10→E78 | Primary hypertension | Disorders of lipoprotein metabolism and other lipidaemias | 201 | 1.91 (1.54-2.36) | 44.88% |
| CVDD | I10→E87 | Primary hypertension | Other disorders of fluid, electrolyte and acid-base balance | 352 | 1.83 (1.56-2.15) | 13.32% |
| CVDD | I10→I25 | Primary hypertension | Chronic ischaemic heart disease | 193 | 1.55 (1.25-1.91) | 27.68% |
| CVDD | I10→I48 | Primary hypertension | Atrial fibrillation and flutter | 237 | 1.52 (1.26-1.84) | 22.36% |
| CVDD | I10→J12 | Primary hypertension | Pneumonia | 324 | 2.05 (1.72-2.43) | 13.06% |
| CVDD | I10→J90 | Primary hypertension | Pleural effusion | 168 | 1.57 (1.26-1.97) | 11.37% |
| CVDD | I10→N17 | Primary hypertension | Acute renal failure | 366 | 2.12 (1.80-2.50) | 13.26% |
| CVDD | I10→Y40 | Primary hypertension | External causes of morbidity related to medical treatment | 209 | 1.84 (1.50-2.27) | 12.71% |
| CVDD | I25→E87 | Chronic ischaemic heart disease | Other disorders of fluid, electrolyte and acid-base balance | 188 | 7.10 (4.82-10.48) | 13.90% |
| CVDD | I25→J12 | Chronic ischaemic heart disease | Pneumonia | 174 | 4.77 (3.38-6.74) | 12.23% |
| CVDD | I25→N17 | Chronic ischaemic heart disease | Acute renal failure | 205 | 4.77 (3.48-6.53) | 12.58% |
| CVDD | I48→E87 | Atrial fibrillation and flutter | Other disorders of fluid, electrolyte and acid-base balance | 221 | 4.56 (3.39-6.12) | 22.45% |
| CVDD | I48→I50 | Atrial fibrillation and flutter | Heart failure | 191 | 4.33 (3.17-5.93) | 27.34% |
| CVDD | I48→J12 | Atrial fibrillation and flutter | Pneumonia | 204 | 5.56 (3.99-7.75) | 20.89% |
| CVDD | I48→J90 | Atrial fibrillation and flutter | Pleural effusion | 158 | 3.91 (2.81-5.44) | 20.46% |
| CVDD | I48→N17 | Atrial fibrillation and flutter | Acute renal failure | 219 | 6.10 (4.37-8.52) | 21.77% |
| CVDD | I48→N18 | Atrial fibrillation and flutter | Chronic kidney disease | 162 | 3.80 (2.75-5.26) | 13.09% |
| CVDD | I50→E87 | Heart failure | Other disorders of fluid, electrolyte and acid-base balance | 212 | 7.03 (4.86-10.18) | 25.71% |
| CVDD | I50→N17 | Heart failure | Acute renal failure | 218 | 8.85 (5.90-13.27) | 23.50% |
| CVDD | N18→E87 | Chronic kidney disease | Other disorders of fluid, electrolyte and acid-base balance | 193 | 9.04 (5.88-13.91) | 21.29% |
| CVDD | N18→J12 | Chronic kidney disease | Pneumonia | 180 | 6.79 (4.60-10.03) | 13.98% |
| CVDD | Y40→E87 | External causes of morbidity related to medical treatment | Other disorders of fluid, electrolyte and acid-base balance | 155 | 3.60 (2.62-4.96) | 19.05% |
| CVDD | Y40→J12 | External causes of morbidity related to medical treatment | Pneumonia | 173 | 4.51 (3.24-6.28) | 13.54% |
| CVDD | Y40→N17 | External causes of morbidity related to medical treatment | Acute renal failure | 169 | 4.08 (2.98-5.60) | 20.61% |
| DSSD | D51→A41 | Other anaemias | Sepsis | 62 | 3.89 (2.32-6.53) | 14.29% |
| DSSD | D51→J12 | Other anaemias | Pneumonia | 58 | 3.88 (2.24-6.71) | 16.49% |
| DSSD | D51→J90 | Other anaemias | Pleural effusion | 55 | 2.74 (1.70-4.42) | 12.82% |
| DSSD | E10→K76 | Diabetes | Other diseases of liver | 50 | 2.43 (1.46-4.04) | 11.76% |
| DSSD | E10→N17 | Diabetes | Acute renal failure | 74 | 5.37 (3.15-9.16) | 11.58% |
| DSSD | F17→N17 | Tobacco abuse | Acute renal failure | 45 | 5.33 (2.62-10.87) | 22.54% |
| DSSD | I10→D51 | Primary hypertension | Other anaemias | 77 | 1.85 (1.32-2.59) | 10.19% |
| DSSD | I10→J12 | Primary hypertension | Pneumonia | 85 | 2.97 (2.06-4.29) | 7.84% |
| DSSD | I10→N17 | Primary hypertension | Acute renal failure | 134 | 2.04 (1.57-2.64) | 12.57% |
| DSSD | I10→Y40 | Primary hypertension | External causes of morbidity related to medical treatment | 67 | 2.07 (1.43-3.01) | 8.42% |
| DSSD | I48→N17 | Atrial fibrillation and flutter | Acute renal failure | 52 | 5.08 (2.74-9.44) | 28.57% |
| DSSD | K29→A09 | Gastritis and duodenitis | Infectious gastroenteritis and colitis | 47 | 5.88 (2.78-12.43) | 0.00% |
| DSSD | K29→A41 | Gastritis and duodenitis | Sepsis | 52 | 5.50 (2.80-10.79) | 3.08% |
| DSSD | K29→J12 | Gastritis and duodenitis | Pneumonia | 59 | 10.50 (4.54-24.26) | 6.25% |
| DSSD | K29→N17 | Gastritis and duodenitis | Acute renal failure | 70 | 6.82 (3.62-12.84) | 4.00% |
| DSSD | K31→N17 | Disease of stomach and duodenum | Acute renal failure | 61 | 6.20 (3.18-12.09) | 4.55% |
| DSSD | K44→N17 | Diaphragmatic hernia | Acute renal failure | 49 | 8.50 (3.65-19.81) | 1.45% |
| DSSD | K76→J12 | Other diseases of liver | Pneumonia | 61 | 9.43 (4.33-20.55) | 15.00% |
| DSSD | K76→J90 | Other diseases of liver | Pleural effusion | 53 | 4.58 (2.45-8.56) | 15.38% |
| DSSD | K76→K31 | Other diseases of liver | Disease of stomach and duodenum | 54 | 10.80 (4.32-27.00) | 36.13% |
| DSSD | K76→N17 | Other diseases of liver | Acute renal failure | 83 | 7.82 (4.17-14.64) | 16.88% |
| DSSD | Y40→A41 | External causes of morbidity related to medical treatment | Sepsis | 53 | 2.61 (1.61-4.22) | 11.90% |
| DSSD | Y40→J12 | External causes of morbidity related to medical treatment | Pneumonia | 55 | 6.09 (3.22-11.52) | 9.88% |
| ESDD | D50→J12 | Iron deficiency anaemia | Pneumonia | 59 | 9.00 (4.12-19.65) | 3.45% |
| ESDD | D51→A41 | Other anaemias | Sepsis | 63 | 4.69 (2.73-8.04) | 11.00% |
| ESDD | D51→J12 | Other anaemias | Pneumonia | 87 | 4.68 (2.95-7.42) | 12.14% |
| ESDD | E66→A41 | Obesity | Sepsis | 54 | 4.92 (2.64-9.15) | 6.94% |
| ESDD | E66→J12 | Obesity | Pneumonia | 83 | 6.13 (3.61-10.39) | 6.03% |
| ESDD | E78→A41 | Disorders of lipoprotein metabolism and other lipidaemias | Sepsis | 60 | 3.09 (1.91-5.00) | 7.89% |
| ESDD | E78→B96 | Disorders of lipoprotein metabolism and other lipidaemias | Bacterial infectious agents | 61 | 4.27 (2.43-7.49) | 3.49% |
| ESDD | E78→I50 | Disorders of lipoprotein metabolism and other lipidaemias | Heart failure | 66 | 4.33 (2.60-7.23) | 11.54% |
| ESDD | E78→I95 | Disorders of lipoprotein metabolism and other lipidaemias | Hypotension | 54 | 3.22 (1.90-5.47) | 15.19% |
| ESDD | E78→J12 | Disorders of lipoprotein metabolism and other lipidaemias | Pneumonia | 108 | 5.86 (3.69-9.30) | 9.63% |
| ESDD | E78→J90 | Disorders of lipoprotein metabolism and other lipidaemias | Pleural effusion | 52 | 3.87 (2.19-6.82) | 6.15% |
| ESDD | E78→K59 | Disorders of lipoprotein metabolism and other lipidaemias | Other functional intestinal disorders | 51 | 3.18 (1.84-5.48) | 3.28% |
| ESDD | E78→N18 | Disorders of lipoprotein metabolism and other lipidaemias | Chronic kidney disease | 75 | 3.36 (2.15-5.25) | 9.57% |
| ESDD | E78→N39 | Disorders of lipoprotein metabolism and other lipidaemias | Disorders of urinary system, possibly infection | 52 | 4.00 (2.23-7.18) | 3.80% |
| ESDD | E78→Y40 | Disorders of lipoprotein metabolism and other lipidaemias | External causes of morbidity related to medical treatment | 61 | 4.50 (2.62-7.74) | 8.42% |
| ESDD | H25→J12 | Disorders of the lens | Pneumonia | 65 | 4.60 (2.63-8.04) | 1.18% |
| ESDD | H25→N18 | Disorders of the lens | Chronic kidney disease | 56 | 5.64 (2.97-10.70) | 8.43% |
| ESDD | H25→N39 | Disorders of the lens | Disorders of urinary system, possibly infection | 50 | 5.00 (2.62-9.55) | 0.00% |
| ESDD | I10→A41 | Primary hypertension | Sepsis | 82 | 1.95 (1.41-2.69) | 6.12% |
| ESDD | I10→C77 | Primary hypertension | Metastatic cancer | 58 | 2.11 (1.43-3.12) | 14.10% |
| ESDD | I10→E78 | Primary hypertension | Disorders of lipoprotein metabolism and other lipidaemias | 69 | 2.15 (1.48-3.11) | 46.54% |
| ESDD | I10→I48 | Primary hypertension | Atrial fibrillation and flutter | 78 | 1.94 (1.38-2.73) | 11.61% |
| ESDD | I10→J12 | Primary hypertension | Pneumonia | 129 | 2.57 (1.94-3.41) | 10.69% |
| ESDD | I10→N39 | Primary hypertension | Disorders of urinary system, possibly infection | 67 | 1.93 (1.34-2.78) | 7.95% |
| ESDD | I10→Y40 | Primary hypertension | External causes of morbidity related to medical treatment | 82 | 1.89 (1.36-2.63) | 10.28% |
| ESDD | I25→I95 | Chronic ischaemic heart disease | Hypotension | 51 | 7.00 (3.34-14.68) | 12.68% |
| ESDD | I25→J12 | Chronic ischaemic heart disease | Pneumonia | 72 | 5.50 (3.11-9.72) | 12.26% |
| ESDD | I48→J12 | Atrial fibrillation and flutter | Pneumonia | 75 | 7.58 (4.15-13.84) | 17.97% |
| ESDD | I51→J12 | Complications and ill-defined descriptions of heart disease | Pneumonia | 53 | 28.50 (6.96-116.74) | 16.30% |
| ESDD | J44→J12 | Chronic obstructive pulmonary disease | Pneumonia | 54 | 4.92 (2.64-9.15) | 5.00% |
| ESDD | K29→A41 | Gastritis and duodenitis | Sepsis | 51 | 5.89 (2.91-11.94) | 1.69% |
| ESDD | K29→J12 | Gastritis and duodenitis | Pneumonia | 69 | 10.43 (4.80-22.65) | 2.22% |
| ESDD | L01→J12 | Skin and subcutaneous infections | Pneumonia | 54 | 12.60 (5.07-31.32) | 8.33% |
| ESDD | M10→J12 | Other inflammatory arthritis | Pneumonia | 67 | 7.40 (3.82-14.32) | 9.89% |
| ESDD | N18→A41 | Chronic kidney disease | Sepsis | 63 | 5.23 (2.89-9.47) | 13.76% |
| ESDD | N18→J12 | Chronic kidney disease | Pneumonia | 93 | 6.29 (3.77-10.50) | 11.88% |
| ESDD | Y40→J12 | External causes of morbidity related to medical treatment | Pneumonia | 88 | 4.12 (2.66-6.38) | 10.77% |
| GSDD | D10→A41 | Benign tumors | Sepsis | 44 | 10.20 (4.07-25.56) | 1.79% |
| GSDD | D10→E87 | Benign tumors | Other disorders of fluid, electrolyte and acid-base balance | 44 | 5.89 (2.91-11.94) | 0.00% |
| GSDD | D10→J12 | Benign tumors | Pneumonia | 44 | 4.36 (2.27-8.40) | 0.00% |
| GSDD | D50→A41 | Iron deficiency anaemia | Sepsis | 47 | 17.33 (5.41-55.50) | 3.17% |
| GSDD | E10→A41 | Diabetes | Sepsis | 49 | 3.19 (1.82-5.59) | 8.06% |
| GSDD | E10→E87 | Diabetes | Other disorders of fluid, electrolyte and acid-base balance | 66 | 7.50 (3.88-14.51) | 4.94% |
| GSDD | E10→J12 | Diabetes | Pneumonia | 53 | 5.36 (2.82-10.21) | 12.86% |
| GSDD | E10→N18 | Diabetes | Chronic kidney disease | 71 | 4.11 (2.46-6.88) | 16.96% |
| GSDD | E10→N39 | Diabetes | Disorders of urinary system, possibly infection | 40 | 3.31 (1.78-6.15) | 7.14% |
| GSDD | E10→Y40 | Diabetes | External causes of morbidity related to medical treatment | 45 | 10.40 (4.15-26.04) | 0.00% |
| GSDD | E66→A41 | Obesity | Sepsis | 52 | 9.00 (3.87-20.92) | 4.69% |
| GSDD | E66→E87 | Obesity | Other disorders of fluid, electrolyte and acid-base balance | 58 | 4.46 (2.45-8.14) | 11.90% |
| GSDD | E66→J12 | Obesity | Pneumonia | 49 | 6.75 (3.21-14.18) | 5.48% |
| GSDD | E78→A41 | Disorders of lipoprotein metabolism and other lipidaemias | Sepsis | 58 | 3.47 (2.09-5.79) | 10.67% |
| GSDD | E78→E87 | Disorders of lipoprotein metabolism and other lipidaemias | Other disorders of fluid, electrolyte and acid-base balance | 94 | 3.31 (2.23-4.92) | 8.47% |
| GSDD | E78→I48 | Disorders of lipoprotein metabolism and other lipidaemias | Atrial fibrillation and flutter | 46 | 2.55 (1.52-4.28) | 14.86% |
| GSDD | E78→I51 | Disorders of lipoprotein metabolism and other lipidaemias | Complications and ill-defined descriptions of heart disease | 42 | 3.75 (1.98-7.09) | 6.35% |
| GSDD | E78→J12 | Disorders of lipoprotein metabolism and other lipidaemias | Pneumonia | 71 | 4.76 (2.82-8.04) | 7.61% |
| GSDD | E78→J20 | Disorders of lipoprotein metabolism and other lipidaemias | Other acute Lower respiratory infections | 36 | 3.45 (1.77-6.76) | 8.16% |
| GSDD | E78→N18 | Disorders of lipoprotein metabolism and other lipidaemias | Chronic kidney disease | 86 | 2.94 (1.97-4.39) | 11.28% |
| GSDD | E78→Y40 | Disorders of lipoprotein metabolism and other lipidaemias | External causes of morbidity related to medical treatment | 48 | 4.46 (2.45-8.14) | 8.22% |
| GSDD | F17→E87 | Tobacco abuse | Other disorders of fluid, electrolyte and acid-base balance | 38 | 4.56 (2.21-9.37) | 9.80% |
| GSDD | H25→A41 | Disorders of the lens | Sepsis | 47 | 5.40 (2.75-10.60) | 3.39% |
| GSDD | H25→E87 | Disorders of the lens | Other disorders of fluid, electrolyte and acid-base balance | 70 | 7.27 (3.87-13.66) | 2.30% |
| GSDD | H25→J12 | Disorders of the lens | Pneumonia | 60 | 6.90 (3.55-13.39) | 0.00% |
| GSDD | H25→W00 | Disorders of the lens | Falls | 36 | 5.29 (2.36-11.86) | 0.00% |
| GSDD | H30→E87 | Disorders of choroid and retina | Other disorders of fluid, electrolyte and acid-base balance | 36 | 9.25 (3.30-25.95) | 2.13% |
| GSDD | I10→E78 | Primary hypertension | Disorders of lipoprotein metabolism and other lipidaemias | 53 | 2.09 (1.39-3.12) | 32.98% |
| GSDD | I10→E87 | Primary hypertension | Other disorders of fluid, electrolyte and acid-base balance | 123 | 2.13 (1.63-2.79) | 11.33% |
| GSDD | I10→J12 | Primary hypertension | Pneumonia | 102 | 2.26 (1.67-3.06) | 7.14% |
| GSDD | I10→Y40 | Primary hypertension | External causes of morbidity related to medical treatment | 79 | 2.10 (1.50-2.94) | 9.18% |
| GSDD | I20→A41 | Angina pectoris | Sepsis | 41 | 5.33 (2.62-10.87) | 4.00% |
| GSDD | I20→E87 | Angina pectoris | Other disorders of fluid, electrolyte and acid-base balance | 44 | 6.86 (3.10-15.15) | 8.47% |
| GSDD | I25→A41 | Chronic ischaemic heart disease | Sepsis | 55 | 5.08 (2.74-9.44) | 13.51% |
| GSDD | I25→E87 | Chronic ischaemic heart disease | Other disorders of fluid, electrolyte and acid-base balance | 67 | 6.08 (3.38-10.93) | 12.00% |
| GSDD | I25→J12 | Chronic ischaemic heart disease | Pneumonia | 55 | 7.62 (3.65-15.93) | 11.49% |
| GSDD | I25→J20 | Chronic ischaemic heart disease | Other acute Lower respiratory infections | 34 | 3.60 (1.79-7.25) | 4.17% |
| GSDD | I25→T80 | Chronic ischaemic heart disease | Complications due to medical treatment | 35 | 5.83 (2.45-13.87) | 10.20% |
| GSDD | I48→A41 | Atrial fibrillation and flutter | Sepsis | 67 | 3.12 (1.99-4.90) | 17.43% |
| GSDD | I48→E87 | Atrial fibrillation and flutter | Other disorders of fluid, electrolyte and acid-base balance | 79 | 5.76 (3.44-9.65) | 19.72% |
| GSDD | I48→J12 | Atrial fibrillation and flutter | Pneumonia | 72 | 4.15 (2.55-6.76) | 21.80% |
| GSDD | I48→L89 | Atrial fibrillation and flutter | Pressure ulcer | 35 | 3.55 (1.82-6.92) | 12.77% |
| GSDD | I49→A41 | Other cardiac arrhythmias | Sepsis | 54 | 8.43 (3.85-18.45) | 7.89% |
| GSDD | I49→E87 | Other cardiac arrhythmias | Other disorders of fluid, electrolyte and acid-base balance | 60 | 13.40 (5.40-33.24) | 14.43% |
| GSDD | J20→A41 | Other acute Lower respiratory infections | Sepsis | 41 | 5.33 (2.62-10.87) | 6.90% |
| GSDD | J20→E87 | Other acute Lower respiratory infections | Other disorders of fluid, electrolyte and acid-base balance | 48 | 2.88 (1.66-5.00) | 12.99% |
| GSDD | J44→A41 | Chronic obstructive pulmonary disease | Sepsis | 39 | 7.50 (3.20-17.58) | 11.76% |
| GSDD | J44→E87 | Chronic obstructive pulmonary disease | Other disorders of fluid, electrolyte and acid-base balance | 50 | 4.82 (2.52-9.22) | 9.21% |
| GSDD | K59→A41 | Other functional intestinal disorders | Sepsis | 48 | 7.86 (3.58-17.25) | 9.72% |
| GSDD | L01→A41 | Skin and subcutaneous infections | Sepsis | 45 | 6.86 (3.10-15.15) | 16.18% |
| GSDD | M10→A41 | Other inflammatory arthritis | Sepsis | 48 | 3.64 (2.02-6.58) | 9.38% |
| GSDD | M10→E87 | Other inflammatory arthritis | Other disorders of fluid, electrolyte and acid-base balance | 65 | 8.00 (4.00-16.00) | 9.78% |
| GSDD | M10→J12 | Other inflammatory arthritis | Pneumonia | 61 | 7.22 (3.60-14.50) | 15.91% |
| GSDD | M10→T80 | Other inflammatory arthritis | Complications due to medical treatment | 35 | 8.75 (3.11-24.62) | 4.08% |
| GSDD | M10→W00 | Other inflammatory arthritis | Falls | 36 | 6.17 (2.60-14.61) | 2.04% |
| GSDD | M15→A41 | Osteoarthritis | Sepsis | 60 | 3.72 (2.21-6.26) | 5.56% |
| GSDD | M15→E87 | Osteoarthritis | Other disorders of fluid, electrolyte and acid-base balance | 72 | 3.50 (2.22-5.51) | 14.56% |
| GSDD | M15→I51 | Osteoarthritis | Complications and ill-defined descriptions of heart disease | 42 | 2.75 (1.55-4.87) | 6.78% |
| GSDD | M15→J12 | Osteoarthritis | Pneumonia | 68 | 5.53 (3.19-9.59) | 11.36% |
| GSDD | M15→W00 | Osteoarthritis | Falls | 44 | 4.08 (2.17-7.68) | 8.20% |
| GSDD | M79→A41 | Other soft tissue disorders, not elsewhere classified | Sepsis | 37 | 13.67 (4.23-44.13) | 2.04% |
| GSDD | M79→E87 | Other soft tissue disorders, not elsewhere classified | Other disorders of fluid, electrolyte and acid-base balance | 45 | 12.00 (4.33-33.28) | 8.06% |
| GSDD | N18→A09 | Chronic kidney disease | Infectious gastroenteritis and colitis | 52 | 4.08 (2.22-7.48) | 8.43% |
| GSDD | N18→A41 | Chronic kidney disease | Sepsis | 103 | 4.07 (2.67-6.21) | 15.92% |
| GSDD | N18→E87 | Chronic kidney disease | Other disorders of fluid, electrolyte and acid-base balance | 140 | 5.64 (3.78-8.43) | 18.38% |
| GSDD | N18→I34 | Chronic kidney disease | Non-rheumatic valve disorders | 58 | 5.27 (2.77-10.05) | 8.79% |
| GSDD | N18→J12 | Chronic kidney disease | Pneumonia | 125 | 6.09 (3.92-9.46) | 14.29% |
| GSDD | N18→L89 | Chronic kidney disease | Pressure ulcer | 42 | 3.00 (1.67-5.38) | 0.00% |
| GSDD | N18→W00 | Chronic kidney disease | Falls | 63 | 4.71 (2.65-8.39) | 13.46% |
| GSDD | N39→A41 | Disorders of urinary system, possibly infection | Sepsis | 62 | 3.50 (2.13-5.75) | 24.58% |
| GSDD | W00→A41 | Falls | Sepsis | 51 | 2.43 (1.50-3.96) | 7.89% |
| GSDD | Y40→A41 | External causes of morbidity related to medical treatment | Sepsis | 69 | 2.80 (1.85-4.25) | 16.98% |
| GSDD | Y40→E87 | External causes of morbidity related to medical treatment | Other disorders of fluid, electrolyte and acid-base balance | 74 | 3.70 (2.33-5.86) | 15.79% |
| MND | E78→E87 | Disorders of lipoprotein metabolism and other lipidaemias | Other disorders of fluid, electrolyte and acid-base balance | 231 | 3.33 (2.58-4.31) | 8.48% |
| MND | E78→J12 | Disorders of lipoprotein metabolism and other lipidaemias | Pneumonia | 231 | 3.54 (2.73-4.60) | 9.06% |
| MND | E78→N17 | Disorders of lipoprotein metabolism and other lipidaemias | Acute renal failure | 256 | 4.06 (3.13-5.26) | 8.09% |
| MND | E78→Y40 | Disorders of lipoprotein metabolism and other lipidaemias | External causes of morbidity related to medical treatment | 226 | 3.00 (2.33-3.86) | 7.67% |
| MND | I10→A41 | Primary hypertension | Sepsis | 367 | 1.66 (1.42-1.94) | 7.05% |
| MND | I10→D51 | Primary hypertension | Other anaemias | 361 | 1.58 (1.35-1.85) | 9.29% |
| MND | I10→E78 | Primary hypertension | Disorders of lipoprotein metabolism and other lipidaemias | 238 | 1.97 (1.62-2.41) | 42.86% |
| MND | I10→E87 | Primary hypertension | Other disorders of fluid, electrolyte and acid-base balance | 390 | 2.01 (1.71-2.36) | 11.02% |
| MND | I10→J12 | Primary hypertension | Pneumonia | 429 | 1.99 (1.71-2.31) | 9.22% |
| MND | I10→J90 | Primary hypertension | Pleural effusion | 324 | 1.51 (1.28-1.78) | 8.84% |
| MND | I10→K59 | Primary hypertension | Other functional intestinal disorders | 316 | 1.49 (1.26-1.76) | 7.57% |
| MND | I10→N17 | Primary hypertension | Acute renal failure | 423 | 2.01 (1.72-2.34) | 10.45% |
| MND | I10→N39 | Primary hypertension | Disorders of urinary system, possibly infection | 270 | 1.47 (1.23-1.76) | 10.53% |
| MND | I10→Y40 | Primary hypertension | External causes of morbidity related to medical treatment | 416 | 1.62 (1.39-1.88) | 8.49% |
| MND | Y40→A41 | External causes of morbidity related to medical treatment | Sepsis | 253 | 3.01 (2.37-3.83) | 24.27% |
| MND | Y40→E87 | External causes of morbidity related to medical treatment | Other disorders of fluid, electrolyte and acid-base balance | 245 | 3.22 (2.51-4.13) | 22.40% |
| MND | Y40→J12 | External causes of morbidity related to medical treatment | Pneumonia | 265 | 3.81 (2.96-4.89) | 12.78% |
| MND | Y40→N17 | External causes of morbidity related to medical treatment | Acute renal failure | 257 | 3.43 (2.68-4.38) | 19.21% |
| RSDD | C77→J12 | Metastatic cancer | Pneumonia | 144 | 1.93 (1.49-2.50) | 13.27% |
| RSDD | D50→J12 | Iron deficiency anaemia | Pneumonia | 115 | 9.31 (5.25-16.49) | 6.25% |
| RSDD | D51→E87 | Other anaemias | Other disorders of fluid, electrolyte and acid-base balance | 135 | 3.33 (2.39-4.65) | 16.38% |
| RSDD | D51→J12 | Other anaemias | Pneumonia | 176 | 3.96 (2.90-5.40) | 12.63% |
| RSDD | E10→E87 | Diabetes | Other disorders of fluid, electrolyte and acid-base balance | 117 | 4.59 (3.07-6.85) | 12.27% |
| RSDD | E10→J12 | Diabetes | Pneumonia | 186 | 4.35 (3.16-5.99) | 13.78% |
| RSDD | E10→N17 | Diabetes | Acute renal failure | 133 | 5.21 (3.48-7.81) | 11.36% |
| RSDD | E66→J12 | Obesity | Pneumonia | 145 | 6.87 (4.44-10.64) | 15.56% |
| RSDD | E66→N17 | Obesity | Acute renal failure | 112 | 8.71 (5.01-15.15) | 11.93% |
| RSDD | E78→E87 | Disorders of lipoprotein metabolism and other lipidaemias | Other disorders of fluid, electrolyte and acid-base balance | 173 | 2.62 (1.99-3.44) | 10.04% |
| RSDD | E78→I50 | Disorders of lipoprotein metabolism and other lipidaemias | Heart failure | 109 | 2.93 (2.05-4.17) | 10.98% |
| RSDD | E78→I95 | Disorders of lipoprotein metabolism and other lipidaemias | Hypotension | 104 | 2.47 (1.74-3.49) | 8.33% |
| RSDD | E78→J12 | Disorders of lipoprotein metabolism and other lipidaemias | Pneumonia | 247 | 4.19 (3.21-5.47) | 10.87% |
| RSDD | E78→N17 | Disorders of lipoprotein metabolism and other lipidaemias | Acute renal failure | 167 | 3.47 (2.56-4.71) | 14.10% |
| RSDD | F17→E87 | Tobacco abuse | Other disorders of fluid, electrolyte and acid-base balance | 112 | 5.55 (3.52-8.73) | 13.89% |
| RSDD | F17→J12 | Tobacco abuse | Pneumonia | 145 | 5.03 (3.42-7.40) | 13.33% |
| RSDD | F32→J12 | Depression | Pneumonia | 114 | 6.05 (3.77-9.71) | 8.88% |
| RSDD | I10→C77 | Primary hypertension | Metastatic cancer | 107 | 2.10 (1.58-2.80) | 15.86% |
| RSDD | I10→E78 | Primary hypertension | Disorders of lipoprotein metabolism and other lipidaemias | 131 | 2.18 (1.67-2.84) | 40.77% |
| RSDD | I10→E87 | Primary hypertension | Other disorders of fluid, electrolyte and acid-base balance | 259 | 1.80 (1.49-2.16) | 14.07% |
| RSDD | I10→I48 | Primary hypertension | Atrial fibrillation and flutter | 166 | 1.58 (1.25-1.99) | 17.65% |
| RSDD | I10→J12 | Primary hypertension | Pneumonia | 403 | 2.02 (1.73-2.36) | 9.67% |
| RSDD | I10→N17 | Primary hypertension | Acute renal failure | 262 | 1.88 (1.56-2.27) | 11.14% |
| RSDD | I10→Y40 | Primary hypertension | External causes of morbidity related to medical treatment | 150 | 1.79 (1.40-2.29) | 11.36% |
| RSDD | I20→J12 | Angina pectoris | Pneumonia | 103 | 7.43 (4.25-12.98) | 7.25% |
| RSDD | I25→E87 | Chronic ischaemic heart disease | Other disorders of fluid, electrolyte and acid-base balance | 117 | 7.11 (4.34-11.65) | 10.38% |
| RSDD | I25→J12 | Chronic ischaemic heart disease | Pneumonia | 152 | 4.94 (3.38-7.22) | 12.96% |
| RSDD | I25→N17 | Chronic ischaemic heart disease | Acute renal failure | 116 | 4.92 (3.23-7.50) | 11.11% |
| RSDD | I48→E87 | Atrial fibrillation and flutter | Other disorders of fluid, electrolyte and acid-base balance | 124 | 5.19 (3.43-7.83) | 24.48% |
| RSDD | I48→I50 | Atrial fibrillation and flutter | Heart failure | 114 | 4.19 (2.83-6.20) | 25.32% |
| RSDD | I48→J12 | Atrial fibrillation and flutter | Pneumonia | 178 | 6.86 (4.61-10.19) | 24.66% |
| RSDD | I48→N17 | Atrial fibrillation and flutter | Acute renal failure | 127 | 6.09 (3.92-9.46) | 25.19% |
| RSDD | J44→E87 | Chronic obstructive pulmonary disease | Other disorders of fluid, electrolyte and acid-base balance | 136 | 4.93 (3.33-7.30) | 17.05% |
| RSDD | J44→I50 | Chronic obstructive pulmonary disease | Heart failure | 110 | 3.66 (2.47-5.41) | 21.35% |
| RSDD | J44→J12 | Chronic obstructive pulmonary disease | Pneumonia | 214 | 5.95 (4.24-8.35) | 17.97% |
| RSDD | J44→N17 | Chronic obstructive pulmonary disease | Acute renal failure | 142 | 5.17 (3.49-7.64) | 17.39% |
| RSDD | J45→J12 | Asthma | Pneumonia | 122 | 15.75 (7.71-32.18) | 9.68% |
| RSDD | K21→J12 | Gastro-oesophageal reflux disease | Pneumonia | 127 | 6.38 (4.03-10.11) | 9.09% |
| RSDD | K29→J12 | Gastritis and duodenitis | Pneumonia | 141 | 4.93 (3.33-7.30) | 3.23% |
| RSDD | K44→J12 | Diaphragmatic hernia | Pneumonia | 141 | 9.67 (5.68-16.45) | 1.12% |
| RSDD | L01→J12 | Skin and subcutaneous infections | Pneumonia | 110 | 6.94 (4.17-11.54) | 9.34% |
| RSDD | M10→E87 | Other inflammatory arthritis | Other disorders of fluid, electrolyte and acid-base balance | 104 | 10.00 (5.52-18.10) | 10.34% |
| RSDD | M10→J12 | Other inflammatory arthritis | Pneumonia | 157 | 7.90 (5.02-12.45) | 11.63% |
| RSDD | M20→J12 | Other joint disorders | Pneumonia | 106 | 9.08 (5.00-16.49) | 9.30% |
| RSDD | M54→J12 | Dorsalgia | Pneumonia | 101 | 21.20 (8.65-51.99) | 7.33% |
| RSDD | N18→E87 | Chronic kidney disease | Other disorders of fluid, electrolyte and acid-base balance | 106 | 6.21 (3.83-10.08) | 23.70% |
| RSDD | N18→J12 | Chronic kidney disease | Pneumonia | 165 | 6.59 (4.40-9.88) | 15.30% |
| RSDD | N39→J12 | Disorders of urinary system, possibly infection | Pneumonia | 167 | 3.61 (2.65-4.92) | 12.54% |
| RSDD | T80→J12 | Complications due to medical treatment | Pneumonia | 127 | 7.67 (4.69-12.53) | 11.42% |
| RSDD | W00→J12 | Falls | Pneumonia | 150 | 4.74 (3.29-6.83) | 12.02% |
| RSDD | Y40→E87 | External causes of morbidity related to medical treatment | Other disorders of fluid, electrolyte and acid-base balance | 120 | 3.07 (2.18-4.31) | 23.04% |
| RSDD | Y40→J12 | External causes of morbidity related to medical treatment | Pneumonia | 210 | 4.44 (3.31-5.97) | 13.06% |
| RSDD | Y40→N17 | External causes of morbidity related to medical treatment | Acute renal failure | 138 | 4.00 (2.84-5.63) | 18.73% |

^*^Combined ICD-10 codes originates from the original ICD-10 code and are displayed in the Additional file 1.

^#^Number of the participants experienced the corresponding temporal disease trajectories leading to causes of death.

**^&^**Number of individuals with high genetic susceptibility to MAFLD having the same D1 and D2 diagnosis date divided by the high genetic risk individuals diagnosed with both D1 and D2. A high value indicates a strong correlation between the two diseases and may exist a reverse causal relationship.

OR, odds ratio; CI, confidence interval. MAFLD, metabolic dysfunction-associated fatty liver disease; MND, malignant neoplasms death; ESDD, endocrine system disease death; CVDD, cardiovascular disease death; RSDD, respiratory system disease death; DSSD, digestive system disease death; GSDD, genitourinary system disease death; UCD, unnatural cause death.

Additional file 1: Table S29. Temporal disease pairs with a significantly increased risk of disease 2 (D2) after disease 1 (D1) in individuals with alcoholic liver disease.

| **D1→D2 code^*^** | **D1 description** | **D2 description** | **No.^#^** | **Relative risk** |
| --- | --- | --- | --- | --- |
| A04→Y99 | Bacterial Intestinal Infections | Death | 12700 | 1.37 |
| A41→Y99 | Sepsis | Death | 99090 | 1.56 |
| A49→Y99 | Bacterial Infection Of Unspecified Site | Death | 28413 | 1.47 |
| B37→Y99 | Candidiasis | Death | 15428 | 1.52 |
| C22→Y99 | Malignant Neoplasm Of Liver And Intrahepatic Bile | Death | 9029 | 2.92 |
| D62→A41 | Acute Posthaemorrhagic Anaemia | Sepsis | 2393 | 1.36 |
| D62→Y99 | Acute Posthaemorrhagic Anaemia | Death | 16093 | 1.22 |
| E13→Y99 | Specified Diabetes Mellitus | Death | 3356 | 1.75 |
| E14→A41 | Diabetes Mellitus | Sepsis | 5053 | 2.03 |
| E14→Y99 | Diabetes Mellitus | Death | 22946 | 1.38 |
| E41→Y99 | Nutritional Marasmus | Death | 3561 | 2.04 |
| E64→Y99 | Sequelae Of Malnutrition And Nutritional Deficien | Death | 9145 | 1.85 |
| E86→Y99 | Volume Depletion | Death | 141474 | 1.38 |
| E87→Y99 | Disorders Of Fluid, Electrolyte And Acid-Base Bal | Death | 58161 | 1.34 |
| F03→Y99 | Dementia | Death | 72375 | 1.36 |
| F05→Y99 | Delirium | Death | 20648 | 1.48 |
| G31→Y99 | Degenerative Diseases Of Nervous System | Death | 6471 | 1.70 |
| G41→Y99 | Status Epilepticus | Death | 2771 | 1.93 |
| G93→Y99 | Disorders Of Brain | Death | 6866 | 2.15 |
| G96→Y99 | Disorders Of Central Nervous System | Death | 9669 | 1.23 |
| I61→I69 | Intracerebral Haemorrhage | Sequelae Of Cerebrovascular Disease | 12146 | 5.10 |
| I61→Y99 | Intracerebral Haemorrhage | Death | 28943 | 1.47 |
| I64→A41 | Stroke | Sepsis | 9252 | 1.29 |
| I64→I69 | Stroke | Sequelae Of Cerebrovascular Disease | 53645 | 5.32 |
| I64→J18 | Stroke | Pneumonia, Organism Unspecified | 26796 | 1.49 |
| I64→Y99 | Stroke | Death | 100501 | 1.46 |
| I69→Y99 | Sequelae Of Cerebrovascular Disease | Death | 81644 | 1.23 |
| I95→Y99 | Hypotension | Death | 18222 | 1.37 |
| I98→Y99 | Disorders Of Circulatory System In Diseases Class | Death | 1266 | 2.66 |
| J15→Y99 | Bacterial Pneumonia | Death | 80836 | 1.44 |
| J18→Y99 | Pneumonia, Organism Unspecified | Death | 256611 | 1.37 |
| J69→Y99 | Pneumonitis Due To Solids And Liquids | Death | 12333 | 1.89 |
| J81→Y99 | Pulmonary Oedema | Death | 24464 | 1.54 |
| J90→Y99 | Pleural Effusion | Death | 21895 | 1.76 |
| J91→Y99 | Pleural Effusion Classified Elsewhere | Death | 10756 | 1.94 |
| J96→Y99 | Respiratory Failure | Death | 94101 | 1.96 |
| K02→Y99 | Dental Caries | Death | 6826 | 1.90 |
| K04→Y99 | Diseases Of Pulp And Periapical Tissues | Death | 7377 | 1.27 |
| K05→Y99 | Gingivitis And Periodontal Diseases | Death | 8931 | 1.56 |
| K42→K72 | Umbilical Hernia | Hepatic Failure | 383 | 2.36 |
| K63→Y99 | Diseases Of Intestine | Death | 9087 | 1.33 |
| K65→E86 | Peritonitis | Volume Depletion | 2043 | 1.52 |
| K65→Y99 | Peritonitis | Death | 13274 | 1.60 |
| K70→A04 | Alcoholic Liver Disease | Bacterial Intestinal Infections | 688 | 3.66 |
| K70→A41 | Alcoholic Liver Disease | Sepsis | 3747 | 2.65 |
| K70→A49 | Alcoholic Liver Disease | Bacterial Infection Of Unspecified Site | 882 | 1.79 |
| K70→B37 | Alcoholic Liver Disease | Candidiasis | 636 | 2.64 |
| K70→C06 | Alcoholic Liver Disease | Unspecified Malignant Neoplasm | 147 | 1.67 |
| K70→C10 | Alcoholic Liver Disease | Malignant Neoplasm Of Oropharynx | 146 | 1.99 |
| K70→C13 | Alcoholic Liver Disease | Malignant Neoplasm Of Hypopharynx | 116 | 2.05 |
| K70→C22 | Alcoholic Liver Disease | Malignant Neoplasm Of Liver And Intrahepatic Bile | 1051 | 12.89 |
| K70→D62 | Alcoholic Liver Disease | Acute Posthaemorrhagic Anaemia | 973 | 5.58 |
| K70→E13 | Alcoholic Liver Disease | Specified Diabetes Mellitus | 333 | 3.24 |
| K70→E14 | Alcoholic Liver Disease | Diabetes Mellitus | 950 | 1.84 |
| K70→E41 | Alcoholic Liver Disease | Nutritional Marasmus | 213 | 3.68 |
| K70→E64 | Alcoholic Liver Disease | Sequelae Of Malnutrition And Nutritional Deficien | 409 | 2.56 |
| K70→E85 | Alcoholic Liver Disease | Amyloidosis | 140 | 8.22 |
| K70→E86 | Alcoholic Liver Disease | Volume Depletion | 3078 | 3.16 |
| K70→E87 | Alcoholic Liver Disease | Disorders Of Fluid, Electrolyte And Acid-Base Bal | 3082 | 3.62 |
| K70→F03 | Alcoholic Liver Disease | Dementia | 713 | 2.50 |
| K70→F05 | Alcoholic Liver Disease | Delirium | 708 | 3.26 |
| K70→G31 | Alcoholic Liver Disease | Degenerative Diseases Of Nervous System | 800 | 11.20 |
| K70→G41 | Alcoholic Liver Disease | Status Epilepticus | 170 | 3.39 |
| K70→G93 | Alcoholic Liver Disease | Disorders Of Brain | 557 | 7.66 |
| K70→G96 | Alcoholic Liver Disease | Disorders Of Central Nervous System | 403 | 1.20 |
| K70→I46 | Alcoholic Liver Disease | Cardiac Arrest | 670 | 1.69 |
| K70→I61 | Alcoholic Liver Disease | Intracerebral Haemorrhage | 593 | 2.62 |
| K70→I64 | Alcoholic Liver Disease | Stroke | 1121 | 1.62 |
| K70→I86 | Alcoholic Liver Disease | Varicose Veins Of Sites | 495 | 14.52 |
| K70→I95 | Alcoholic Liver Disease | Hypotension | 558 | 1.99 |
| K70→I98 | Alcoholic Liver Disease | Disorders Of Circulatory System In Diseases Class | 979 | 36.47 |
| K70→J15 | Alcoholic Liver Disease | Bacterial Pneumonia | 1768 | 1.44 |
| K70→J22 | Alcoholic Liver Disease | Acute Lower Respiratory Infection | 170 | 1.37 |
| K70→J69 | Alcoholic Liver Disease | Pneumonitis Due To Solids And Liquids | 518 | 3.05 |
| K70→J81 | Alcoholic Liver Disease | Pulmonary Oedema | 319 | 1.64 |
| K70→J90 | Alcoholic Liver Disease | Pleural Effusion | 504 | 1.87 |
| K70→J91 | Alcoholic Liver Disease | Pleural Effusion Classified Elsewhere | 273 | 2.37 |
| K70→J96 | Alcoholic Liver Disease | Respiratory Failure | 2635 | 1.76 |
| K70→K02 | Alcoholic Liver Disease | Dental Caries | 392 | 2.44 |
| K70→K04 | Alcoholic Liver Disease | Diseases Of Pulp And Periapical Tissues | 310 | 1.21 |
| K70→K05 | Alcoholic Liver Disease | Gingivitis And Periodontal Diseases | 368 | 1.45 |
| K70→K42 | Alcoholic Liver Disease | Umbilical Hernia | 1094 | 5.14 |
| K70→K55 | Alcoholic Liver Disease | Vascular Disorders Of Intestine | 211 | 1.85 |
| K70→K63 | Alcoholic Liver Disease | Diseases Of Intestine | 345 | 1.84 |
| K70→K65 | Alcoholic Liver Disease | Peritonitis | 1993 | 8.67 |
| K70→K71 | Alcoholic Liver Disease | Toxic Liver Disease | 1082 | 16.65 |
| K70→K72 | Alcoholic Liver Disease | Hepatic Failure | 6325 | 30.51 |
| K70→K74 | Alcoholic Liver Disease | Fibrosis And Cirrhosis Of Liver | 5179 | 23.10 |
| K70→L89 | Alcoholic Liver Disease | Decubitus Ulcer And Pressure Area | 524 | 2.15 |
| K70→L97 | Alcoholic Liver Disease | Ulcer Of Lower Limb | 514 | 1.55 |
| K70→M80 | Alcoholic Liver Disease | Osteoporosis With Pathological Fracture | 569 | 2.15 |
| K70→M81 | Alcoholic Liver Disease | Osteoporosis Without Pathological Fracture | 981 | 1.45 |
| K70→N17 | Alcoholic Liver Disease | Acute Renal Failure | 1241 | 3.22 |
| K70→N18 | Alcoholic Liver Disease | Chronic Kidney Disease | 817 | 1.25 |
| K70→N19 | Alcoholic Liver Disease | Unspecified Kidney Failure | 695 | 1.47 |
| K70→N30 | Alcoholic Liver Disease | Cystitis | 2156 | 1.66 |
| K70→N39 | Alcoholic Liver Disease | Disorders Of Urinary System | 1657 | 1.57 |
| K70→Y99 | Alcoholic Liver Disease | Death | 21871 | 2.93 |
| K71→Y99 | Toxic Liver Disease | Death | 2710 | 1.53 |
| K74→A41 | Fibrosis And Cirrhosis Of Liver | Sepsis | 1606 | 2.41 |
| K74→C22 | Fibrosis And Cirrhosis Of Liver | Malignant Neoplasm Of Liver And Intrahepatic Bile | 896 | 24.18 |
| K74→E86 | Fibrosis And Cirrhosis Of Liver | Volume Depletion | 1454 | 2.30 |
| K74→G93 | Fibrosis And Cirrhosis Of Liver | Disorders Of Brain | 329 | 9.90 |
| K74→K72 | Fibrosis And Cirrhosis Of Liver | Hepatic Failure | 2958 | 40.56 |
| K74→Y99 | Fibrosis And Cirrhosis Of Liver | Death | 10457 | 2.34 |
| L89→Y99 | Decubitus Ulcer And Pressure Area | Death | 21232 | 1.74 |
| L97→Y99 | Ulcer Of Lower Limb | Death | 22617 | 1.47 |
| M80→Y99 | Osteoporosis With Pathological Fracture | Death | 31191 | 1.47 |
| M81→M80 | Osteoporosis Without Pathological Fracture | Osteoporosis With Pathological Fracture | 14268 | 3.60 |
| M81→Y99 | Osteoporosis Without Pathological Fracture | Death | 46593 | 1.56 |
| N17→Y99 | Acute Renal Failure | Death | 25205 | 1.68 |
| N18→Y99 | Chronic Kidney Disease | Death | 44624 | 1.75 |
| N19→Y99 | Unspecified Kidney Failure | Death | 30055 | 1.72 |
| N30→A41 | Cystitis | Sepsis | 25299 | 1.93 |
| N30→J15 | Cystitis | Bacterial Pneumonia | 14748 | 1.22 |
| N30→K72 | Cystitis | Hepatic Failure | 959 | 1.24 |
| N30→Y99 | Cystitis | Death | 128617 | 1.21 |
| N39→J15 | Disorders Of Urinary System | Bacterial Pneumonia | 12423 | 1.25 |
| N39→J96 | Disorders Of Urinary System | Respiratory Failure | 9865 | 1.37 |
| N39→Y99 | Disorders Of Urinary System | Death | 87756 | 1.38 |

^*^Original ICD-10 code recorded in the Additional file 1;

^#^Number of the participants experienced the corresponding temporal disease trajectories.

Additional file 1: Table S30. Temporal disease pairs with a significantly increased risk of disease 2 (D2) after disease 1 (D1) in individuals with other alcoholic liver diseases.

| **D1→D2 code^*^** | **D1 description** | **D2 description** | **No.^#^** | **Relative risk** |
| --- | --- | --- | --- | --- |
| A41→Y99 | Sepsis | Death | 99090 | 1.56 |
| A49→Y99 | Bacterial Infection Of Unspecified Site | Death | 28413 | 1.47 |
| C22→Y99 | Malignant Neoplasm Of Liver And Intrahepatic Bile | Death | 9029 | 2.92 |
| C24→Y99 | Malignant Neoplasm Of Other And Unspecified Parts | Death | 4965 | 2.66 |
| C25→Y99 | Malignant Neoplasm Of Pancreas | Death | 21364 | 2.83 |
| E13→Y99 | Other Specified Diabetes Mellitus | Death | 3356 | 1.75 |
| E41→Y99 | Nutritional Marasmus | Death | 3561 | 2.04 |
| E86→Y99 | Volume Depletion | Death | 141474 | 1.38 |
| F05→Y99 | Delirium | Death | 20648 | 1.48 |
| I61→I69 | Intracerebral Haemorrhage | Sequelae Of Cerebrovascular Disease | 12146 | 5.10 |
| I61→Y99 | Intracerebral Haemorrhage | Death | 28943 | 1.47 |
| J96→Y99 | Respiratory Failure | Death | 94101 | 1.96 |
| K76→A41 | Other Liver disease | Sepsis | 2036 | 1.91 |
| K76→A49 | Other Liver disease | Bacterial Infection Of Unspecified Site | 709 | 1.50 |
| K76→C22 | Other Liver disease | Malignant Neoplasm Of Liver And Intrahepatic Bile | 665 | 10.99 |
| K76→C24 | Other Liver disease | Malignant Neoplasm Of Other And Unspecified Parts | 190 | 4.17 |
| K76→C25 | Other Liver disease | Malignant Neoplasm Of Pancreas | 321 | 1.79 |
| K76→E13 | Other Liver disease | Other Specified Diabetes Mellitus | 185 | 3.04 |
| K76→E41 | Other Liver disease | Nutritional Marasmus | 178 | 1.89 |
| K76→E86 | Other Liver disease | Volume Depletion | 1665 | 1.70 |
| K76→F05 | Other Liver disease | Delirium | 330 | 1.73 |
| K76→I46 | Other Liver disease | Cardiac Arrest | 347 | 1.37 |
| K76→I61 | Other Liver disease | Intracerebral Haemorrhage | 252 | 1.41 |
| K76→J96 | Other Liver disease | Respiratory Failure | 1531 | 1.49 |
| K76→M80 | Other Liver disease | Osteoporosis With Pathological Fracture | 435 | 1.36 |
| K76→M81 | Other Liver disease | Osteoporosis Without Pathological Fracture | 968 | 1.22 |
| K76→N18 | Other Liver disease | Chronic Kidney Disease | 695 | 1.27 |
| K76→Y99 | Other Liver disease | Death | 9658 | 1.61 |
| M80→Y99 | Osteoporosis With Pathological Fracture | Death | 31191 | 1.37 |
| M81→A41 | Osteoporosis Without Pathological Fracture | Sepsis | 6785 | 1.23 |
| M81→E86 | Osteoporosis Without Pathological Fracture | Volume Depletion | 11888 | 1.32 |
| M81→E87 | Osteoporosis Without Pathological Fracture | Disorders Of Fluid, Electrolyte And Acid-Ba | 6640 | 1.39 |
| M81→J96 | Osteoporosis Without Pathological Fracture | Respiratory Failure | 7752 | 1.63 |
| M81→M80 | Osteoporosis Without Pathological Fracture | Osteoporosis With Pathological Fracture | 14268 | 3.60 |
| M81→Y99 | Osteoporosis Without Pathological Fracture | Death | 46593 | 1.26 |
| N18→Y99 | Chronic Kidney Disease | Death | 44624 | 1.75 |

^*^Original ICD-10 code recorded in the Additional file 1;

^#^Number of the participants experienced the corresponding temporal disease trajectories.
